# Exploring Passive Permeability Profiles of Cyclic Heptapeptide Chemical Space Uncovers Bioactivity of Mortiamide Scaffold Driven by Colloidal Aggregation

**DOI:** 10.1002/cbic.70329

**Published:** 2026-05-14

**Authors:** Jaru Taechalertpaisarn, Alexander Engstrom, Maria Sajimon, Beverley M. Rabbitts, Vitor H. Balasco Serrão, Satoshi Ono, Jevgenij A. Raskatov, Timothy C. Johnstone, R. Scott Lokey

**Affiliations:** ^1^ Department of Chemistry and Biochemistry University of California, Santa Cruz Santa Cruz California USA; ^2^ Department of Science Technology and Innovation Faculty of Science Chulabhorn Royal Academy Bangkok Thailand; ^3^ Biomolecular Cryo‐Electron Microscopy Facility University of California, Santa Cruz Santa Cruz California USA; ^4^ Discovery Technology Laboratories Innovative Research Division Mitsubishi Tanabe Pharma Corporation Yokohama Kanagawa Japan; ^5^ Xeureka, Inc Minato‐ku Tokyo Japan

**Keywords:** colloidal aggregation, macrocycles, membrane permeability, natural products, pan‐assay interference compounds

## Abstract

We investigated the passive permeability and cytotoxicity of cyclic heptapeptides based on the mortiamide family of natural products. Of all possible stereoisomeric backbones, the natural product's scaffold was among the two most lipophilic as measured by hydrocarbon‐water partition coefficients. Using one‐bead‐one‐compound synthesis, we generated a ∼66,000‐member library based on the mortiamide scaffold and identified numerous compounds that showed low micromolar cytotoxicity against synovial sarcoma and breast cancer cell lines. Physicochemical characterization revealed that these compounds, including the known mortiamides, have very low aqueous solubilities and form amyloid‐like fibril aggregates. Multiple lines of evidence—including detergent‐reversible enzyme inhibition, thioflavin T fluorescence, transmission electron microscopy, and equipotent enantiomeric pairs—demonstrate that the observed bioactivity arises from colloidal aggregation rather than specific target engagement. Our findings highlight the critical importance of early physicochemical evaluation in natural product‐inspired drug discovery and underscore how aggregation‐prone scaffolds can generate misleading structure–activity relationships.

## Graphical abstract

A comprehensive stereochemical and physicochemical survey of cyclic heptapeptide chemical space reveals that the mortiamide natural product scaffold possesses intrinsically low aqueous solubility and forms amyloid‐like fibril aggregates. Multiple orthogonal assays demonstrate that the observed cytotoxicity arises from colloidal aggregation rather than specific target engagement, underscoring the importance of early physicochemical profiling in natural product‐inspired drug discovery.

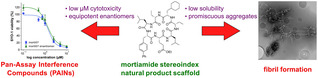

## 1 Introduction

Drug discovery programs are increasingly turning toward molecules that lie within the “beyond rule of 5” (bRo5) chemical space as therapeutic targets become increasingly more difficult to modulate using classic Ro5‐compliant small molecules [1, 2, 3, 4, 5]. Conventional small molecules may not interact effectively with many of the large, flat surfaces found in these challenging targets, particularly among those involving protein–protein interactions [6, 7, 8, 9]. Cyclic peptides have emerged as a promising class of molecules that combine the membrane permeability of small molecules with the extended target‐binding capabilities of larger biologics (proteins or antibodies) [10, 11, 12].

Cyclic peptides have distinct pharmacological advantages over their linear counterparts [9, 13, 14]. Cyclization generally increases binding affinity relative to linear peptides due to minimal entropic loss upon target binding owing to the rigidity of the cyclic structure [15, 16]. The constrained backbone of cyclic peptides can also hinder protease binding, enhancing stability against proteolytic degradation [17]. Furthermore, cyclization can improve membrane permeability by facilitating intramolecular hydrogen bonding (IMHB). Crossing an apolar membrane requires desolvation of polar atoms, and IMHB can mitigate this energy barrier by reducing the number of solvation sites and burying polar hydrogen donors, thereby enhancing molecular lipophilicity [18, 19, 20]. Cyclosporine A, an immunosuppressant [21], is a classic example of a bRo5 macrocycle with remarkably high oral bioavailability (∼40%) [22] despite its substantial molecular weight (1203 Da). Structural analyses have elucidated its “chameleonic” behavior—the ability to adapt its conformation in polar vs. apolar environments—which effectively conceals polar hydrogen atoms through an IMHB network during membrane transit [23, 24, 25, 26].

Nature has provided a bountiful source of cyclic peptides that can be harnessed for therapeutic purposes, including antitumor agents, antibiotics, and antifungals [14]. Oxytocin and vasopressin are among the earliest cyclic peptides used clinically. In the past three decades, over 40 natural and synthetic cyclic peptides have been approved for medical use [9, 27]. However, only cyclosporine A and analogs such as romidepsin and voclosporin act on intracellular protein targets [14], highlighting the challenges in developing membrane‐permeable cyclic peptides. Recently, Merck has developed the oral macrocycle enlicitide decanoate (MK‐0616) [28, 29], which inhibits the interaction between the protein PCSK9 and the low‐density lipoprotein receptor and is in clinical development for the treatment of hypercholesterolemia [30].

Medium‐sized macrocycles with MW < 1000 Da, such as cyclic heptapeptides, are promising drug modalities for intracellular targets because they are larger than traditional small molecules yet small enough to pass through cellular membranes [9, 31]. Numerous bioactive cyclic heptapeptides have been identified in nature [32], including cordyheptapeptides [33], scytalidamides [34], unguisins [35, 36], and virotoxins [37, 38]. Notably, mortiamides are among the simplest cyclic heptapeptides, containing only aliphatic and aromatic amino acids, an unusually high proportion of α‐D‐amino acids, and no tertiary amide bonds (i.e., lacking proline or *N*‐methyl α‐amino acids). Four mortiamide derivatives (A–D; see Figure S1 for structures) were discovered from a *Mortierella* sp. fungus in a marine sediment from Northern Canada [39, 40] and have demonstrated antimalarial activity against *P*. *falciparum* [41].

Inspired by the simplicity of the mortiamide scaffold, we set out to investigate the passive permeability profiles of cyclic heptapeptide chemical space and associated bioactivities. Our studies revealed two stereochemical groups with high passive permeability; one of these was identified as a mortiamide‐like motif, while the other was an epimer. We prepared a library of approximately 200,000 mortiamide scaffold molecules using a one‐bead‐one‐compound (OBOC) synthesis strategy. Preliminary screening uncovered numerous hits, some with sub‐micromolar cytotoxicities and significant cell line selectivity. However, in general, this scaffold exhibited extremely low aqueous solubility and a pronounced propensity for fibril formation in aqueous media. Our findings underscore that not all natural products are suitable as scaffolds for targeting protein interactions and highlight the necessity of initial physicochemical evaluations prior to launching ligand discovery campaigns, particularly in a cell‐based screening context.

## 2 Results and Discussion

### 2.1 Cyclic Heptapeptides Have Highly Diverse Shake Flask Partitioning Across Stereoisomers

To investigate the passive permeability of cyclic heptapeptide chemical space, we designed two libraries encompassing all possible stereoisomers and spanning a broad lipophilicity range at the side chain level (Scheme S1). We chose to measure lipophilicity because this parameter correlates strongly with transcellular drug permeation [42, 43, 44]. Cyclic heptapeptide scaffolds containing seven chiral α‐carbon centers have 128 theoretical stereoisomers; however, without any *N*‐alkyl amino acids or proline, to a first approximation (barring specific side chain‐backbone interactions), multiple stereoisomers exhibit identical backbone geometries due to circular permutation. Additionally, enantiomeric pairs adopt identical conformations in an achiral environment. By employing a previously published algorithm [45], we reduced the theoretical number of 128 stereoisomers into 10 groups, which we term **stereoindices** (see Scheme S3 for an example). Libraries were synthesized by split‐and‐pool solid‐phase synthesis, incorporating deuterated amino acids to facilitate sequencing by mass spectrometry. Linear peptides were cyclized in solution to yield a total of 1600 cyclic heptapeptides across 16 sublibraries (Scheme S2). Approximately half of the cyclic heptapeptides in these libraries could be directly sequenced based on their unique masses. The remaining half were deconvoluted via tandem mass spectrometry with the CycLS algorithm [46].

**FIGURE 1:**
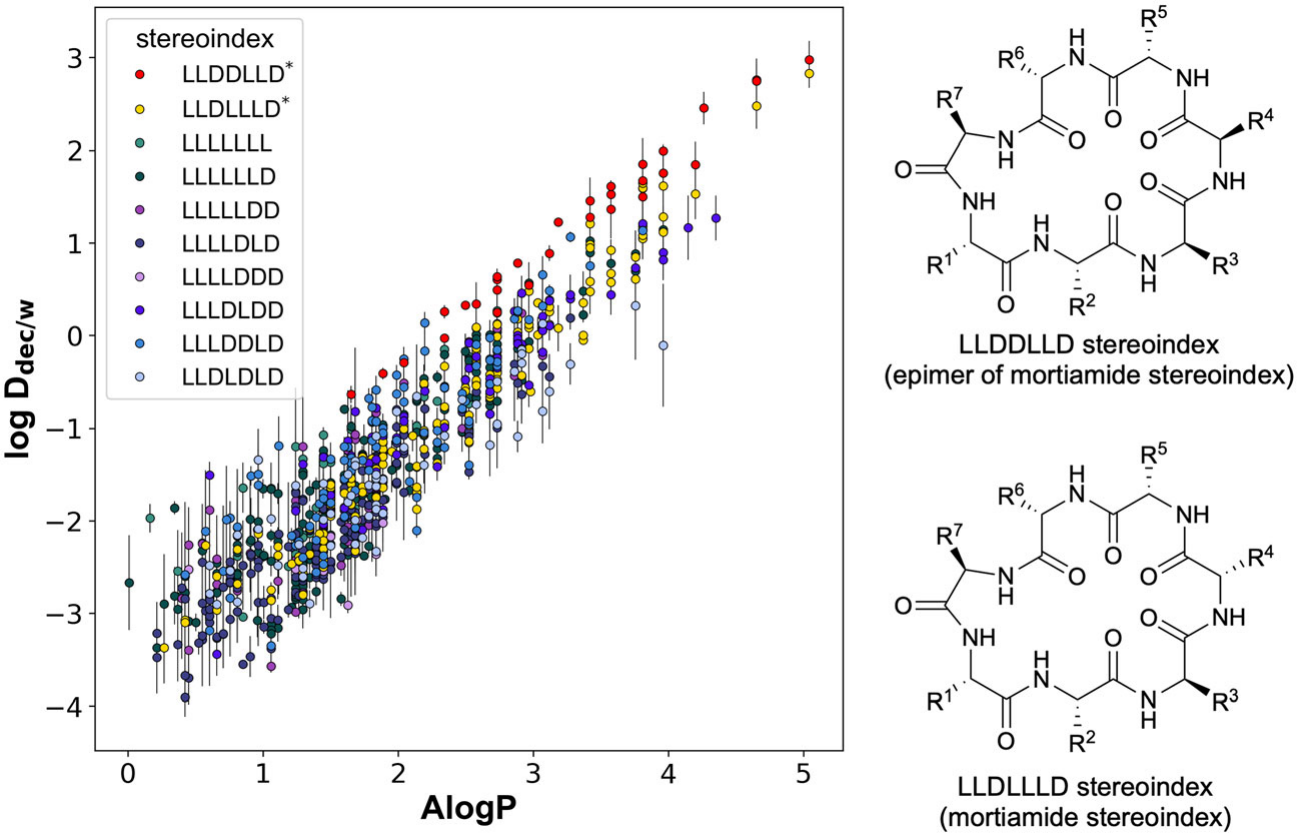
Experimental log *D*_dec/w_ vs. AlogP for cyclic heptapeptides. Only compounds that partitioned into both layers (896 compounds) are plotted. Compounds with disproportionately high distribution into the organic layer (log *D*_dec/w_) relative to others of similar AlogP are likely to have higher transcellular membrane permeability. The top two stereoindices that partition most into the organic phase are LLDDLLD (red) and LLDLLLD (yellow). The log *D*_dec/w_ values are the means  ±  SD of two independent experiments (*n* = 2).

The calculated atomistic 2D octanol/water partition coefficients (AlogP) of the entire set ranged from −0.94 to 5.04. Partition coefficients of these cyclic heptapeptides were quantified in a 1,9‐decadiene/PBS two‐phase system (log *D*_dec/w_), as this partitioning showed a strong correlation with MDCK cell permeability assay results [43]. Shake‐flask partitioning experiments confirmed that 896 cyclic heptapeptides distributed into both the organic and aqueous layers, whereas 381 molecules partitioned exclusively into the aqueous layer (i.e., partitioning into the organic layer was below the limit of detection) (Figure 1). The remaining 323 molecules were not detected in the MS analysis due to incomplete cyclization of certain configurations or side‐chain compositions, as well as peak overlapping that prevented the CycLS algorithm from accurately deducing sequences. A positive correlation between experimental log *D*_dec/w_ and AlogP indicated that more lipophilic molecules partition preferentially into the organic layer. The time‐sensitive nature of these high‐throughput experiments precluded obtaining three replicates for each sample; however, the strong linear correlation between AlogP and log *D*_dec/w_ within each scaffold provides independent validation of the consistency of measurements across the library.

Within the same AlogP range, partitioning among different stereoindices varied by over 100‐fold, implying that backbone conformation strongly influences the sequestration of polar functionality (particularly hydrogen‐bond donors)—a major determinant for improving membrane permeability of macrocycles. Among the 10 stereoindices, LLDDLLD and LLDLLLD exhibited the greatest distribution into the hydrocarbon layer compared to other isomers (highlighted as red and yellow points, respectively). Interestingly, LLDLLLD corresponds to the stereoindex of the mortiamide scaffold (synthesized in this library as its enantiomer). The experimental lipophilicity of compounds sharing this stereoindex varied by up to ten‐fold among different analogs of similar calculated AlogP, indicating that side‐chain composition also plays a role in excluding hydrogen donors. Inverting one stereocenter from the mortiamide stereoindex (yielding the LLDDLLD stereoindex) produced the highest and most consistent partitioning into the organic layer, largely independent of side‐chain composition. The uniform partitioning across different lipophilicity values suggests that the LLDDLLD stereochemistry adopts conformations that effectively shield its hydrogen donors regardless of side‐chain variation.

### 2.2 OBOC Library Synthesis and Screening

**FIGURE 2:**
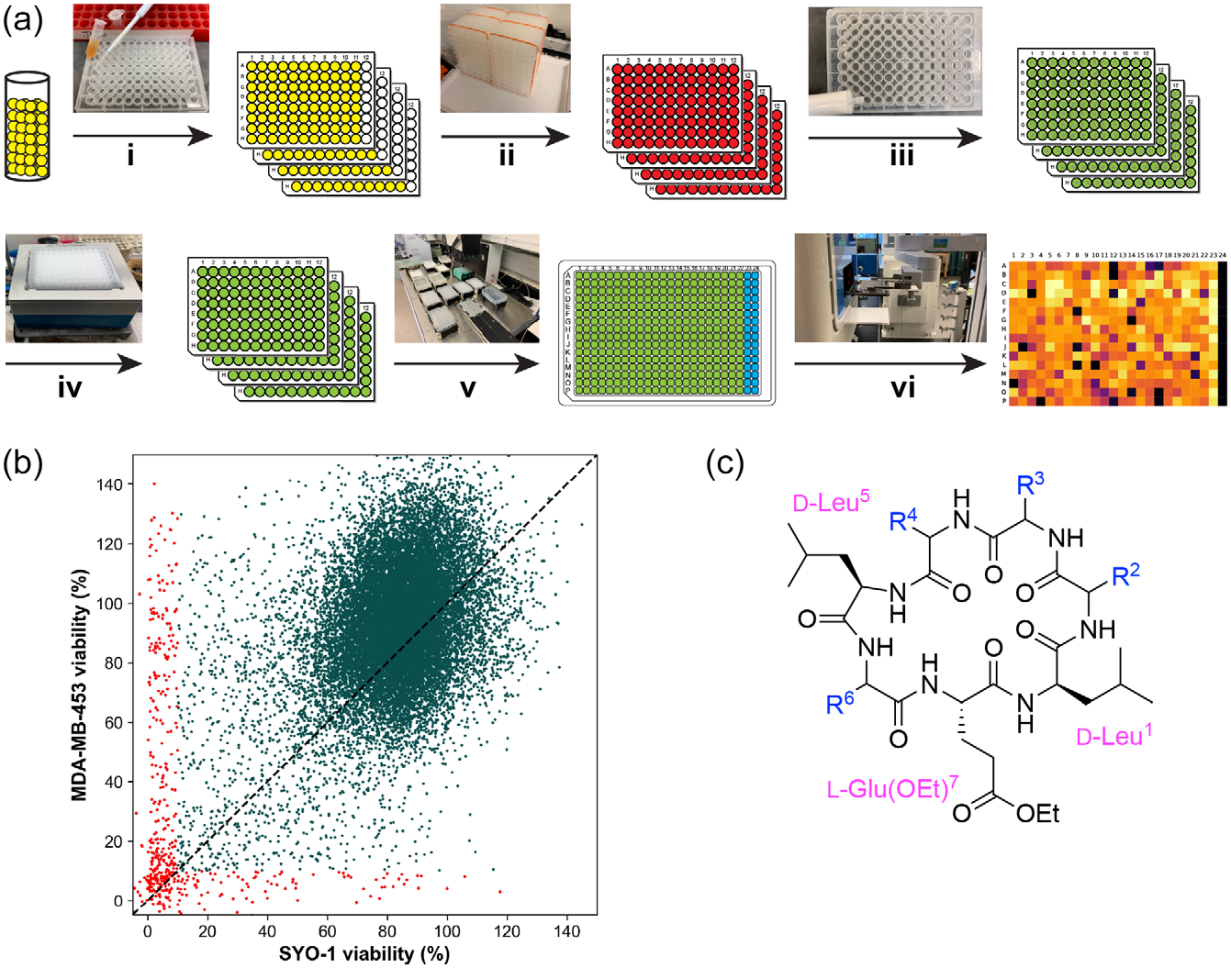
(a) Workflow of OBOC library preparation and screening. (i) Cyclic heptapeptide beads (∼5–10 beads per well) were distributed into 96‐well microplates. (ii) Resin beads were treated with EtOH·HCl, and the plates were shaken overnight. (iii) Acidic solutions were neutralized with Na_2_CO_3_. (iv) Each well received ∼100 μL CHCl_3_, then solutions were filtered and dried. (v) Crude products were resuspended in 10 μL DMSO and reformatted into 384‐well plates. (vi) Stock solutions were screened against SYO‐1 and MDA‐MB‐453 cell lines (CellTiter‐Glo assay). (b) Percent viability of SYO‐1 versus MDA‐MB‐453 for library mixtures. Mixtures reducing cell viability below 10% in either or both cell lines are red. (c) Core cyclic heptapeptide structure used, with side‐chain variable positions R^2^–R^4^ and R^6^ (structures of side chains in Scheme S4).

Results from the partitioning experiments encouraged us to explore the bioactivity of high‐permeability cyclic heptapeptides that could potentially traverse cell membranes and perturb intracellular targets. We selected the mortiamide stereochemical index, LLDLLLD, as a core backbone to prepare a cyclic heptapeptide library using a OBOC strategy (Figure 2c). By using 16 amino acids at four variable positions and an allyl ester as an orthogonal protecting group, linear peptides were cyclized on‐resin, resulting in 65,536 unique sequences. We designed our library to contain at least three copies per unique sequence, ultimately yielding ∼200,000 cyclic heptapeptides (Scheme S4). This library was prepared in microtiter plates by distributing ∼5–10 beads per well and cleaving the macrocycles from the beads while simultaneously esterifying the glutamate side‐chain carboxylate with EtOH·HCl (Figure 2a) [47]. The acidic solutions were then neutralized with Na_2_CO_3_ pellets, solids were filtered out, and the crude products were resuspended in DMSO. Stock solutions were formatted into 384‐well plates for subsequent screening. The calculated AlogP values of this library ranged from −2 to 12 (Figure S2). Purity assessment by LC‐MS indicated that no truncated linear peptides or uncyclized intermediates were present in this library (Figure S3).

We assessed bioactivity in this library by screening against two cancer cell lines—synovial sarcoma (SYO‐1) and breast cancer (MDA‐MB‐453)—and measuring cell viability with a CellTiter‐Glo assay. The initial high‐throughput screen yielded a ∼1%–2% hit rate (defined as mixtures reducing cell viability to 10% or lower in one or both cell lines; Figure 2b). Following this, 271 active mixtures were rescreened at high (1×) and low (0.2×) concentrations in triplicate, confirming 83 active mixtures, which were then subjected to LC‐MS/MS analysis. Using the CycLS program to match MSMS fragments to a virtual fragment library, a total of 556 cyclic heptapeptides were identified and sequenced from these active mixtures.

To pinpoint the active compounds within the mixtures, we leveraged the library's redundancy: statistically, each cyclic peptide was present in at least three different mixtures. Identical peptides recurring across multiple active mixtures were presumed to be hits (Figure S4a). By matching their LC retention times and MS/MS fragmentation fingerprints, we confirmed seven cyclic heptapeptides that fulfilled these criteria (Figure S4c–k). However, many mixtures were ‘contaminated’ with highly lipophilic cyclic heptapeptides that may have interfered with the cell viability assays. Therefore, we also selected additional mixtures containing less lipophilic members (AlogP < 5.3) for further evaluation (Figure S4b), ultimately yielding 34 individual compounds from five active mixtures.

### 2.3 Resynthesis and Hit Validation

Seven cyclic heptapeptides present in multiple active mixtures were resynthesized by standard solid‐phase peptide synthesis (Scheme S5), and their potencies were re‐evaluated by the CellTiter‐Glo assay (**morti01**–**morti07**; Table 1). Notably, five compounds inhibited cell proliferation in both SYO‐1 and MDA‐MB‐453 cell lines at low micromolar concentrations, with **morti03** and **morti07** exhibiting submicromolar potencies. We also synthesized several analogs with alternative side‐chain compositions based on an active hit (**morti01**) and two inactive library members (**morti02** and **morti05**) to probe structure–activity relationships. Incorporating two adjacent 3,4‐difluorophenyl side chains retained similar potency to **morti01** (analog **morti09**), but activity was completely lost when an alanine was inserted between them (**morti08**). Swapping an Ala for a cyclohexylalanine (Cha) or a 3,4‐difluorophenylalanine (Phe(3,4‐diF)) in the inactive **morti02** produced macrocycles with strong and moderate cytotoxicity, respectively (**morti13** and **morti14**). Interestingly, **morti07** was the most potent among five constitutional isomers (**morti05**, **morti10**, **morti11**, **morti12**) and showed ∼8‐fold selectivity toward SYO‐1 cells. Of these isomers, only **morti07** featured a bulky aromatic biphenylalanine (Bip) at position 6; the others had a small aliphatic 4‐aminobutyric acid (Abu) at that position, correlating with reduced potency and complete loss of selectivity.

**TABLE 1:** Potency and PAMPA property of selected cyclic heptapeptides.

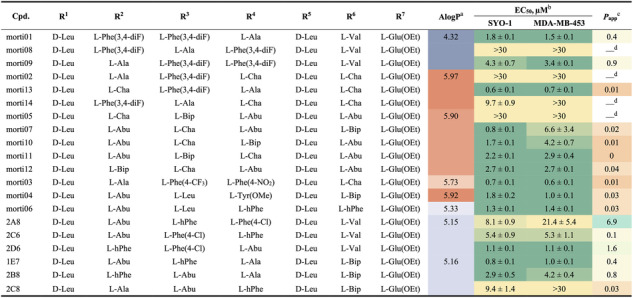											

a Atomistic calculated octanol/water partition coefficient (AlogP), calculated using Discovery Studio software. The higher value indicates the higher lipophilicity.

b EC_50_ represents the half‐maximal effective concentrations to induce cell death determined by CellTiter‐Glo viability assays. The data are expressed as mean values  ±  s.e.m. (*n* = 4)*.*

c Apparent permeability rate from the parallel artificial membrane permeability assay (PAMPA), in units of 10^−6^ cm/s.

d Em dash indicates not applicable.

A focused set of 34 cyclic heptapeptides (the hits with relatively lower lipophilicities, i.e., AlogP  <  5.3) was synthesized, along with two additional variants suggested by the CycLS program, for a total of 102 compounds. We utilized SynPhase Lanterns (solid supports with RFID tags) [48] to facilitate parallel synthesis and sequence deconvolution of this set (Scheme S6). Screening of this focused library yielded 27 active compounds that reduced cell viability in either SYO‐1 or MDA‐MB‐453 cells (see Tables 1 and S2). A few candidates (e.g., **1E7**, **2B3**, **2B5**, **2E5**) exhibited sub‐micromolar potencies comparable to **morti03** and **morti07**, while the majority showed only moderate activity. We also observed differing levels of bioactivity among some isomeric compounds. For example, the **2A8**, **2C6**, and **2D6** isomers showed up to a 20‐fold range of EC_50_ values in the cell viability assay, although none demonstrated clear selectivity between the two cell lines.

Results from hit validation revealed only loose correlations in side‐chain preferences at each variable position. Small aliphatic side chains predominantly occupied the R^2^ position (and to a lesser extent R^4^), whereas aromatic or β‐branched side chains were frequently found at R^3^ and R^6^ among the active compounds. Moreover, the positions of side chains on the cyclic backbone impacted biological activity; as seen with the isomer pairs mentioned above, simply rearranging side‐chain positions markedly altered potency. These observations highlight a complex structure–activity relationship (SAR) for this cyclic heptapeptide scaffold with respect to cytotoxicity.

### 2.4 Bioactivity Links to Physicochemical Properties

**FIGURE 3:**
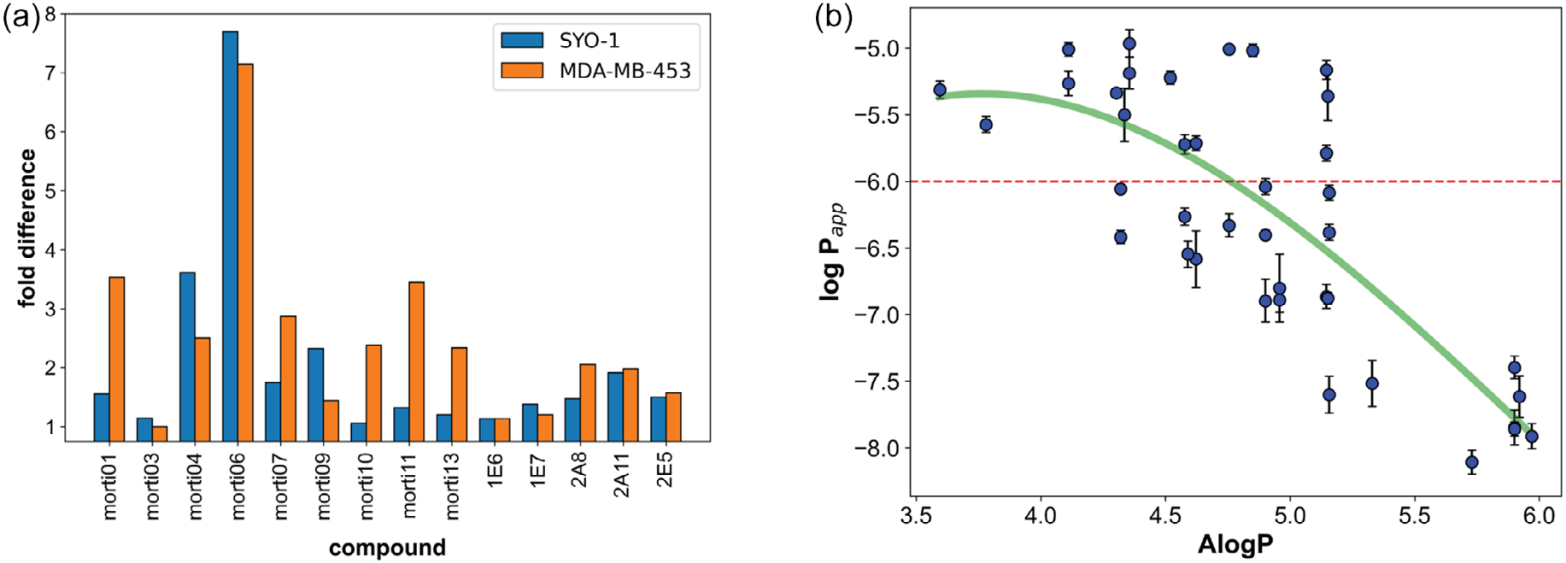
(a) Ratio of EC_50_ values between each potent inhibitor and its enantiomer in CellTiter‐Glo assays. All pairs showed nearly identical potency in both SYO‐1 and MDA‐MB‐453 cells. The largest disparity was for **morti06**, whose enantiomer was less potent; however, **morti06** also exhibited an unusually steep Hill slope (see Table S4). (b) PAMPA permeability (log *P*_app_) of synthetic cyclic heptapeptides as a function of AlogP. The red dashed line indicates the minimum acceptable oral permeability cutoff (*P*_app_ = 1 × 10^−6 ^ cm/s, i.e., log *P*_app_ = −6.0).

The high potency and selectivity of **morti07** prompted us to conduct more in‐depth SAR studies. However, we were unable to identify any analog that surpassed **morti07** in bioactivity or selectivity, nor any clear pattern linking side‐chain identity at a given position to biological activity (see Table S3). Notably, many active cyclic peptides exhibited steep Hill slopes in cell‐based assays. Because a deviation from a Hill slope of −1.0 can suggest assay interference, such as compound aggregation [49, 50, 51], we synthesized 14 enantiomers of selected active hits to further investigate the nature of the observed bioactivity. Generally, enantiomeric compounds exhibit distinct bioactivities due to specific interactions with chiral biological targets like enzymes or receptors. However, we found that our active peptides and their enantiomeric counterparts showed almost identical potencies in both cell lines (Figure 3a and Table S4). In most cases, the EC_50_ ratios (active/enantiomer) were ∼1, with the largest difference being less than 4‐fold. The one exception was **morti06**, whose enantiomer was modestly less potent; however, the steep dose–response curve observed at high concentrations raised concerns about the reliability of that activity (Table S4). These results—compounded by the steep Hill slopes—suggest that the bioactivities of these cyclic heptapeptides stem from physicochemical characteristics rather than target specificity.

Next, we evaluated the passive membrane permeability of the active macrocycles using a parallel artificial membrane permeability assay (PAMPA). We observed a downward parabolic relationship between log *P*_app_ and AlogP: permeability peaked at ∼10 × 10^−6 ^ cm/s (log *P*_app _ ≈  −5.0) around an AlogP of 4.4 (Figure 3b and Table S2). Beyond this optimal lipophilicity, many cyclic heptapeptides showed dramatically lower permeability, suggesting that these compounds had fallen off a ‘solubility cliff’ where limited aqueous solubility constrains membrane permeation [43]. Intriguingly, the sequence of side chains was pivotal in influencing permeability. For instance, in the isomeric series **2A8**, **2D6,** and **2C6**, permeabilities differ by up to ∼50‐fold from the lowest observed rate (**2C6**: 0.1  × 10^−6 ^ cm/s), depending on the positions of the small (Abu) and large (Phe(4‐Cl), hPhe) side chains relative to the scaffold backbone (Table 1). Similar effects were observed in other series: for example, isomers **1E7**, **2B8**, and **2C8** showed up to 30‐fold increases in permeability relative to the lowest rate (**2C8**: 0.03  ×  10^−6 ^ cm/s), and two other isomers (**1D3** and **1F3**) differed by ∼20‐fold from the lowest (**1F3**: 0.5 ×  10^−6 ^ cm/s) simply due to side‐chain rearrangements (Table S2). Additionally, we observed >100‐fold differences in *P*_app_ among cyclic heptapeptides of comparable lipophilicity (AlogP ∼5.15; Figure 3b). These findings underscore the significant impact of side‐chain distribution on passive permeability, potentially because certain side‐chain patterns restrict access to low‐polar‐surface‐area conformations that favor membrane transit.

Thermodynamic aqueous solubility was also assessed for select active compounds in PBS buffer. Because this measurement requires substantial material, a subset of the most active macrocycles was tested (Table 2). Overall, these compounds displayed extremely low solubility (<1 μM). The most potent peptides, **morti07** and **2B3**, were insoluble under these conditions, while even the more hydrophilic analogs (e.g., **1E6** and **7‐A** ^**6**^) had solubilities below their effective concentrations in the cell viability assays (Table 2). Considering the low solubility and moderate permeability of many active compounds, as well as the equipotency of enantiomeric pairs, we speculated that the apparent bioactivities of these cyclic peptides were primarily driven by their physicochemical properties (aggregation, precipitation, membrane adherence, etc.) rather than specific target engagement.

**TABLE 2:** Comparison of solubility between mortiamide stereoindex and epimer.

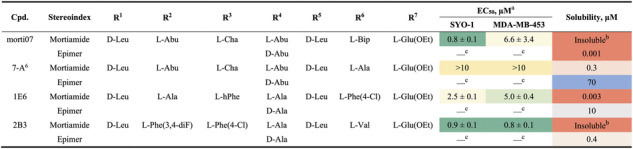											

a EC_50_ represents the half‐maximal effective concentrations to induce cell death determined by CellTiter‐Glo viability assays. The data are expressed as mean values  ±  s.e.m. (*n* = 4)*.*

b “Insoluble” indicates not detectable in MS.

c Em dash indicates not applicable.

### 2.5 Low Solubility Is an Intrinsic Property of Mortiamide Scaffold

Given the overall low solubility of the active inhibitors, we next examined whether this characteristic is related to the mortiamide backbone geometry itself. We prepared the natural product mortiamides A–D (which were not included in our synthetic library). The backbones of these natural products are enantiomeric to those in our library (natural mortiamides have configuration LDDDLDD, versus the LLDLLLD scaffold in the library), and they also lack the ethyl ester on the glutamic acid side chain (bearing a hydrophobic side chain instead; Table 3). Unfortunately, we were unable to obtain mortiamide C due to synthetic challenges, specifically an inability to cyclize this compound. Like the synthetic cyclic heptapeptides, the natural products mortiamides A, B, and D all exhibited very low aqueous solubility. Interestingly, mortiamide B was significantly more soluble than mortiamides A and D despite its substantially higher calculated lipophilicity. Further studies are needed to understand how a single amino acid change (Leu in mortiamide A vs. Phe in mortiamide B) leads to such a dramatic increase in solubility. While side‐chain composition can influence solubility (as evidenced by the difference between mortiamides A and B), the low overall solubility of the mortiamide scaffold is more likely governed by the impact of its conformational preferences on its tendency to aggregate in aqueous solution.

**TABLE 3:** Experimental data of natural product mortiamides and their derivatives.

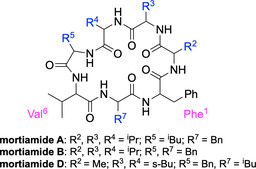						
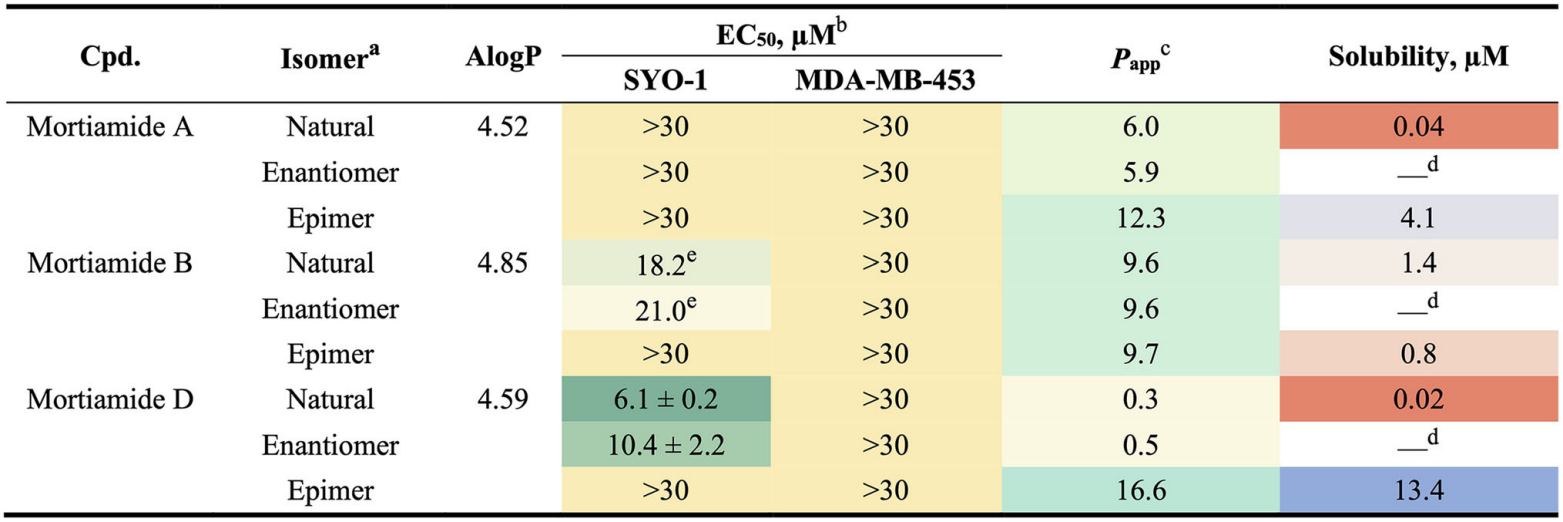						

a “Natural” refers to the configuration of the natural product, LDDDLDD; “enantiomer” refers to the mirror image of the natural product, DLLLDLL; “epimer” refers to the LDDLLDD configuration.

b EC_50_ represents the half‐maximal effective concentrations to induce cell death determined by CellTiter‐Glo viability assays. The data are expressed as mean values  ±  s.e.m. (*n* = 7)*.*

c Apparent permeability rate from the parallel artificial membrane permeability assay (PAMPA), in units of 10^−6 ^ cm/s.

d Em dash indicates not applicable.

e Standard error of mean cannot be determined due to high Hill slopes.

To confirm the effect of backbone geometry on solubility, we synthesized *epimers* of the mortiamides by altering their stereochemical configuration from LDDDLDD to LDDLLDD (corresponding to the LLDLLLD vs. LLDDLLD stereoindices, respectively). The epimeric LLDDLLD stereochemistry demonstrated the highest distribution to the organic layer in our partitioning experiments (Figure 1). As shown in Table 3, the epimers of mortiamides A and D exhibited over 100‐fold greater aqueous solubility than their natural isomers, while mortiamide B's epimer was slightly less soluble than the natural form. Similar solubility trends were observed in our synthetic cyclic heptapeptides (Table 2): despite their high overall lipophilicities, the epimers of **morti07** and **2B3** were sparingly soluble (in PBS), representing an improvement over the completely insoluble parent scaffolds. Remarkably, the more hydrophilic compounds **7‐A** ^**6**^ and **1E6** saw dramatic solubility increases of over 200‐fold and 3000‐fold, respectively, upon epimerization. Collectively, these findings underscore that both backbone geometry and side‐chain composition are crucial factors underlying the very low aqueous solubility of the mortiamide stereoindex.

Additionally, we evaluated the bioactivity of the natural mortiamides and their derivatives (Table 3). Mortiamide D showed modest inhibition of SYO‐1 cell viability at concentrations below 10 μM. In contrast, mortiamide B exhibited only weak cytotoxicity, and mortiamide A was inactive. None of the natural mortiamides showed activity against the MDA‐MB‐453 cell line. Notably, as observed with our synthetic cyclic peptides, the enantiomers of mortiamides A, B, and D had nearly identical potencies to their natural counterparts, and all displayed high Hill slope coefficients. This suggests that the cytotoxicity of these natural products, while relatively weak, is likewise influenced by their physicochemical properties (aggregation, etc.). Consistent with this observation, the much more soluble mortiamide epimers showed no cytotoxic activity in either cell line.

### 2.6 Active Cyclic Heptapeptides Form Amyloid‐Like Fibril Aggregates

**FIGURE 4:**
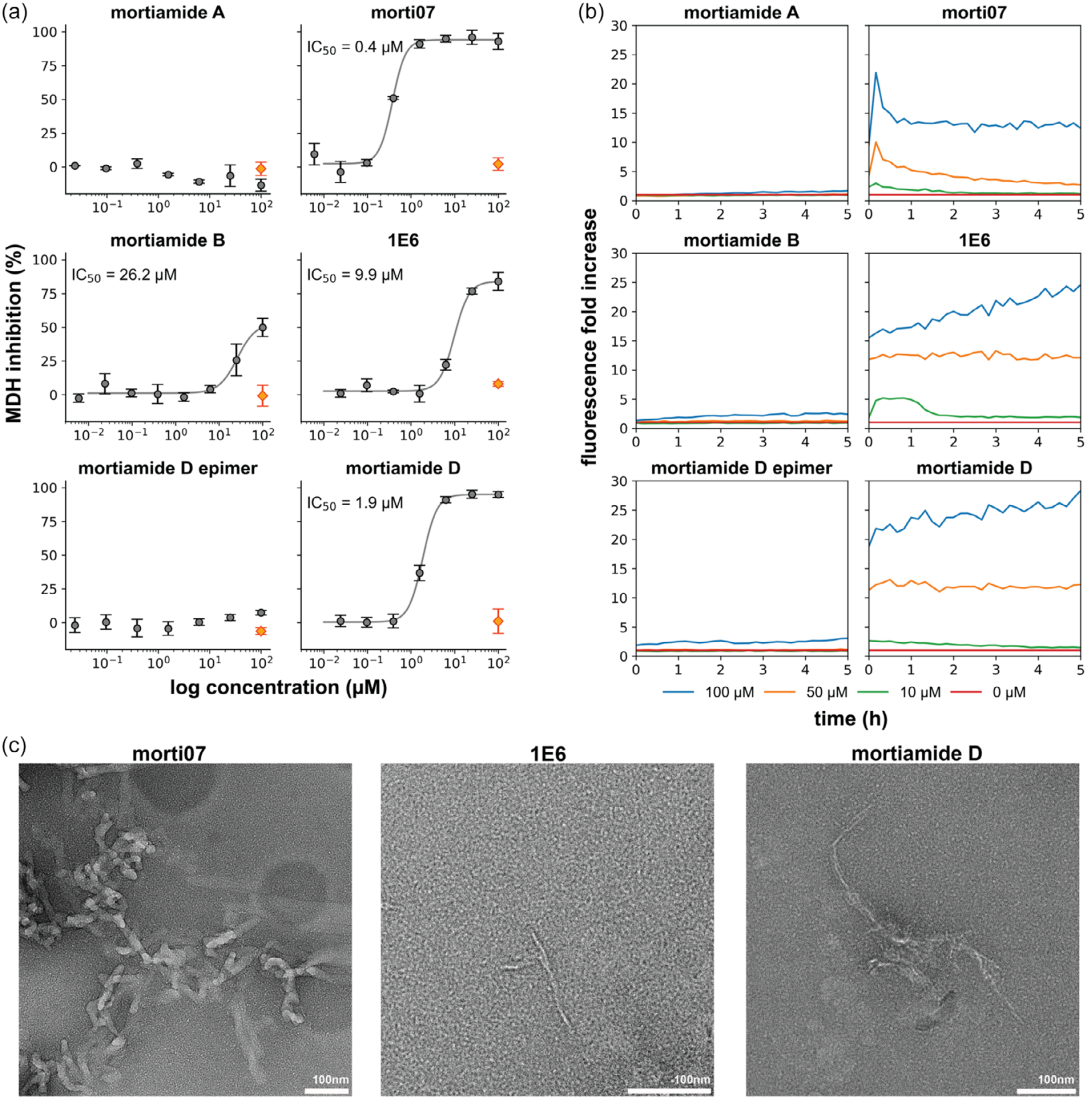
(a) Detergent‐dependent inhibition in the MDH counter‐screen assay. Concentration–response curves for MDH inhibition (gray circles) are completely shifted upward (orange diamonds) upon pre‐incubation with 0.01% Triton X‐100. All measurements were performed in triplicate. (b) ThT fluorescence increases after incubation with cyclic heptapeptides at various concentrations. The magnitude of fluorescence change corresponds with the aggregation propensities observed in the MDH assay. (c) Representative TEM micrographs of aggregates formed by active cyclic heptapeptides. Images are collected after incubating the sample quiescently for 24 h at 37°C (*100 μM*, *PBS buffer* (*pH 7.4*))*.* Scale bars are as shown.

Colloidal aggregation is a major issue that can lead to false positives in drug discovery screens. While one indicator of compound aggregation is an unusually steep dose–response (Hill) slope in high‐throughput assays, this criterion is not universally applicable to all aggregators [49, 50]. Thus, additional experiments are necessary to substantiate whether lead candidates act via aggregation. To classify a molecule as an aggregator, a common test is to determine whether it inhibits an enzyme, such as malate dehydrogenase (MDH), whose activity is sensitive to the presence of lipophilic aggregates, and whether this inhibition is attenuated by disrupting colloid formation with a detergent [52, 53]. Compounds that inhibit the activity of MDH in a detergent‐dependent manner indicate the presence of colloidal aggregates. Of seven cyclic heptapeptides tested, five inhibited MDH activity at 100 μM in PBS buffer. This inhibition was rescued by addition of 0.01% Triton X‐100 (a non‐ionic detergent), indicating an aggregation‐based mechanism (Figure 4a). We next performed dose–response analyses to determine IC_50_ values for MDH inhibition by these compounds; the IC_50_ results aligned closely with their EC_50_ values from the cell viability assay. For example, **morti07**, which showed strong cell potency against SYO‐1, inhibited MDH at submicromolar concentrations, whereas the weakly potent mortiamide B disrupted enzyme activity (and cell viability) at much higher concentrations (>10 μM). Mortiamide D and **1E6** were active in both MDH enzyme and cell assays in the 1–10 μM range, whereas inactive mortiamide A and the mortiamide D epimer did not cause MDH inhibition even at 100 μM. Although **7‐A** ^**6**^ was biologically inactive in cell assays, its MDH IC_50_ above 10 μM suggests that its true EC_50_ was higher than the top concentration tested in the cell viability experiment (Figure S5).

To further investigate the aggregation of these compounds, we measured changes in the fluorescent dye thioflavin T (ThT), whose fluorescence increases upon association with protein aggregates such as amyloid fibrils [54]. While ThT is generally used to detect protein fibrils, we reasoned that cyclic peptide aggregates might similarly restrict the rotational freedom of ThT molecules, leading to fluorescence enhancement. Indeed, we observed concentration‐dependent ThT fluorescence increases for the peptides, and these changes correlated with the IC_50_ values from the MDH assay. The non‐aggregating or weakly aggregating compounds caused only 2–4‐fold fluorescence increases at 100 μM and no significant change at lower concentrations (mortiamide A, mortiamide B, and mortiamide D epimer; Figure 4b). In contrast, the known aggregators (mortiamide D, **morti07**, and **1E6**) produced significant fluorescence enhancement even at the lowest tested concentration (10 μM). Unlike amyloidogenic proteins, which typically exhibit a lag phase before fibril formation, our cyclic peptides enhanced ThT fluorescence immediately from the outset of measurements. We hypothesize that these cyclic heptapeptides nucleate into aggregates much faster than amyloid‐forming peptides, allowing ThT to bind rapidly to a large initial surface area of nuclei. This could explain the sharp increase in fluorescence at early time points. Over time, as the nuclei convert to larger aggregates, the total accessible ThT‐binding surface may decrease, contributing to a drop in fluorescence intensity. These effects were especially pronounced for **morti07**, and at the lowest concentrations of mortiamide D and **1E6**.

Considering the fluorescence enhancement observed in ThT binding assay, we further investigated these peptide aggregates using electron microscopy (Figures 4c and S15). TEM images of **morti07** confirmed the formation of rod‐like fibrils of approximately 24.5 Å width. We also observed globular (amorphous) aggregates interspersed among the fibrils. Mortiamide D and **1E6** also formed fibrils that are relatively thin compared to **morti07** fibrils, whereas no fibril‐like aggregations were observed for the other cyclic heptapeptides that were inactive (Figure S14). This is consistent with the ThT‐binding results. Additionally, we conducted structural analysis in cryogenic conditions, or cryo‐electron microscopy (cryoEM), for more detailed analysis. CryoEM imaging, followed by processing using CryoSPARC v4.5, revealed that the **morti07** fibril population contains only a single class with a straight‐looking morphology (Figure S16). The poorly twisted nature of the fibril limited the ability to obtain sub‐nanometric resolution on the helical reconstructed volume. In contrast to these fibrils, cryoEM analysis of mortiamide A confirmed the formation of spherical aggregates of ∼13 nm in diameter (Figure S17). These findings support the conclusion that the fluorescence enhancements observed in the ThT‐binding assay are linked to fibril formation by the more active cyclic heptapeptides.

To gather further evidence of colloid formation, we used DLS to measure particle sizes of two representative aggregating peptides, **morti07** and **1E6**, in PBS at 50 μM. As expected, both compounds greatly increased light scattering intensity, with estimated particle radii of ∼1000 nm (Figure S6). In contrast, the scattering intensity of a mortiamide A solution remained unchanged, consistent with other assays indicating that this molecule does not form colloidal particles in solution. We also noted that the particle size distributions of **morti07** and **1E6** broadened and shifted over time, highlighting the dynamic nature of their aggregation process.

Results from these experiments collectively support the conclusion that the mortiamide scaffold readily aggregates in aqueous solution. Notably, high hydrophobicity alone does not determine aggregation; side‐chain combinations and stereochemical configuration also influence colloid formation. For instance, both **1E6** and **7‐A** ^**6**^ (which are more hydrophilic than the non‐aggregating mortiamide A) still exhibit characteristic enzyme sequestration and detergent‐reversible inhibition of MDH, indicating that hydrophilicity does not guarantee lack of aggregation. Conversely, substituting a more hydrophobic Phe side chain for Leu in mortiamide A yields mortiamide B, which does aggregate at high concentrations. Meanwhile, mortiamide D—whose calculated hydrophobicity is similar to that of mortiamide A—strongly inhibits MDH and enhances ThT fluorescence, likely due to its distinct side‐chain composition relative to mortiamides A and B. Converting a single stereocenter of mortiamide D (to its epimer) abolishes MDH inhibition and ThT fluorescence increases, suggesting that backbone conformation critically influences intermolecular aggregation. Moreover, the similar potencies observed in the MDH assay and the cell viability assay for the active peptides support the idea that their cytotoxic effects are intrinsically linked to their colloidal behavior. Based on our TEM observations, we speculate that fibril formation by the active compounds (e.g., mortiamide D, **morti07**, and **1E6**) underlies their cytotoxic activity against the cancer cell lines. Additionally, our use of the ThT fluorescence assay illustrates the potential of this dye for investigating macrocycle aggregation.

### 2.7 Experimental and Computational Analysis of the Mortiamide Scaffold

**FIGURE 5:**
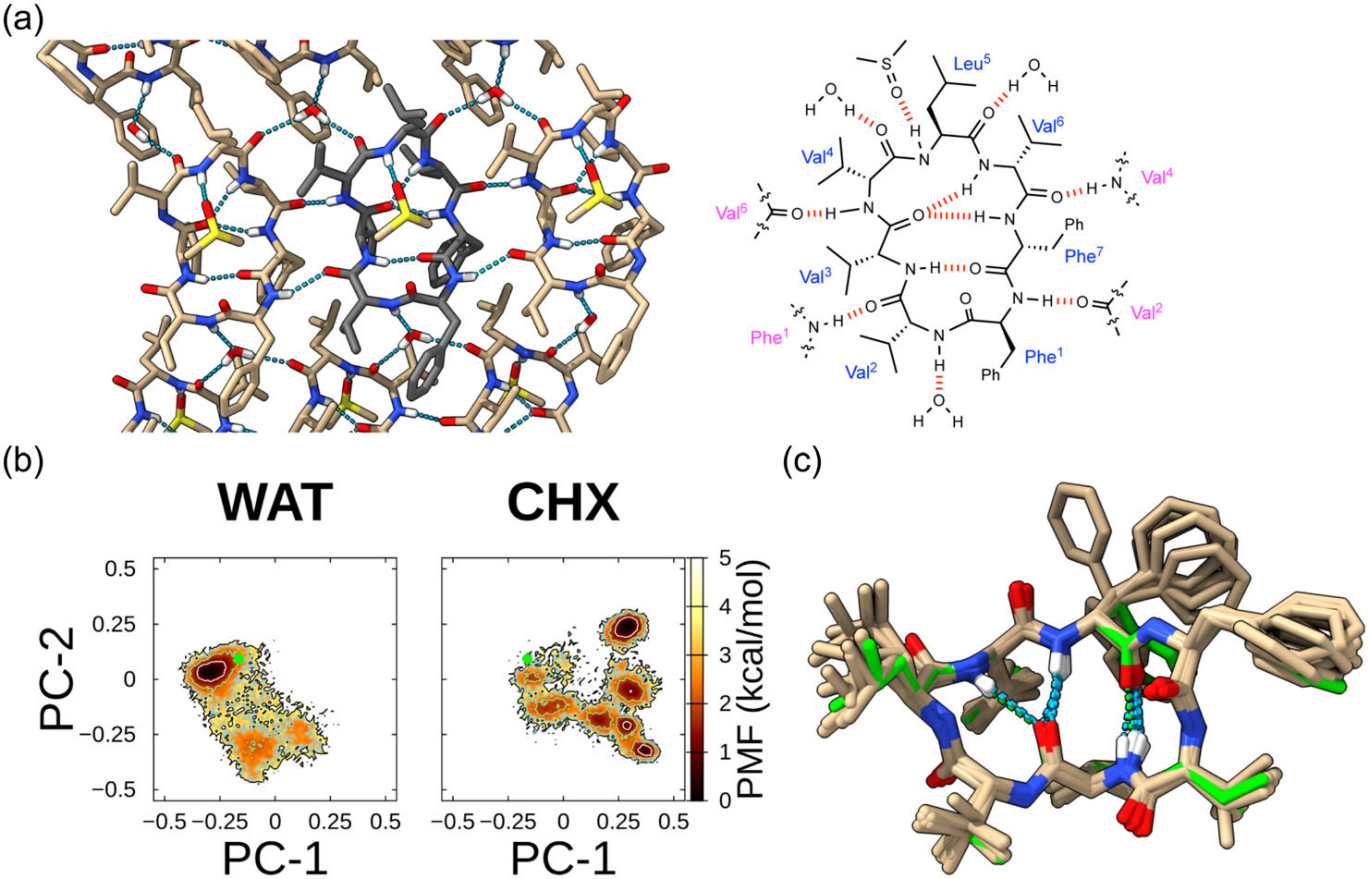
(a) Crystal structure of mortiamide A highlighting the intramolecular and intermolecular hydrogen‐bond network. Three backbone amide protons (Val3, Val6, Phe7) form intramolecular hydrogen bonds, while Phe1 NH and Val4 NH form intermolecular hydrogen bonds with neighboring molecules. Val2 NH and Leu5 NH form hydrogen bonds with water and DMSO molecules, respectively. A single water molecule bridges three cyclic heptapeptide molecules in the lattice. (b) Free‐energy landscape of mortiamide A along principal components 1 and 2 at 300 K, in water (WAT) and cyclohexane (CHX). The X‐ray crystal conformation (green dot) is overlaid. Contour lines for 1.0, 2.0, 3.0, and 4.0 kcal/mol are shown in white, green, sky‐blue, and black, respectively. (c) Overlay of mortiamide A's crystal structure (green) with the top 10 simulated McMD conformers (water, 300 K) having backbone RMSD < 0.5 Å from the crystal.

To better understand the structural basis of the mortiamide scaffold's behavior, we solved the single‐crystal X‐ray structure of mortiamide A. Crystals were obtained by vapor diffusion (DMSO/water) and diffracted to 0.83 Å resolution. The crystal structure revealed several intra‐ and intermolecular hydrogen bonds (Figures 5a and S10). Specifically, the backbone amide protons of Val3 and Phe7 form transannular hydrogen bonds, yielding a type II β‐turn and an antiparallel β‐sheet‐like motif. Additionally, the Val6 NH donates an intramolecular hydrogen bond to the Val3 C=O, forming a type II′ β‐turn. Regarding intermolecular interactions, four hydrogen bonds connect neighboring molecules: two between Phe1 NH and Val2 C=O, and two between Val4 NH and Val6 C=O of adjacent macrocycles. Three polar groups (Val2 NH, Val4 C=O, and Leu5 C=O) each engage in hydrogen bonding with water molecules; notably, one water molecule bridges three macrocycle units in the crystal lattice by linking Val2 NH of one molecule to Val4 C=O of a second and Leu5 C=O of a third. Furthermore, the only remaining free NH (Leu5) interacts with the sulfoxide of a DMSO molecule positioned above the ring. We also obtained a crystal structure of the mortiamide B enantiomer under similar conditions. The hydrogen‐bonding network in the mortiamide B enantiomer was essentially identical to that of mortiamide A, except that the lattice water molecule in B's crystal connects only two macrocycles (between Val2 NH and Val4 C=O; see Figures S7 and S11). This subtle difference in crystal packing may explain the higher solubility of mortiamide B, as mortiamide A's more extensive hydrogen‐bond network could require more energy (and stronger solvation) to disrupt.

We then investigated the conformational ensemble of mortiamide A in different solvents using multicanonical molecular dynamics (McMD) simulations [55]. The MD‐derived free energy landscapes indicated that mortiamide A predominantly adopted one major conformer (>60% population) in water, whereas it sampled multiple conformations in the nonpolar solvent cyclohexane (Figures 5b and S9). This behavior is opposite to the common observation that lipophilic, membrane‐permeable macrocycles such as cyclosporine A are often relatively flexible in aqueous environments and more conformationally restricted in low‐dielectric (apolar) media [55]. Calculating the backbone RMSD between the major conformer in water and the X‐ray crystal structure yielded ca. 0.82 Å. This degree of deviation is considered reasonable given the crystal state, where multiple molecules interact due to crystal packing (plus the influence of DMSO), compared to the structure of a single molecule in a solvent. Franca^39^ reported backbone RMSDs consistently below 2 Å for mortiamides A–D. Furthermore, approximately 5% of the total water‐phase MD conformers matched the backbone of the X‐ray crystal structure within 0.5 Å RMSD (Figure 5c).

To evaluate whether diverse amino acids would alter the backbone conformation of the mortiamide scaffold, we prepared single‐crystal X‐ray diffraction structures of two LLDLLLD cyclic heptapeptides (**morti15** and **morti16**). In these structures, the side chains featured the heteroatom‐containing residues glutamic ethyl ester and thiazolyl alanine (Figures S12−S13). Comparisons between these two crystals and the mortiamide B enantiomer—all of which have matched configurations—revealed that they adopted identical backbone conformations in the crystal lattice (Figure S8). In both cases, the backbone RMSDs were below 0.4 Å, indicating that the scaffold conformation remained largely fixed regardless of side‐chain composition [67].

## 3 Conclusion

In summary, a comprehensive examination of the ten **stereoindices** (stereochemical cluster groups) representing all possible cyclic heptapeptide diastereoisomeric scaffolds (including their enantiomers) revealed two isomers with exceptionally high intrinsic lipophilicity. One corresponds to the enantiomeric configuration of the natural product mortiamides (LLDLLLD), and the other to its epimer (LLDDLLD). Notably, the LLDLLLD stereoindex showed highly variable partitioning behavior depending on side‐chain composition (suggesting that different side chains modulate its ability to hide polar groups), whereas the LLDDLLD stereoindex exhibited consistently high partitioning into hydrophobic phases, implying that its conformations effectively shield polar surface area regardless of side‐chain variation.

We synthesized a library of over 200,000 cyclic heptapeptides based on the LLDLLLD mortiamide scaffold via solid‐phase combinatorial chemistry. Several hits from this library showed low‐ to sub‐micromolar cytotoxicities in synovial sarcoma and breast cancer cells. However, these active compounds uniformly displayed low aqueous solubility and a strong tendency to aggregate in aqueous solution as confirmed by a series of orthogonal experiments, including detergent‐sensitive inhibition of a counter‐screen enzyme (MDH), enhancement of ThT fluorescence upon peptide incubation, visualization of fibril aggregate by TEM, and detection of colloidal particles by light scattering. Furthermore, each active compound's enantiomer was essentially equipotent to the parent, reinforcing that the observed bioactivities are due to nonspecific physicochemical effects rather than stereospecific target engagement.

These false‐positive cytotoxic effects in cell‐based assays run counter to the typical behavior of colloidal aggregators, which more often cause false negatives due to sequestration of active monomer [53]. Unlike the spherical colloids formed by most small‐molecule aggregators, our cyclic peptides form amyloid‐like fibrillar aggregates. To our knowledge, previous reports of synthetic small‐molecule fibrils are limited to certain coumarin‐based compounds [56, 57, 58]. The cytotoxicity of these ‘chemical fibrils’ is reminiscent of the toxic effects exerted by amyloid fibrils on cultured cells and neurons [59]. We speculate that the cytotoxicity of our mortiamide cyclic heptapeptides is similarly linked to their fibrillization. The mechanism proposed by Wells et al. suggests that chemical fibrils enter cells via endocytosis and accumulate in lysosomes. The fibrils cause intracellular vesicles to leak their contents, leading to the activation of caspases and apoptosis [58]. However, further studies are required to elucidate how fibrils formed by LLDLLLD mortiamide peptides trigger cell death.

Natural product mortiamides have been reported as antiparasitic (antiplasmodial) agents, and various structural modifications have been explored to improve their efficacy [60, 61]. Our findings indicate that these natural products possess very low solubility, and their cytotoxic potencies against cancer cells are comparable to those of their enantiomers. Intriguingly, the aqueous solubilities of mortiamides A–D are lower than the IC_50_ values reported for their antimalarial activity [41]. This discrepancy raises the possibility that the bioactivities of mortiamides may arise from their propensity to aggregate, leading to nonspecific toxicity. Recently, Bergeron et al. (Lawrence's group) reported that mortiamide D has poor permeability in both PAMPA and Caco‐2 assays but exhibits high recovery from cellular extracts [61]. This observation suggests that mortiamide D tends to accumulate in or on cell membranes. Together with our reports, this leads us to postulate that mortiamides act as aggregating agents that localize on cellular membranes, potentially compromising membrane integrity and resulting in cell death.

The data presented here suggest that subtle differences in side chains and backbone geometries of macrocyclic peptides can lead to vastly different physicochemical properties and assumed protein‐binding characteristics. Nevertheless, by focusing on backbone and/or side‐chain patterns, libraries can be designed to include a large number of permeable cyclic peptides. This type of strategy may have utility in identifying novel intracellular bioactive compounds, but considerations such as nonspecific aggregation‐based interference must be carefully addressed. We encourage researchers to conduct counter‐screening assays during the early stages of the drug discovery pipeline, especially when pursuing hits from cell‐based assays. Lane et al., previously described experimental protocols—such as adding detergent to fluorescence polarization (FP) assays or employing SPR and ITC as orthogonal assays—to de‐risk interference and identify promiscuous off‐target activity [62]. Furthermore, work by Shoichet et al. demonstrated that adding 0.025% Tween‐80 to cell‐based assays can eliminate aggregators without compromising cell viability [53]. We anticipate our analyses will serve as a complementary framework for triaging compounds with nonspecific behavior, ultimately enhancing the quality of initial hits in drug discovery.

## 4 Experimental Section

### 4.1 Materials and Methods

All reactions were carried out under an inert atmosphere (nitrogen or argon, where stated) with dry solvents under anhydrous conditions. Glassware for anhydrous reactions was dried in an oven at 140°C for a minimum of 6 h prior to use. Dry solvents were obtained by passing the previously degassed solvents through activated alumina columns. Reagents were purchased at a high commercial quality (typically 97% or higher) and used without further purification, unless otherwise stated. Amino acids, COMU, HATU, Pd(PPh_3_)_4,_ and 1,9‐decadiene were purchased from Combi‐Blocks. HOAt was purchased from AAPPTec. Piperidine was purchased from Spectrum Chemical. HFIP was purchased from Oakwood Chemical. ^i^Pr_2_NEt was purchased from Acros Organics. 2‐Chlorotrityl chloride polystyrene resin was purchased from Rapp‐Polymere. Synphase Lanterns were purchased from Mimotopes. General purity assessment of individual cyclic heptapeptides was determined on an Advion AVANT HPLC‐expression CMS system using a C18 Kinetex colum (30  ×  2.1 mm, 2.6 µm 100 Å) at a flow rate of 0.5 mL/min. The mobile phase was composed of 0.1% (v/v) formic acid in Milli‐Q water (solvent A) and 0.1% formic acid in CH_3_CN (solvent B). The gradient elution was ramped from 20 to 100% B over 4 min and held at 100% B for 1 min. The detection was performed using a UV detector at 200 nm and 254 nm or ELSD detector (Agilent), and the column temperature in the oven was 50°C. All key individual synthesized compounds are ≥95% pure by HPLC or NMR analysis.

### 4.2 General Protocol of UPLC‐MS Analysis

UPLC‐MS analyses of cyclic peptides were performed via a Thermo Scientific Ultimate 3000 UPLC system, using a Thermo Hypersil GOLD C18 30 mm  × 2.1 mm (1.9 µm) column (#25002‐032130). The mobile phase was composed of 0.1% (v/v) formic acid in Milli‐Q water (solvent A) and 0.1% formic acid in MeCN (solvent B). Flow rate was set to 1 mL/min and a gradient method was as followed: 0.0–0.5 min, 5% MeCN; 0.5–0.75 min, ramp to 95% MeCN; 0.75–3.0 min, 95% MeCN; 3.0–3.5 min, 5% MeCN. Mass identification and quantification used an inline Thermo Scientific Orbitrap VelosPro (FTMS mode), tuned for maximum ionization of cyclosporin A, background ion locking on octyl phthalate, 200–2000 AMU mass windows, using ±0.02 AMU windows for integration.

### 4.3 Shake Flask Partition Experiment

Cyclic heptapeptides (400 µM, 8 µL) in DMSO were mixed in a 1:1 solution of 1,9‐decadiene and PBS buffer (1.6 mL) and agitated by vortex (30 min) and sonication (30 min). Analytes were centrifuged for 10 min at 16,000×*g* to separate the two layers. The 150 µL aliquots from each layer, in quadruplicate, were transferred to microplates and evaporated overnight with a Genevac centrifugal evaporator. Samples were resuspended in 150 µL of 1:1 MeCN/H_2_O, and the partitioning was determined by UPLC‐MS as described above.

### 4.4 Parallel Artificial Membrane Permeability Assay (PAMPA)

A 96‐well donor plate with 0.45 µm hydrophobic Immobilon‐P membrane supports (Millipore MAIPNTR10) was loaded with 5 µL of 1% w/v lecithin in *n*‐dodecane. Cyclic heptapeptides in PBS solutions containing 5% DMSO (2 µM) were loaded into the donor plate (150 µL), which was then placed on top of the acceptor plate (Millipore MSSACCEPTOR) containing 300 µL of 5% DMSO in PBS buffer. The assays were run in quadruplicate for ∼18 h at 20°C. The donor and acceptor plates were separated, and 50 µL solutions of each well (both donor and acceptor plates) were mixed with 50 µL MeOH in new microplates. The analytes were determined by UPLC‐MS, and the apparent permeabilities (P_app_) were calculated as described previously [63].

### 4.5 Thermodynamic Solubility Assay

Stock solutions of cyclic heptapeptides in MeOH (10 mM, 10 µL) were dispensed to a 96‐well microplate and dried under compressed air with a microplate evaporator (Evaporex, SPT Labtech). Dried cyclic heptapeptides were resuspended in 100 µL PBS, and the microplate was sealed and sonicated for 30 min. Samples were gently shaken at 37°C for 24 h. The mixtures were centrifuged for 1 min and filtered through the MultiScreen aqueous solubility filter plate (MSSLBPC10). Filtrates (50 µL) were diluted with MeOH (50 µL) and quantified by UPLC‐MS. Standard curves were acquired in triplicate from serial dilution of stock solutions in DMSO (100–0.001 µM). Analytes were tested in five replicates and averaged.

### 4.6 Counter‐Screening Enzyme MDH Assays

Enzyme MDH inhibition assays were followed according to published protocols [50] with some modifications. Assays were performed in 250 µL of 50 mM KPi buffer, pH 7.0, with the final DMSO concentration at 1% v/v in triplicate. Compounds were mixed and preincubated with 1.5 µg/mL MDH enzyme (MilliporeSigma, 442610‐10KU) in 96‐well Costar UV‐transparent microplates (Corning, 3635) for 5 min at 25°C. Then, the MDH reactions were initiated by the addition of nicotinamide adenine dinucleotide (NADH; Sigma–Aldrich, N8129) and oxaloacetic acid (OAA; Combi‐Blocks, QB‐3671), both at 200 µM final concentration. The solutions were mixed and immediately recorded the absorbance at 340 nm every 5 s for a total of 120 s with an Envision plate reader (Perkin Elmer). Initial rates were divided by the DMSO control rate to determine the percent enzyme activity. Compounds were initially screened at 100 µM with or without 0.01% v/v Triton X‐100, and rescreened at 100, 25, 6.25, 1.56, 0.39, 0.097, 0.024, and 0.006 µM final concentrations for the concentration–response curve. Curcumin was used as the positive control in this work.

### 4.7 Thioflavin T Assay (ThT)

The Thioflavin T assays were adapted from the protocol used for detecting the formation of amyloid fibrils [54]. Briefly, ThT dye was dissolved in PBS buffer and filtered through a 0.2 µm syringe filter, and the concentration was determined using an extinction coefficient of 36 mM^−1^ cm^−1^ at 412 nm. The ThT solution was prepared at a 50 µM final concentration. In 384‐well plates, compounds in DMSO stock solution were dosed using the Echo 650 liquid handler at 0, 10, 50, and 100 µM final concentrations (400  nL). The assays were initiated by the addition of 40 µL ThT solution, and the ThT fluorescence intensities were measured at room temperature using an Envision plate reader from the top of the microplate with an excitation filter of 450 nm and an emission filter of 490 nm. The measurements were recorded every 10 min for over 20 h.

### 4.8 Dynamic Light Scattering (DLS)

Cyclic heptapeptides (10 mM in DMSO) were diluted to 50 µM with filtered (0.22 µm) PBS buffer. The solutions were mixed and analyzed at room temperature on Zetasizer Nano ZS90 (Malvern Instruments) with 4 mW red laser (632.8 nm) at a scattering angle of 90°. The laser power is 100% and the integration time was 80 s. The measurements were recorded every 10 min for a total of 3 h.

### 4.9 Transmission Electron Microscopy (TEM)

Compounds in 10 mM stock solutions of each sample in DMSO were diluted to 100 µM in PBS buffer (pH = 7.4) and incubated at 37°C for 24 h. Carbon‐coated grids that are previously subjected to glow discharge were used (Ted Pella, Catalog No. 01701‐F) and 3.5 μL of sample aliquots were placed onto carbon‐coated grids. After a 30 s incubation, the grids were rinsed with Milli‐Q water, followed by staining with 3.5 μL of 2% uranyl acetate for 30 s. Blotting and air‐drying were performed thereafter. Images were captured using a Thermo Fisher Scientific Glacios cryo‐TEM operating at 200 kV and equipped with a Ceta 16 MP CCD detector, utilizing magnifications ranging from 73,000 to 92,000× at a defocus of −2.5 to −4.0 μm.

### 4.10 Cryo‐EM Grids Preparation

The compounds were prepared similarly as for the TEM grids preparation and 3.5 µL of samples were gently deposited on previously glow‐discharged Quantifoil R1.2/1.3 300 mesh grids, then fast‐plunged into liquid ethane using a Thermo Fisher Scientific (TFS) Vitrobot Mark IV set at 100% humidity and 22°C, and blotted for 2.5 s. Imaging session was performed at the UCSC Biomolecular cryo‐EM Facility using a TFS Glacios 200 kV coupled to a TFS Falcon4i. The 4,227 for **morti07** and 1,025 images for mortiamide A were acquired at an accelerating voltage of 200 kV and nominal magnification of ×92,000, giving a calibrated pixel size of 1.6 Å, and total fluence of 40  e/Å^2^.

### 4.11 Single‐Particle Cryo‐EM Data Processing and Helical Reconstruction

Preprocessing was performed using cryoSPARC v.4.5, including motion correction and CTF estimation. After movie curation, fibril picking was performed within the 20–200 Å range, and the resulting particles were cropped to 800 pixels. These particles underwent additional selection through 2D classification, leading to 10,320 particles that were subsequently used for the initial 3D reconstruction and further helical refinement. Particles assigned to the representative group underwent another round of 2D classification to enhance cleanliness before undergoing nonuniform refinement. The resulting map achieved a final ‘gold‐standard’ resolution of ∼9.1 Å. Similarly, the round particles were obtained from the initial 1025 images from mortiamide A and, after pre‐processed, a total of 137,312 particles were identified. After several rounds of 2D and 3D classification, the final non‐uniform refinement provided an overall resolution (FSC_0.143_) of ∼9.3 Å

### 4.12 Cell Lines

SYO‐1 cell line was obtained from the Vaske lab (Department of Molecular, Cell, and Development Biology, University of California, Santa Cruz), and MDA‐MB‐453 was purchased and authenticated from the UCSF Cell and Genome Engineering Core (CCLZR289). SYO‐1 was maintained in minimum essential medium (MEM, Corning, 10‐010‐CM) with 10% fetal bovine serum (FBS, Gibco, 16000‐044) and 1% penicillin‐streptomycin (Gibco, 15070063). MDA‐MB‐453 was maintained in Dulbecco's modified Eagle's medium (DMEM, Gibco, 11965‐092) with 10% fetal bovine serum (FBS, Gibco, 16000‐044) and 1% penicillin‐streptomycin (Gibco, 15070063). Both cell lines were grown at 37°C and 5% CO_2_.

### 4.13 Cell Viability Assay

Cell viability assays were tested in the 384‐ or 1536‐well formats. SYO‐1 and MDA‐MB‐453 were cultured as described in the Cell lines section. In 1536‐well microplate, SYO‐1 or MDA‐MB‐453 cell lines were seeded at a density of 200 and 400 cells/well (4 µL), respectively, using the Biotek EL406 (Agilent). After ∼6 h, compounds in DMSO stock solutions were dosed at the 0.3% v/v DMSO concentration (12.5  nL) using the Echo 650 liquid handler (Beckman Coulter). Cells were incubated for 72 h at 37°C with 5% CO_2_ atmosphere. CellTiter‐Glo reagents (Promega) were added to each well (3 µL), and the plates were centrifuged for 1 min and incubated at room temperature in the dark for 10 min. Luminescence was measured using the Envision plate reader (Perkin Elmer). The percent inhibition values were calculated by subtracting the luminescence intensities of the sample wells from the average background (no cells) and comparing these values to the controls (DMSO‐treated wells). For 384‐well plate, SYO‐1 and MDA‐MB‐453 were seeded at a density of 2000 and 3000 cells/well (30 µL), respectively. The samples were dosed at 100  nL, and the CellTiter‐Glo reagents were added at 20 µL. All other parameters were the same as those outlined for the 1536‐well format.

### 4.14 Conformational Search McMD Simulations

The procedure of the conformational search was same as previous report [55]. Briefly, flat potential energy was obtained corresponding to the temperature between at *T* = 280 and 1505 K, and after the production run, 3.36  million conformers were obtained. The free energy landscape was calculated using this ensemble, and 5000 structures at *T* = 300 K were used to evaluate the backbone RMSD relative to the crystal structure.

## Supporting Information

Additional supporting information can be found online in the Supporting Information section. The authors have cited additional references within the Supporting Information [64, 65, 66].

## Funding

This work was supported by National Institutes of Health (R35GM148282, 1S10OD028730, S10OD02509, R01GM129325, and MRI2018501).

## Conflicts of Interest

The authors declare no conflicts of interest.

## Supporting information

Supplementary Material

## Data Availability

The data that support the findings of this study are available in the supplementary material of this article.
