# Supplementary material for: Exploring Passive Permeability Profiles of Cyclic Heptapeptide Chemical Space Uncovers Bioactivity of Mortiamide Scaffold Driven by Colloidal Aggregation
Source: Chembiochem. 2026 May 14;27(9):e70329. doi: 10.1002/cbic.70329 (PMC13173399; doi:10.1002/cbic.70329)
Supplement: Supplementary file 1 — Supplementary Material [file CBIC-27-e70329-s001.pdf]

Supporting Information

Exploring Passive Permeability Profiles of Cyclic Heptapeptide Chemical Space Uncovers Bioactivity of Mortiamide Scaffold Driven by Colloidal Aggregation

Jaru Taechalertpaisarn,[a, d] Alexander Engstrom,[a] Maria Sajimon,[a] Beverley M. Rabbitts,[a] Vitor H. Balasco Serrão,[a, b] Satoshi Ono,[c] Jevgenij A. Raskatov,[a] Timothy C. Johnstone,[a] R. Scott Lokey*[a]

[a] Department of Chemistry and Biochemistry, University of California, Santa Cruz, 1156 High Street, Santa Cruz, California 95064, United States

[b] Biomolecular Cryo-Electron Microscopy Facility, University of California, Santa Cruz, 1156 High Street, Santa Cruz, California 95064, United States

[c] Discovery Technology Laboratories, Innovative Research Division, Mitsubishi Tanabe Pharma Corporation, 1000 Kamoshida-cho, Aoba-ku, Yokohama, Kanagawa 227-0033, Japan

[d] Department of Science, Technology and Innovation, Faculty of Science, Chulabhorn Royal Academy, Bangkok, 10210, Thailand

*Correspondence to slokey@ucsc.edu

Table of Contents

Design of Cyclic Heptapeptide Libraries for Lipophilicity Study S3
Synthesis Scheme of Cyclic Heptapeptide Libraries for Lipophilicity Study S4
An Example of Stereoindexing from Circular Permutations and Enantiomers of DLLLLLL-Cyclic Heptapeptide S5
Synthesis Scheme of a One-Bead-One-Compound (OBOC) Library S6
Synthesis Scheme of Individual Cyclic Heptapeptides by Standard Solid-Support Resins S8
Synthesis Scheme of Individual Cyclic Heptapeptides by SynPhase Lanterns S9
Structures of Mortiamide Natural Product S11
Lipophilicity Distribution of a One-Bead-One-Compound Cyclic Heptapeptide Library S11
Purity Assessment of OBOC Library S12
Hit Selections S22
Inhibition of MDH Activity by **7-A⁶** Cyclic Heptapeptide S32
Size Distribution by Dynamic Light Scattering S33
Hydrogen Bond Network of Mortiamide B Enantiomer in the Crystal Lattice S34
Comparison of Backbone Conformations of Cyclic Heptapeptides with Diverse Side Chains S34
Backbone Hydrogen Bond Pattern of Mortiamide A S35
General Procedure of X-ray Structure Determination S35
Supplemental Figures of TEM and CryoEM S38
Experimental Data S42
NMR Spectra S46
LC-MS Spectra S65
References S113

Design of Cyclic Heptapeptide Libraries for Lipophilicity Study

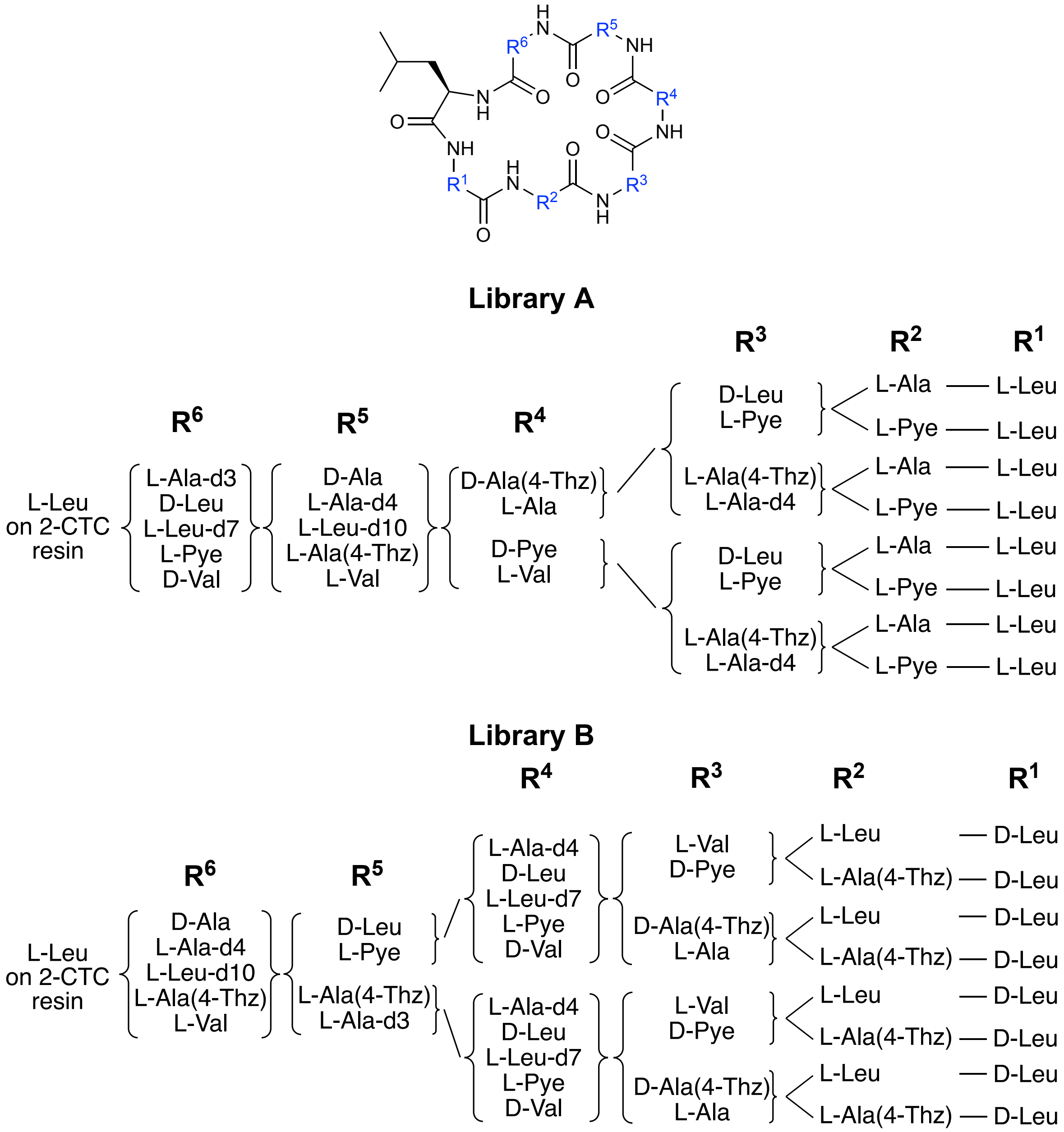

Scheme S1. Design of split-and-pool synthesis for the log $D_{dec/w}$ partitioning experiment. Structures of side chains are shown in *Scheme S4*.

Synthesis Scheme of Cyclic Heptapeptide Libraries for Lipophilicity Study

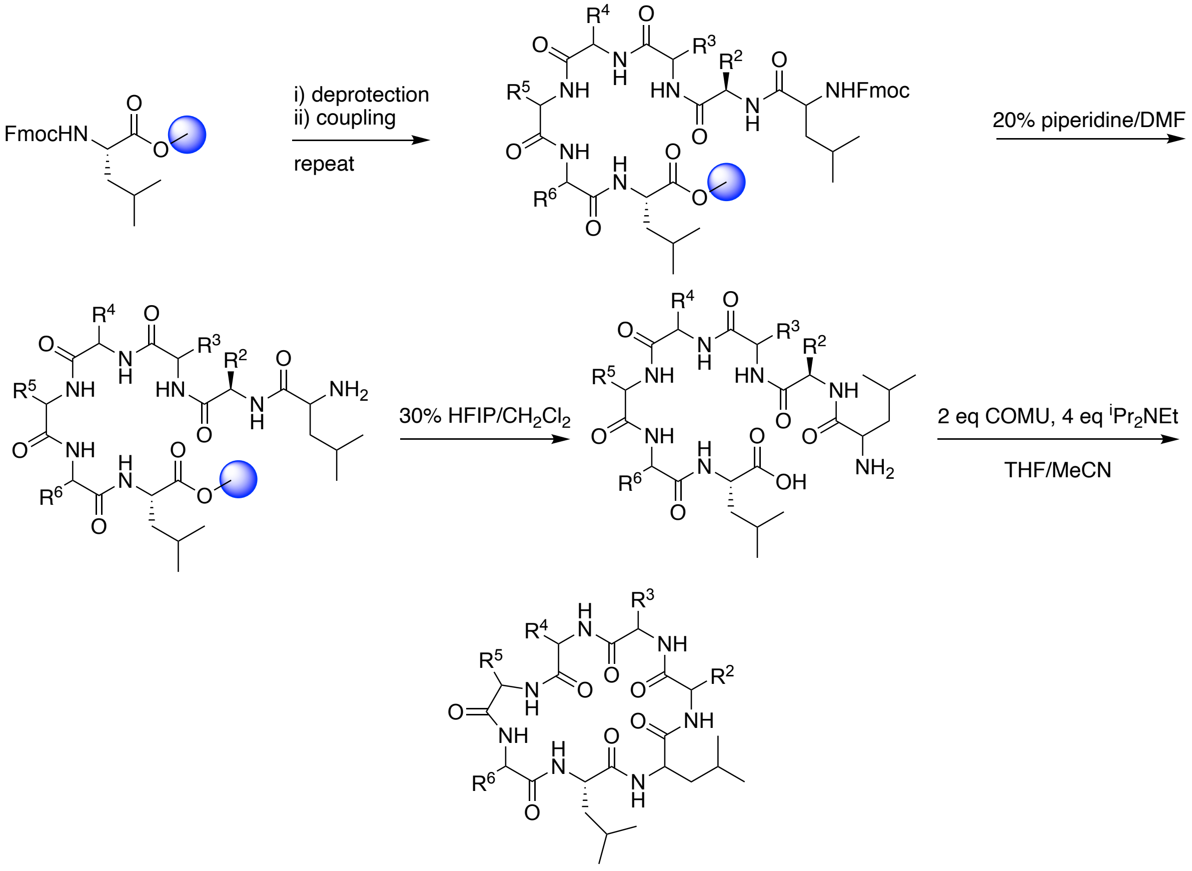

Scheme S2. Synthesis scheme of cyclic heptapeptides for the partitioning study. Deprotection: 20% piperidine in DMF, 25 ºC, twice (20+10 min); Coupling: 4 eq amino acids, 4 eq HATU, 8 eq $^{i}Pr_2NEt$ in DMF, 25 ºC, 1 h.

Synthesis of Cyclic Heptapeptide Libraries for Lipophilicity Study. Libraries A and B were synthesized by the split-and-pool method as described. Commercial pre-loaded 2-chlorotrityl-L-leucine resin (0.3 mmol) was split equally according to *Scheme S1*. Couplings were performed using Fmoc-protected amino acids (4 eq, 0.25 M), HATU (4 eq), and $^{i}Pr_2NEt$ (8 eq) in DMF at room temperature for 1 h. Resin was pooled together and washed thoroughly with 5x DMF. Then, Fmoc deprotection was performed by treating the resin with 20% piperidine in DMF twice for 20 and 10 minutes. The resin was washed successively with 5x DMF, 5x CH_2Cl_2, and 5x DMF. Once complete, resin was split according to *Scheme S1*, and the synthesis process was repeated. After coupling amino acids at the R^2 position, resins from each group remained separate, and the synthesis was continued to couple the R^1 amino acids. Linear heptapeptides from each sublibrary were cleaved in the 30% HFIP/CH_2Cl_2 mixture. Solutions were collected and dried completely, and the linear heptapeptides were cyclized by the solution of 2 eq COMU and 4 eq $^{i}Pr_2NEt$ in THF. The reactions were stirred for 24 h, and each sublibrary was purified by trap-and-elute method using the Biotage Isolute® 103 200 mg / 6 mL columns.

An Example of Stereoindexing from Circular Permutations and Enantiomers of DLLLLLL-Cyclic Heptapeptide

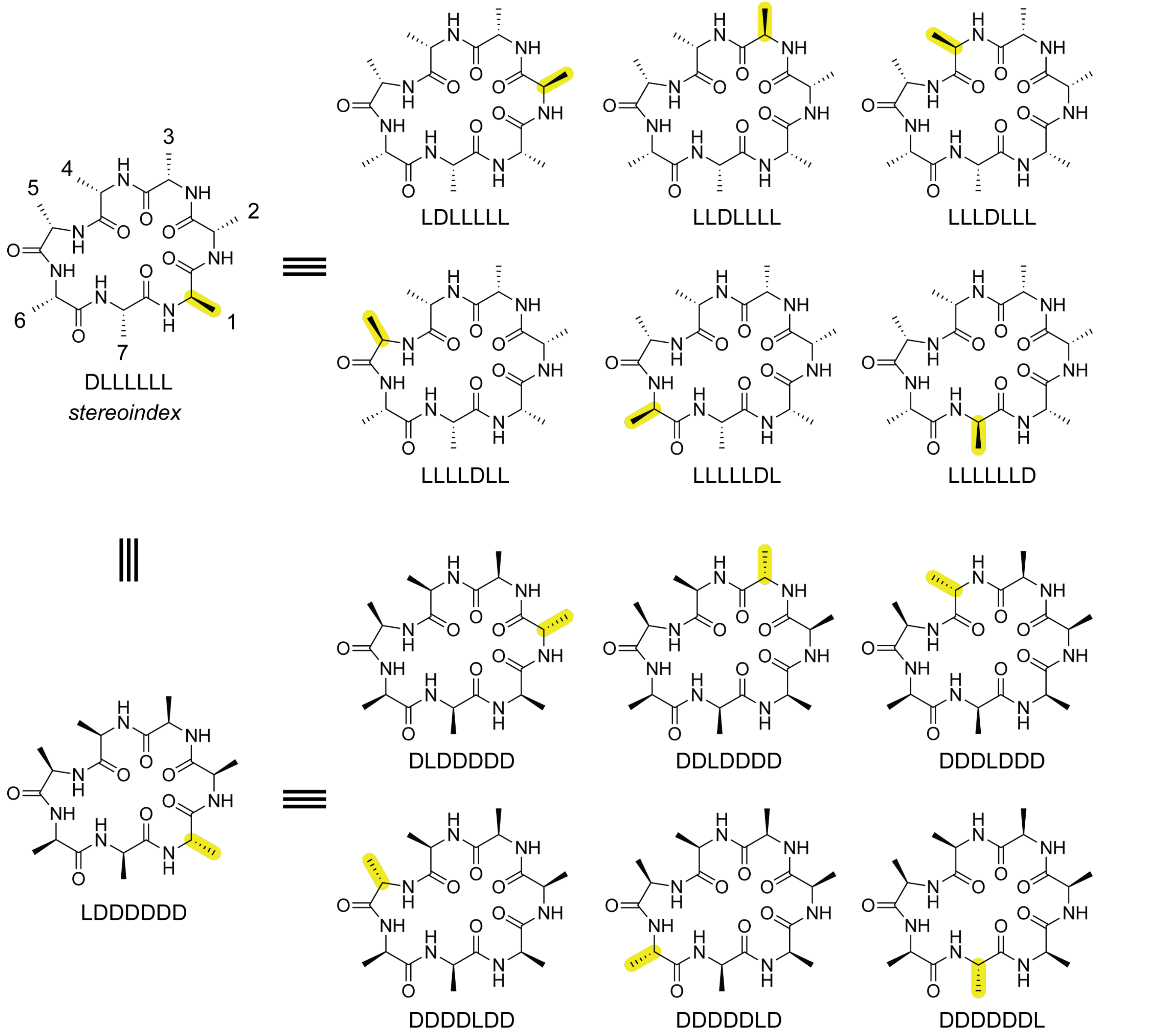

Scheme S3. An example of stereoindexing. A DLLLLLL-cyclic heptapeptide has six circular permutations (top two rows) and seven mirror images (bottom two rows). All 14 isomers have identical backbone geometries in an achiral environment and can be binned into the 'DLLLLLL' stereoindex.

Synthesis Scheme of a One-Bead-One-Compound (OBOC) Library

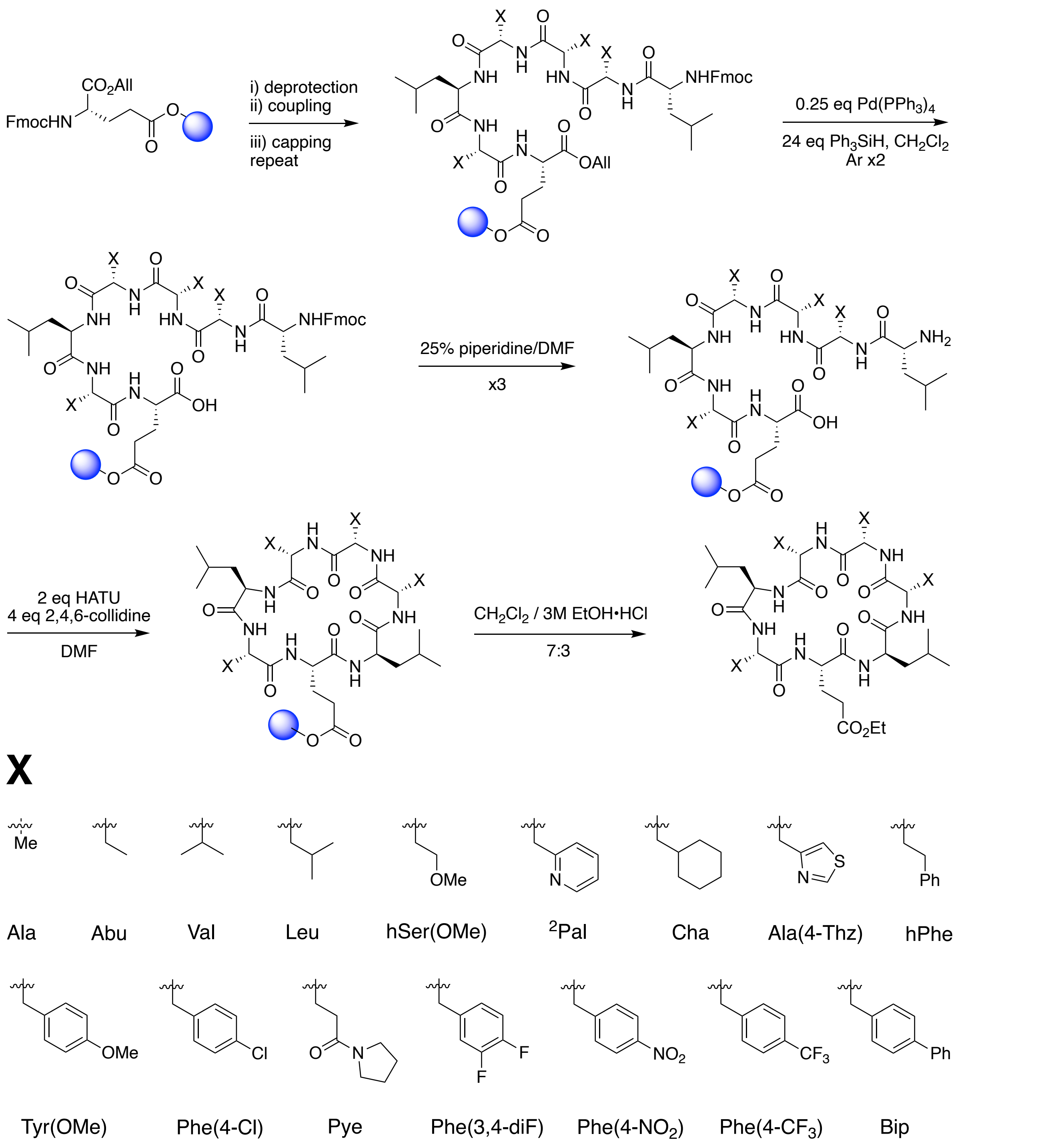

Scheme S4. Synthesis scheme of a one-bead-one-compound cyclic heptapeptide library. Deprotection: 2% DBU, 5% piperidine in DMF, 25 ºC, 30 min twice; Coupling: 4 eq amino acids, 3.8 eq HATU, 6 eq $^iPr_2NEt$ in DMF, 25 ºC, 3 h; Capping: Ac_2O, $^iPr_2NEt$, DMF, 10 min, 25 ºC.

Synthesis of a One-Bead-One-Compound (OBOC) Library. 2-Chlorotrityl chloride macrobeads (Rapp Polymere 250-315 μm) were swelled in the 1:1 CH_2Cl_2 and DMF mixture for 1 h, then L-Fmoc-Glu-OAll (1.1 eq), $^iPr_2NEt$ (2 eq) in DMF were added under argon atmosphere. The solution was mechanically shaken overnight and drained. Blocking unreacted resin was performed with a solution of 1:2:17 $^iPr_2NEt/MeOH/CH_2Cl_2$ for 30 minutes, and the process was repeated using a fresh blocking solution. Resin was washed with 5x

DMF, 3x CH_2Cl_2, 3x MeOH, 3x CH_2Cl_2, and dried under vacuum overnight. The loading capacity was determined from the standard Fmoc quantification protocol.

Approximately 2.5 g of pre-loaded L-Fmoc-Glu-OAll macrobeads were used for preparing the OBOC cyclic heptapeptide library. Fmoc deprotection was carried out with 2% DBU and 5% piperidine in DMF for 30 minutes twice. Resin was washed thoroughly with 6x DMF, 3x CH_2Cl_2, and 6x DMF. Macrobeads were split equally into 16 groups for amino acid couplings at variable positions (R^2, R^3, R^4, and R^6) or pooled into one group at the D-leucine positions (R^1 and R^5). Couplings were performed using Fmoc-protected amino acids (4 eq), HATU (3.8 eq), and $^iPr_2NEt$ (6 eq) in DMF for 3 h. The reactions were assessed by the Kaiser test to determine the completeness, and the coupling was repeated if the test was positive. Resin was combined and washed with 6x DMF, 3x CH_2Cl_2, and then washed again with 6x DMF. Unreacted amines were capped with a 1:1 solution of 20% Ac_2O and 1 M $^iPr_2NEt$ in DMF for 10 minutes. The deprotection-coupling-capping steps were repeated until the Fmoc-protected linear heptapeptides were completely synthesized.

On-bead cyclization was carried out in the solid-phase peptide synthesis vessel. Resins were swelled in CH_2Cl_2 under an argon atmosphere. The solution of $Pd(PPh_3)_4$ (0.25 eq) and $PhSiH_3$ (24 eq) in CH_2Cl_2 was added to the resin while the solution was bubbled with argon continuously. The vessel was sealed, protected from light, and mechanically shaken under argon for 2 h. The solution was drained, and the process was repeated with a fresh deallylation solution. Palladium complexes were washed 3 times with the solution of 0.02 M sodium *N*,*N*-diethyldithiocarbamate in DMF for 20 minutes each. Then, resin was washed with 5x DMF, 5x CH_2Cl_2, 5x DMF, and the Fmoc deprotection was carried out with 25% piperidine in DMF for 30 minutes three times. The deprotection solution was drained, and the resin was washed three times with 20% $^iPr_2NEt$ in DMF for 20 minutes each to remove excess piperidine salt. After washing resin beads with 5x DMF, 5x CH_2Cl_2, and 5x DMF, the head-to-tail cyclization on the resin was performed with HATU (2 eq) and 2,4,6-collidine (4 eq) in DMF overnight. Resin was washed successively with 5x DMF, 3x CH_2Cl_2, 3x MeOH, 3x CH_2Cl_2, and completely dried under vacuum overnight.

Approximately 5 -10 macrobeads of cyclic heptapeptides were distributed to 96-well plates, and the 10 µL solution of 3M HCl in EtOH and CH_2Cl_2 (3:7) was added to each well. Microplates were sealed and shaken overnight. Mixtures were neutralized by the addition of sodium carbonate pellets and were filtered through 0.7 µm glass fiber filter plates (Agilent 200965-100) by dissolving crude materials in 100 µL of $CHCl_3$. Solutions were concentrated with a Genevac centrifugal evaporator, and cyclic heptapeptides were resuspended in 10 µL DMSO. Stock solutions were transferred to the 384-well low dead volume cyclic olefin copolymer source microplates (Beckman Coulter; product no. 001-12782) for further screening.

Synthesis Scheme of Individual Cyclic Heptapeptides by Standard Solid-Support Resins

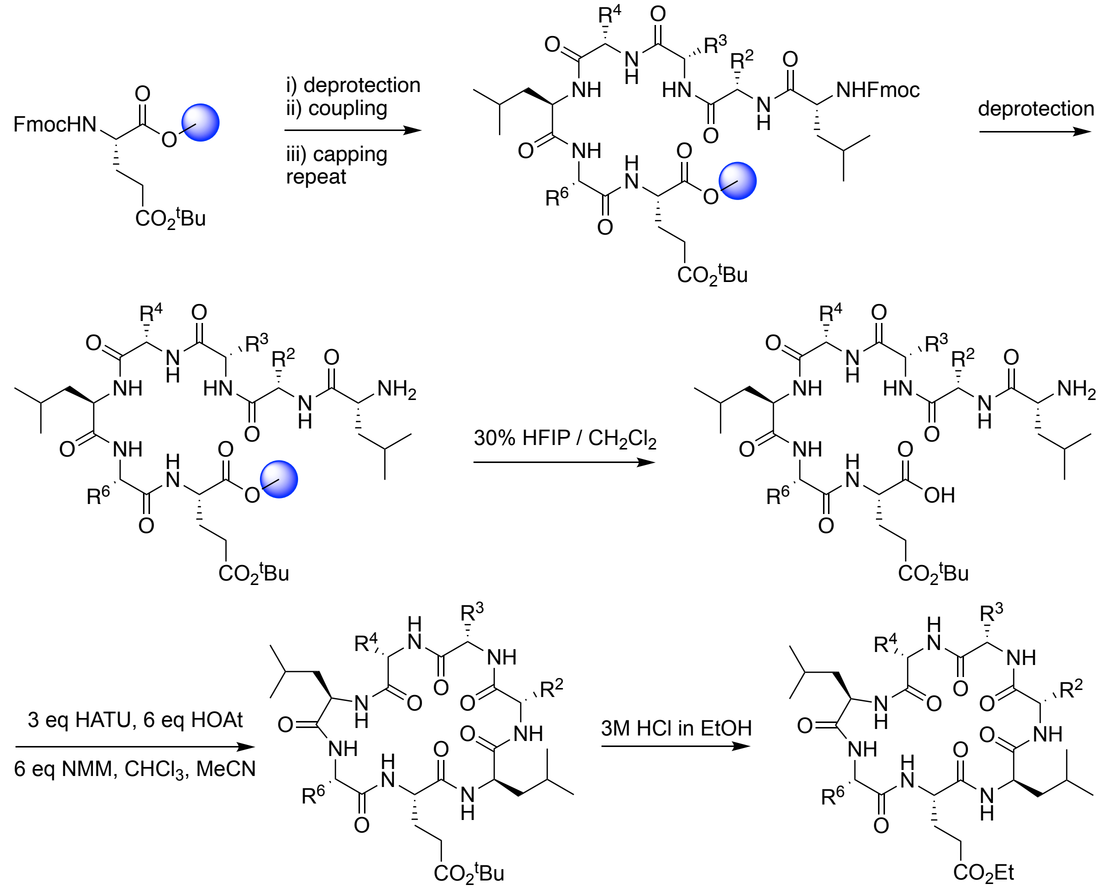

Scheme S5. Synthesis scheme of individual cyclic heptapeptides by solid-support resins. Deprotection: 2% DBU, 5% piperidine in DMF, 75 ºC, 10 min twice; Coupling: 4 eq amino acids, 3.8 eq HATU, 6 eq $^{i}Pr_2NEt$ in DMF, 75 ºC, 30 min; Capping: Ac_2O, $^{i}Pr_2NEt$, DMF, 5 min, 25 ºC.

Synthesis of Individual Cyclic Heptapeptides by Standard Solid-Support Resins. Linear peptides were synthesized using an automated peptide synthesizer (Prelude X, Protein Technologies). Pre-loaded Fmoc-amino acid on 2-chlorotrityl resin was used at a 0.05 mmol scale. Fmoc deprotection was carried out with 2% DBU and 5% piperidine in DMF for 10 minutes at 75 °C twice. Couplings were performed using Fmoc-protected amino acids (4 eq), HATU (3.8 eq), and $^{i}Pr_2NEt$ (6 eq) in DMF (0.1 M with respect to amino acid) for 30 minutes at 75 °C. A capping step was performed after each coupling step with a 1:1 mixture of 20% Ac_2O and 1 M $^{i}Pr_2NEt$ in DMF. Each coupling, deprotection, and capping step was followed by washing steps with DMF (6x), CH_2Cl_2 (3x), and DMF (3x). Complete linear peptides were cleaved off the resin with 30% HFIP in CH_2Cl_2 for 1 h three times, with a CH_2Cl_2 elution equivalent to 5 resin volumes in between each step. Solvents were removed under compressed air to obtain linear peptides as solids.

Crude linear peptides (1 eq) and HOAt (6 eq) were dissolved in a mixture of chloroform and acetonitrile (2:1, 30 mL). *N*-Methylmorpholine (6 eq) was added followed by HATU (3 eq). The reactions were stirred for 12-24 h until complete cyclization was achieved as monitored by LC-MS. The solutions were reduced in vacuo and purified by reverse-phase column chromatography using Biotage Isolera Prime equipped with Biotage Sfär Bio C18 D 10 g column eluting with the 20-100% MeCN/H_2O gradient modified with 0.1% TFA over 20 column volumes at a flow rate of 25 mL/min.

Synthesis Scheme of Individual Cyclic Heptapeptides by SynPhase Lanterns

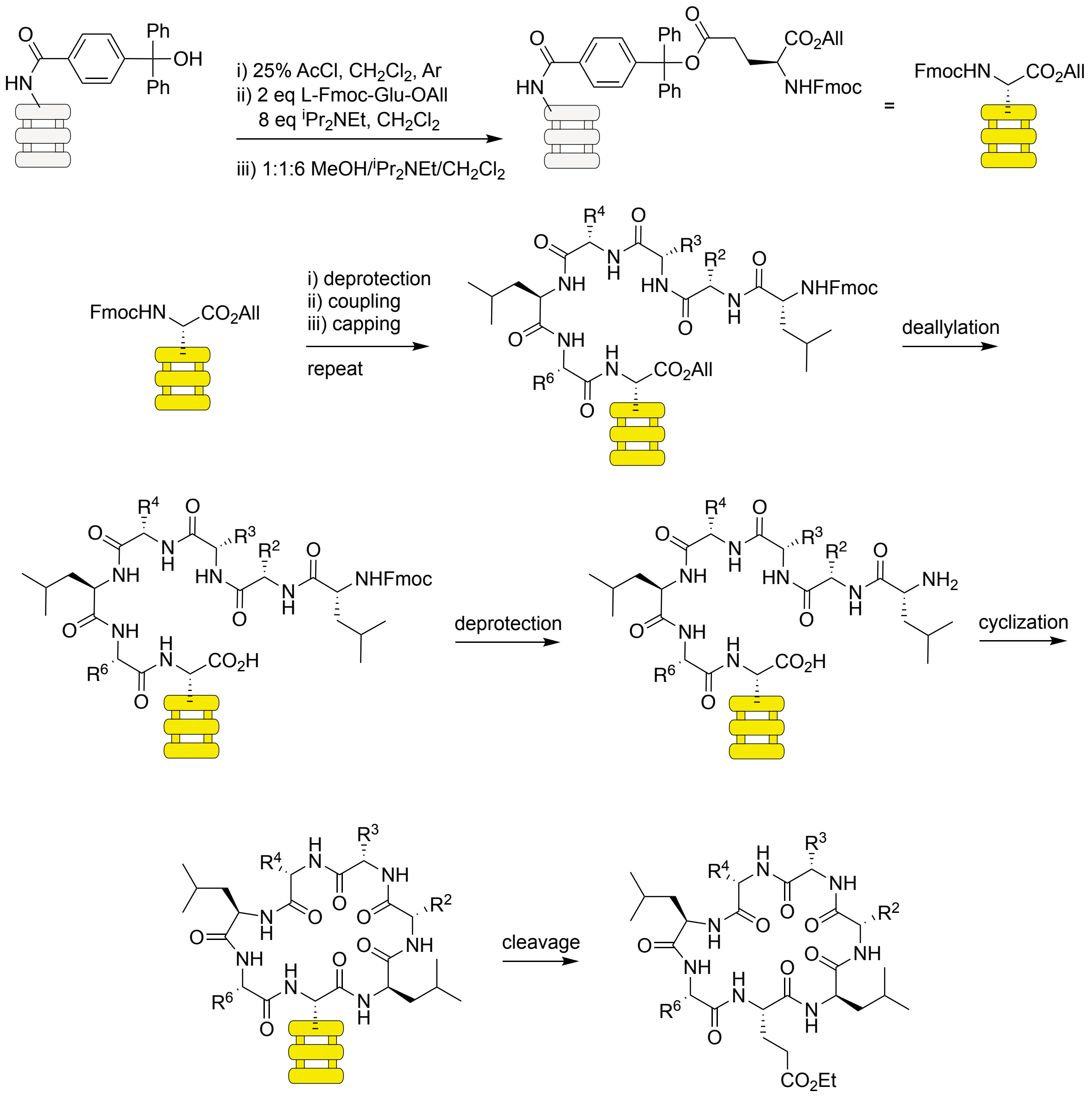

Scheme S6. Synthesis scheme of individual cyclic heptapeptides by SynPhase Lanterns. Deprotection: 20% piperidine in DMF, 25 ºC, 2+2+20 min; Coupling: 10 eq amino acids, 10 eq HATU, 20 eq $^iPr_2NEt$ in DMF, 70 ºC, 1 h; Capping: Ac_2O, $^iPr_2NEt$, DMF, 30 min, 25 ºC; Deallylation: 1.8 eq $Pd(PPh_3)_4$, 15 eq NMM, 15 eq AcOH in CH_2Cl_2; Cyclization: 6 eq HATU, 12 eq 2,4,6-collidine in DMF, 50 ºC, 1 h; Cleavage: 4M HCl in dioxane and EtOH (3:1), 25 ºC, overnight.

Synthesis of Individual Cyclic Heptapeptides by SynPhase Lanterns. In a large SPE tube, L-series trityl alcohol SynPhase Lanterns (100 pieces, Mimotopes) were soaked in dry CH_2Cl_2 for 30 minutes under argon atmosphere. Solvent was drained under positive pressure, and lanterns were soaked again in dry CH_2Cl_2. After removing the solvent, lanterns were activated by the addition of 30 mL of dry CH_2Cl_2 and 10 mL of AcCl, and lanterns were shaken under an argon atmosphere for 24 h. The solution was drained under positive pressure, and lanterns were washed by soaking in dry CH_2Cl_2 3 times for 20 minutes each. A solution of L-Fmoc-Glu-OAll (2 eq), $^iPr_2NEt$ (8 eq) in dry 30 mL CH_2Cl_2 was added, and lanterns were shaken for 24 h. After draining the coupling solution, lanterns were briefly washed 3 times with CH_2Cl_2, and unreacted lanterns were blocked by a capping solution of 30:5:5 CH_2Cl_2/ $^iPr_2NEt$/MeOH for 30 minutes. The blocking solution was drained, and lanterns were washed thoroughly with 3x CH_2Cl_2, 3x DMF, 3x

CH_2Cl_2, and dried under vacuum overnight. Lanterns were cut in half and inserted with RFID glass tags, and their identification numbers were paired with the corresponding amino-acid sequences. Each lantern contains approximately 5 μmol of L-Fmoc-Glu-OAll.

Dried lanterns were swollen in CH_2Cl_2 for 10 minutes and drained. The 20% piperidine in DMF solution was added to the lanterns and shaken three times (2 x 2 x 20 minutes) to deprotect Fmoc group. Lanterns were briefly washed three times with DMF and soaked for 4 minutes three times. Then, the process was repeated with CH_2Cl_2, and the lanterns were dried at 60 ºC for 10 minutes. Fmoc-amino acids (10 eq), HATU (10 eq), and $^iPr_2NEt$ (20 eq) in DMF (0.2 M) were added to the dried lanterns, and the solutions were incubated at 70 ºC for 1 h. Coupling solutions were drained, and the lanterns were washed with DMF and CH_2Cl_2. Poor coupling conversion was observed in every activated Fmoc-amino acid that reacted with the 2-pyridylalanine; therefore, a second coupling was required to complete the reaction. Once reactions were complete, lanterns were treated with the solution of 10% Ac_2O and 17.5% $^iPr_2NEt$ in DMF for 30 minutes and washed as described above.

A deallylation solution containing $Pd(PPh_3)_4$ (1.8 eq), *N*-methylmorpholine (15 eq), and acetic acid (15 eq) in CH_2Cl_2 was prepared under argon atmosphere. The solution was transferred to the SPE tube containing lanterns under argon, and the solution was protected from light and shaken overnight. Lanterns were washed with DMF, CH_2Cl_2, and DMF, and excess palladium complexes were removed by treating with a solution of 2.5% sodium *N*,*N*-diethyldithiocarbamate and 2.5% *N*-methylmorpholine in DMF three times for 20 minutes each. After washing with DMF, excess sodium *N*,*N*-diethyldithiocarbamate was removed with a 20% H_2O/DMF solution for 20 minutes twice. Lanterns were then washed with 3x DMF, 3x CH_2Cl_2, and dried. After deprotecting the Fmoc group as described above, lanterns were washed with 20% $^iPr_2NEt$ in DMF three times for 20 minutes each. Cyclization solution containing HATU (6 eq) and 2,4,6-collidine (12 eq) in DMF was added to the lanterns, and the reactions were incubated at 50 ºC for 1 h. The solution was drained, and the washing step was performed as described above.

To prepare stock solutions in the microplates, each lantern was distributed to the 96-well 2.0 mL deep well plates, and the 250 μL mixture of 4M HCl/dioxane and EtOH (3:1) was added to each well. Plates were sealed and shaken overnight. Solutions were removed under dry compressed nitrogen gas. Crude materials were resuspended in 250 μL acetonitrile, sonicated for 10 minutes, and mixed well. The cyclic heptapeptide solutions (50 μL) were transferred to microplates, dried under compressed nitrogen, and resuspended in 100 μL of DMSO to yield approximately 10 mM stock solutions.

Structures of Mortiamide Natural Product

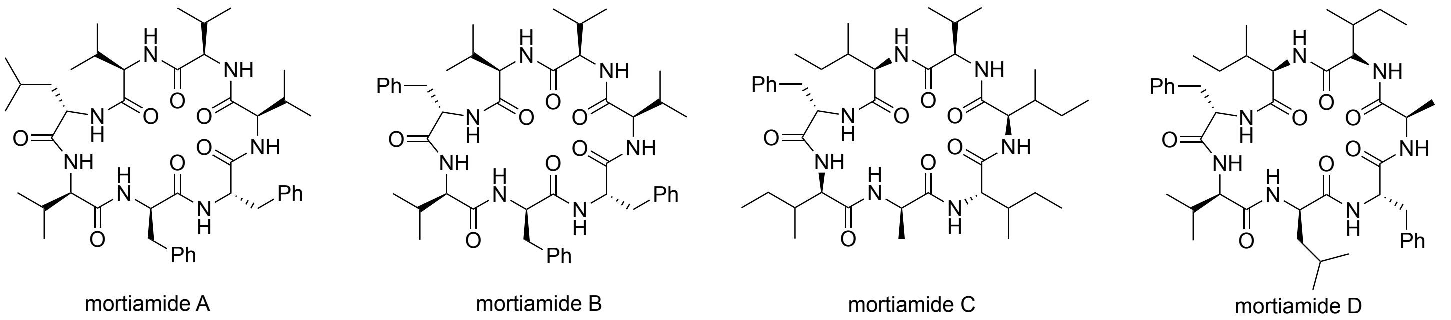

Figure S1. Structures of mortiamide natural product.

Lipophilicity Distribution of a One-Bead-One-Compound Cyclic Heptapeptide Library

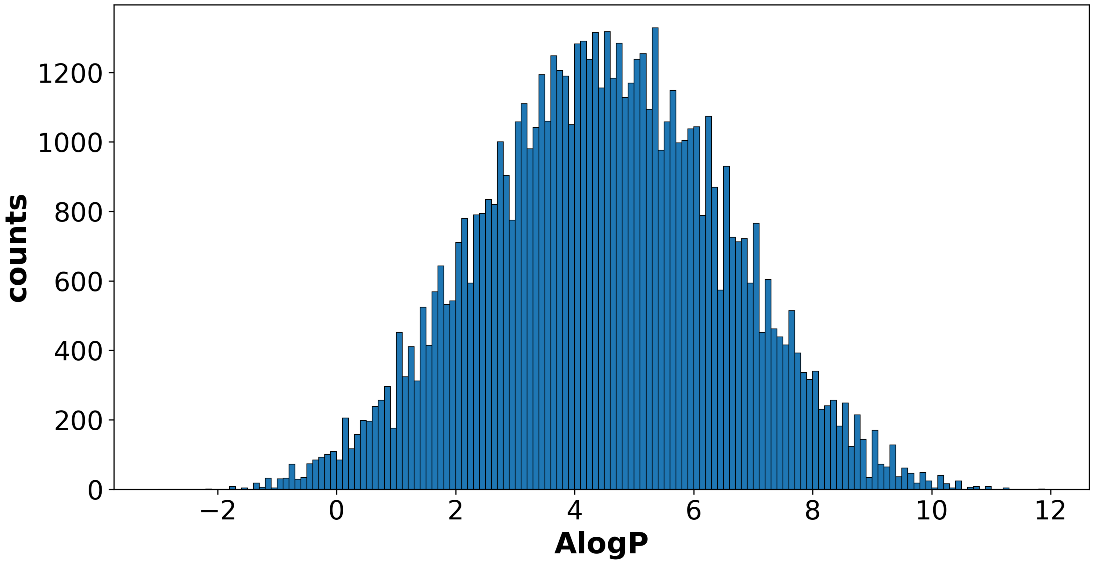

Figure S2. Calculated AlogP distribution of cyclic heptapeptide library. AlogP values range between -2 to 12 with a mean of 4.5.

Purity Assessment of OBOC Library

a

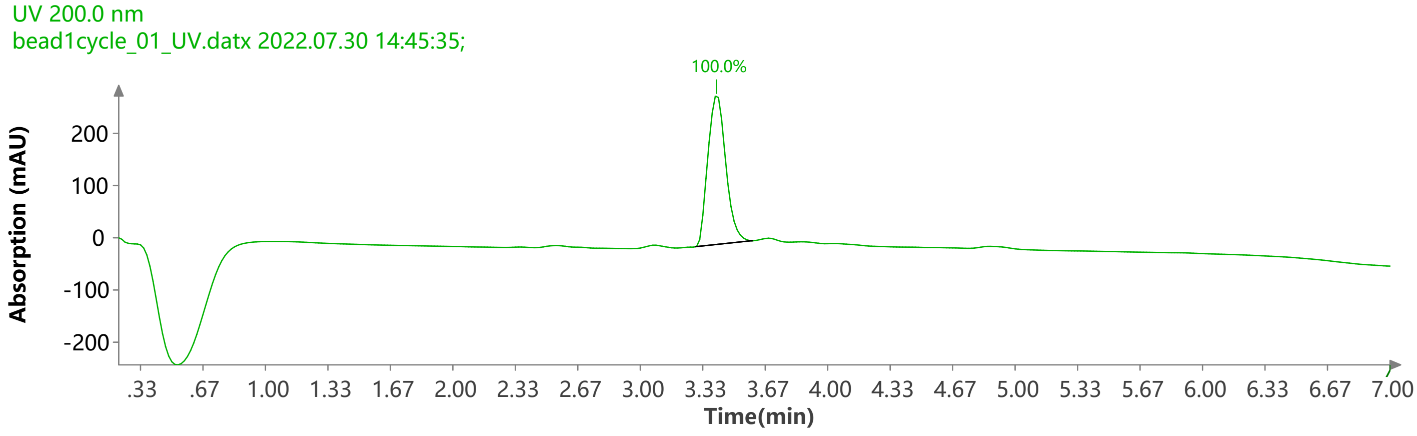

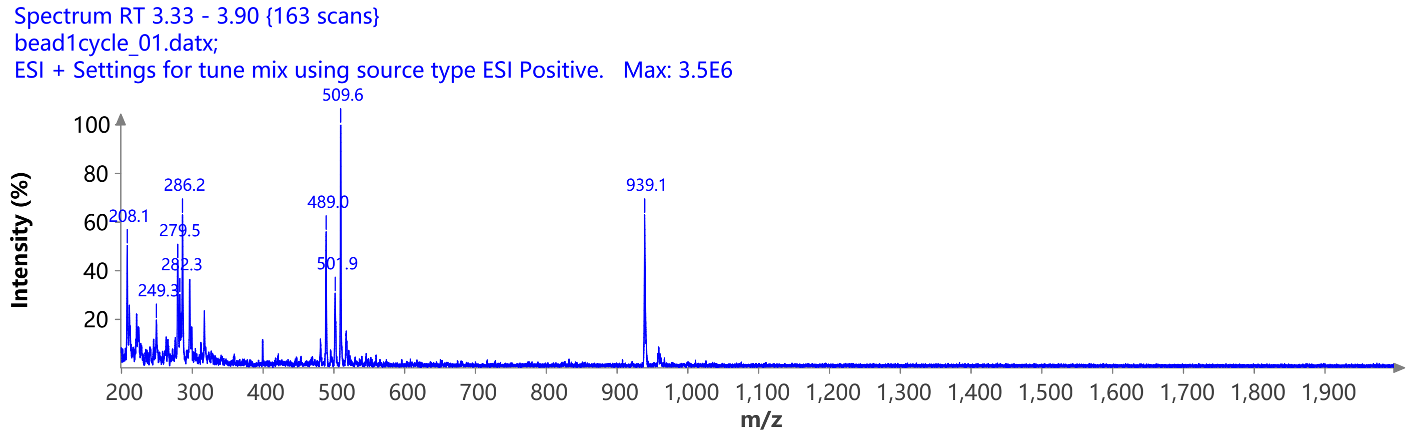

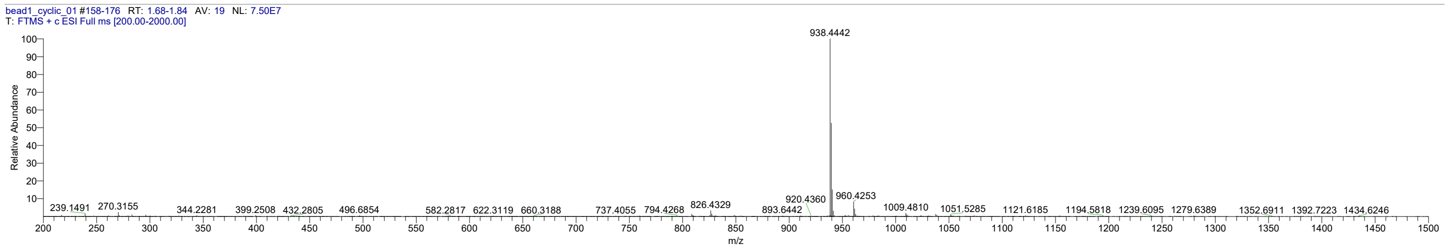

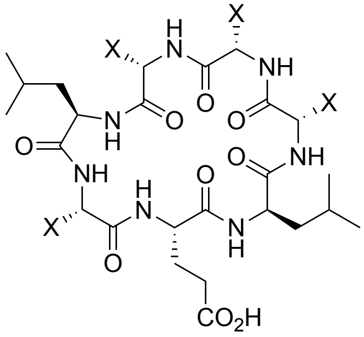

Possible sequence: [Ala, Leu, Phe(3,4-diF), Phe(4-CF_3)]
[Abu, Val, Phe(3,4-diF), Phe(4-CF_3)]

b

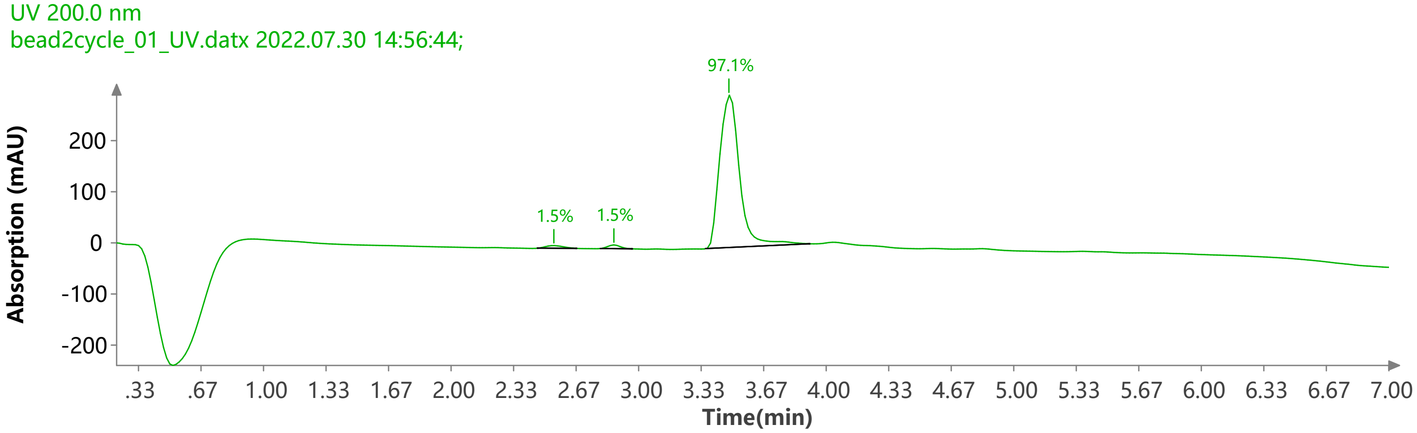

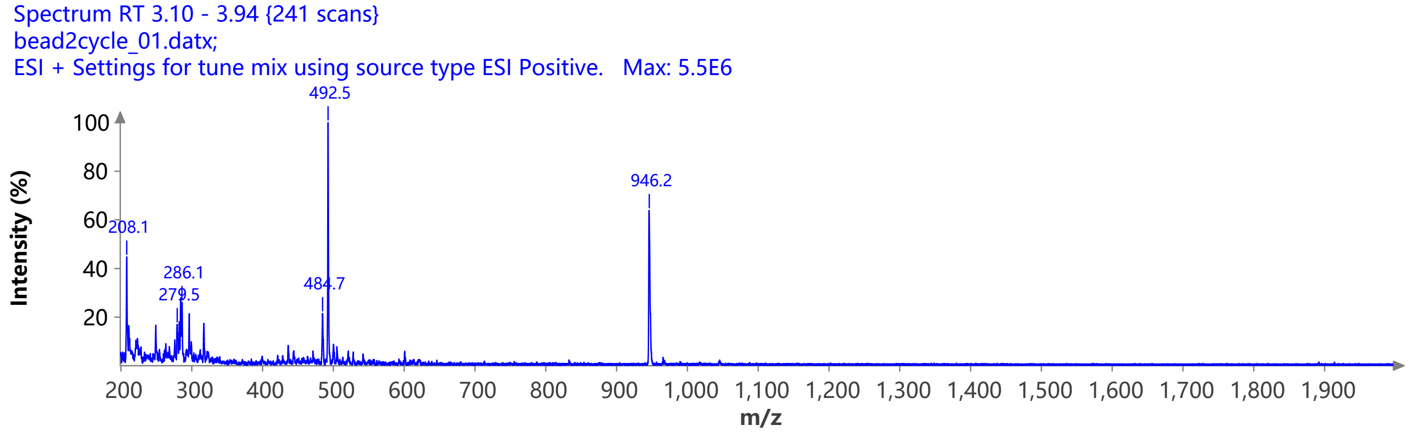

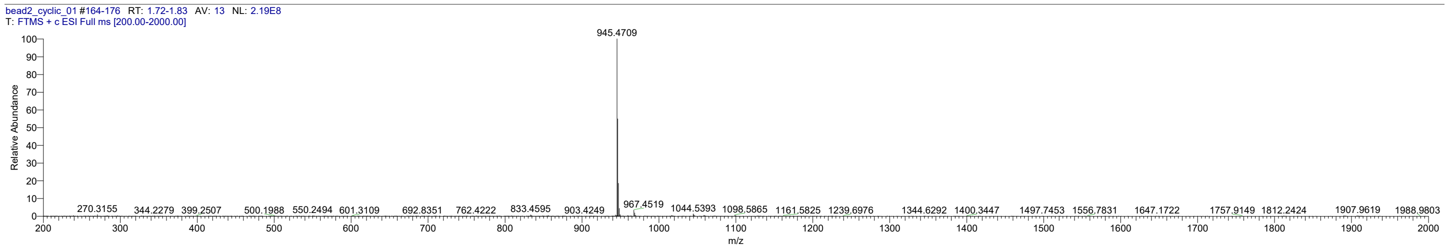

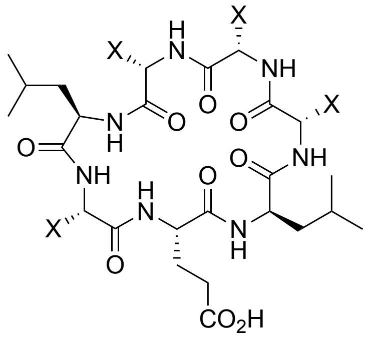

Possible sequence: [Val, Cha, Ala(4-Thz), Phe(3,4-diF)]

c

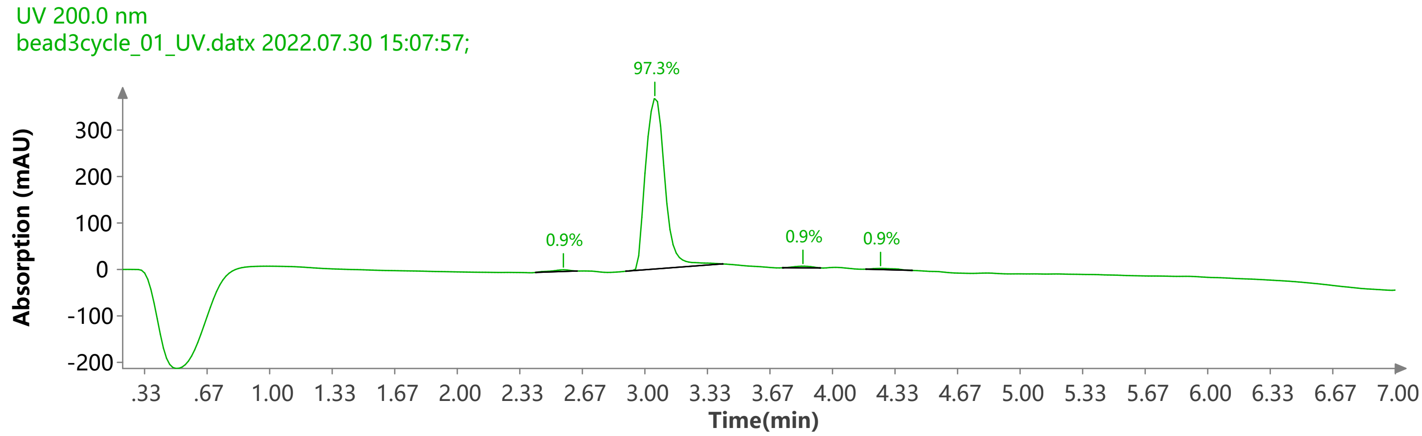

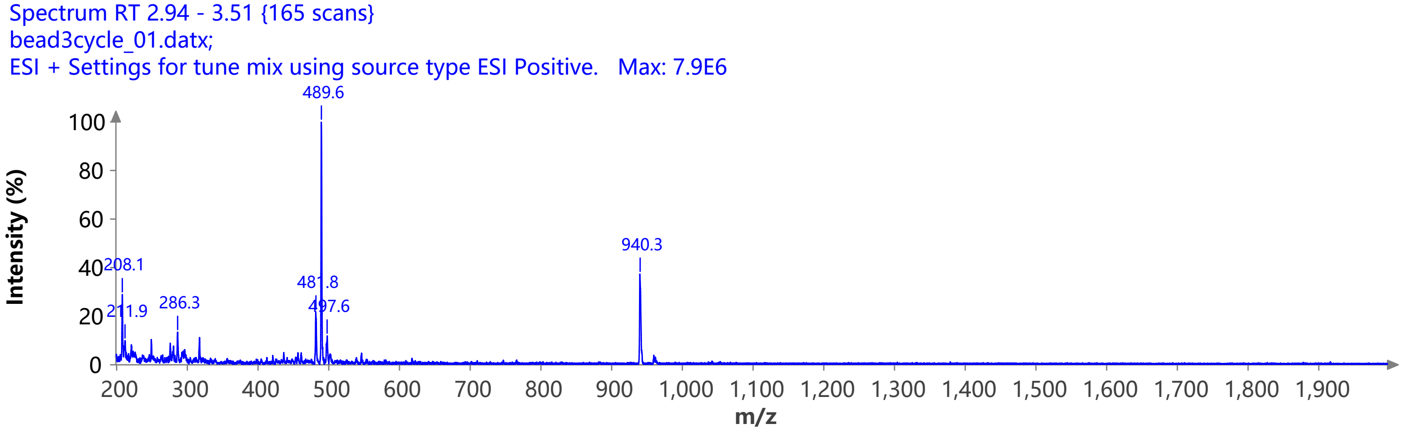

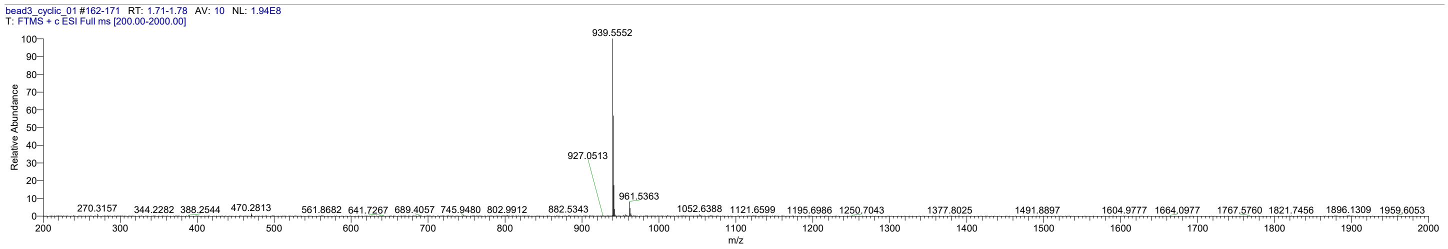

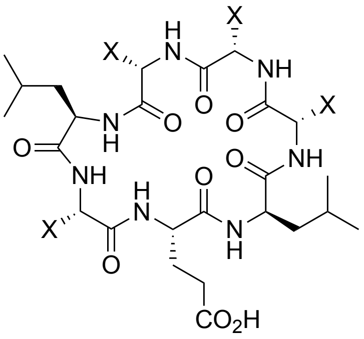

Possible sequence: [Ala, Cha, Tyr(OMe), Pye]
[Ala, Phe(4-NO_2), Cha, Cha]

d

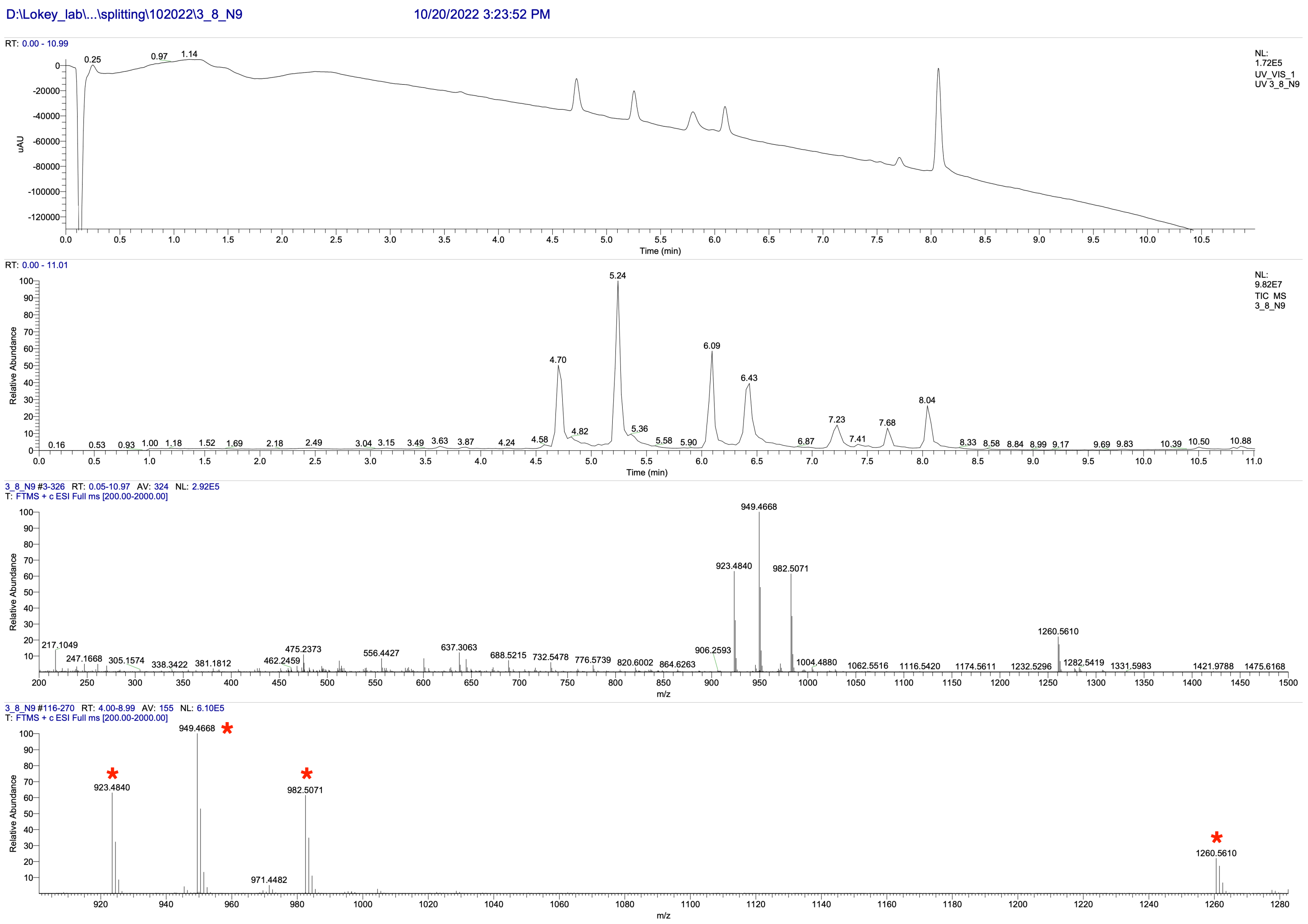
D:\Lokey_lab\...\splitting\102022\3_8_N9
10/20/2022 3:23:52 PM
RT: 0.00 - 10.99
NL: 1.72E5 UV_VIS_1 UV 3_8_N9
uAU
Time (min)
RT: 0.00 - 11.01
NL: 9.82E7 TIC MS 3_8_N9
Relative Abundance
Time (min)
3_8_N9 #3-326 RT: 0.05-10.97 AV: 324 NL: 2.92E5
T: FTMS + c ESI Full ms [200.00-2000.00]
949.4668
923.4840
982.5071
1260.5610
m/z
3_8_N9 #116-270 RT: 4.00-8.99 AV: 155 NL: 6.10E5
T: FTMS + c ESI Full ms [200.00-2000.00]
949.4668
923.4840
982.5071
971.4482
1260.5610
m/z

e

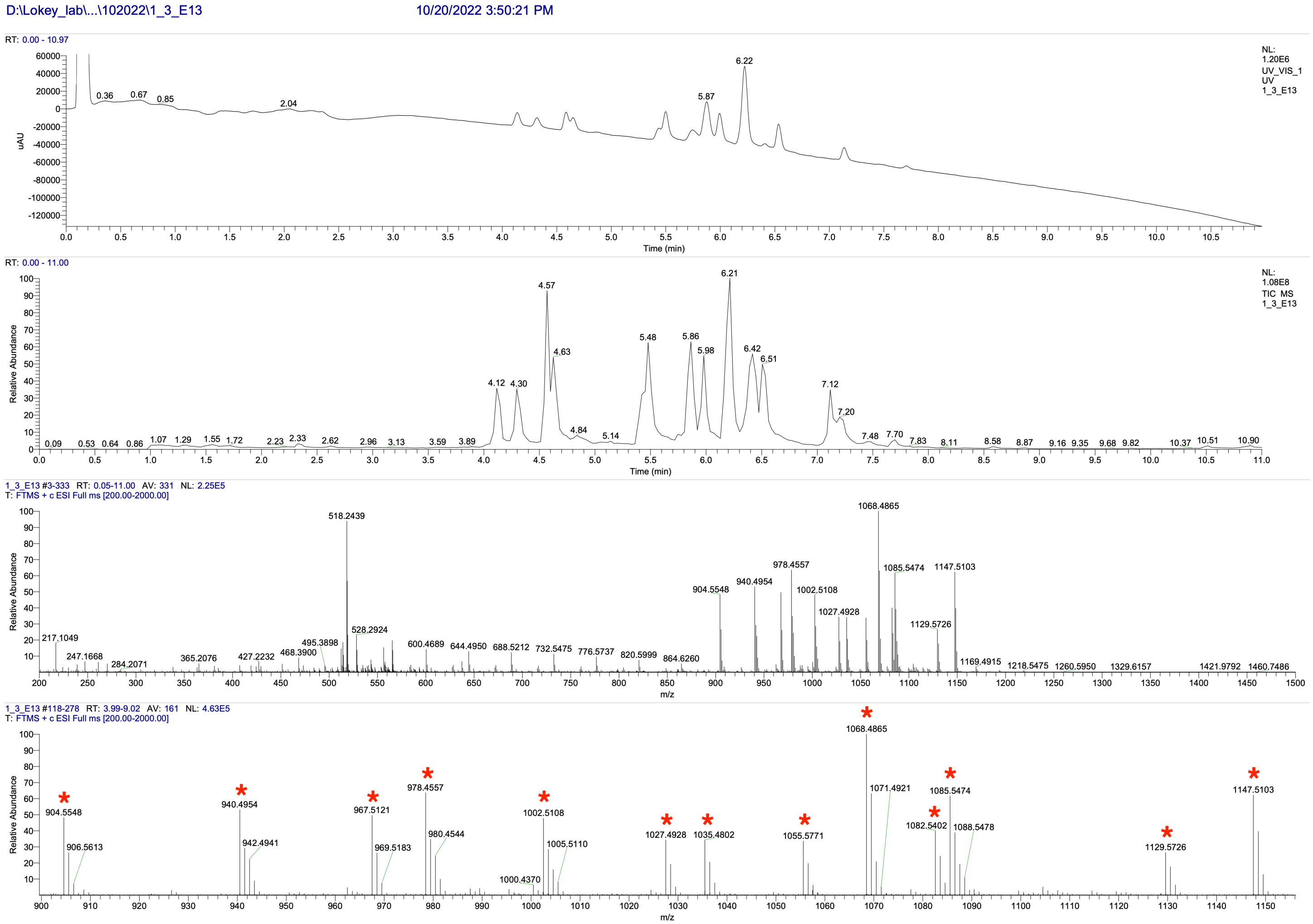
D:\Lokey_lab\...\102022\1_3_E13
10/20/2022 3:50:21 PM
RT: 0.00 - 10.97
NL: 1.20E6 UV_VIS_1 UV 1_3_E13
uAU
0.36
0.67
0.85
2.04
5.87
6.22
Time (min)
RT: 0.00 - 11.00
NL: 1.08E8 TIC MS 1_3_E13
Relative Abundance
0.09
0.53
0.64
0.86
1.07
1.29
1.55
1.72
2.23
2.33
2.62
2.96
3.13
3.59
3.89
4.12
4.30
4.57
4.63
4.84
5.14
5.48
5.86
5.98
6.21
6.42
6.51
7.12
7.20
7.48
7.70
7.83
8.11
8.58
8.87
9.16
9.35
9.68
9.82
10.37
10.51
10.90
Time (min)
1_3_E13 #3-333 RT: 0.05-11.00 AV: 331 NL: 2.25E5
T: FTMS + c ESI Full ms [200.00-2000.00]
Relative Abundance
217.1049
247.1668
284.2071
365.2076
427.2232
468.3900
495.3898
518.2439
528.2924
600.4689
644.4950
688.5212
732.5475
776.5737
820.5999
864.6260
904.5548
940.4954
978.4557
1002.5108
1027.4928
1068.4865
1085.5474
1129.5726
1147.5103
1169.4915
1218.5475
1260.5950
1329.6157
1421.9792
1460.7486
m/z
1_3_E13 #118-278 RT: 3.99-9.02 AV: 161 NL: 4.63E5
T: FTMS + c ESI Full ms [200.00-2000.00]
Relative Abundance
904.5548
906.5613
940.4954
942.4941
967.5121
969.5183
978.4557
980.4544
1000.4370
1002.5108
1005.5110
1027.4928
1035.4802
1055.5771
1068.4865
1071.4921
1082.5402
1085.5474
1088.5478
1129.5726
1147.5103
m/z

f

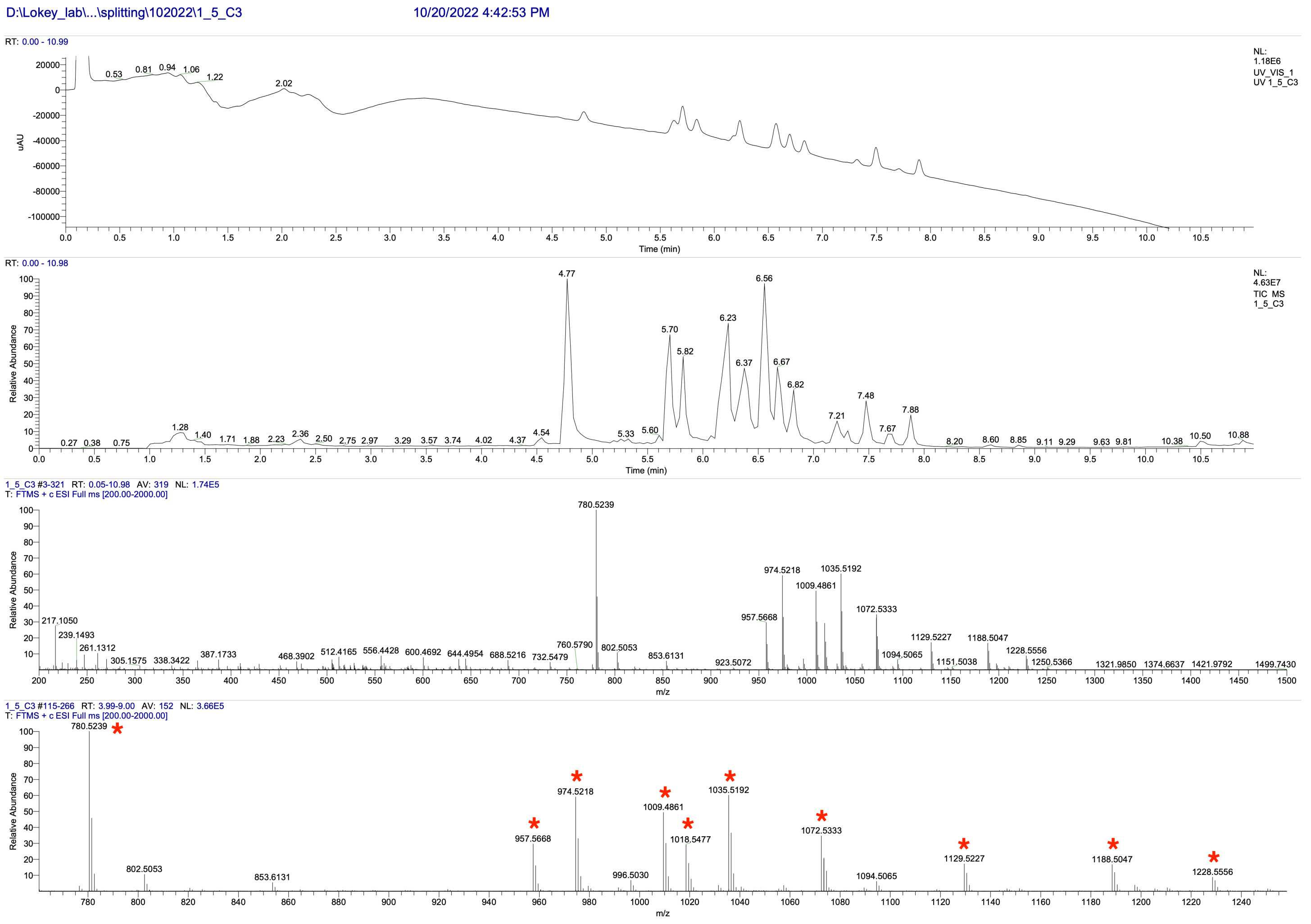

D:\Lokey_lab\...\splitting\102022\1_5_C3
10/20/2022 4:42:53 PM
RT: 0.00 - 10.99
NL: 1.18E6 UV_VIS_1 UV 1_5_C3
uAU
Time (min)
0.53 0.81 0.94 1.06 1.22 2.02
RT: 0.00 - 10.98
NL: 4.63E7 TIC MS 1_5_C3
Relative Abundance
4.77 5.70 5.82 6.23 6.37 6.56 6.67 6.82 7.21 7.48 7.67 7.88
0.27 0.38 0.75 1.28 1.40 1.71 1.88 2.23 2.36 2.50 2.75 2.97 3.29 3.57 3.74 4.02 4.37 4.54 5.33 5.60 8.20 8.60 8.85 9.11 9.29 9.63 9.81 10.38 10.50 10.88
1_5_C3 #3-321 RT: 0.05-10.98 AV: 319 NL: 1.74E5
T: FTMS + c ESI Full ms [200.00-2000.00]
780.5239 974.5218 1035.5192 1009.4861 1072.5333 957.5668 1129.5227 1188.5047 1228.5556 1250.5366
217.1050 239.1493 261.1312 305.1575 338.3422 387.1733 468.3902 512.4165 556.4428 600.4692 644.4954 688.5216 732.5479 760.5790 802.5053 853.6131 923.5072 1094.5065 1151.5038 1321.9850 1374.6637 1421.9792 1499.7430
m/z
1_5_C3 #115-266 RT: 3.99-9.00 AV: 152 NL: 3.66E5
T: FTMS + c ESI Full ms [200.00-2000.00]
780.5239 802.5053 853.6131 957.5668 974.5218 996.5030 1009.4861 1018.5477 1035.5192 1072.5333 1094.5065 1129.5227 1188.5047 1228.5556
m/z

g

D:\Lokey_lab\...\091222\plate10_F16_02 9/13/2022 8:26:52 AM

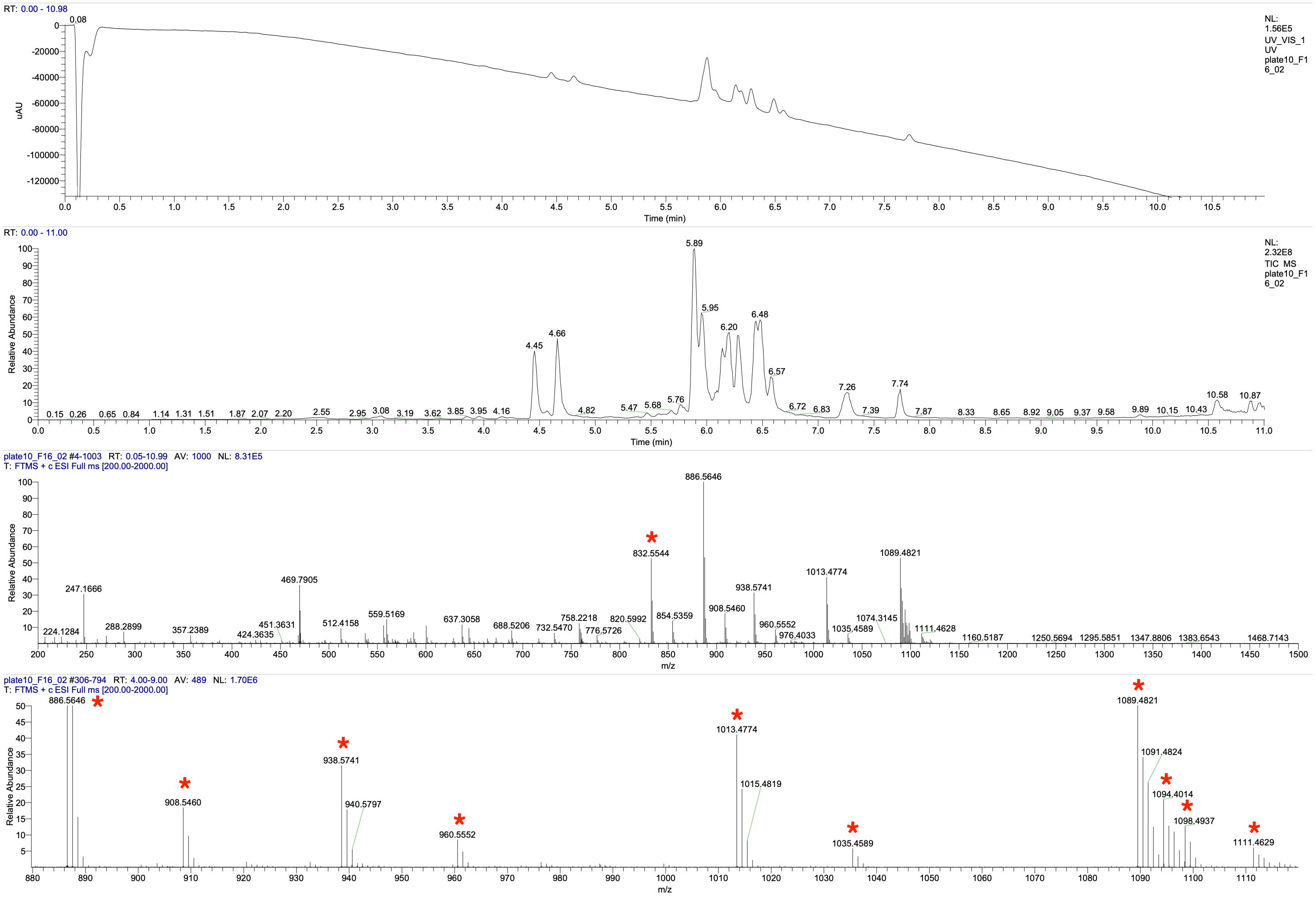

h

D:\Lokey_lab\...\091222\plate10_J3_02 9/13/2022 9:59:45 AM

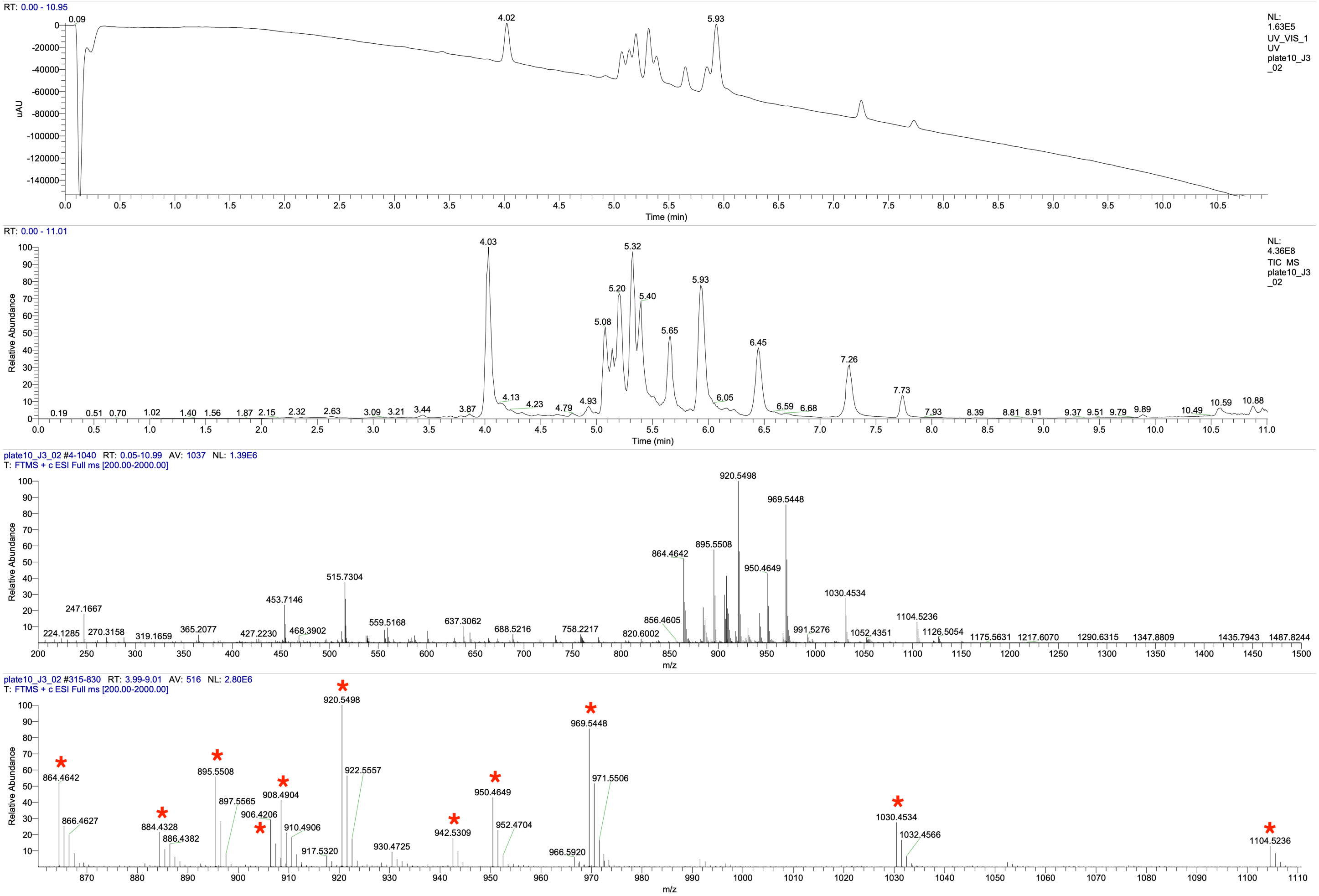

i

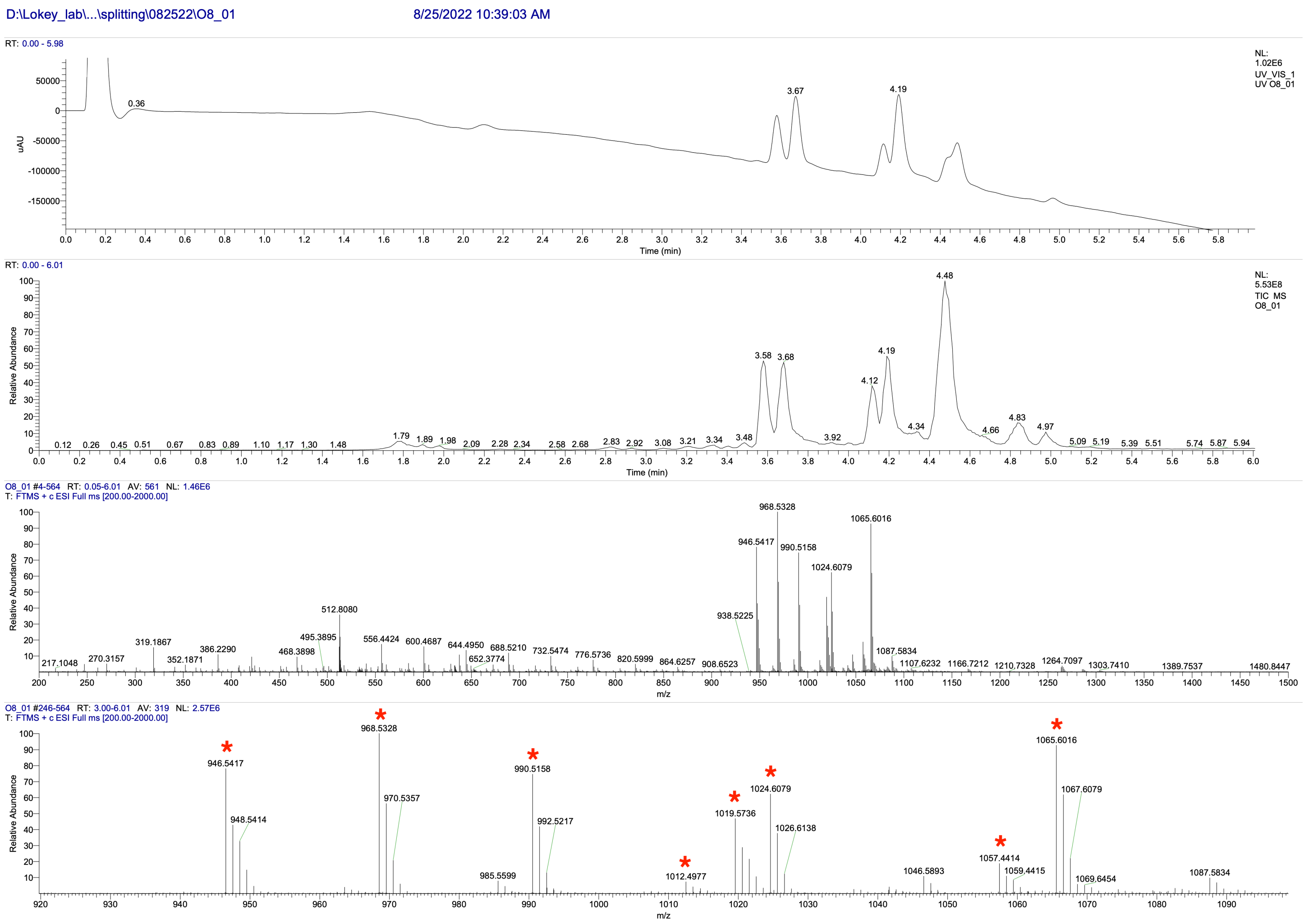
D:\Lokey_lab\...\splitting\082522\O8_01
8/25/2022 10:39:03 AM
RT: 0.00 - 5.98
NL: 1.02E6 UV_VIS_1 UV O8_01
uAU
Time (min)
RT: 0.00 - 6.01
NL: 5.53E8 TIC MS O8_01
Relative Abundance
Time (min)
O8_01 #4-564 RT: 0.05-6.01 AV: 561 NL: 1.46E6
T: FTMS + c ESI Full ms [200.00-2000.00]
m/z
O8_01 #246-564 RT: 3.00-6.01 AV: 319 NL: 2.57E6
T: FTMS + c ESI Full ms [200.00-2000.00]
m/z

j

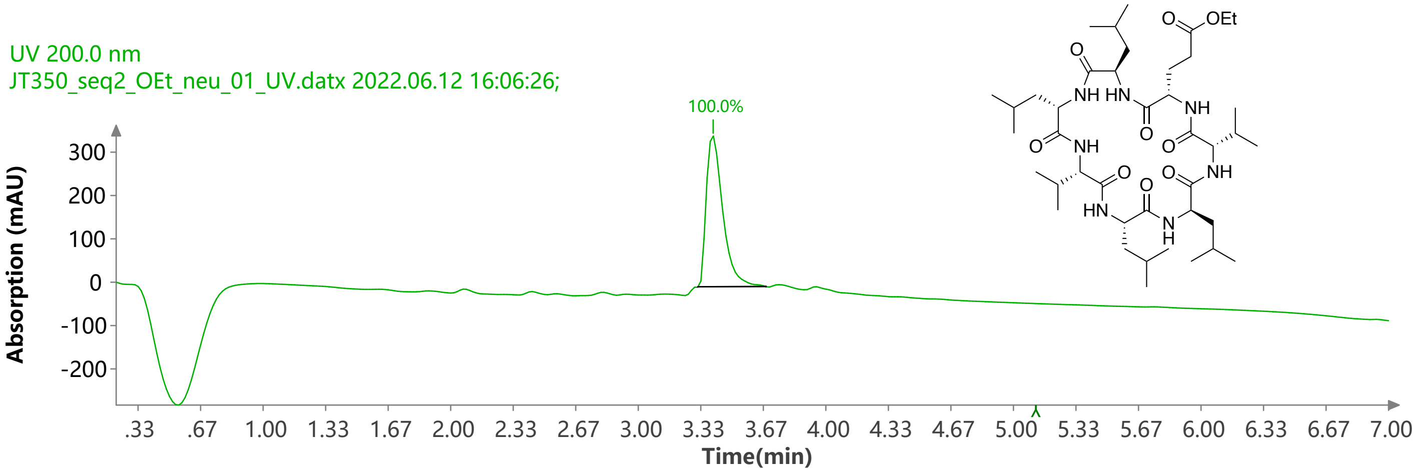

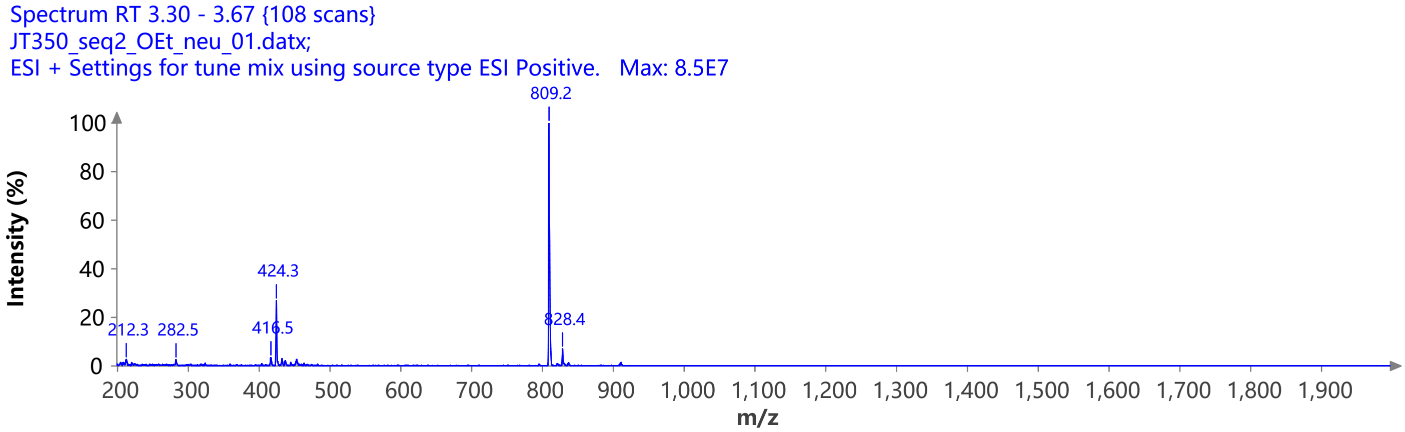

Figure S3. (a–c) Purity assessment of individual beads. Each bead from the library synthesis was cleaved in a HFIP/CH_2Cl_2 solution and analyzed by LC-MS and high-resolution mass spectrometry (HRMS). Possible sequences were determined by matching the observed high-resolution masses to the theoretical masses within the OBOC library. (d–i) LC-MS spectra of selected mixtures from the OBOC library. Asterisks indicate the exact masses of cyclic heptapeptides with a mass error below 5 ppm relative to the theoretical masses in the OBOC library. (j) A representative LC-MS chromatogram demonstrating the purity of a cyclic heptapeptide synthesized according to the OBOC synthesis protocol. The estimated yield is 23% based on a 0.03 mmol scale.

Hit Selections

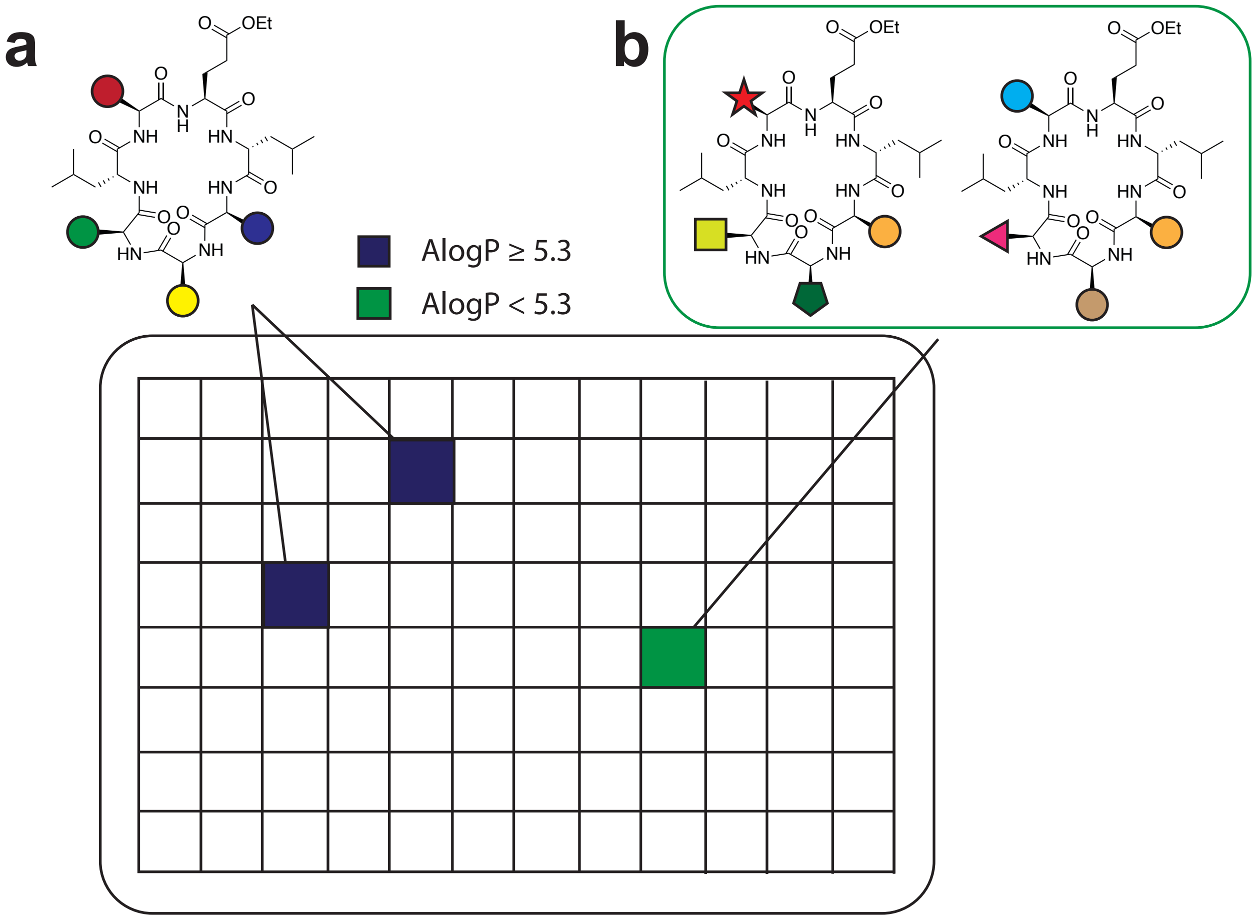

C **(morti01)**

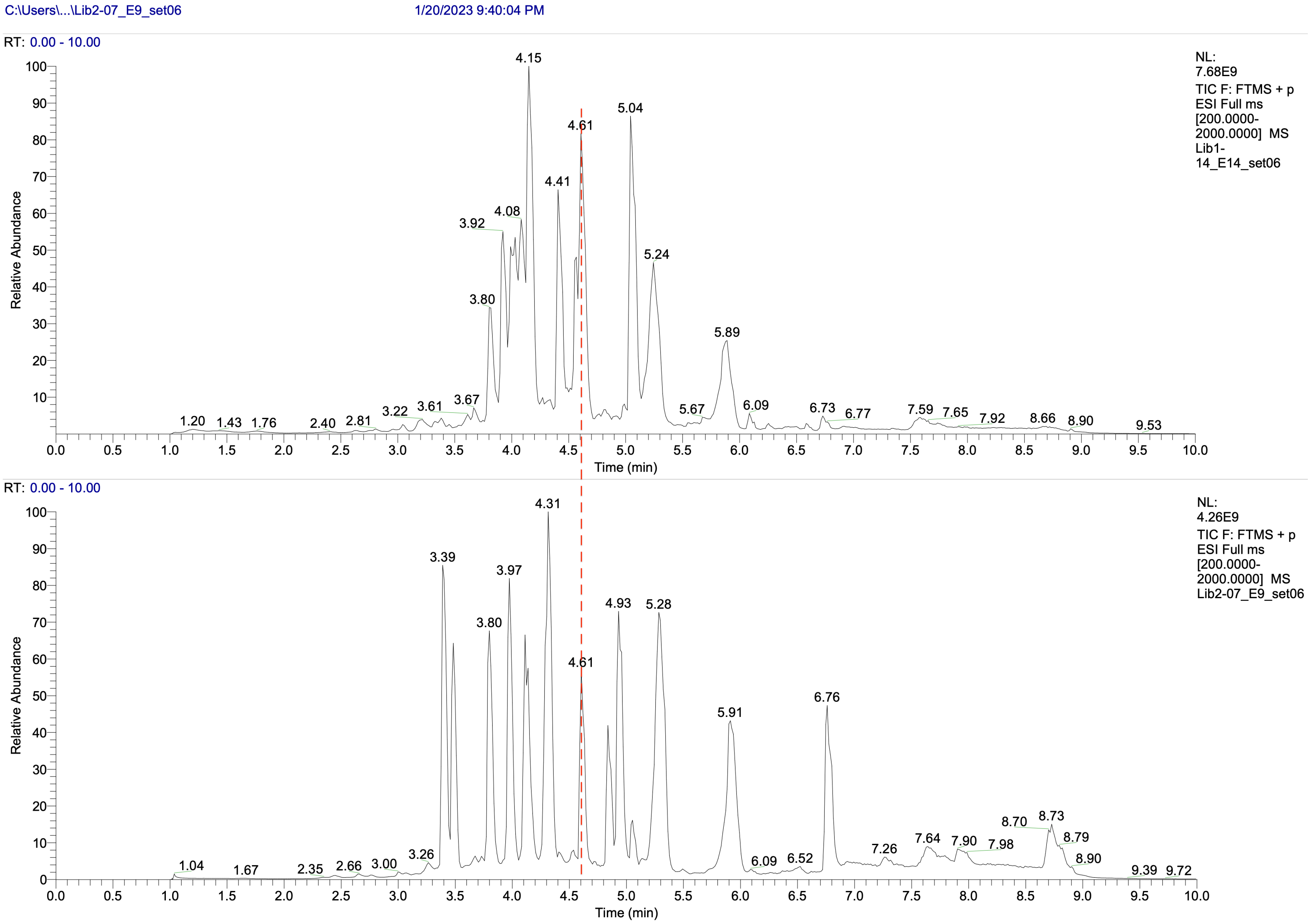
C:\Users\...\Lib2-07_E9_set06
1/20/2023 9:40:04 PM
RT: 0.00 - 10.00
NL: 7.68E9
TIC F: FTMS + p ESI Full ms [200.0000-2000.0000] MS Lib1-14_E14_set06
Relative Abundance
Time (min)
4.15
5.04
4.61
4.41
4.08
3.92
5.24
3.80
5.89
1.20
1.43
1.76
2.40
2.81
3.22
3.61
3.67
5.67
6.09
6.73
6.77
7.59
7.65
7.92
8.66
8.90
9.53
RT: 0.00 - 10.00
NL: 4.26E9
TIC F: FTMS + p ESI Full ms [200.0000-2000.0000] MS Lib2-07_E9_set06
Relative Abundance
Time (min)
4.31
3.39
3.97
3.80
4.93
5.28
4.61
5.91
6.76
1.04
1.67
2.35
2.66
3.00
3.26
6.09
6.52
7.26
7.64
7.90
7.98
8.70
8.73
8.79
8.90
9.39
9.72

d **(morti01)**

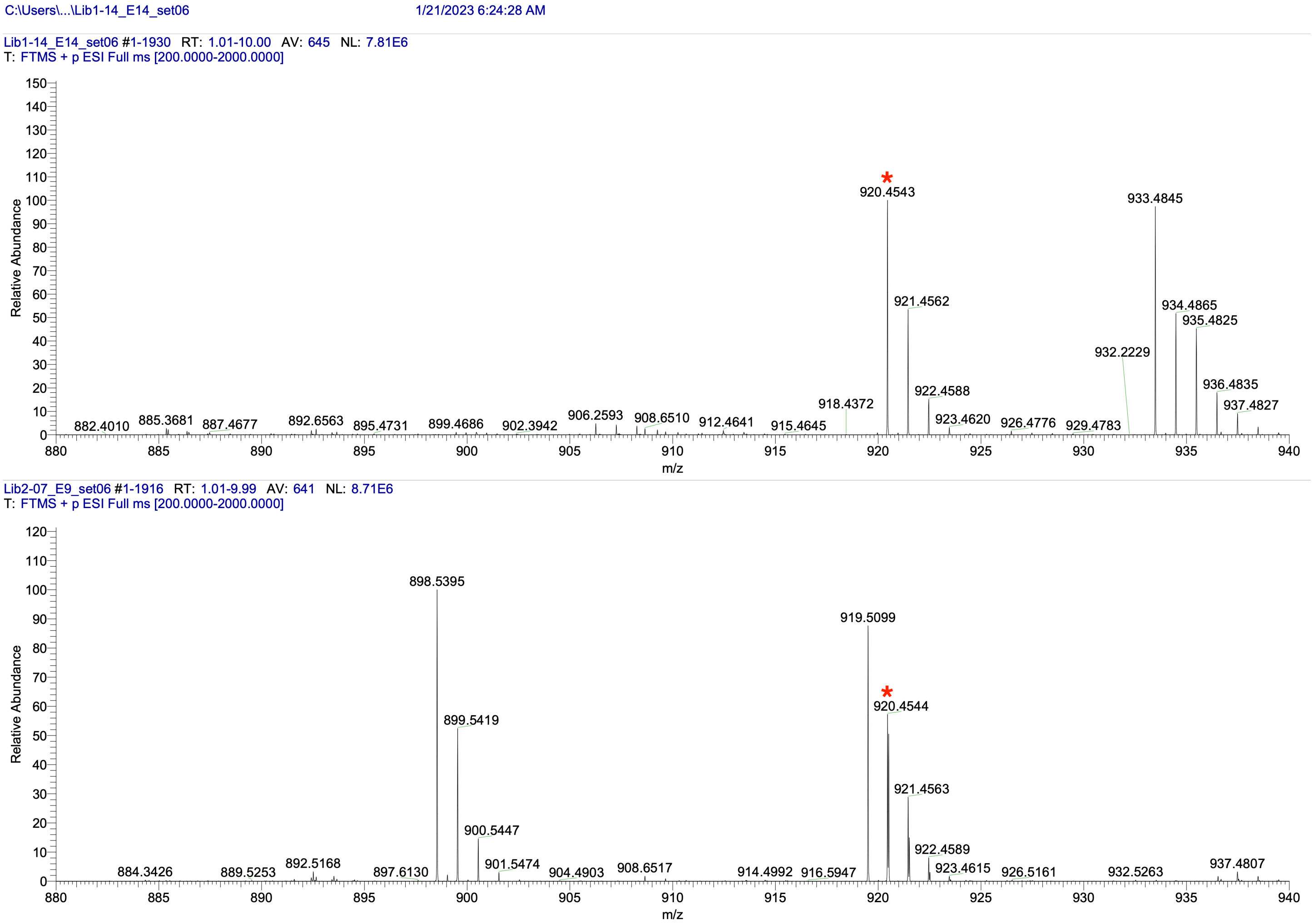

C:\Users\...\Lib1-14_E14_set06
1/21/2023 6:24:28 AM
Lib1-14_E14_set06 #1-1930 RT: 1.01-10.00 AV: 645 NL: 7.81E6
T: FTMS + p ESI Full ms [200.0000-2000.0000]
Relative Abundance
m/z
*
920.4543
933.4845
921.4562
934.4865
935.4825
932.2229
922.4588
936.4835
937.4827
918.4372
923.4620
926.4776
929.4783
915.4645
912.4641
908.6510
906.2593
902.3942
899.4686
895.4731
892.6563
887.4677
885.3681
882.4010
Lib2-07_E9_set06 #1-1916 RT: 1.01-9.99 AV: 641 NL: 8.71E6
T: FTMS + p ESI Full ms [200.0000-2000.0000]
898.5395
919.5099
920.4544
899.5419
921.4563
900.5447
922.4589
901.5474
923.4615
937.4807
884.3426
889.5253
892.5168
897.6130
904.4903
908.6517
914.4992
916.5947
926.5161
932.5263

e (morti01)

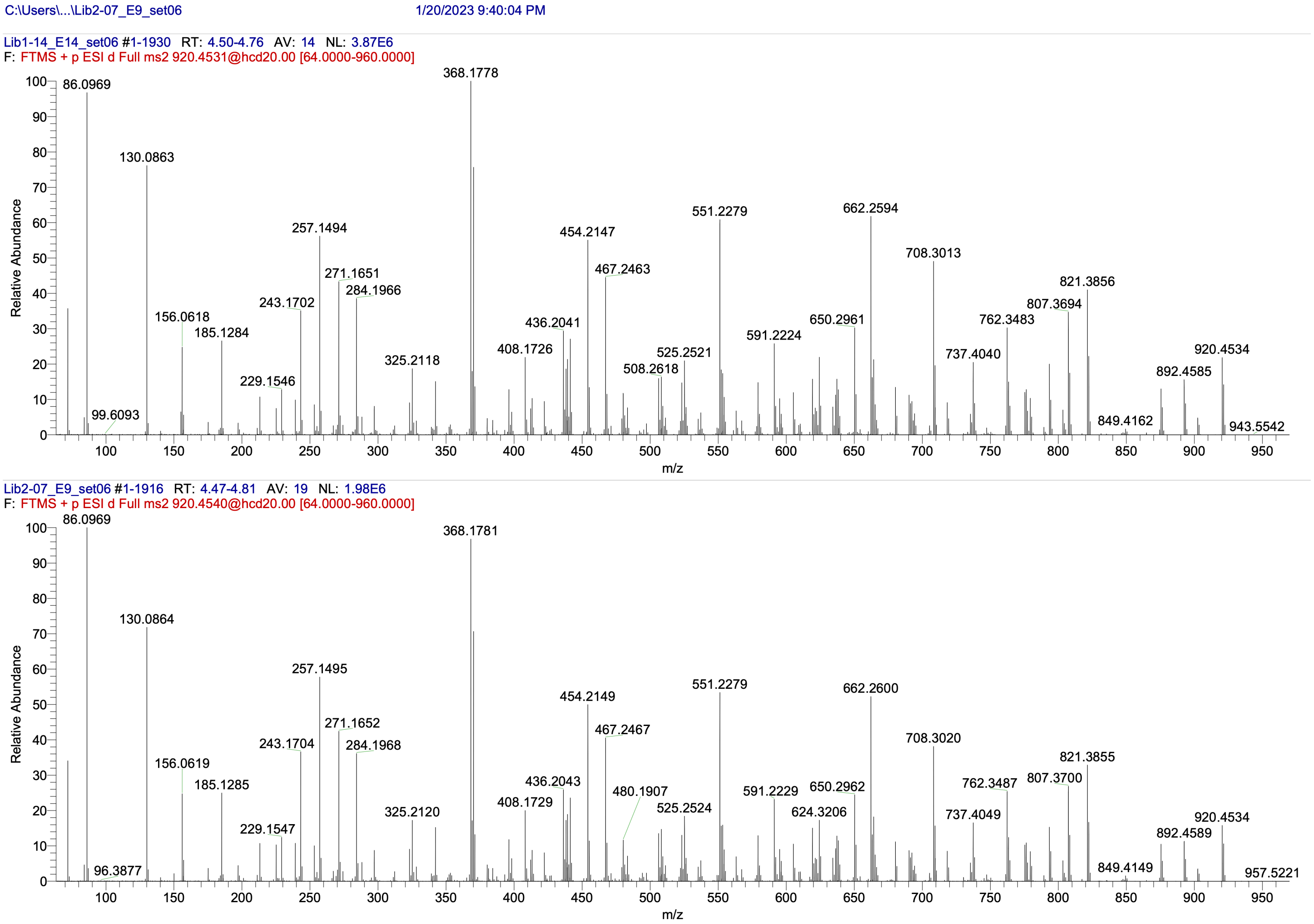
C:\Users\...\Lib2-07_E9_set06
1/20/2023 9:40:04 PM
Lib1-14_E14_set06 #1-1930 RT: 4.50-4.76 AV: 14 NL: 3.87E6
F: FTMS + p ESI d Full ms2 920.4531@hcd20.00 [64.0000-960.0000]
Relative Abundance
m/z
86.0969
368.1778
130.0863
257.1494
454.2147
551.2279
662.2594
708.3013
271.1651
284.1966
467.2463
821.3856
243.1702
807.3694
156.0618
436.2041
762.3483
650.2961
185.1284
591.2224
408.1726
920.4534
325.2118
525.2521
737.4040
508.2618
892.4585
229.1546
99.6093
849.4162
943.5542
Lib2-07_E9_set06 #1-1916 RT: 4.47-4.81 AV: 19 NL: 1.98E6
F: FTMS + p ESI d Full ms2 920.4540@hcd20.00 [64.0000-960.0000]
86.0969
368.1781
130.0864
257.1495
551.2279
662.2600
454.2149
271.1652
467.2467
243.1704
284.1968
708.3020
156.0619
821.3855
185.1285
436.2043
762.3487
807.3700
480.1907
591.2229
650.2962
325.2120
408.1729
624.3206
525.2524
737.4049
920.4534
229.1547
892.4589
96.3877
849.4149
957.5221

f (morti02)

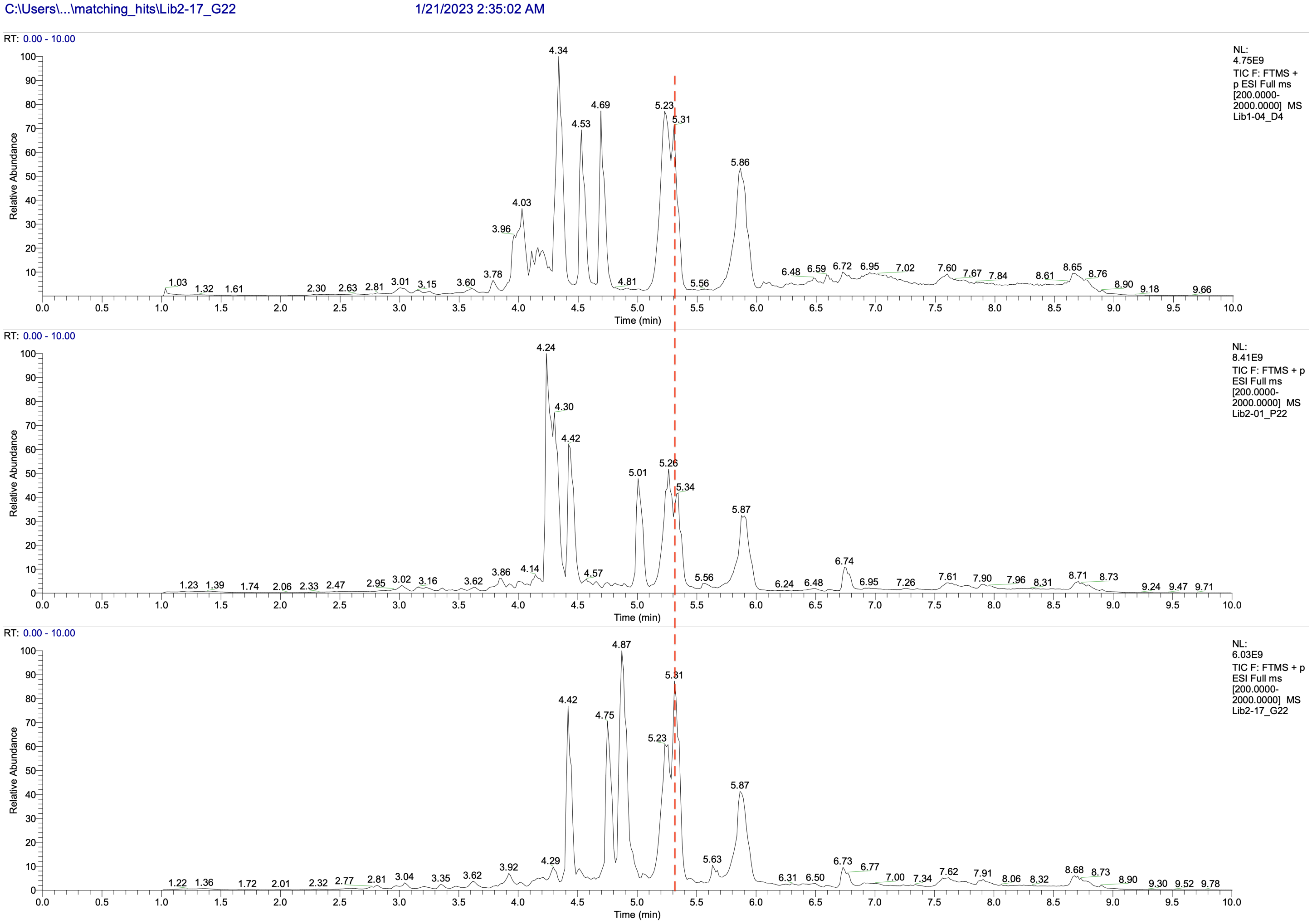
C:\Users\...\matching_hits\Lib2-17_G22
1/21/2023 2:35:02 AM
RT: 0.00 - 10.00
NL: 4.75E9
TIC F: FTMS + p ESI Full ms [200.0000-2000.0000] MS Lib1-04_D4
RT: 0.00 - 10.00
NL: 8.41E9
TIC F: FTMS + p ESI Full ms [200.0000-2000.0000] MS Lib2-01_P22
RT: 0.00 - 10.00
NL: 6.03E9
TIC F: FTMS + p ESI Full ms [200.0000-2000.0000] MS Lib2-17_G22
Relative Abundance
Time (min)

g **(morti02)**

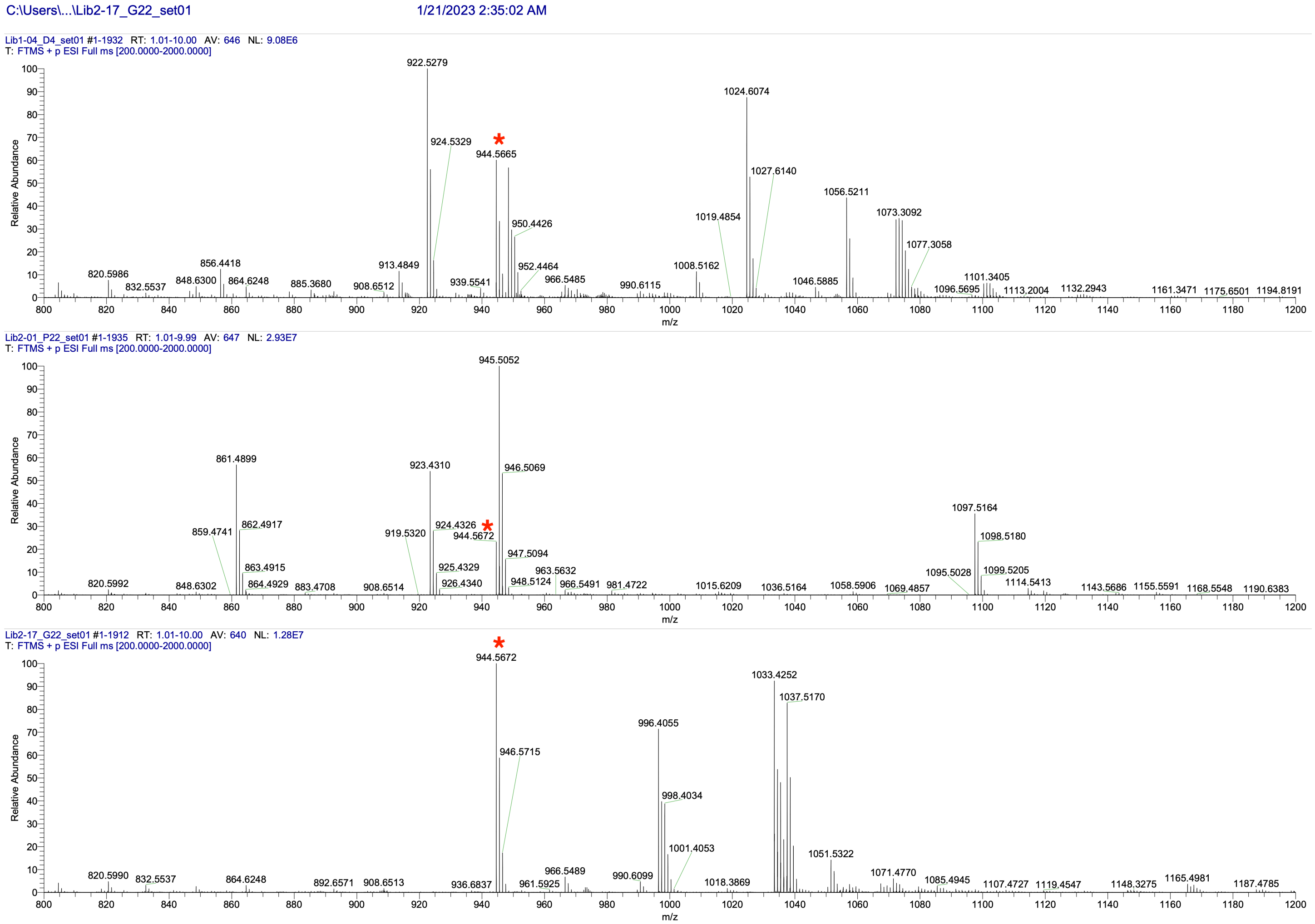
C:\Users\...\Lib2-17_G22_set01
1/21/2023 2:35:02 AM
Lib1-04_D4_set01 #1-1932 RT: 1.01-10.00 AV: 646 NL: 9.08E6
T: FTMS + p ESI Full ms [200.0000-2000.0000]
Lib2-01_P22_set01 #1-1935 RT: 1.01-9.99 AV: 647 NL: 2.93E7
T: FTMS + p ESI Full ms [200.0000-2000.0000]
Lib2-17_G22_set01 #1-1912 RT: 1.01-10.00 AV: 640 NL: 1.28E7
T: FTMS + p ESI Full ms [200.0000-2000.0000]
Relative Abundance
m/z
922.5279
1024.6074
944.5665
945.5052
944.5672
1033.4252
1037.5170
996.4055

h **(morti02)**

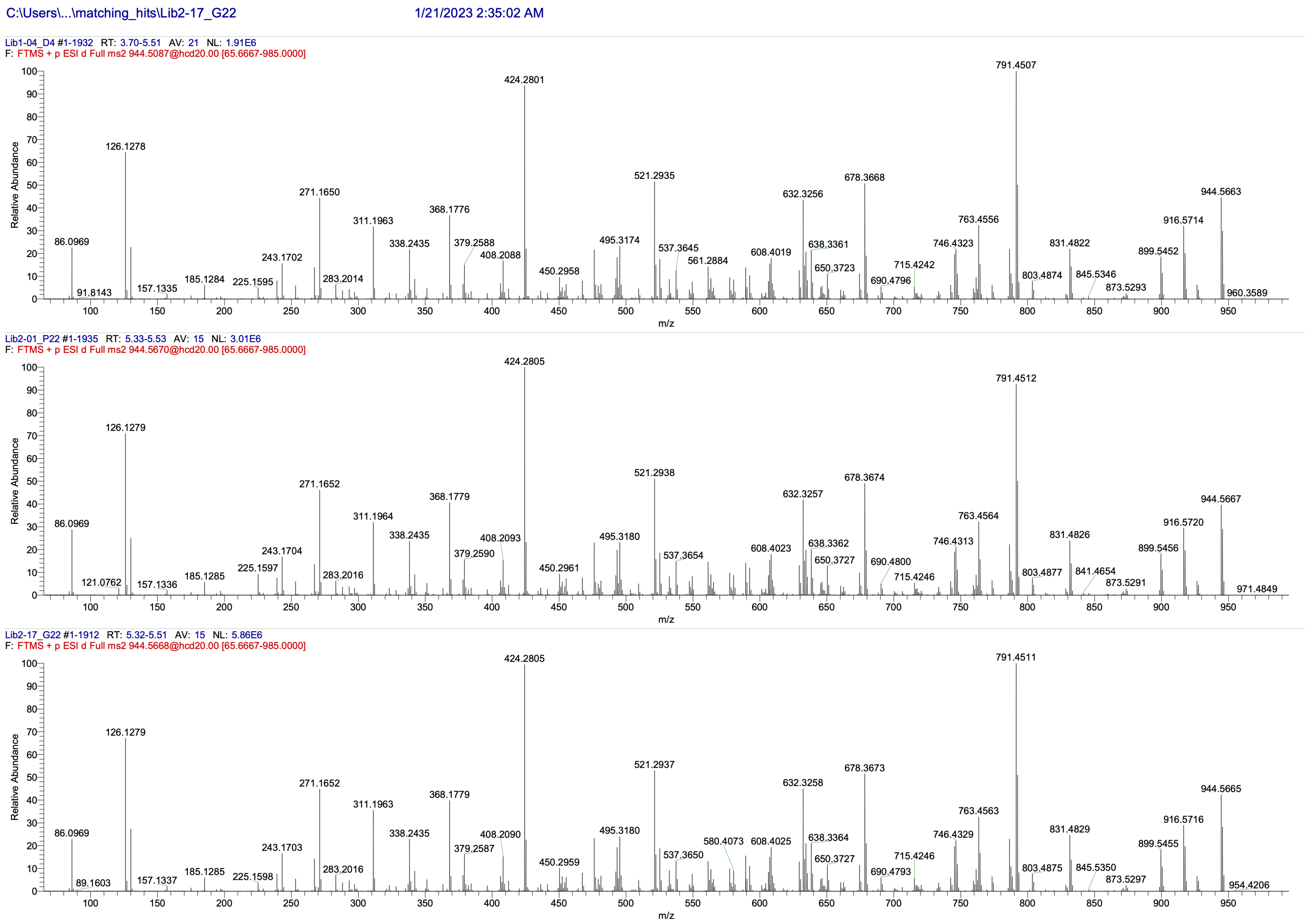
C:\Users\...\matching_hits\Lib2-17_G22
1/21/2023 2:35:02 AM
Lib1-04_D4 #1-1932 RT: 3.70-5.51 AV: 21 NL: 1.91E6
F: FTMS + p ESI d Full ms2 944.5087@hcd20.00 [65.6667-985.0000]
Lib2-01_P22 #1-1935 RT: 5.33-5.53 AV: 15 NL: 3.01E6
F: FTMS + p ESI d Full ms2 944.5670@hcd20.00 [65.6667-985.0000]
Lib2-17_G22 #1-1912 RT: 5.32-5.51 AV: 15 NL: 5.86E6
F: FTMS + p ESI d Full ms2 944.5668@hcd20.00 [65.6667-985.0000]
Relative Abundance
m/z

i (morti07)

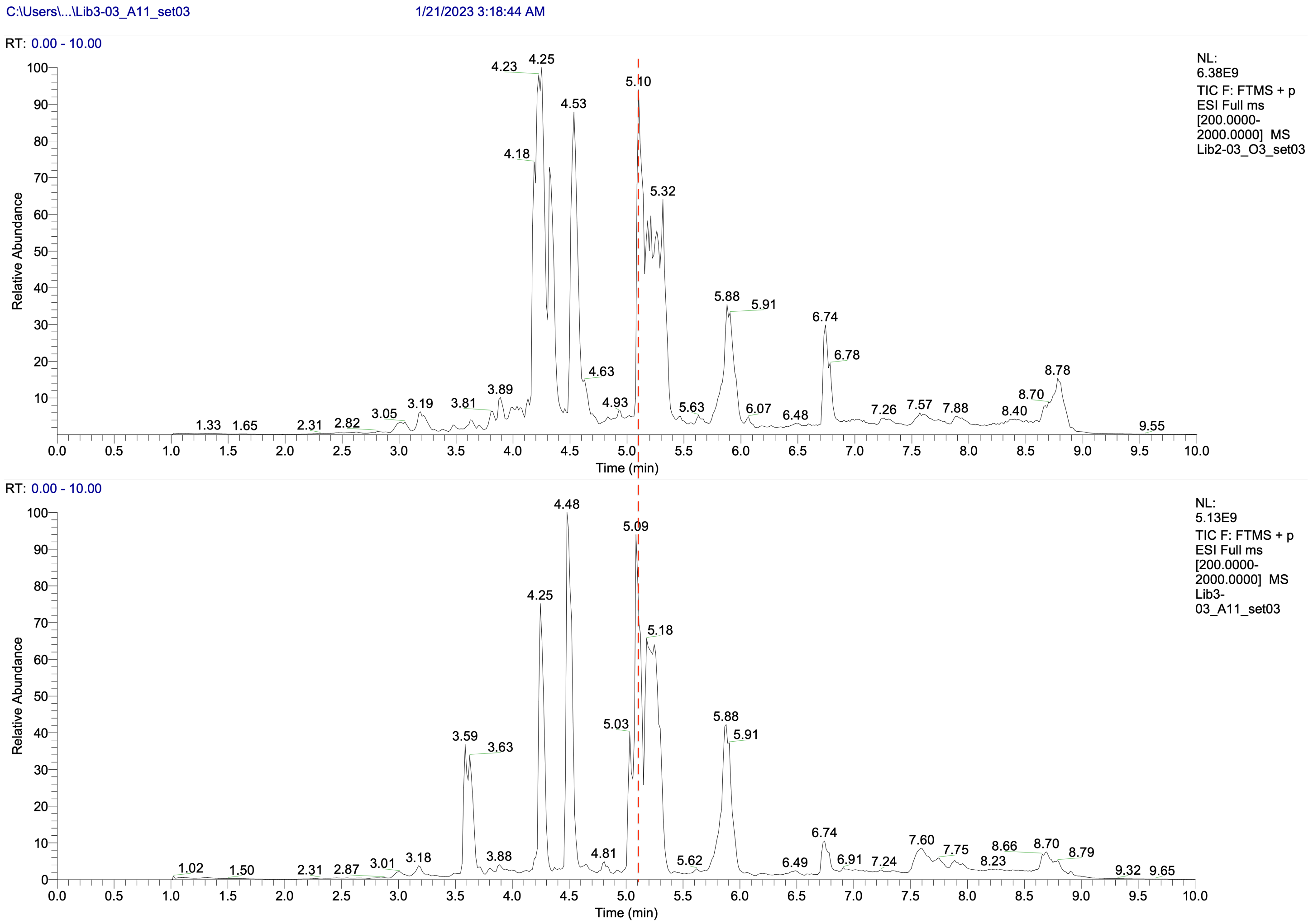

C:\Users\...\Lib3-03_A11_set03
1/21/2023 3:18:44 AM
RT: 0.00 - 10.00
NL: 6.38E9
TIC F: FTMS + p ESI Full ms [200.0000-2000.0000] MS Lib2-03_O3_set03
Relative Abundance
Time (min)
RT: 0.00 - 10.00
NL: 5.13E9
TIC F: FTMS + p ESI Full ms [200.0000-2000.0000] MS Lib3-03_A11_set03
Relative Abundance
Time (min)

j (morti07)

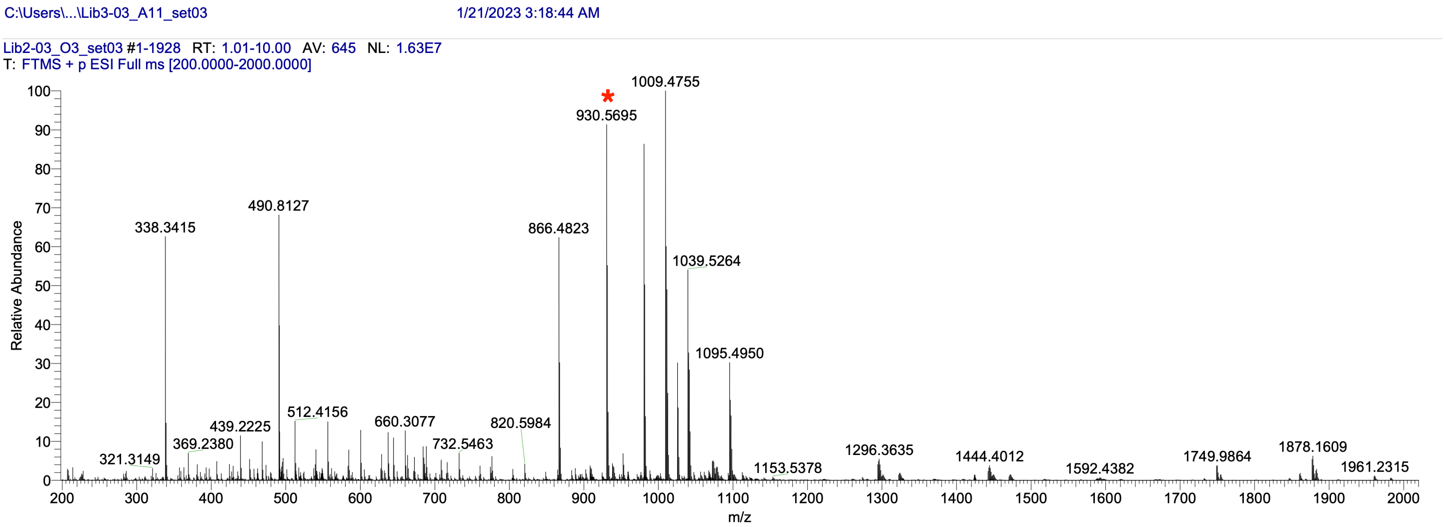
C:\Users\...\Lib3-03_A11_set03
1/21/2023 3:18:44 AM
Lib2-03_O3_set03 #1-1928 RT: 1.01-10.00 AV: 645 NL: 1.63E7
T: FTMS + p ESI Full ms [200.0000-2000.0000]
Relative Abundance
m/z
*
1009.4755
930.5695
490.8127
338.3415
866.4823
1039.5264
1095.4950
512.4156
439.2225
660.3077
820.5984
732.5463
369.2380
321.3149
1153.5378
1296.3635
1444.4012
1592.4382
1749.9864
1878.1609
1961.2315

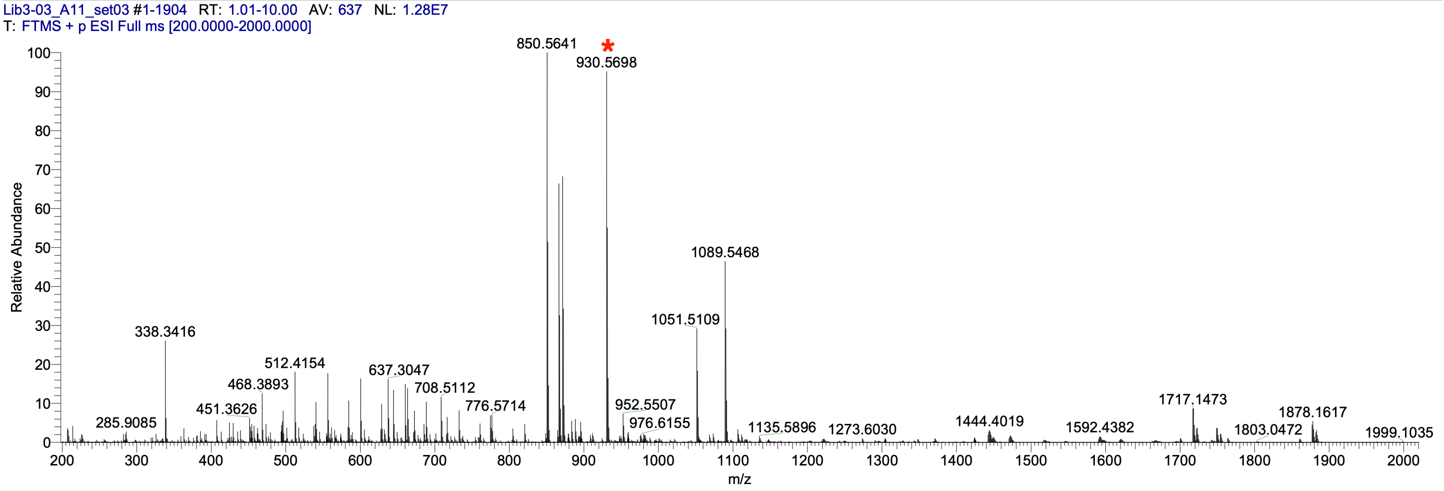
Lib3-03_A11_set03 #1-1904 RT: 1.01-10.00 AV: 637 NL: 1.28E7
T: FTMS + p ESI Full ms [200.0000-2000.0000]
Relative Abundance
m/z
*
850.5641
930.5698
1089.5468
1051.5109
338.3416
512.4154
637.3047
468.3893
708.5112
451.3626
776.5714
952.5507
976.6155
285.9085
1135.5896
1273.6030
1444.4019
1592.4382
1717.1473
1878.1617
1803.0472
1999.1035

k (morti07)

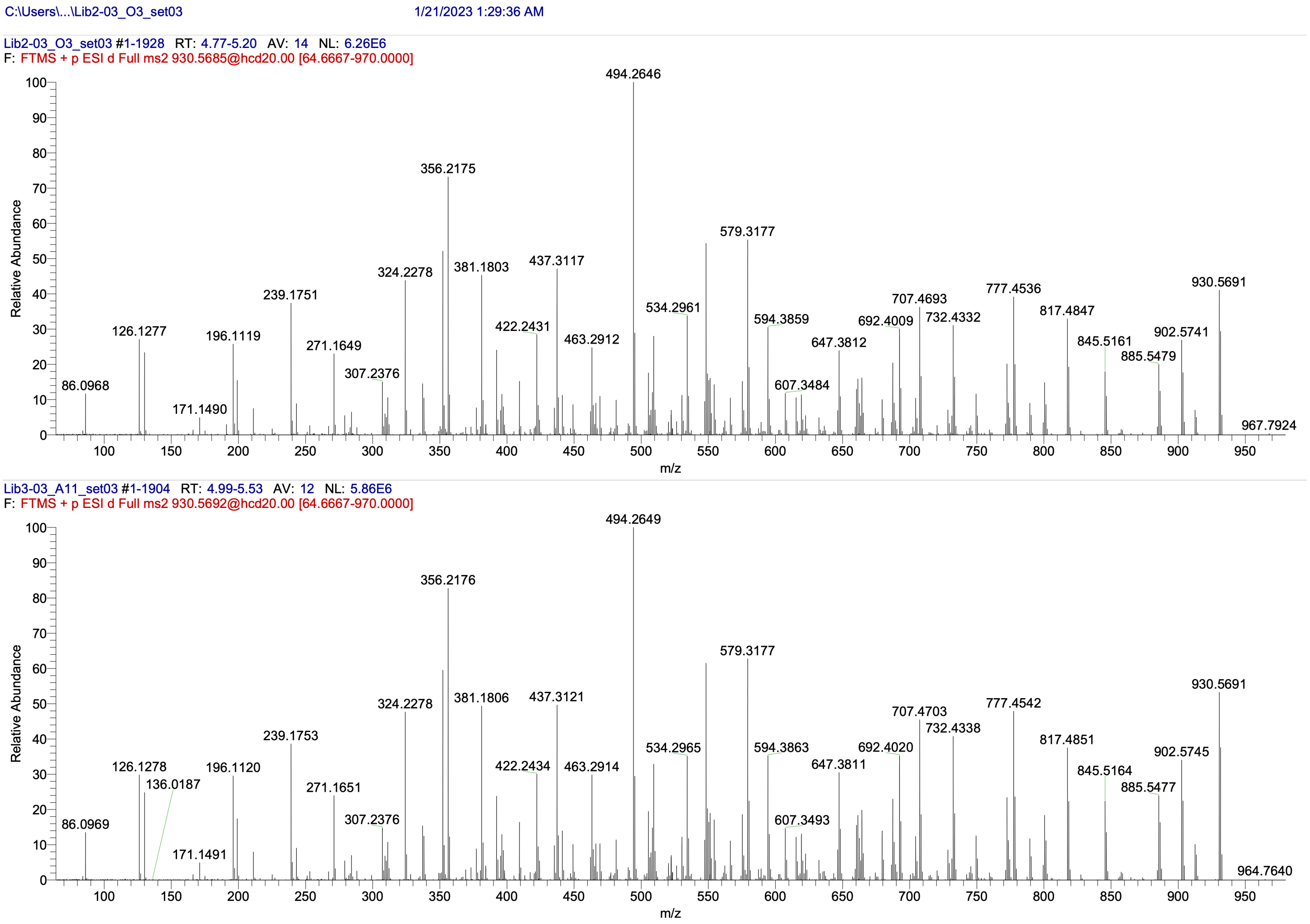

Figure S4. An example of hit selections. Two scenarios to select compounds for further studies: (a) identical compounds present in two or more active mixtures, or (b) all compounds in an active mixture have AlogP values less than 5.3. (c-k) Examples of hit deconvolution (**morti01**, **morti02**, and **morti07**) identified by matching LC retention times (indicated by red dashed line) and MS/MS fragmentation fingerprints (derived from precursor masses indicated by asterisks) across multiple active mixtures.

Inhibition of MDH Activity by 7-A^6 Cyclic Heptapeptide

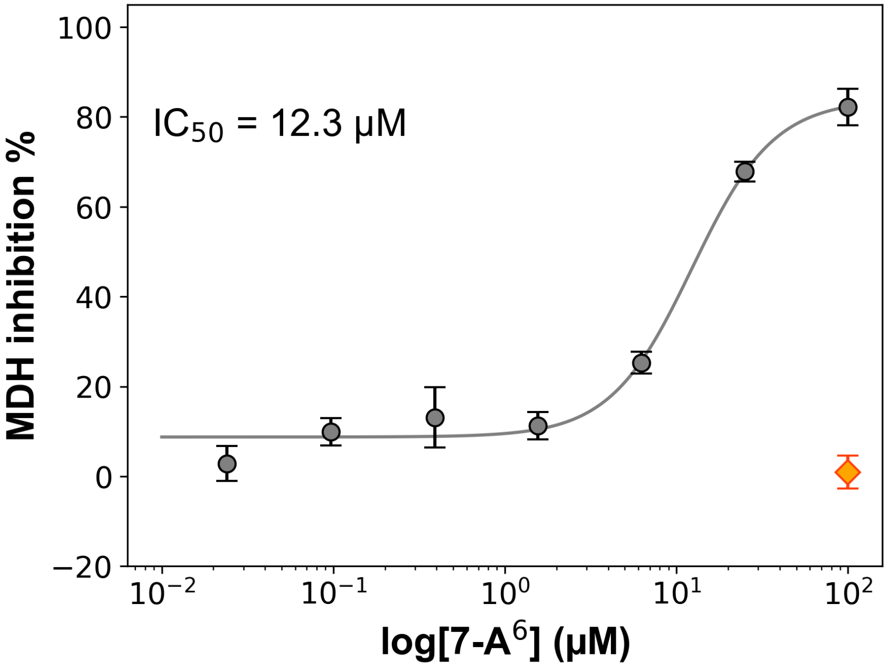

Figure S5. Percent inhibition of MDH activity by the **7-A^6** cyclic heptapeptide. Concentration-response curve of MDH inhibition is reported in gray circles. Percent inhibition after preincubation with Triton X-100 is shown in an orange diamond.

Size Distribution by Dynamic Light Scattering

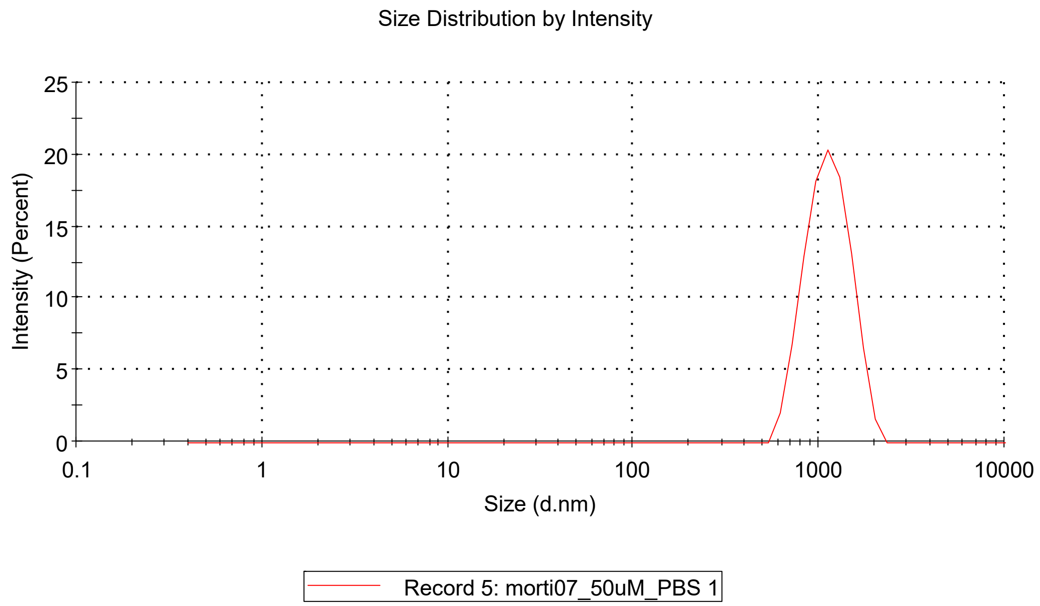

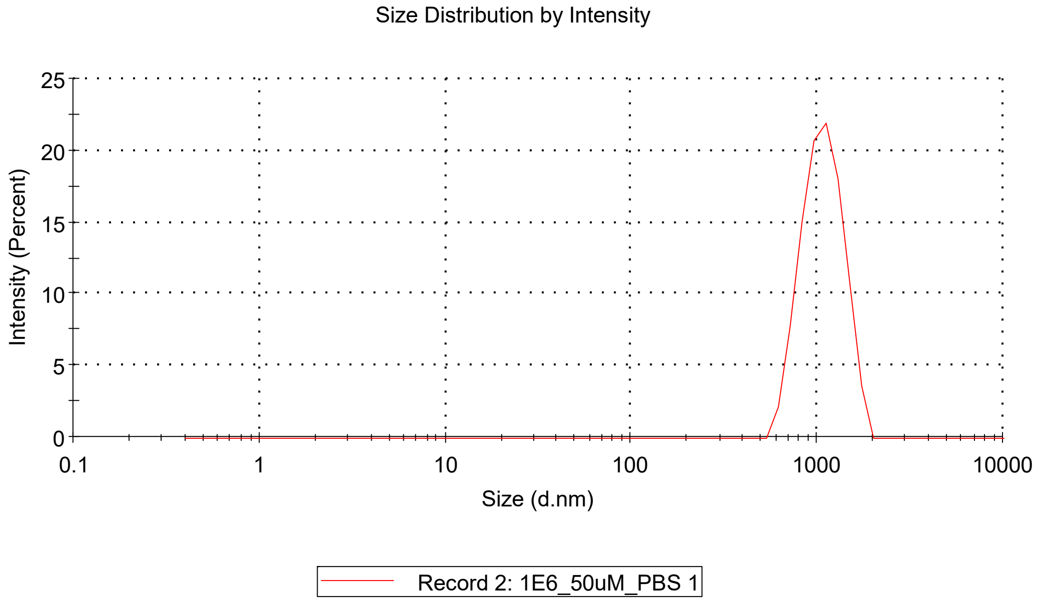

Figure S6. Size distribution of **morti07** (top) and **1E6** (bottom) by dynamic light scattering.

Hydrogen Bond Network of Mortiamide B Enantiomer in the Crystal Lattice

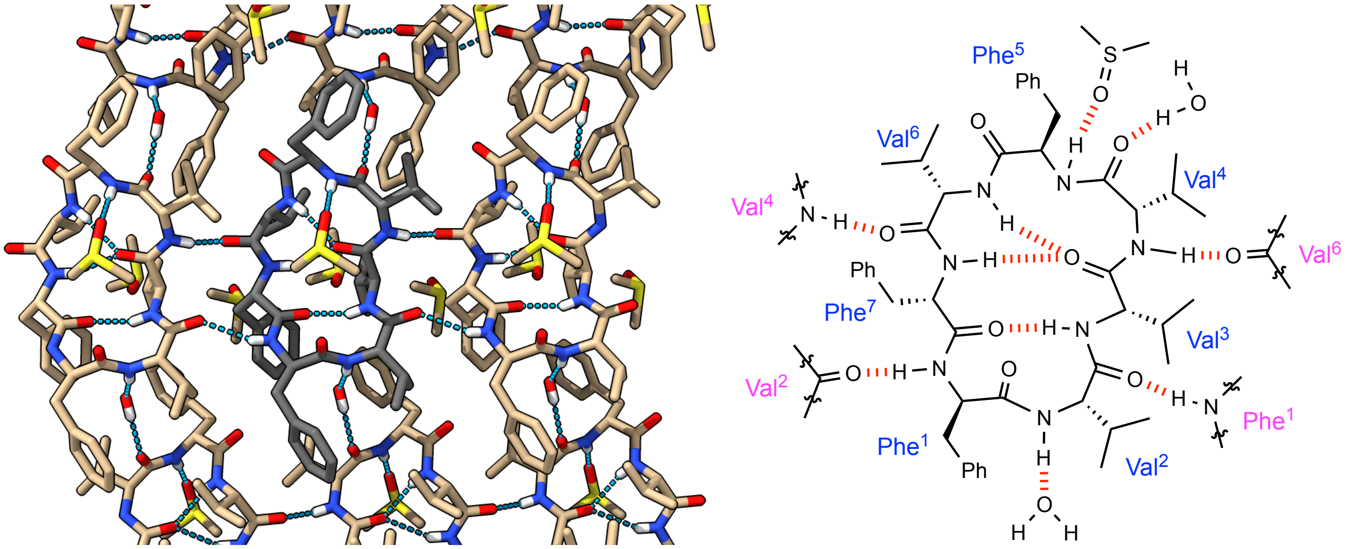

Figure S7. Crystal structure and hydrogen bond network of mortiamide B enantiomer.

Comparison of Backbone Conformations of Cyclic Heptapeptides with Diverse Side Chains

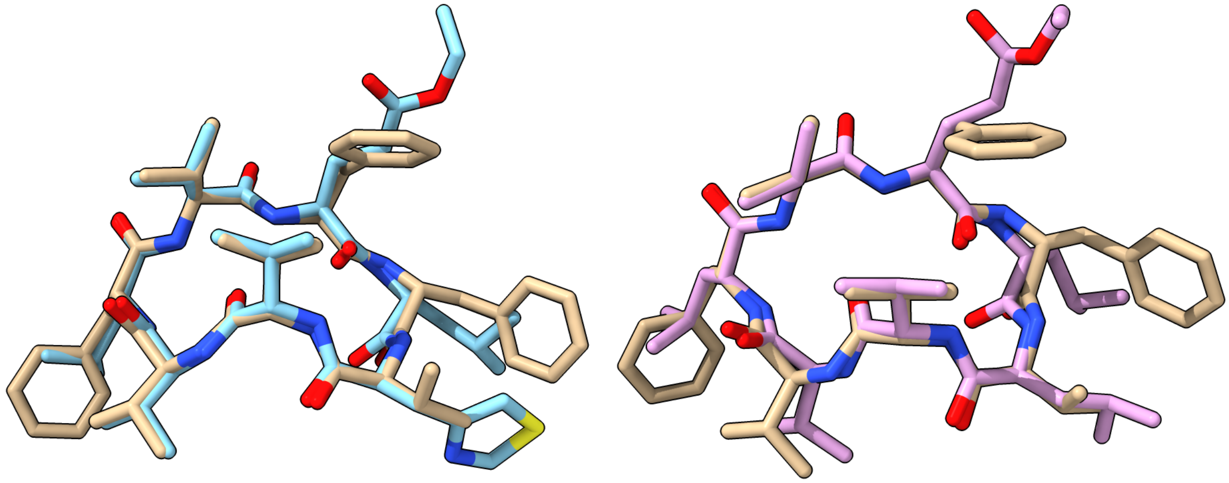

Figure S8. Overlays of the **morti15** (blue) and **morti16** (magenta) on the mortiamide B enantiomer (gold) crystal structures. The backbone RMSDs of these pairs are 0.24 Å and 0.36 Å, respectively.

Backbone Hydrogen Bond Pattern of Mortiamide A

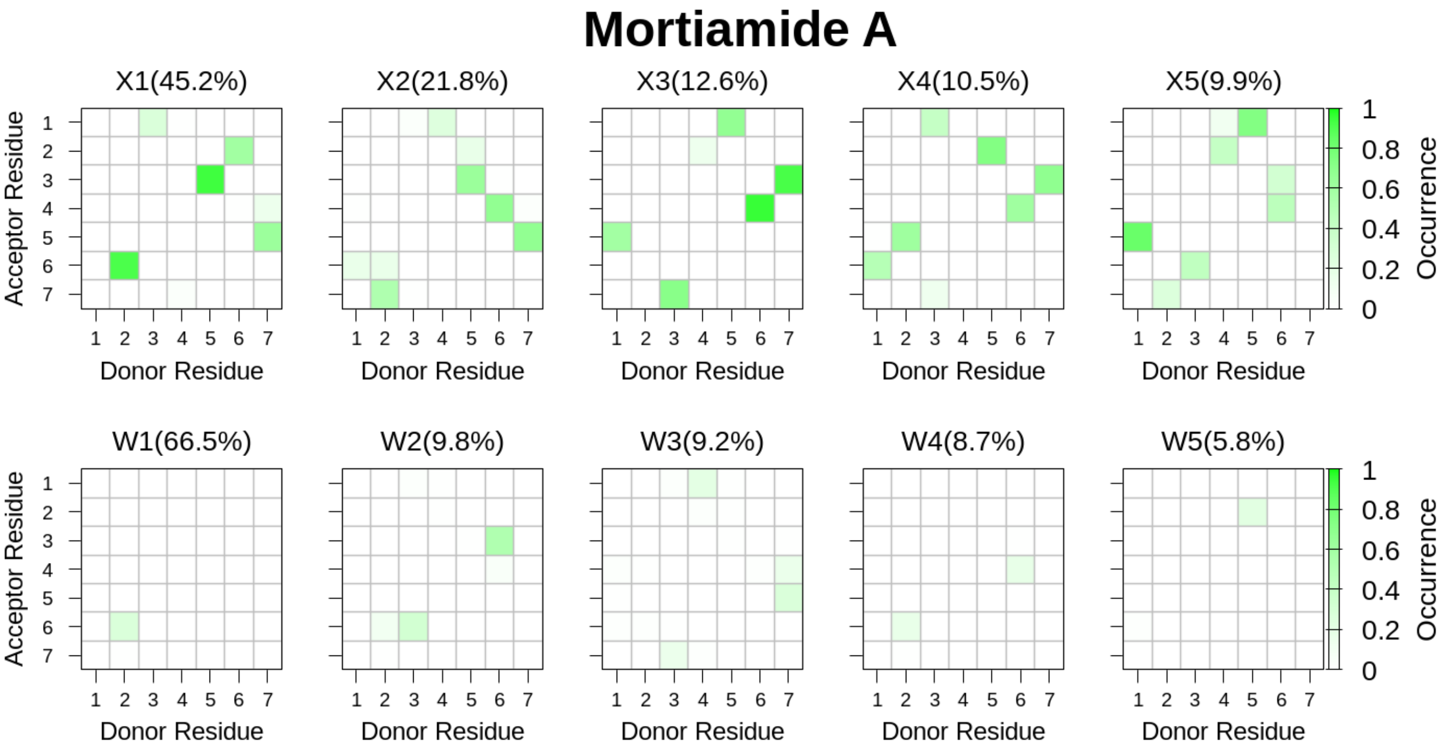

Figure S9. Backbone hydrogen bond pattern of mortiamide A. Top and bottom rows represent the conformational ensemble in cyclohexane and water, respectively.

General Procedure of X-ray Structure Determination

Crystals of mortiamide A, enantiomer of mortiamide B, **morti15**, and **morti16** were obtained by vapor diffusion of water into DMSO solutions of the compounds. In each case, a prism was microscopically selected under crossed polarizers, mounted on a MiTeGen polyimide loop, and cooled to 150 K on a Rigaku Synergy-S X-ray diffractometer. Diffraction of Cu Kα radiation from a PhotonJet-S microfocus source was detected using a HyPix-6000HE hybrid photon counting detector. Bijvoet pairs were collected for absolute structure determination. Screening, indexing, data collection, and data processing were performed with CrysAlis^{Pro}. The structure was solved using SHELXT and refined using SHELXL following established strategies.[1-3] All non-H atoms were refined anisotropically. C-bound H atoms were placed at calculated positions and refined with a riding model. As indicated in the corresponding CIFs, for O–H and N–H groups, H atoms were either placed at calculated positions and similarly refined or placed at the locations of maxima in the difference Fourier synthesis and refined semi-freely using distance restraints. All H-atom isotropic displacement parameters were constrained to 1.5 × U_{eq} for methyl groups and 1.2 × U_{eq} for all others, where U_{eq} is the equivalent isotropic thermal parameter of the atom to which the H atom is bound. Anomalous dispersion was used to refine the absolute structure (Flack) parameter of each structure.

Table S1. Crystal data and refinement parameters

	mortiamide A·H_2O·DMSO	mortiamide B enantiomer·H_2O·2 DMSO	**morti15**·2H_2O·3 DMSO	**morti16**·2H_2O· DMSO
Empirical formula	$C_{46}H_{73}N_7O_9S$	$C_{51}H_{77}N_7O_{10}S_2$	$C_{46}H_{88}N_8O_{14}S_4$	$C_{42}H_{81}N_7O_{12}S$
Formula weight	900.17	1012.31	1105.48	908.19
Temperature (K)	150(2)	150(1)	150 (2)	150 (2)
Wavelength (Å)	1.54184	1.54184	1.54184	1.54184
Crystal system	orthorhombic	orthorhombic	orthorhombic	tetragonal
Space group	$P2_12_12_1$	$P2_12_12_1$	$P2_12_12_1$	$P4_3$
a (Å)	9.3329(1)	9.4102(2)	9.7451(1)	16.3551(2)
b (Å)	22.3443(2)	23.5369(6)	20.6186(3)	
c (Å)	24.2225(2)	24.7635(4)	30.1270(4)	20.3300(4)
Volume (Å^3)	5051.29(8)	5484.8(2)	6053.43(13)	5438.06(17)
Z	4	4	4	4
ρ_{calc} (Mg/m^3)	1.184	1.226	1.213	1.109
Crystal size (mm^3)	0.20 × 0.04 × 0.03	0.15 × 0.03 × 0.02	0.24 × 0.15 × 0.08	0.5 × 0.11 × 0.06
θ range (°)	5.38 to 136.484	5.18 to 136.474	5.868 to 136.496	5.404 to 136.398
Total reflections	66763	33280	35196	36438
Unique reflections	9253	9975	11025	9922
Parameters	608	660	711	702
Completeness (%)	100	99.9	99.9	100
R_{int}	0.0381	0.0598	0.0381	0.0551
R_1 (I > 2σ)	0.0280	0.0462	0.0637	0.0613
R_1 (all data)	0.0303	0.0596	0.0695	0.0641
wR_2 (I > 2σ)	0.0629	0.0913	0.1824	0.1646
wR_2 (all data)	0.0637	0.0959	0.1882	0.1668
Goodness of fit, *S*	1.063	1.027	1.049	1.046
Flack parameter	0.007(15)	0.01(3)	0.03(3)	0.08(5)

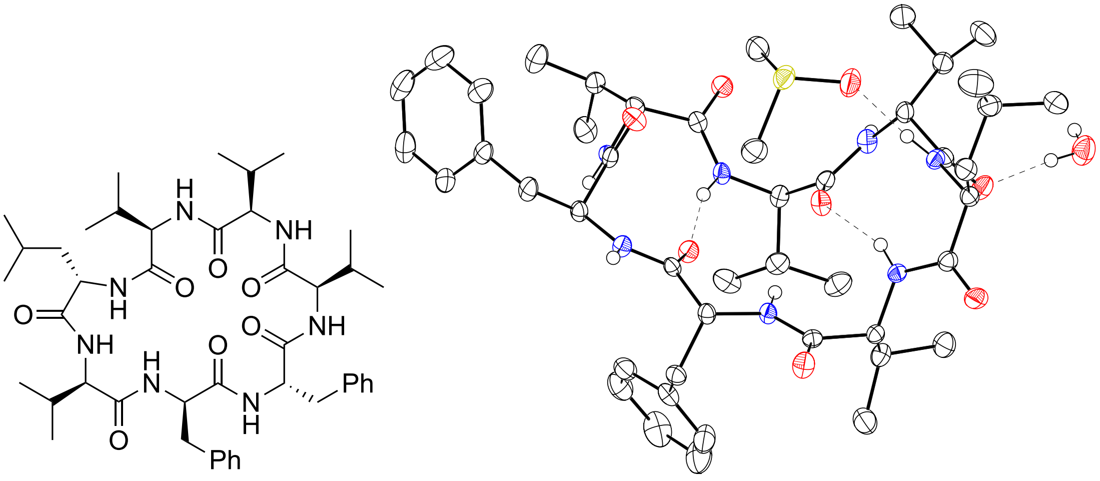

Figure S10. Thermal ellipsoid plot (50% probability level) of the asymmetric unit of the crystal structure of mortiamide A. C-bound H atoms omitted for clarity. Color code: O red, N blue, S yellow, C black, H white spheres of arbitrary radius. Hydrogen bonds are shown as dashed lines.

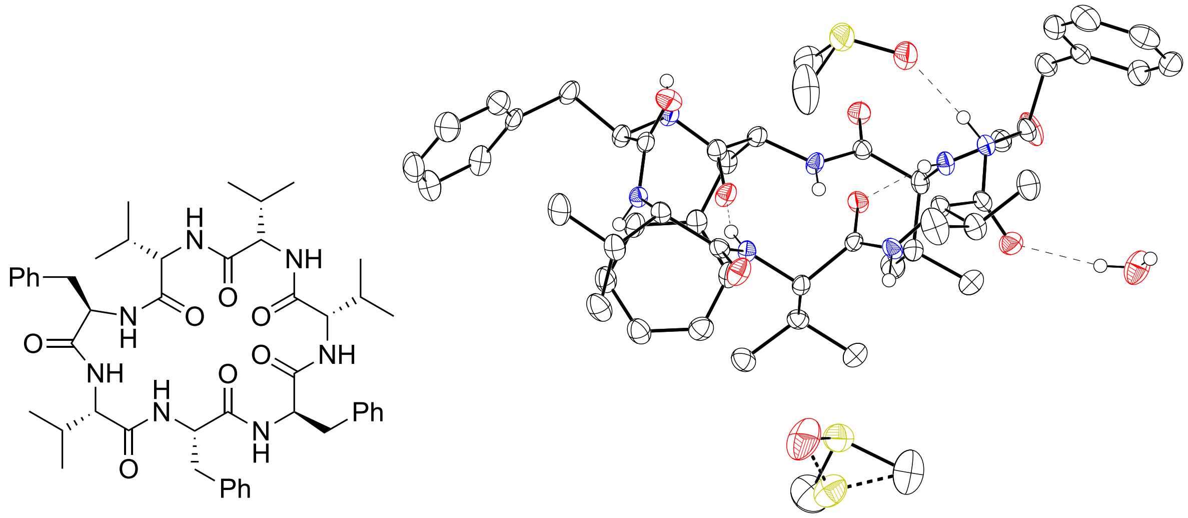

Figure S11. Thermal ellipsoid plot (50% probability level) of the asymmetric unit of the crystal structure of the mortiamide B enantiomer. C-bound H atoms omitted for clarity. Color code: O red, N blue, S yellow, C black, H white spheres of arbitrary radius. Hydrogen bonds are shown as dashed lines. One DMSO molecule is disordered across two positions; the minor component is shown with dashed bonds.

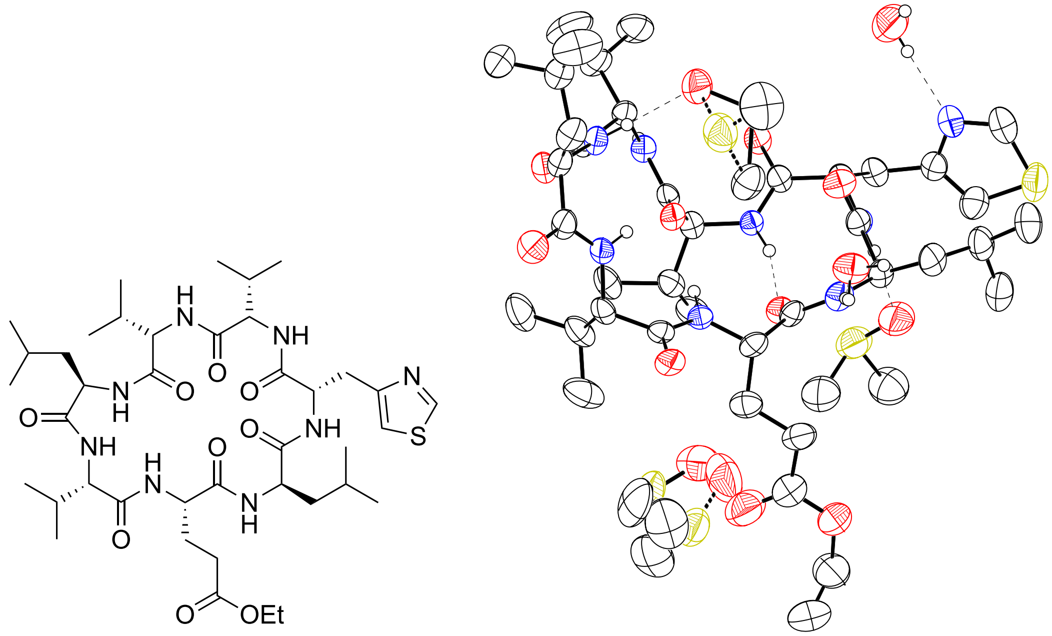

Figure S12. Thermal ellipsoid plot (50% probability level) of the asymmetric unit of the crystal structure of **morti15**. C-bound H atoms omitted for clarity. Color code: O red, N blue, S yellow, C black, H white spheres of arbitrary radius. Hydrogen bonds are shown as dashed lines. Two DMSO molecules were disordered across two positions; the minor components are shown with dashed bonds.

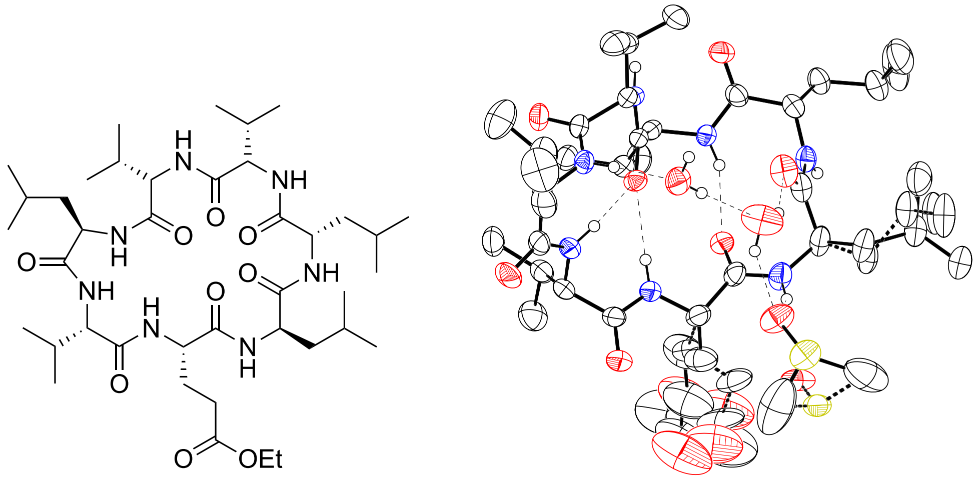

Figure S13. Thermal ellipsoid plot (50% probability level) of the asymmetric unit of the crystal structure of **morti16**. C-bound H atoms omitted for clarity. Color code: O red, N blue, S yellow, C black, H white spheres of arbitrary radius. Hydrogen bonds are shown as dashed lines. Two side chains are disordered across two positions; the minor components are shown with dashed bonds.

Supplemental Figures of TEM and CryoEM

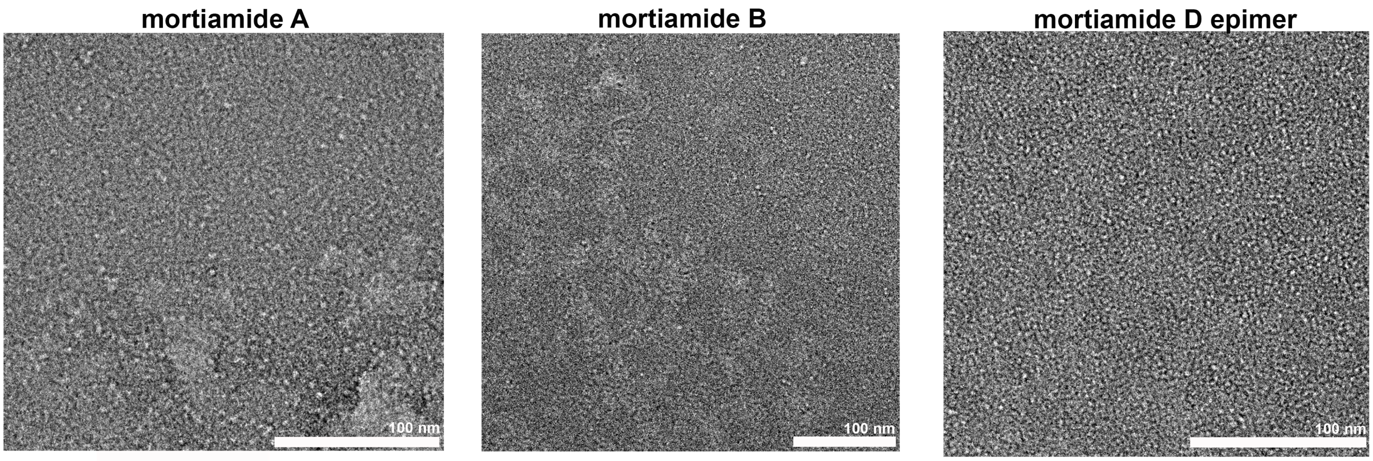

Figure S14. Negative stain Transmission Electron Microscopy (TEM) images of aggregates formed by inactive cyclic heptapeptides mortiamide A, mortiamide B, and mortiamide D epimer. Images are collected after incubating the sample quiescently for 24 h at 37 °C (100 μM, PBS buffer (pH 7.4)). Scale bars are as shown in the Figure.

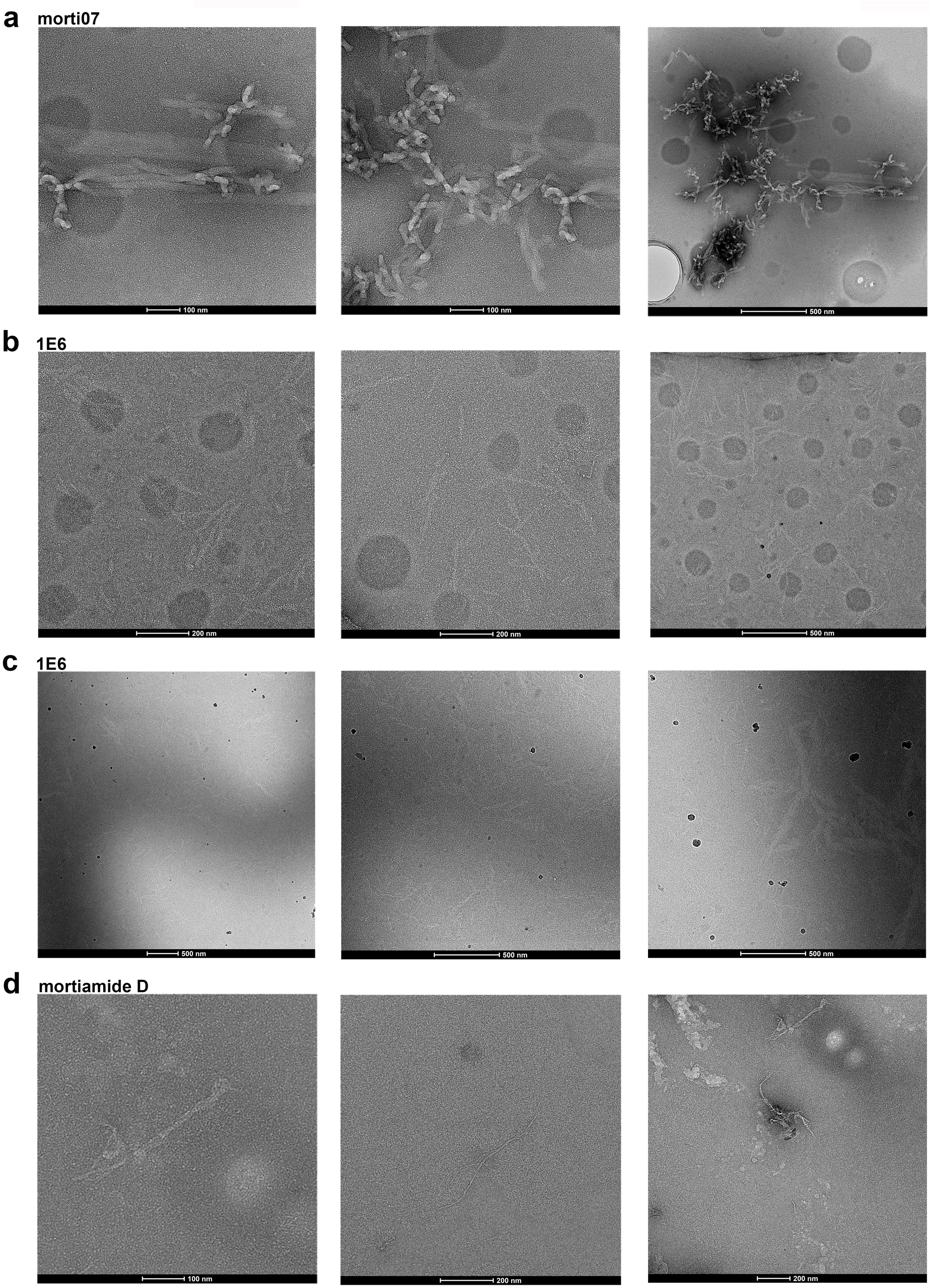

Figure S15. Negative stain Transmission Electron Microscopy (TEM) images of fibrils formed by active cyclic heptapeptides. (a) Fibril aggregates formed by **morti07** after incubating for 24 h. (b) Aggregates formed by **1E6** soon after dissolution. (c) Aggregates formed by **1E6** after incubating for 30

mins. (d) Aggregates formed by mortiamide D after 24 h. All are dissolved at 100 μM concentration in PBS buffer (pH 7.4) and incubated at 37 °C. Scale bars are as shown in the Figure.

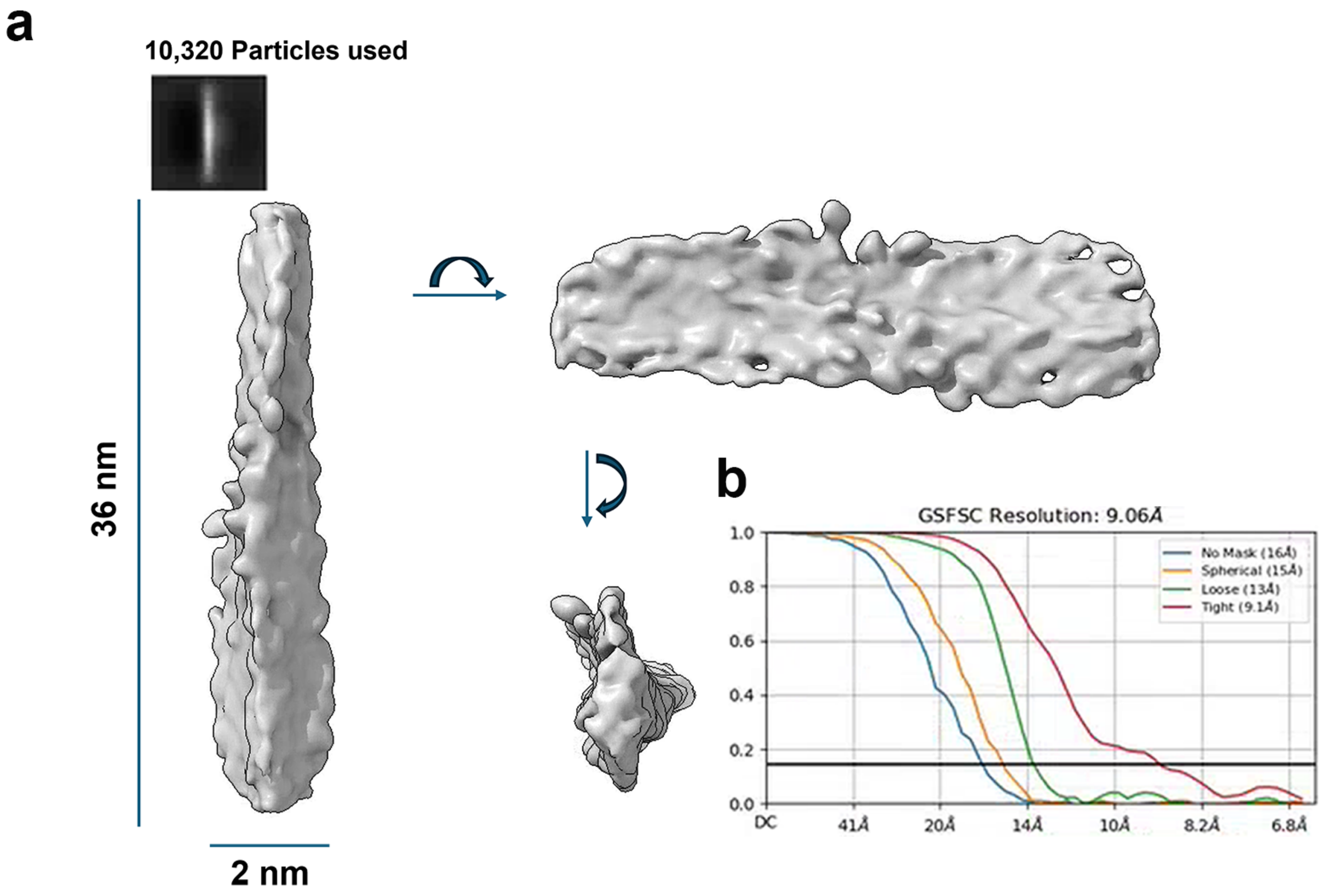

Figure S16. Helical Reconstruction CryoEM analysis for **morti07** fibrils. (a) After a few rounds of cleaning using 2D classification, one class with 10,320 particles has been selected for helical refinement. The reconstructed fibril map - views from different axes are shown. (b) *Fourier* Shell Correlation (FSC) curve estimates an overall resolution at FSC0.143 of ~9 Å.

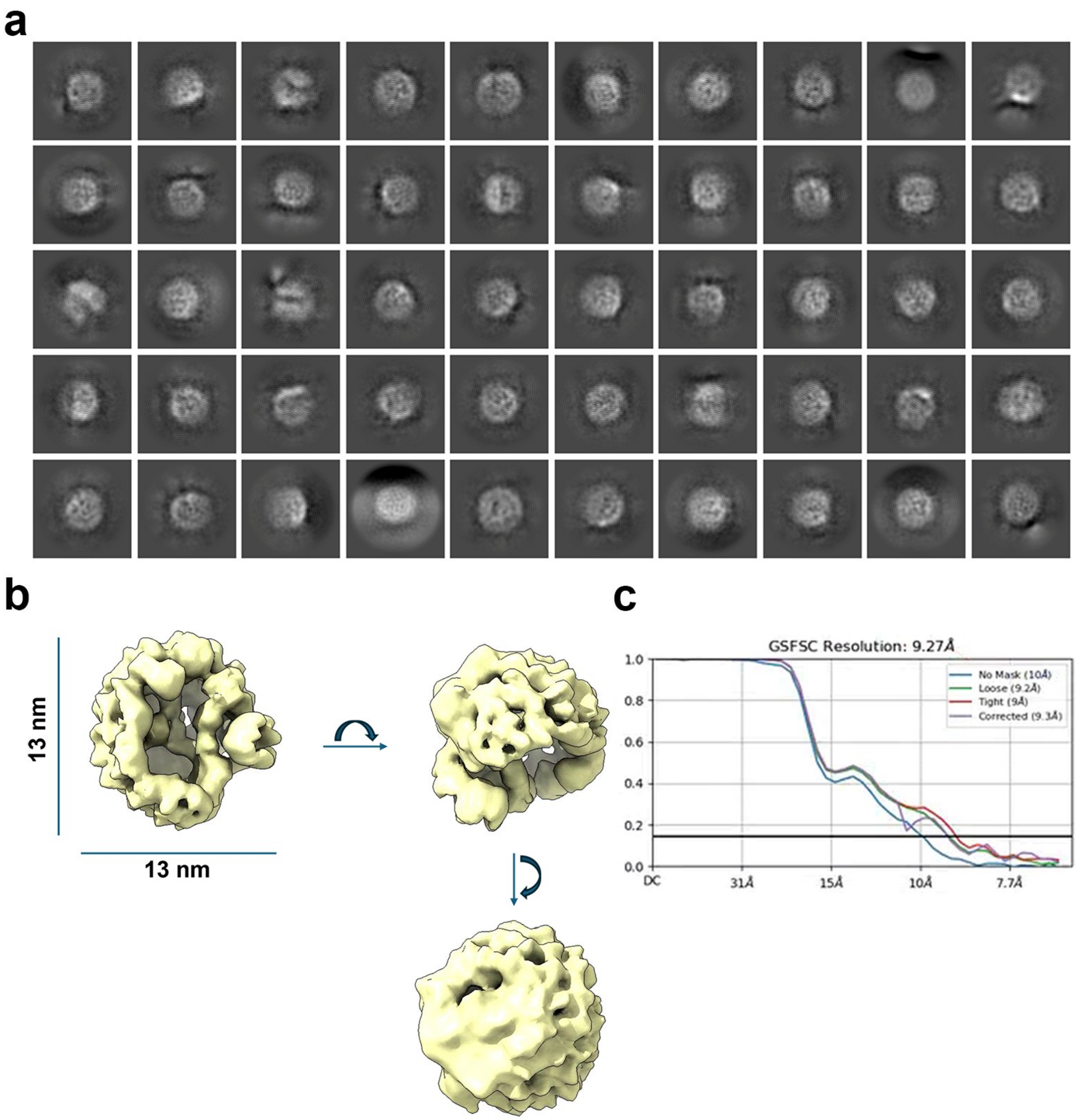

Figure S17. Single-Particle CryoEM analysis for mortiamide A spherical aggregates. (a) 2D classes obtained for mortiamide A spherical aggregates when cropped with a box size of 192 pixels. (b) After a few rounds of cleaning using 2D classification, the top classes containing 137,312 particles has been selected for non-uniform refinement. The reconstructed volume at an overall resolution of ~9.3 Å and views from different axes are shown. (c) *Fourier* Shell Correlation (FSC) curve for the 3D map.

Experimental Data

Table S2. Biological potency and PAMPA property of active cyclic heptapeptides

compound	R^1	R^2	R^3	R^4	R^5	R^6	R^7	AlogP	EC_{50} (μM)		P_{app} ($\times 10^{-6}$ cm/s)	log P_{app}
									SYO-1	MDA-MB-453		
**morti01**	D-Leu	L-Phe(3,4-diF)	L-Phe(3,4-diF)	L-Ala	D-Leu	L-Val	L-Glu(OEt)		1.8 ± 0.1	1.5 ± 0.1	0.38 ± 0.04	-6.42 ± 0.05
**morti08**	D-Leu	L-Phe(3,4-diF)	L-Ala	L-Phe(3,4-diF)	D-Leu	L-Val	L-Glu(OEt)	4.32	>30	>30	N/A	N/A
**morti09**	D-Leu	L-Ala	L-Phe(3,4-diF)	L-Phe(3,4-diF)	D-Leu	L-Val	L-Glu(OEt)		4.3 ± 0.7	3.4 ± 0.1	0.88 ± 0.07	-6.06 ± 0.03
**morti02**	D-Leu	L-Ala	L-Phe(3,4-diF)	L-Cha	D-Leu	L-Cha	L-Glu(OEt)		>30	>30	N/A	N/A
**morti13**	D-Leu	L-Cha	L-Phe(3,4-diF)	L-Ala	D-Leu	L-Cha	L-Glu(OEt)	5.97	0.6 ± 0.1	0.7 ± 0.1	0.012 ± 0.003	-7.91 ± 0.09
**morti14**	D-Leu	L-Phe(3,4-diF)	L-Ala	L-Cha	D-Leu	L-Cha	L-Glu(OEt)		9.7 ± 0.9	>30	N/A	N/A
**morti05**	D-Leu	L-Cha	L-Bip	L-Abu	D-Leu	L-Abu	L-Glu(OEt)		>30	>30	N/A	N/A
**morti07**	D-Leu	L-Abu	L-Cha	L-Abu	D-Leu	L-Bip	L-Glu(OEt)		0.8 ± 0.1	6.6 ± 3.4	0.015 ± 0.004	-7.85 ± 0.13
**morti10**	D-Leu	L-Abu	L-Cha	L-Bip	D-Leu	L-Abu	L-Glu(OEt)	5.90	1.7 ± 0.1	4.2 ± 0.7	0.014 ± 0.002	-7.86 ± 0.05
**morti11**	D-Leu	L-Abu	L-Bip	L-Cha	D-Leu	L-Abu	L-Glu(OEt)		2.2 ± 0.1	2.9 ± 0.4	0	N/A
**morti12**	D-Leu	L-Bip	L-Cha	L-Abu	D-Leu	L-Abu	L-Glu(OEt)		2.7 ± 0.1	2.7 ± 0.1	0.041 ± 0.008	-7.40 ± 0.08
**morti03**	D-Leu	L-Ala	L-Phe(4-CF_3)	L-Phe(4-NO_2)	D-Leu	L-Cha	L-Glu(OEt)	5.73	0.7 ± 0.1	0.6 ± 0.1	0.006 ± 0.004	-8.11 ± 0.09
**morti04**	D-Leu	L-Abu	L-Leu	L-Tyr(OMe)	D-Leu	L-Bip	L-Glu(OEt)	5.92	1.8 ± 0.2	1.0 ± 0.1	0.025 ± 0.009	-7.62 ± 0.15
**morti06**	D-Leu	L-Abu	L-Leu	L-hPhe	D-Leu	L-hPhe	L-Glu(OEt)	5.33	1.3 ± 0.1	1.4 ± 0.1	0.032 ± 0.01	-7.52 ± 0.17
**1A3**	D-Leu	L-Abu	L-hSer(OMe)	L-Cha	D-Leu	L-Cha	L-Glu(OEt)	4.11	8.0 ± 0.5	8.2 ± 0.6	5.52 ± 1.12	-5.26 ± 0.09
**2A9**	D-Leu	L-Abu	L-Cha	L-hSer(OMe)	D-Leu	L-Cha	L-Glu(OEt)		10.3 ± 0.6	11.6 ± 0.3	9.76 ± 1.13	-5.01 ± 0.05
**1A10**	D-Leu	L-Ala	L-Phe(4-CF_3)	L-hPhe	D-Leu	L-Val	L-Glu(OEt)		4.0 ± 0.6	7.5 ± 0.7	0.13 ± 0.50	-6.90 ± 0.16
**1C5**	D-Leu	L-Phe(4-CF_3)	L-hPhe	L-Ala	D-Leu	L-Val	L-Glu(OEt)	4.90	1.0*	2.3 ± 0.4	0.92 ± 0.13	-6.04 ± 0.06
**2B5**	D-Leu	L-hPhe	L-Phe(4-CF_3)	L-Ala	D-Leu	L-Val	L-Glu(OEt)		0.6*	0.5 ± .0.1	0.40 ± 0.03	-6.40 ± 0.04
**1C10**	D-Leu	L-Phe(4-Cl)	L-Tyr(OMe)	L-Abu	D-Leu	L-Abu	L-Glu(OEt)	4.36	10.0 ± 0.8	11.8 ± 0.5	11.00 ± 2.78	-4.97 ± 0.10
**2A11**	D-Leu	L-Abu	L-Phe(4-Cl)	L-Tyr(OMe)	D-Leu	L-Abu	L-Glu(OEt)		10.9 ± 2.8	10.1 ± 2.2	6.67 ± 1.93	-5.19 ± 0.12
**1D3**	D-Leu	L-Phe(4-Cl)	L-Leu	L-Abu	D-Leu	L-Leu	L-Glu(OEt)	4.76	7.9 ± 0.3	14.4 ± 0.5	9.83 ± 0.60	-5.01 ± 0.03
**1F3**	D-Leu	L-Abu	L-Leu	L-Leu	D-Leu	L-Phe(4-Cl)	L-Glu(OEt)		6.7 ± 0.2	7.6 ± 0.2	0.47 ± 0.10	-6.33 ± 0.09
**1D6**	D-Leu	L-Abu	L-Bip	L-hSer(OMe)	D-Leu	L-Cha	L-Glu(OEt)	4.96	5.4*	4.3 ± 0.4	0.17 ± 0.09	-6.80 ± 0.25
**1E10**	D-Leu	L-hSer(OMe)	L-Bip	L-Abu	D-Leu	L-Cha	L-Glu(OEt)		6.6 ± 0.4	14.8 ± 1.3	0.13 ± 0.03	-6.89 ± 0.09
**2A8**	D-Leu	L-Abu	L-hPhe	L-Phe(4-Cl)	D-Leu	L-Val	L-Glu(OEt)		8.1 ± 0.9	21.4 ± 5.4	6.90 ± 1.14	-5.16 ± 0.07
**2C6**	D-Leu	L-Abu	L-Phe(4-Cl)	L-hPhe	D-Leu	L-Val	L-Glu(OEt)	5.15	5.4 ± 0.9	5.3 ± 1.1	0.14 ± 0.03	-6.87 ± 0.09
**2D6**	D-Leu	L-hPhe	L-Phe(4-Cl)	L-Abu	D-Leu	L-Val	L-Glu(OEt)		1.1 ± 0.1	1.1 ± 0.1	1.63 ± 0.24	-5.79 ± 0.06
**1E7**	D-Leu	L-Abu	L-hPhe	L-Ala	D-Leu	L-Bip	L-Glu(OEt)		0.8 ± 0.1	1.0 ± 0.1	0.42 ± 0.06	-6.38 ± 0.06
**2B8**	D-Leu	L-hPhe	L-Abu	L-Ala	D-Leu	L-Bip	L-Glu(OEt)	5.16	2.9 ± 0.5	4.2 ± 0.4	0.83 ± 0.10	-6.09 ± 0.06
**2C8**	D-Leu	L-Ala	L-Abu	L-hPhe	D-Leu	L-Bip	L-Glu(OEt)		9.4 ± 1.4	>30	0.03 ± 0.01	-7.60 ± 0.14
**2B3**	D-Leu	L-Phe(3,4-diF)	L-Phe(4-Cl)	L-Ala	D-Leu	L-Val	L-Glu(OEt)	4.58	0.9 ± 0.1	0.8 ± 0.1	1.90 ± 0.32	-5.72 ± 0.07

**2C9**	D-Leu	L-Ala	L-Phe(4-Cl)	L-Phe(3,4-diF)	D-Leu	L-Val	L-Glu(OEt)		11.4 ± 1.8	>30	0.55 ± 0.08	-6.27 ± 0.06
**2E8**	D-Leu	L-Ala	L-Phe(4-Cl)	L-hPhe	D-Leu	L-Val	L-Glu(OEt)	4.62	3.5 ± 0.2	12.7 ± 1.3	0.28 ± 0.12	-6.58 ± 0.21
**2E11**	D-Leu	L-hPhe	L-Phe(4-Cl)	L-Ala	D-Leu	L-Val	L-Glu(OEt)		4.4 ± 1.2	3.0 ± 0.2	1.94 ± 0.25	-5.71 ± 0.06
**1B7**	D-Leu	L-hSer(OMe)	L-Phe(3,4-diF)	L-Ala	D-Leu	L-Phe(4-CF$_3$)	L-Glu(OEt)	3.60	7.2 ± 0.4	8.8 ± 0.5	4.90 ± 0.76	-5.31 ± 0.07
**1D9**	D-Leu	L-Val	L-Val	L-Val	D-Leu	L-Bip	L-Glu(OEt)	5.15	5.4*	5.2*	4.60 ± 1.68	-5.36 ± 0.19
**1E6**	D-Leu	L-Ala	L-hPhe	L-Ala	D-Leu	L-Phe(4-Cl)	L-Glu(OEt)	3.78	2.5 ± 0.1	5.0 ± 0.4	2.68 ± 0.36	-5.57 ± 0.06
**2C3**	D-Leu	L-Ala	L-^{2}Pal	L-hPhe	D-Leu	L-Cha	L-Glu(OEt)	4.34	5.9 ± 0.3	7.9 ± 0.5	3.36 ± 1.33	-5.50 ± 0.20
**2D2**	D-Leu	L-hPhe	L-Phe(4-Cl)	L-Ala	D-Leu	L-Abu	L-Glu(OEt)	4.30	3.2 ± 0.4	4.4*	4.61 ± 0.33	-5.34 ± 0.03
**2E5**	D-Leu	L-Val	L-Leu	L-Abu	D-Leu	L-Bip	L-Glu(OEt)	5.15	0.9 ± 0.1	1.1 ± 0.1	0.13 ± 0.01	-6.88 ± 0.04

*Standard error of mean cannot be determined due to high Hill slopes.

Table S3. Biological potency of **morti07** derivatives.

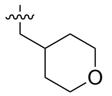

Thp

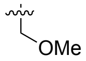

Ser(OMe)

compound	R^1	R^2	R^3	R^4	R^5	R^6	R^7	AlogP	EC$_{50}$ (μM)	
									SYO-1	MDA-MB-453
**7-A^1**	D-Ala	L-Abu	L-Cha	L-Abu	D-Leu	L-Bip	L-Glu(OEt)	4.67	5.6*	5.5*
**7-A^2**	D-Leu	L-Ala	L-Cha	L-Abu	D-Leu	L-Bip	L-Glu(OEt)	5.37	>10	>10
**7-A^3**	D-Leu	L-Abu	L-Ala	L-Abu	D-Leu	L-Bip	L-Glu(OEt)	3.67	>10	>10
**7-A^4**	D-Leu	L-Abu	L-Cha	L-Ala	D-Leu	L-Bip	L-Glu(OEt)	5.37	1.9 ± 0.1	2.2 ± 0.4
**7-A^5**	D-Leu	L-Abu	L-Cha	L-Abu	D-Ala	L-Bip	L-Glu(OEt)	4.67	>10	>10
**7-A^6**	D-Leu	L-Abu	L-Cha	L-Abu	D-Leu	L-Ala	L-Glu(OEt)	2.82	>10	>10
**7-A^7**	D-Leu	L-Abu	L-Cha	L-Abu	D-Leu	L-Bip	L-Ala	5.62	2.3*	>10
**7-Hse^2**	D-Leu	L-hSer(OMe)	L-Cha	L-Abu	D-Leu	L-Bip	L-Glu(OEt)	4.96	>10	>10
**7-Hse^4**	D-Leu	L-Abu	L-Cha	L-hSer(OMe)	D-Leu	L-Bip	L-Glu(OEt)	4.96	2.4 ± 0.1	3.6 ± 0.4
**7-Hse^{24}**	D-Leu	L-hSer(OMe)	L-Cha	L-hSer(OMe)	D-Leu	L-Bip	L-Glu(OEt)	4.02	>10	>10
**7-A^{17}**	D-Ala	L-Abu	L-Cha	L-Abu	D-Leu	L-Bip	L-Ala	4.39	>10	>10
**7-Hse^{2}A^7**	D-Leu	L-hSer(OMe)	L-Cha	L-Abu	D-Leu	L-Bip	L-Ala	4.68	>5	>10
**7-A^{57}**	D-Leu	L-Abu	L-Cha	L-Abu	D-Ala	L-Bip	L-Ala	4.39	>10	>10
**7-Hse^{4}A^7**	D-Leu	L-Abu	L-Cha	L-hSer(OMe)	D-Leu	L-Bip	L-Ala	4.68	1.6 ± 0.2	6.4*
**7-S^2**	D-Leu	L-Ser(OMe)	L-Cha	L-Abu	D-Leu	L-Bip	L-Glu(OEt)	4.89	4.8 ± 0.3	4.2*
**7-S^4**	D-Leu	L-Abu	L-Cha	L-Ser(OMe)	D-Leu	L-Bip	L-Glu(OEt)	4.89	1.8 ± 0.2	1.9 ± 0.3
**7-S^{2}A^7**	D-Leu	L-Ser(OMe)	L-Cha	L-Abu	D-Leu	L-Bip	L-Ala	4.62	4.9*	5.1*
**7-S^{4}A^7**	D-Leu	L-Abu	L-Cha	L-Ser(OMe)	D-Leu	L-Bip	L-Ala	4.62	3.0 ± 0.3	3.0 ± 0.3
**7-S^{2}Pye^7**	D-Leu	L-Ser(OMe)	L-Cha	L-Abu	D-Leu	L-Bip	L-Pye	4.57	>10	>10

**7-A^{1}Pye^7**	D-Ala	L-Abu	L-Cha	L-Abu	D-Leu	L-Bip	L-Pye	4.34	>10	>10
**7-Hse^{2}Pye^7**	D-Leu	L-hSer(OMe)	L-Cha	L-Abu	D-Leu	L-Bip	L-Pye	4.63	>10	>10
**7-A^{5}Pye^7**	D-Leu	L-Abu	L-Cha	L-Abu	D-Ala	L-Bip	L-Pye	4.34	>10	>10
**7-S^{4}Pye^7**	D-Leu	L-Abu	L-Cha	L-Ser(OMe)	D-Leu	L-Bip	L-Pye	4.57	>10	>10
**7-Hse^{4}Pye^7**	D-Leu	L-Abu	L-Cha	L-hSer(OMe)	D-Leu	L-Bip	L-Pye	4.63	>10	>10
**7-S^{2}NMe$_2$^{7}**	D-Leu	L-Ser(OMe)	L-Cha	L-Abu	D-Leu	L-Bip	L-Glu(NMe$_2$)	4.10	>10	>10
**7-Hse^{2}NMe$_2$^{7}**	D-Leu	L-hSer(OMe)	L-Cha	L-Abu	D-Leu	L-Bip	L-Glu(NMe$_2$)	4.17	>10	>10
**7-S^{4}NMe$_2$^{7}**	D-Leu	L-Abu	L-Cha	L-Ser(OMe)	D-Leu	L-Bip	L-Glu(NMe$_2$)	4.10	>10	>10
**7-Hse^{4}NMe$_2$^{7}**	D-Leu	L-Abu	L-Cha	L-hSer(OMe)	D-Leu	L-Bip	L-Glu(NMe$_2$)	4.17	>10	>10
**7-Thp^3**	D-Leu	L-Abu	L-Thp	L-Abu	D-Leu	L-Bip	L-Glu(OEt)	4.13	>5	>5

*Standard error of mean cannot be determined due to high Hill slopes.

Table S4. Comparison of bioactivity between active cyclic heptapeptides and their enantiomeric counterpart.

**compound**	**EC$_{50}$ μM (Hill slope)**	
	**SYO-1**	**MDA**
**morti01**	1.8 ± 0.1 (-3.6)	1.5 ± 0.1 (-8.7)
**morti01 enantiomer**	2.8 ± 0.2 (-3.3)	5.3* (-16.3)
**morti03**	0.7 ± 0.1 (-2.5)	0.6 ± 0.1 (-3.9)
**morti03 enantiomer**	0.8 ± 0.1 (-2.1)	0.4 ± 0.1 (-3.3)
**morti04**	1.8 ± 0.2 (-1.8)	1.0 ± 0.1 (-2.3)
**morti04 enantiomer**	6.5 ± 5.3 (-1.5)	2.5 ± 1.0 (-0.9)
**morti06**	1.3 ± 0.1 (-2.5)	1.4 ± 0.1 (-1.6)
**morti06 enantiomer**	>10 (-21.8)	>10 (-27.3)
**morti07**	0.8 ± 0.1 (-6.0)	6.6 ± 3.4 (-0.7)
**morti07 enantiomer**	1.4 ± 0.2 (-2.0)	2.3 ± 0.4 (-1.5)
**morti09**	4.3 ± 0.7 (-10.3)	3.4 ± 0.1 (-3.9)
**morti09 enantiomer**	>10 (-21.5)	4.9* (-28.6)
**morti10**	1.7 ± 0.1 (-2.3)	4.2 ± 0.7 (-1.2)
**morti10 enantiomer**	1.8 ± 1.0 (-0.9)	>10 (-0.3)
**morti11**	2.2 ± 0.1 (-1.8)	2.9 ± 0.4 (-1.1)
**morti11 enantiomer**	2.9 ± 0.4 (-1.7)	>10 (-0.3)
**morti13**	0.6 ± 0.1 (-12.9)	0.7 ± 0.1 (-7.2)
**morti13 enantiomer**	0.5 ± 0.1(-5.4)	0.3 ± 0.1 (-1.8)
**1E6**	2.5 ± 0.1 (-3.2)	5.0 ± 0.4 (-1.8)
**1E6 enantiomer**	2.2 ± 0.1 (-5.4)	4.4 ± 0.3 (-2.1)
**1E7**	0.8 ± 0.1 (-2.2)	1.0 ± 0.1 (-1.8)
**1E7 enantiomer**	1.1 ± 0.3 (-11.5)	1.2 ± 0.1 (-5.5)
**2A8**	8.1 ± 0.9 (-4.5)	21.4 ± 5.4 (-2.0)
**2A8 enantiomer**	5.5 ± 0.4 (-7.6)	10.4 ± 0.7 (-2.3)

**2A11**	10.9 ± 2.8 (-10.5)	10.1 ± 2.2 (-8.5)
**2A11 enantiomer**	5.7* (-24.1)	5.1 ± 0.3 (-8.1)
**2E5**	0.9 ± 0.1 (-3.7)	1.1 ± 0.1 (-3.1)
**2E5 enantiomer**	0.6* (-51.9)	0.7 ± 0.1 (-4.0)

*Standard error of mean cannot be determined due to high Hill slopes.

Table S5. Experimental data of natural product mortiamides and their derivatives.

compound	R^1	R^2	R^3	R^4	R^5	R^6	R^7	AlogP	EC_{50} (μM)		P_{app} ($x10^{-6}$ cm/s)	log P_{app}	solubility (μM)
									SYO-1	MDA-MB-453			
**Mortiamide A**	L-Phe	D-Val	D-Val	D-Val	L-Leu	D-Val	D-Phe	4.52	>30	>30	6.02 ± 0.69	-5.22 ± 0.05	0.04 ± 0.01
**Mortiamide A enantiomer**	D-Phe	L-Val	L-Val	L-Val	D-Leu	L-Val	L-Phe		>30	>30	5.85 ± 0.33	-5.23 ± 0.02	N/A
**Mortiamide A epimer**	L-Phe	D-Val	D-Val	L-Val	L-Leu	D-Val	D-Phe		>30	>30	12.25 ± 1.42	-4.91 ± 0.05	4.1 ± 0.7
**Mortiamide B**	L-Phe	D-Val	D-Val	D-Val	L-Phe	D-Val	D-Phe	4.85	18.2*	>30	9.61 ± 1.01	-5.02 ± 0.05	1.4 ± 0.1
**Mortiamide B enantiomer**	D-Phe	L-Val	L-Val	L-Val	D-Phe	L-Val	L-Phe		21.0*	>30	9.57 ± 0.60	-5.02 ± 0.03	N/A
**Mortiamide B epimer**	L-Phe	D-Val	D-Val	L-Val	L-Phe	D-Val	D-Phe		>30	>30	9.69 ± 0.55	-5.01 ± 0.02	0.8 ± 0.1
**Mortiamide D**	L-Phe	D-Ala	D-Ile	D-Ile	L-Phe	D-Val	D-Leu	4.59	6.1 ± 0.2	>30	0.29 ± 0.07	-6.55 ± 0.10	0.02 ± 0.01
**Mortiamide D enantiomer**	D-Phe	L-Ala	L-Ile	L-Ile	D-Phe	L-Val	L-Leu		10.4 ± 2.2	>30	0.50 ± 0.12	-6.31 ± 0.10	N/A
**Mortiamide D epimer**	L-Phe	D-Ala	D-Ile	L-Ile	L-Phe	D-Val	D-Leu		>30	>30	16.59 ± 2.43	-4.78 ± 0.06	13.4 ± 1.4

*Standard error of mean cannot be determined due to high Hill slopes.

NMR Spectra

Mortiamide A

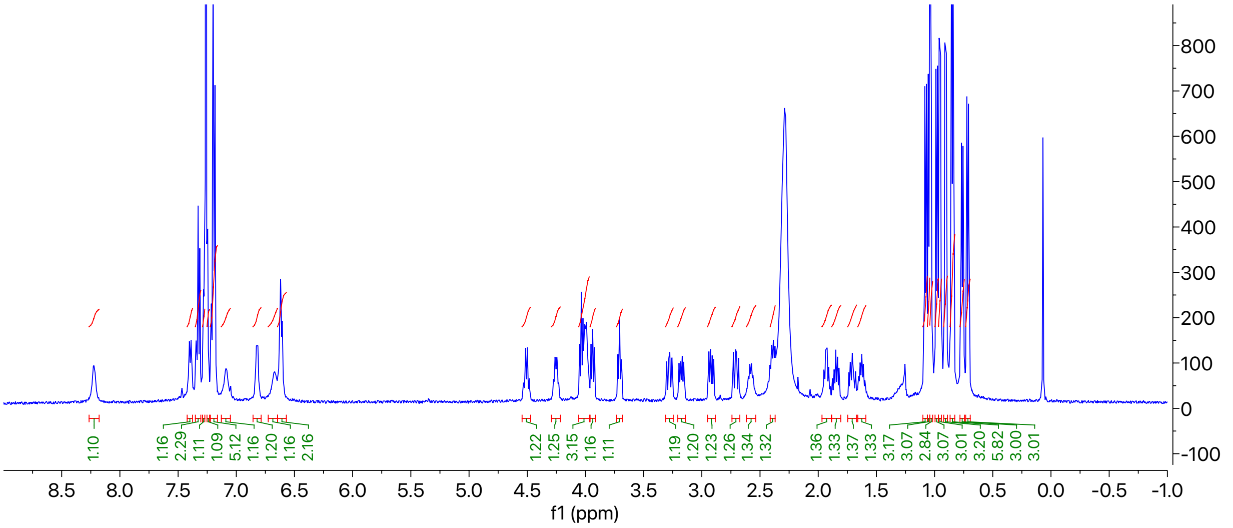

^{1}H NMR (500 MHz, Chloroform-*d*) δ 8.22 (br, 1H), 7.40 (d, *J* = 7.8 Hz, 1H), 7.33 (t, *J* = 7.4 Hz, 2H), 7.28 (s, 1H), 7.25 (s, 1H), 7.22 – 7.16 (m, 4H), 7.09 (br, 1H), 6.82 (d, *J* = 6.9 Hz, 1H), 6.67 (br, 1H), 6.61 (m, 2H), 4.51 (q, *J* = 7.9 Hz, 1H), 4.25 (q, *J* = 7.4 Hz, 1H), 4.02 (m, 3H), 3.94 (dd, *J* = 8.7, 6.4 Hz, 1H), 3.71 (t, *J* = 7.3 Hz, 1H), 3.28 (dd, *J* = 13.6, 9.1 Hz, 1H), 3.18 (dd, *J* = 13.6, 7.0 Hz, 1H), 2.92 (dd, *J* = 13.8, 6.8 Hz, 1H), 2.71 (dd, *J* = 13.7, 8.7 Hz, 1H), 2.62 – 2.54 (m, 1H), 2.41 – 2.37 (m, 2H), 1.93 (m, 1H), 1.85 (m, 1H), 1.75 – 1.67 (m, 1H), 1.63 (m, 1H), 1.08 (d, *J* = 6.6 Hz, 3H), 1.05 (d, *J* = 6.7 Hz, 3H), 1.03 (d, *J* = 6.7 Hz, 3H), 0.98 (d, *J* = 6.7 Hz, 3H), 0.95 (d, *J* = 6.5 Hz, 3H), 0.91 (d, *J* = 6.5 Hz, 3H), 0.85 (d, *J* = 6.8, 6H), 0.76 (d, *J* = 6.8 Hz, 3H), 0.72 (d, *J* = 6.8 Hz, 3H).

Mortiamide A enantiomer

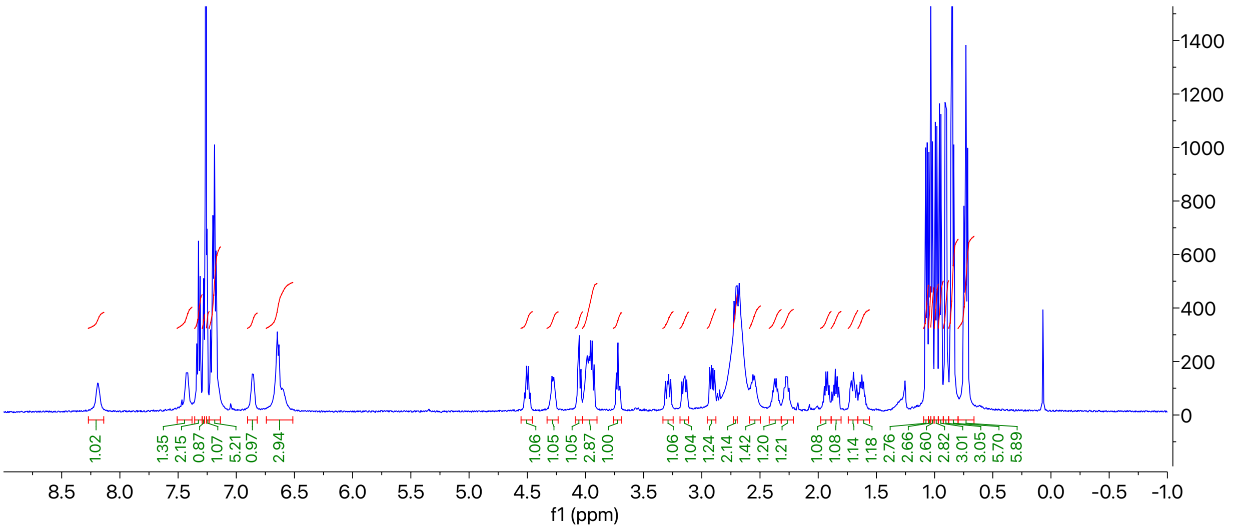

^{1}H NMR (500 MHz, Chloroform-*d*) δ 8.19 (br, 1H), 7.42 (d, *J* = 7.4 Hz, 1H), 7.32 (t, *J* = 7.4 Hz, 2H), 7.28 (s, 1H), 7.25 (s, 1H), 7.23 – 7.14 (m, 5H), 6.86 (d, *J* = 6.9 Hz, 1H), 6.64 (br, 3H), 4.50 (q, *J* = 7.8 Hz, 1H), 4.28 (q, *J* = 8.3 Hz, 1H), 4.05 (t, *J* = 7.2 Hz, 1H), 3.96 (m, 3H), 3.72 (t, *J* = 7.1 Hz, 1H), 3.29 (dd, *J* = 13.7, 8.8 Hz, 1H), 3.15 (dd, *J* = 13.6, 7.3 Hz, 1H), 2.91 (dd, *J* = 13.7, 6.9 Hz, 1H), 2.73 – 2.70 (m, 1H), 2.55 (m, 1H), 2.37 (m, 1H), 2.27 (m, 1H), 1.92 (m, 1H), 1.89 – 1.80 (m, 1H), 1.74 – 1.66 (m, 1H), 1.62 (m, 1H), 1.07 (d, *J* = 6.6 Hz, 3H), 1.04 (d, *J* = 7.2 Hz, 3H), 1.02 (d, *J* = 7.2 Hz, 3H), 0.99 (d, *J* = 6.7 Hz, 3H), 0.95 (d, *J* = 6.5 Hz, 3H), 0.90 (d, *J* = 6.5 Hz, 3H), 0.85 (t, *J* = 6.2 Hz, 6H), 0.73 (t, *J* = 7.8 Hz, 6H).

Mortiamide A epimer

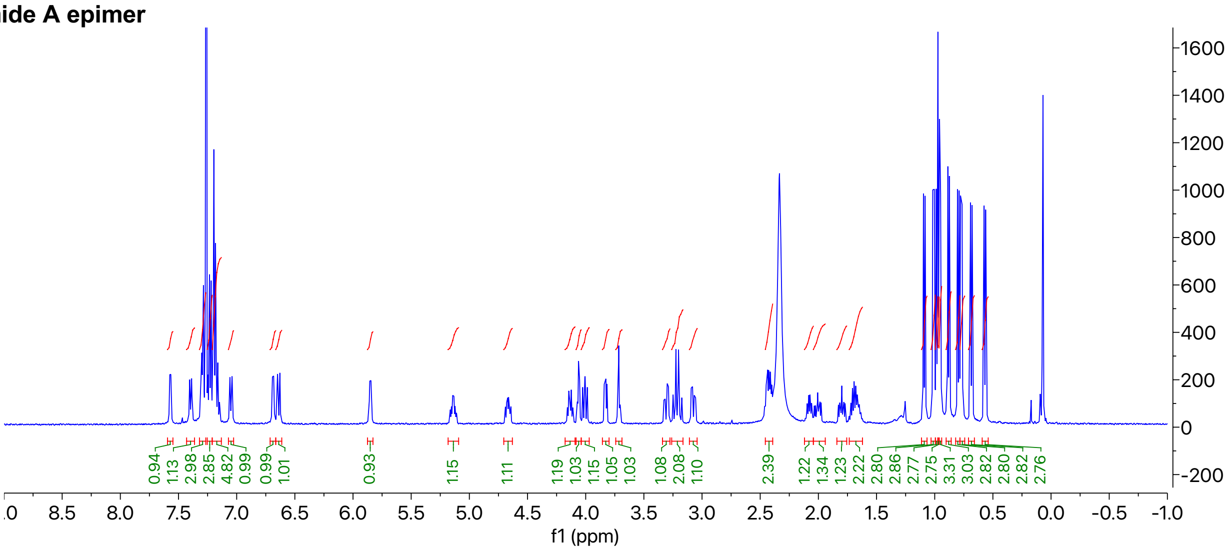

^{1}H NMR (500 MHz, Chloroform-*d*) δ 7.57 (d, *J* = 4.6 Hz, 1H), 7.40 (d, *J* = 8.3 Hz, 1H), 7.29 (m, 3H), 7.25 – 7.21 (m, 3H), 7.21 – 7.13 (m, 5H), 7.05 (d, *J* = 9.1 Hz, 1H), 6.69 (d, *J* = 5.2 Hz, 1H), 6.64 (d, *J* = 10.0 Hz, 1H), 5.85 (d, *J* = 5.2 Hz, 1H), 5.14 (td, *J* = 10.5, 5.2 Hz, 1H), 4.67 (td, *J* = 9.4, 5.3 Hz, 1H), 4.13 (dt, *J* = 11.1, 6.0 Hz, 1H), 4.06 (dd, *J* = 5.1, 3.8 Hz, 1H), 4.01 (dd, *J* = 10.8, 9.1 Hz, 1H), 3.83 (dd, *J* = 7.2, 4.6 Hz, 1H), 3.72 (t, *J* = 5.1 Hz, 1H), 3.31 (dd, *J* = 14.3, 5.3 Hz, 1H), 3.21 (q, *J* = 14.0, 13.1 Hz, 2H), 3.07 (dd, *J* = 13.2, 6.0 Hz, 1H), 2.46 – 2.39 (m, 2H), 2.12 – 2.04 (m, 1H), 2.01 (m, 1H), 1.80 (m, 1H), 1.69 (m, 2H), 1.09 (d, *J* = 7.0 Hz, 3H), 1.01 (d, *J* = 6.9 Hz, 3H), 0.98 (d, *J* = 7.2 Hz, 3H), 0.96 (d, *J* = 7.0 Hz, 3H), 0.95 (d, *J* = 4.6 Hz, 3H), 0.88 (d, *J* = 6.6 Hz, 3H), 0.80 (d, *J* = 6.9 Hz, 3H), 0.77 (d, *J* = 6.7 Hz, 3H), 0.69 (d, *J* = 6.9 Hz, 3H), 0.57 (d, *J* = 6.8 Hz, 3H).

Mortiamide B

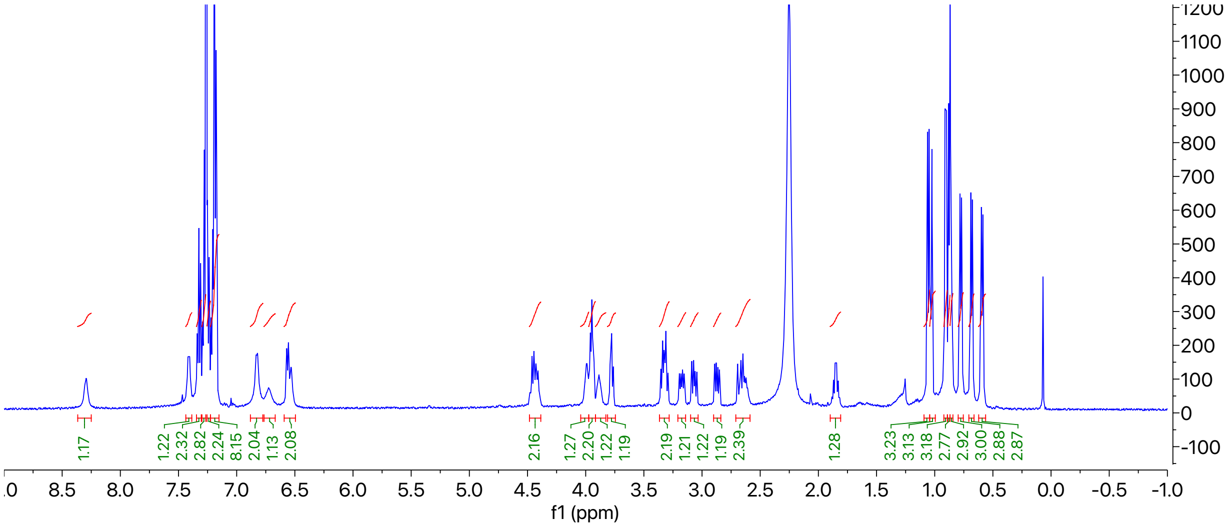

^{1}H NMR (500 MHz, Chloroform-*d*) δ 8.30 (br, 1H), 7.41 (d, *J* = 7.5 Hz, 1H), 7.33 (t, *J* = 7.4 Hz, 2H), 7.29 (m, 3H), 7.24 (m, 2H), 7.19 (m, 8H), 6.83 (br, 2H), 6.72 (br, 1H), 6.59 – 6.50 (m, 2H), 4.44 (m, 2H), 3.99 (m, 1H), 3.97 – 3.92 (m, 2H), 3.88 (m, 1H), 3.78 (t, *J* = 7.3 Hz, 1H), 3.32 (dt, *J* = 13.6, 8.5 Hz, 2H), 3.17 (dd, *J* = 13.7, 6.9 Hz, 1H), 3.07 (dd, *J* = 13.7, 7.9 Hz, 1H), 2.87 (dd, *J* = 13.7, 7.0 Hz, 1H), 2.66 (m, 2H), 1.85 (m, 1H), 1.05 (d, *J* = 6.7 Hz, 3H), 1.03 (d, *J* = 6.7 Hz, 3H), 0.91 (d, *J* = 6.7 Hz, 3H), 0.88 (d, *J* = 6.6 Hz, 3H), 0.86 (d, *J* = 6.7 Hz, 3H), 0.78 (d, *J* = 6.8 Hz, 3H), 0.68 (d, *J* = 6.8 Hz, 3H), 0.59 (d, *J* = 6.8 Hz, 3H).

Mortiamide B enantiomer

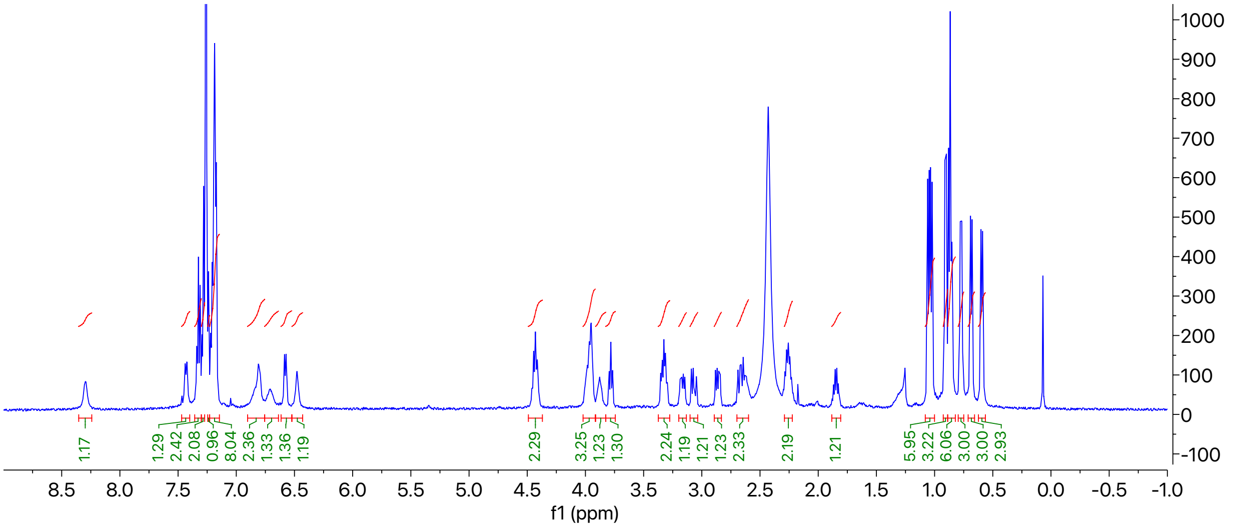

^{1}H NMR (500 MHz, Chloroform-*d*) δ 8.30 (br, 1H), 7.43 (d, *J* = 7.6 Hz, 1H), 7.33 (m, 2H), 7.29 (m, 2H), 7.24 (s, 1H), 7.23 – 7.14 (m, 8H), 6.80 (br, 2H), 6.71 (br, 1H), 6.58 (d, *J* = 6.9 Hz, 1H), 6.48 (br, 1H), 4.44 (m, 2H), 4.02 – 3.92 (m, 3H), 3.88 (m, 1H), 3.78 (t, *J* = 7.3 Hz, 1H), 3.33 (dt, *J* = 13.7, 7.1 Hz, 2H), 3.17 (dd, *J* = 13.8, 6.9 Hz, 1H), 3.07 (dd, *J* = 13.7, 7.9 Hz, 1H), 2.86 (dd, *J* = 13.7, 7.0 Hz, 1H), 2.65 (m, 2H), 2.26 (m, 2H), 1.85 (m, 1H), 1.04 (m, 6H), 0.91 (d, *J* = 6.7 Hz, 3H), 0.87 (t, *J* = 6.8 Hz, 6H), 0.77 (d, *J* = 6.8 Hz, 3H), 0.68 (d, *J* = 6.8 Hz, 3H), 0.59 (d, *J* = 6.8 Hz, 3H).

Mortiamide B epimer

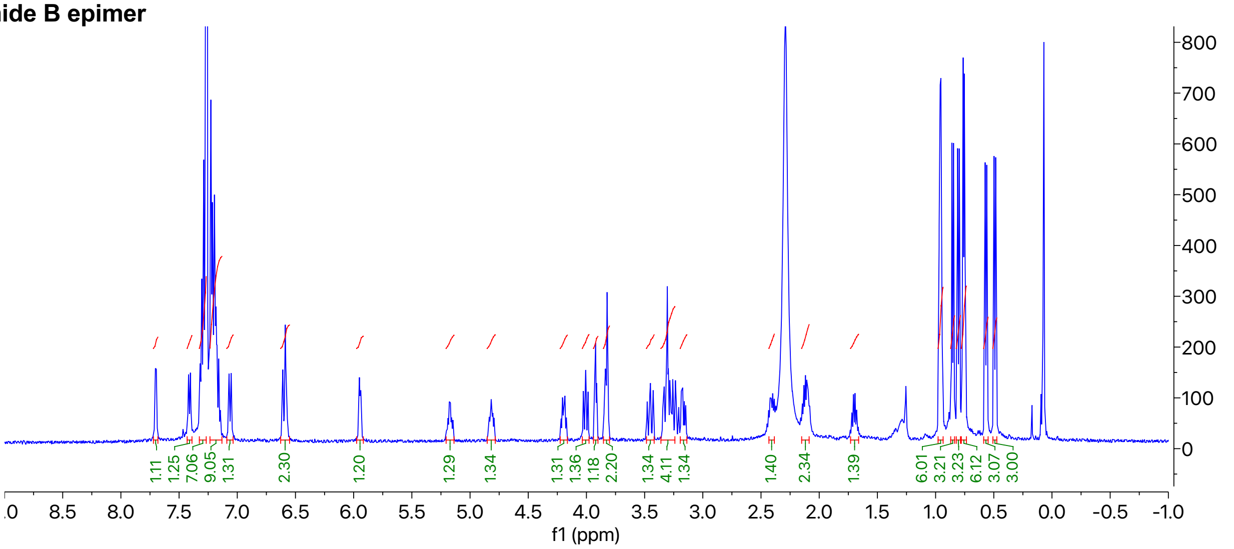

^{1}H NMR (500 MHz, Chloroform-*d*) δ 7.70 (d, *J* = 4.3 Hz, 1H), 7.41 (d, *J* = 8.2 Hz, 1H), 7.33 – 7.27 (m, 7H), 7.23 – 7.13 (m, 9H), 7.06 (d, *J* = 9.3 Hz, 1H), 6.63 – 6.55 (m, 2H), 5.95 (d, *J* = 5.1 Hz, 1H), 5.17 (td, *J* = 10.6, 5.2 Hz, 1H), 4.82 (ddd, *J* = 12.5, 8.3, 4.6 Hz, 1H), 4.19 (dt, *J* = 10.6, 6.1 Hz, 1H), 4.04 – 3.98 (m, 1H), 3.92 (t, *J* = 4.3 Hz, 1H), 3.83 (dt, *J* = 10.0, 4.7 Hz, 2H), 3.45 (dd, *J* = 14.2, 11.4 Hz, 1H), 3.36 – 3.24 (m, 4H), 3.17 (dd, *J* = 13.2, 6.2 Hz, 1H), 2.40 (m, 1H), 2.15 – 2.09 (m, 2H), 1.69 (m, 1H), 0.96 (dd, *J* = 6.7, 3.4 Hz, 6H), 0.85 (d, *J* = 6.9 Hz, 3H), 0.80 (d, *J* = 7.0 Hz, 3H), 0.76 (dd, *J* = 6.8, 2.3 Hz, 6H), 0.57 (d, *J* = 6.8 Hz, 3H), 0.49 (d, *J* = 7.0 Hz, 3H).

Mortiamide D

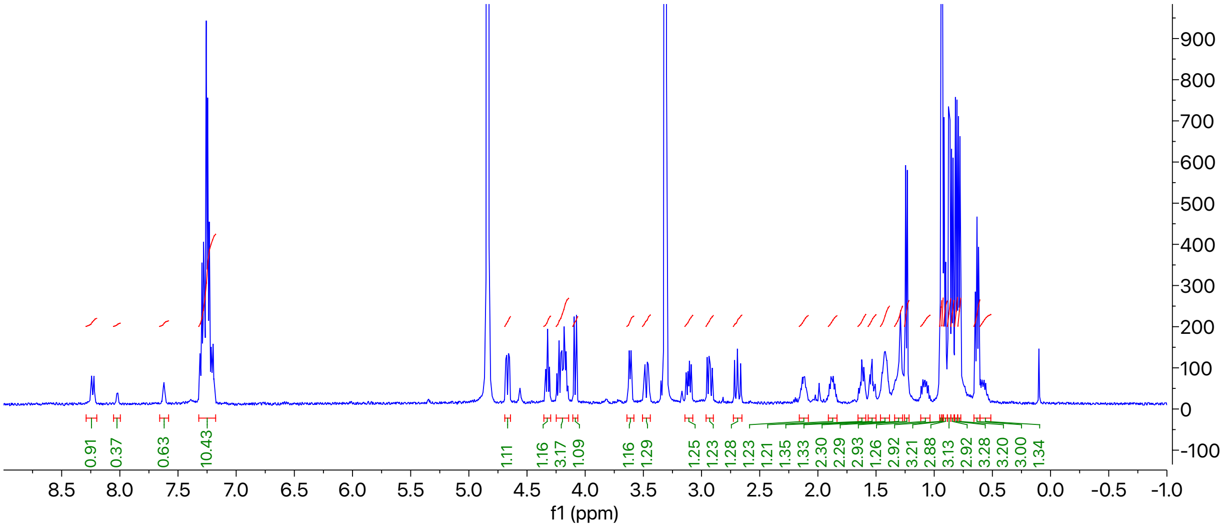

^{1}H NMR (500 MHz, Methanol-d_4) δ 8.23 (d, J = 10.0 Hz, 1H), 8.02 (d, J = 4.9 Hz, 1H), 7.62 (br, 1H), 7.32 – 7.18 (m, 10H), 4.67 (dd, J = 11.9, 4.0 Hz, 1H), 4.32 (t, J = 8.1 Hz, 1H), 4.25 – 4.14 (m, 3H), 4.08 (d, J = 9.9 Hz, 1H), 3.61 (d, J = 8.1 Hz, 1H), 3.48 (dd, J = 14.1, 4.0 Hz, 1H), 3.11 (dd, J = 13.6, 8.0 Hz, 1H), 2.93 (dd, J = 13.5, 8.2 Hz, 1H), 2.69 (dd, J = 14.2, 11.9 Hz, 1H), 2.16 – 2.08 (m, 1H), 1.87 (m, 1H), 1.62 (m, 1H), 1.53 (m, 1H), 1.42 (m, 2H), 1.29 (m, 2H), 1.24 (d, J = 7.4 Hz, 3H), 1.07 (m, 1H), 0.94 (d, J = 1.6 Hz, 3H), 0.93 (d, J = 1.7 Hz, 3H), 0.91 (d, J = 7.3 Hz, 3H), 0.87 (d, J = 6.5 Hz, 3H), 0.84 (d, J = 6.5 Hz, 3H), 0.81 (d, J = 6.6 Hz, 3H), 0.78 (d, J = 6.6 Hz, 3H), 0.66 – 0.61 (m, 3H), 0.61 – 0.51 (m, 1H).

Mortiamide D enantiomer

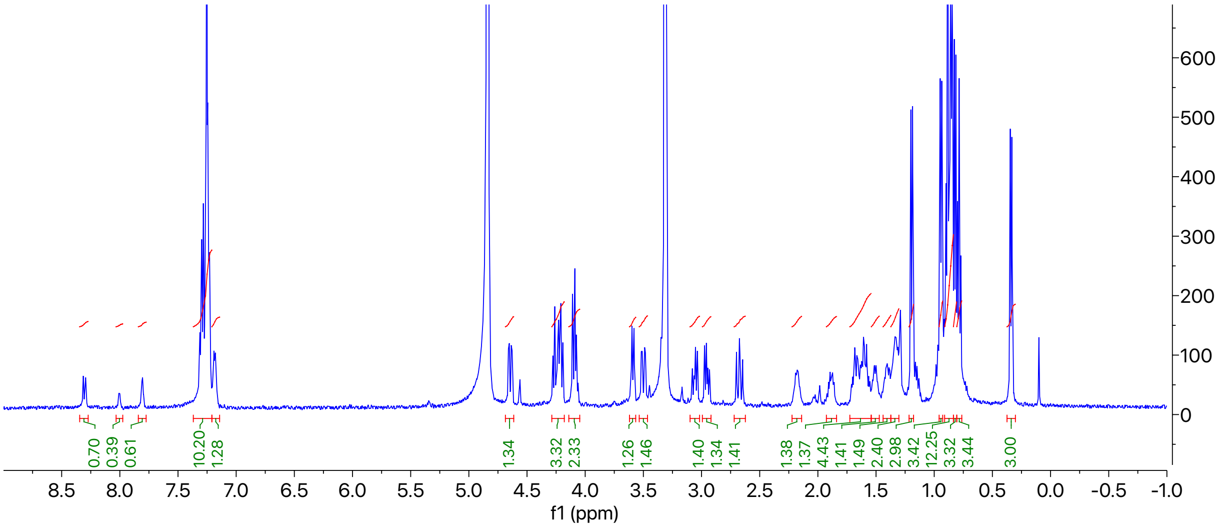

^{1}H NMR (500 MHz, Methanol-d_4) δ 8.30 (d, J = 10.1 Hz, 1H), 8.00 (d, J = 5.0 Hz, 1H), 7.81 (br, 1H), 7.37 – 7.21 (m, 10H), 7.18 (m, 1H), 4.64 (dd, J = 12.0, 3.8 Hz, 1H), 4.29 – 4.18 (m, 3H), 4.14 – 4.05 (m, 2H), 3.59 (d, J = 8.7 Hz, 1H), 3.50 (dd, J = 14.3, 3.9 Hz, 1H), 3.06 (dd, J = 13.4, 8.4 Hz, 1H), 2.95 (dd, J = 13.4, 7.8 Hz, 1H), 2.67 (dd, J = 14.2, 12.1 Hz, 1H), 2.18 (m, 1H), 1.89 (m, 1H), 1.73 – 1.54 (m, 4H), 1.51 (m, 1H), 1.44 – 1.37 (m, 1H), 1.33 (m, 2H), 1.19 (d, J = 7.3 Hz, 3H), 0.94 (d, J = 6.5 Hz, 3H), 0.91 – 0.83 (m, 12H), 0.82 (d, J = 6.6 Hz, 3H), 0.79 (t, J = 7.3 Hz, 3H), 0.34 (d, J = 6.8 Hz, 3H).

Mortiamide D epimer

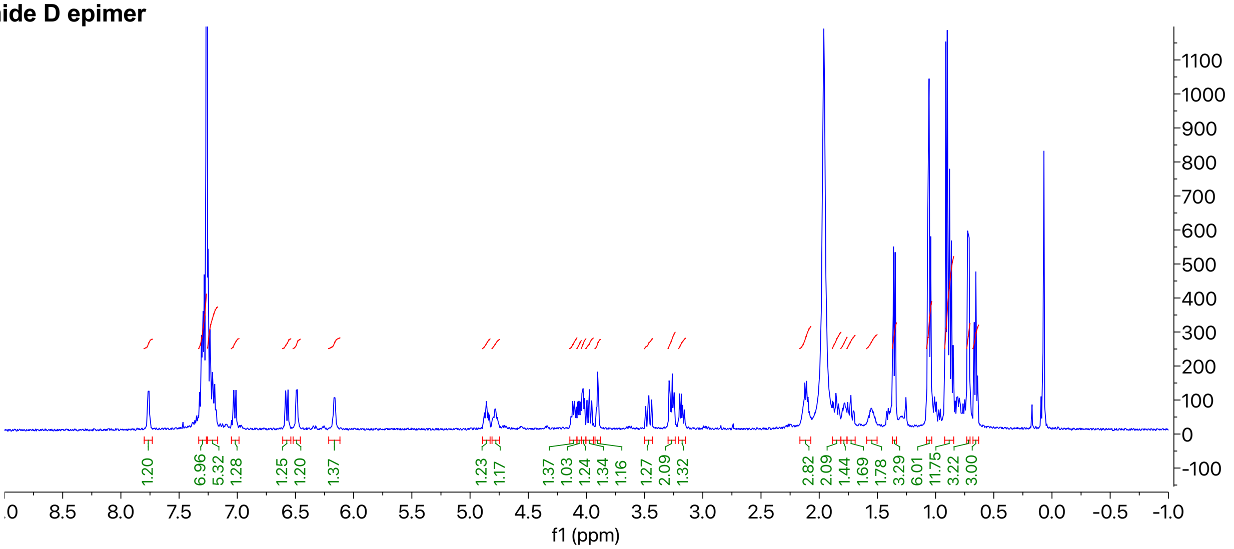

^{1}H NMR (500 MHz, Chloroform-*d*) δ 7.76 (d, *J* = 4.5 Hz, 1H), 7.33 – 7.27 (m, 7H), 7.25 – 7.17 (m, 5H), 7.02 (d, *J* = 9.2 Hz, 1H), 6.57 (d, *J* = 9.9 Hz, 1H), 6.49 (d, *J* = 4.7 Hz, 1H), 6.16 (d, *J* = 4.8 Hz, 1H), 4.89 – 4.83 (m, 1H), 4.78 (m, 1H), 4.11 (m, 1H), 4.06 (m, 1H), 4.03 (m, 1H), 3.97 (dd, *J* = 11.0, 9.2 Hz, 1H), 3.90 (t, *J* = 4.3 Hz, 1H), 3.47 (dd, *J* = 14.3, 11.8 Hz, 1H), 3.30 – 3.24 (m, 2H), 3.18 (dd, *J* = 13.5, 7.4 Hz, 1H), 2.12 (m, 3H), 1.86 (m, 2H), 1.78 (m, 1H), 1.73 (m, 2H), 1.56 (m, 2H), 1.35 (d, *J* = 7.3 Hz, 3H), 1.06 (t, *J* = 6.9 Hz, 6H), 0.92 – 0.84 (m, 12H), 0.72 (d, *J* = 6.9 Hz, 3H), 0.65 (t, *J* = 7.1 Hz, 3H).

Morti07

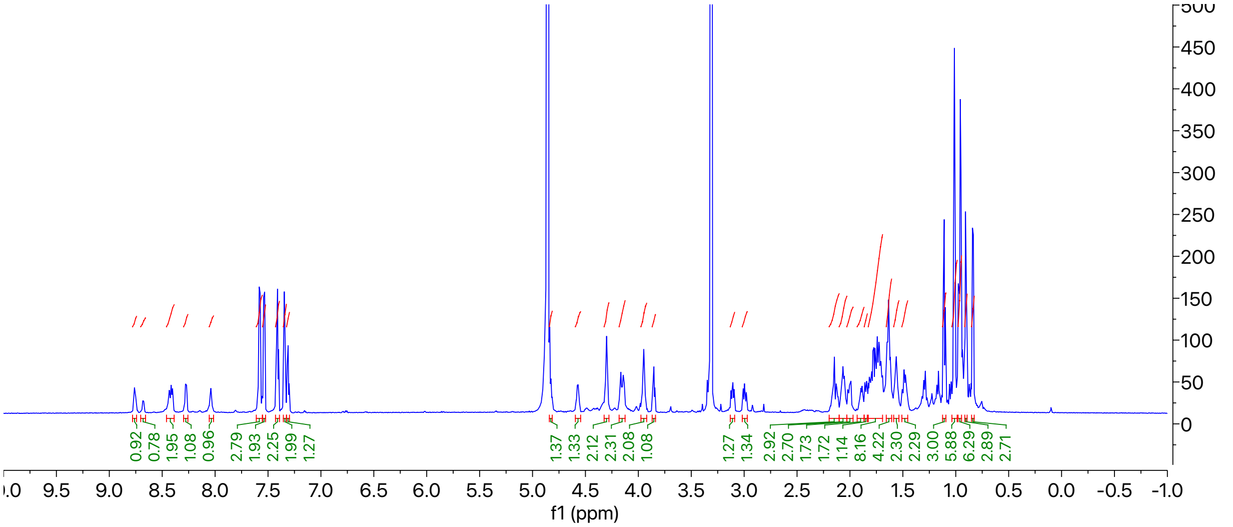

^{1}H NMR (800 MHz, Methanol-*d*$_4$) δ 8.76 (d, *J* = 7.7 Hz, 1H), 8.68 (d, *J* = 8.0 Hz, 1H), 8.46 – 8.39 (m, 2H), 8.27 (d, *J* = 8.7 Hz, 1H), 8.04 (br, 1H), 7.58 (d, *J* = 7.7 Hz, 3H), 7.55 – 7.52 (m, 2H), 7.41 (t, *J* = 7.5 Hz, 2H), 7.34 (d, *J* = 7.7 Hz, 2H), 7.31 (t, *J* = 7.3 Hz, 1H), 4.83 (m, 1H), 4.57 (q, *J* = 8.2 Hz, 1H), 4.30 (m, 2H), 4.15 (m, 2H), 3.95 (m, 2H), 3.85 (t, *J* = 7.4 Hz, 1H), 3.11 (dd, *J* = 14.2, 7.9 Hz, 1H), 2.99 (dd, *J* = 14.1, 8.6 Hz, 1H), 2.15 (m, 3H), 2.06 (m, 3H), 2.03 – 1.97 (m, 2H), 1.89 (m, 2H), 1.85 (m, 1H), 1.83 – 1.69 (m, 8H), 1.64 (m, 4H), 1.56 (m, 2H), 1.51 – 1.45 (m, 2H), 1.11 (t, *J* = 7.1 Hz, 3H), 1.01 (m, 6H), 0.98 – 0.95 (m, 6H), 0.90 (d, *J* = 6.6 Hz, 3H), 0.84 (d, *J* = 6.7 Hz, 3H).

Morti07

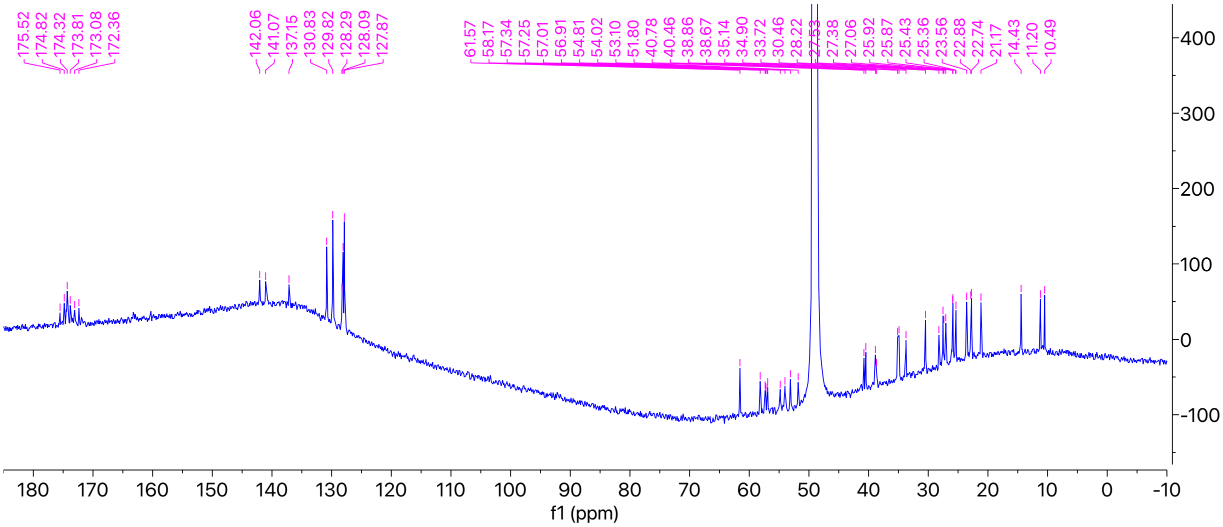

^{13}C NMR (201 MHz, Methanol-*d*$_4$) δ 175.52, 174.82, 174.32, 173.81, 173.08, 172.36, 142.06, 141.07, 137.15, 130.83, 129.82, 128.29, 128.09, 127.87, 61.57, 58.17, 57.34, 57.25, 57.01, 56.91, 54.81, 54.02, 53.10, 51.80, 40.78, 40.46, 38.86, 38.67, 35.14, 34.90, 33.72, 30.46, 28.22, 27.53, 27.38, 27.06, 25.92, 25.87, 25.43, 25.36, 23.56, 22.88, 22.74, 21.17, 14.43, 11.20, 10.49.

Morti07 ^{1}H-^{1}H COSY

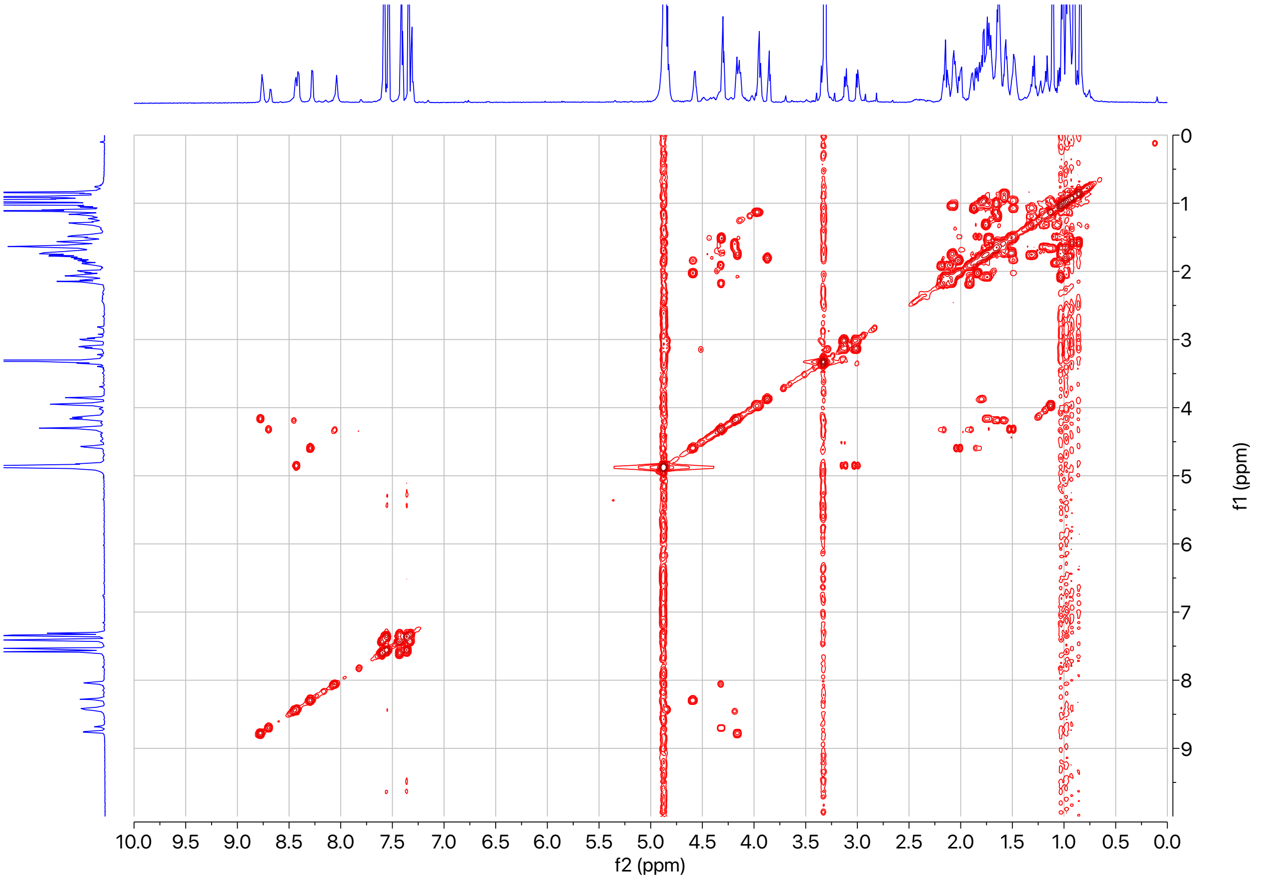

Morti07 ^{1}H-^{13}C HSQC

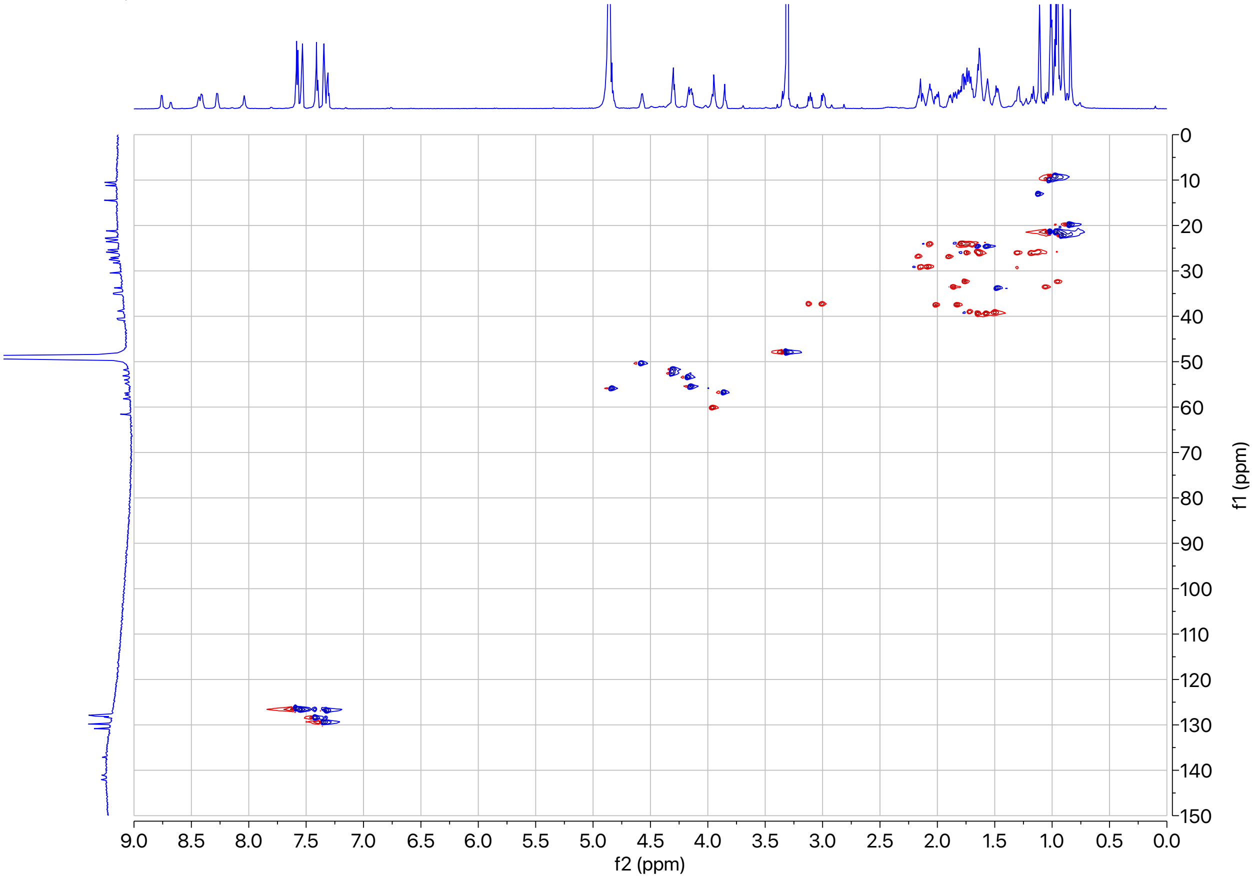

Morti07 ^{1}H-^{13}C HMBC

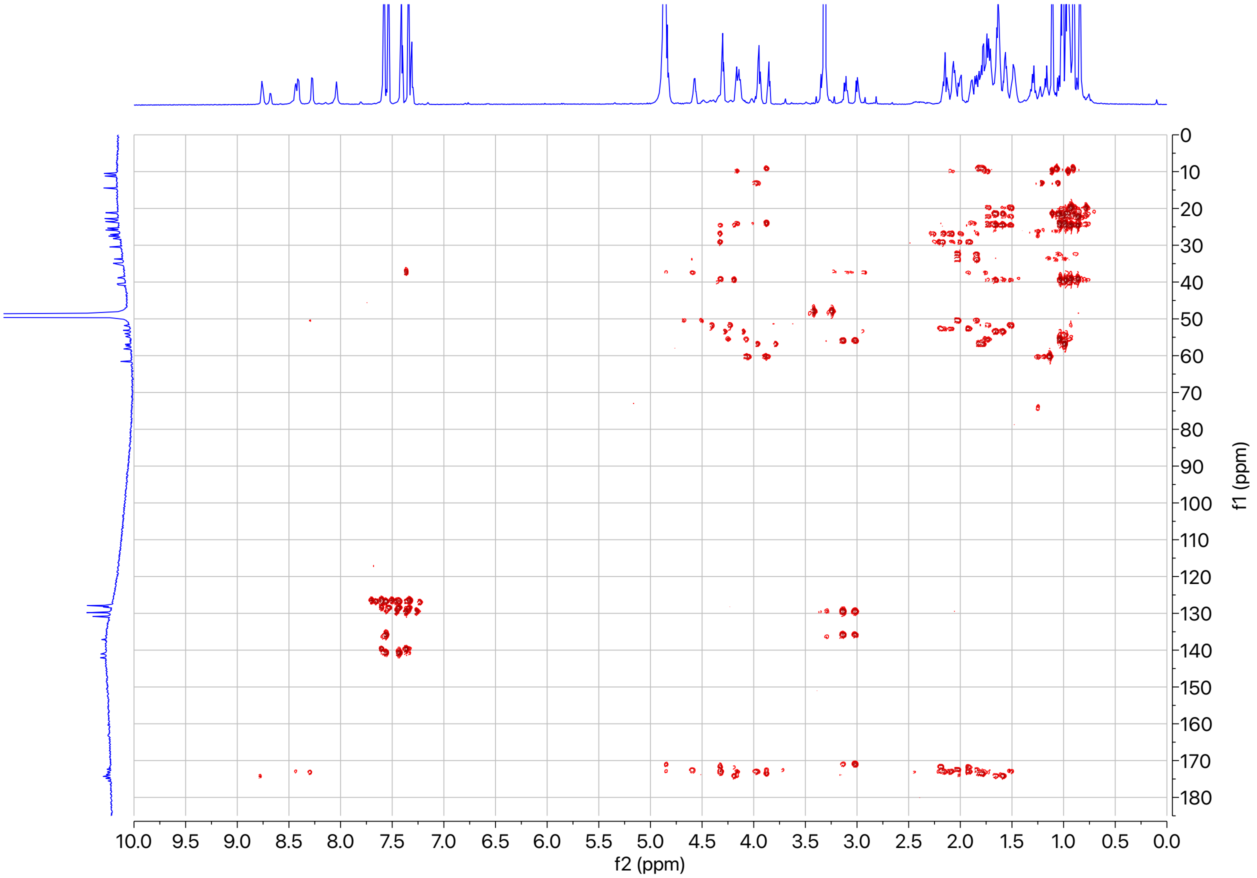

Morti07 ¹H-¹H TOCSY

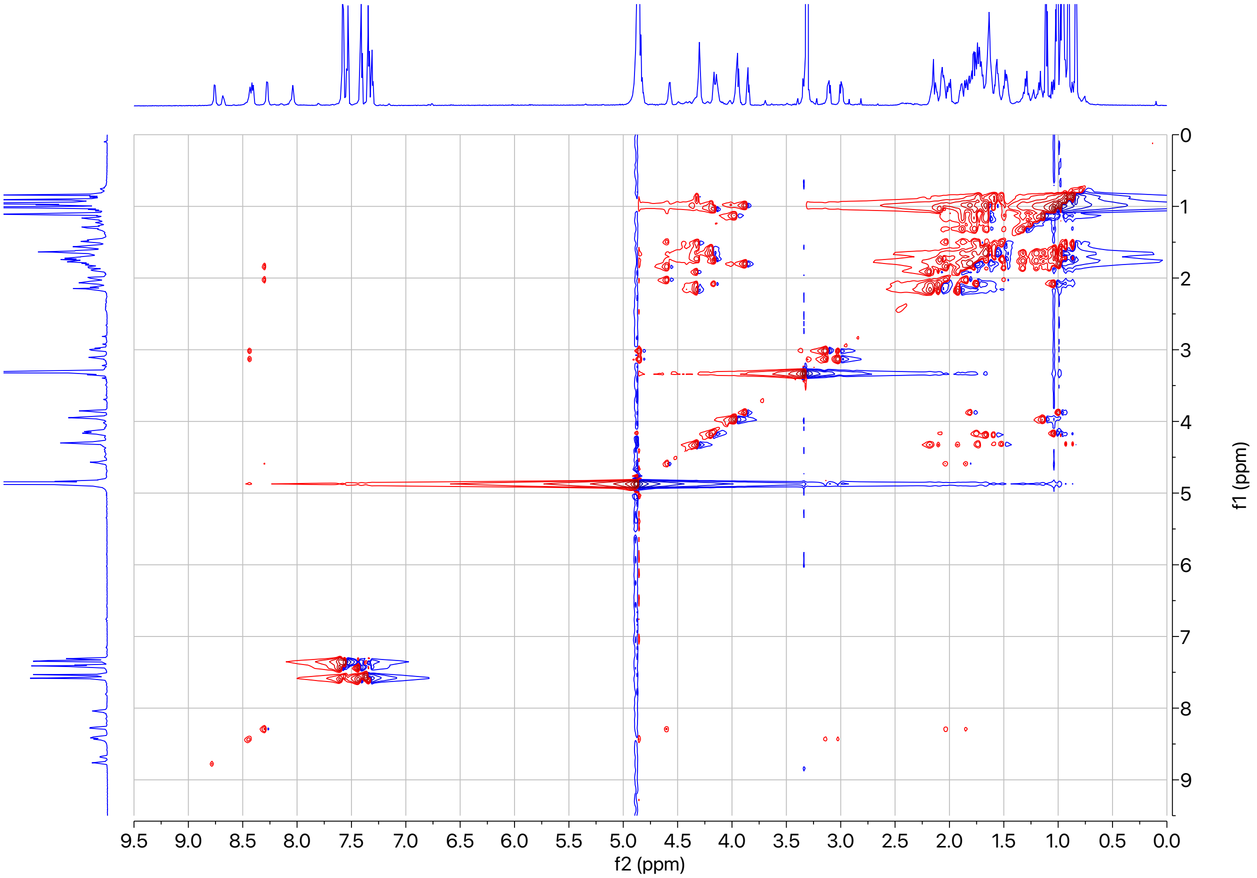

Morti07 ¹H-¹³C HSQC-TOCSY

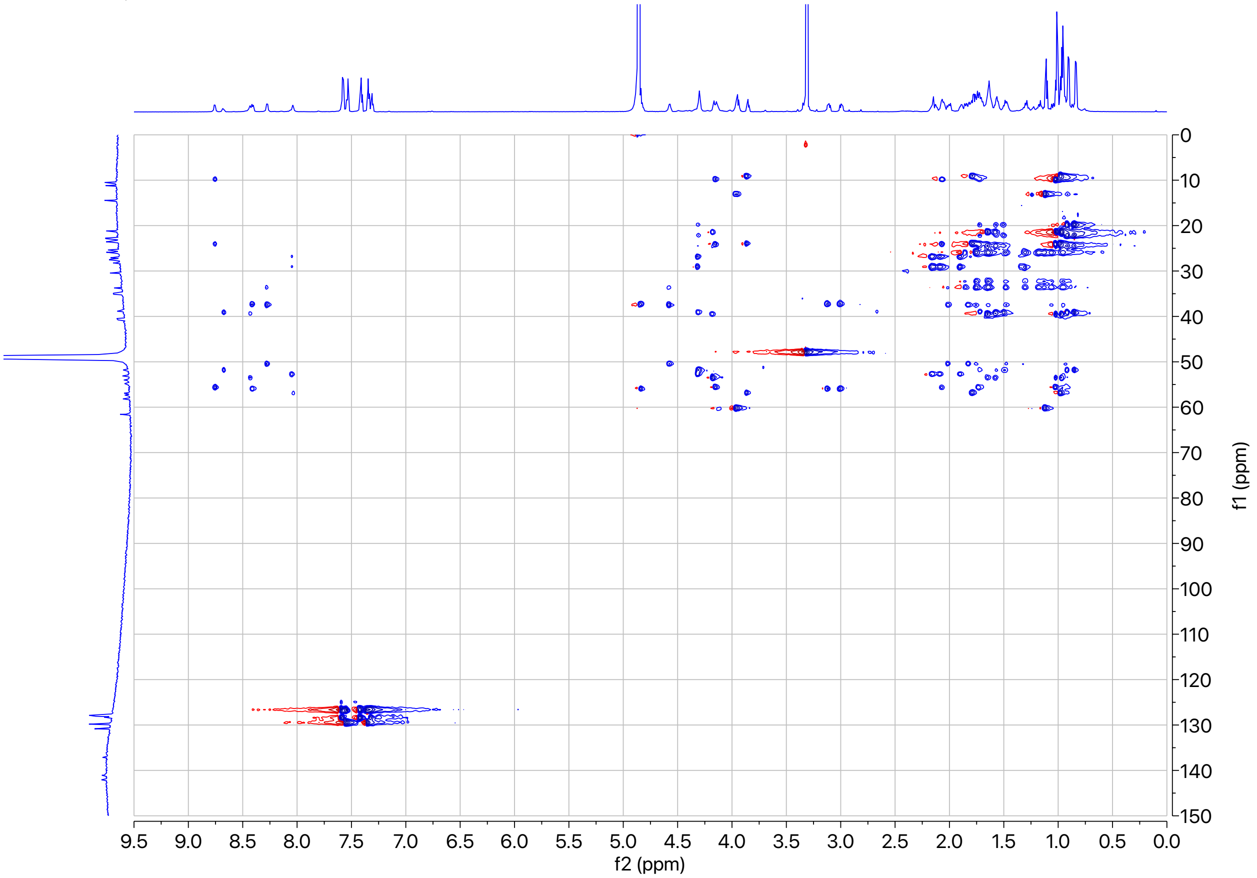

Morti07 epimer

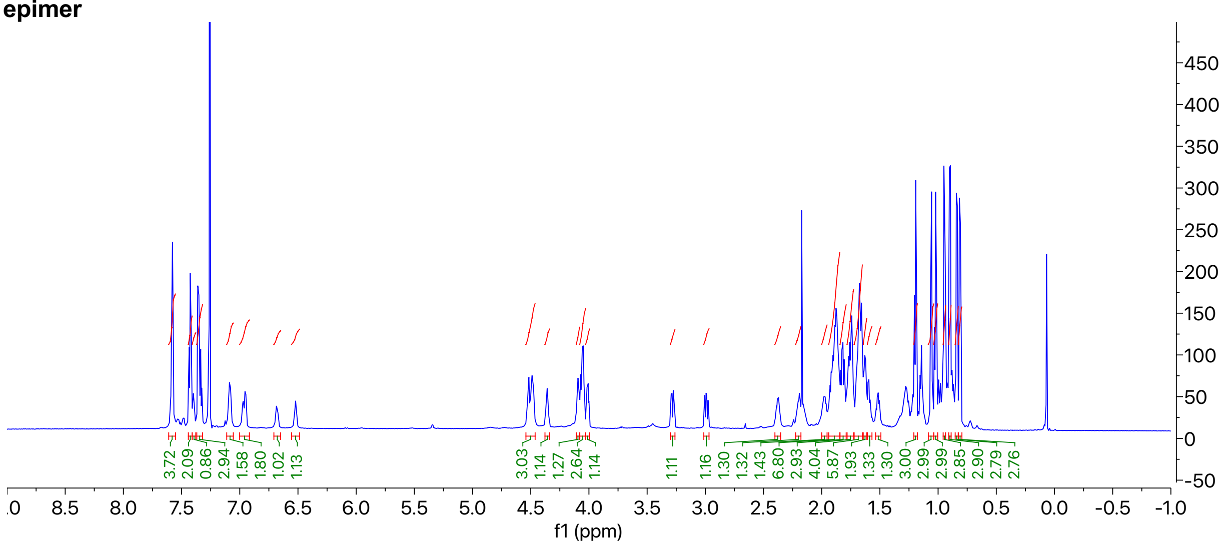

^{1}H NMR (800 MHz, Chloroform-*d*) δ 7.58 (m, 4H), 7.43 (t, *J* = 7.5 Hz, 2H), 7.40 (br, 1H), 7.37 – 7.32 (m, 3H), 7.08 (br, 2H), 7.00 – 6.92 (m, 2H), 6.68 (br, 1H), 6.55 – 6.49 (br, 1H), 4.50 (m, 3H), 4.36 (m, 1H), 4.09 (m, 1H), 4.06 (m, 3H), 4.01 (m, 1H), 3.28 (dd, *J* = 14.1, 5.7 Hz, 1H), 2.99 (dd, *J* = 14.0, 9.0 Hz, 1H), 2.38 (m, 1H), 2.20 (m, 1H), 1.98 (m, 1H), 1.90 (m, 7H), 1.81 (m, 3H), 1.75 (m, 4H), 1.68 (m, 6H), 1.64 – 1.61 (m, 2H), 1.61 – 1.57 (m, 1H), 1.51 (m, 1H), 1.19 (t, *J* = 7.2 Hz, 3H), 1.06 (t, *J* = 7.5 Hz, 3H), 1.02 (t, *J* = 7.4 Hz, 3H), 0.95 (d, *J* = 6.6 Hz, 3H), 0.90 (d, *J* = 6.5 Hz, 3H), 0.84 (d, *J* = 6.6 Hz, 3H), 0.81 (d, *J* = 6.6 Hz, 3H).

Morti07 epimer

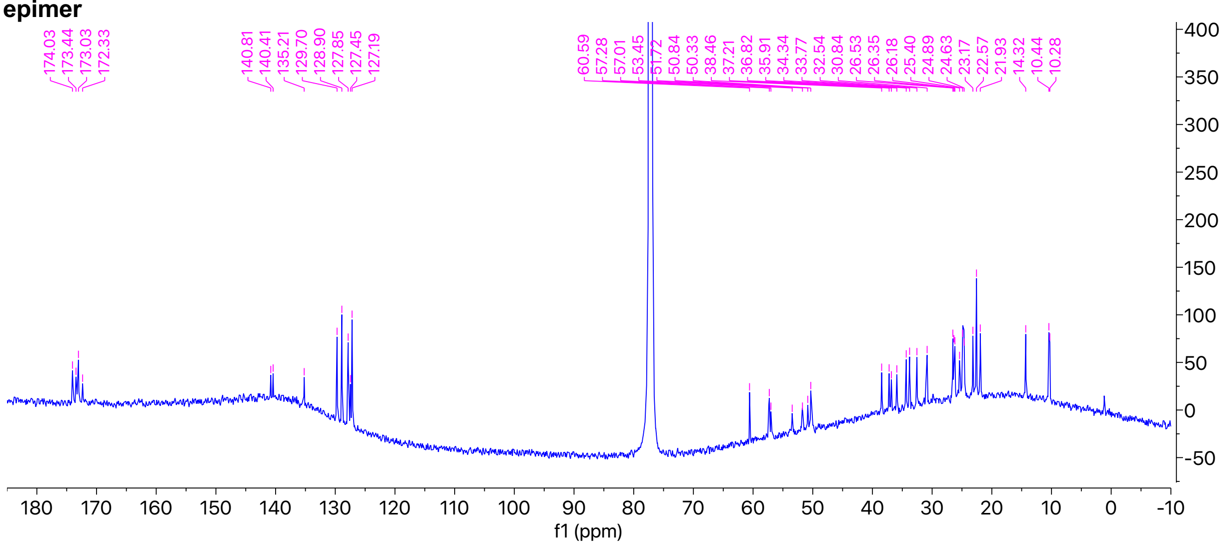

^{13}C NMR (201 MHz, Chloroform-*d*) δ 174.03, 173.44, 173.03, 172.33, 140.81, 140.41, 135.21, 129.70, 128.90, 127.85, 127.45, 127.19, 60.59, 57.28, 57.01, 53.45, 51.72, 50.84, 50.33, 38.46, 37.21, 36.82, 35.91, 34.34, 33.77, 32.54, 30.84, 26.53, 26.35, 26.18, 25.40, 24.89, 24.63, 23.17, 22.57, 21.93, 14.32, 10.44, 10.28.

1E6

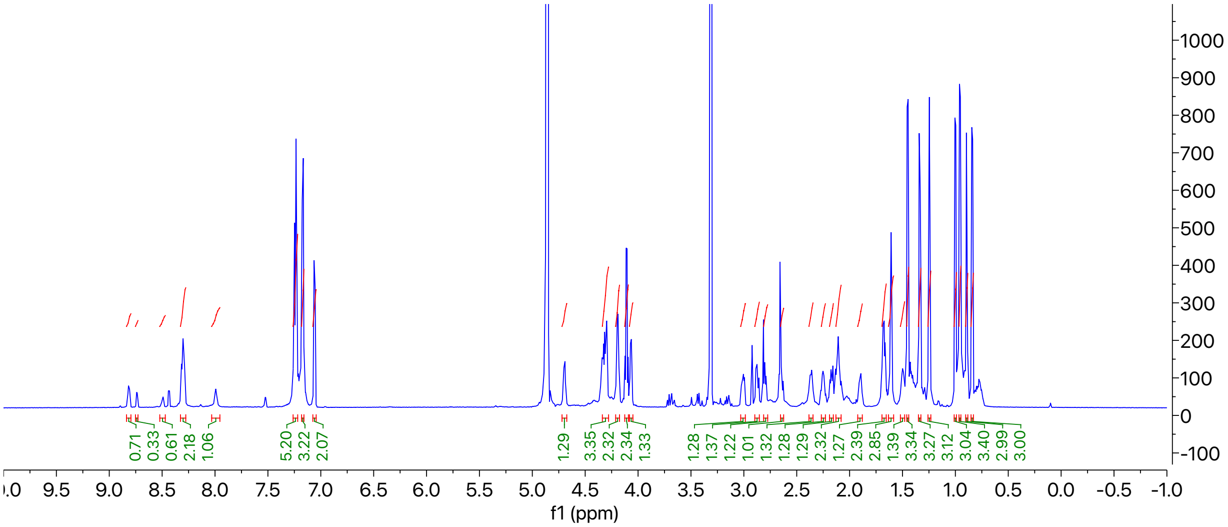

^{1}H NMR (800 MHz, Methanol-d_4) δ 8.84 – 8.80 (br, 1H), 8.74 (d, *J* = 4.4 Hz, 1H), 8.50 (br, 1H), 8.33 – 8.28 (m, 2H), 7.99 (br, 1H), 7.26 – 7.22 (m, 5H), 7.17 (d, *J* = 8.0 Hz, 3H), 7.06 (d, *J* = 8.0 Hz, 2H), 4.70 (q, *J* = 8.2 Hz, 1H), 4.34 – 4.28 (m, 3H), 4.19 (m, 2H), 4.11 (q, *J* = 7.1 Hz, 2H), 4.07 (q, *J* = 7.0 Hz, 1H), 3.03 – 2.99 (m, 1H), 2.87 (dd, *J* = 14.2, 8.9 Hz, 1H), 2.81 – 2.77 (m, 1H), 2.65 – 2.62 (m, 1H), 2.38 – 2.35 (m, 1H), 2.25 (m, 1H), 2.19 – 2.15 (m, 1H), 2.10 (m, 2H), 1.92 – 1.88 (m, 1H), 1.67 (m, 2H), 1.61 (t, *J* = 7.3 Hz, 3H), 1.50 (m, 1H), 1.45 (d, *J* = 7.3 Hz, 3H), 1.34 (d, *J* = 7.0 Hz, 3H), 1.25 (t, *J* = 7.1 Hz, 3H), 1.00 (d, *J* = 6.5 Hz, 3H), 0.96 (d, *J* = 6.6 Hz, 3H), 0.89 (d, *J* = 6.6 Hz, 3H), 0.84 (d, *J* = 6.5 Hz, 3H).

1E6

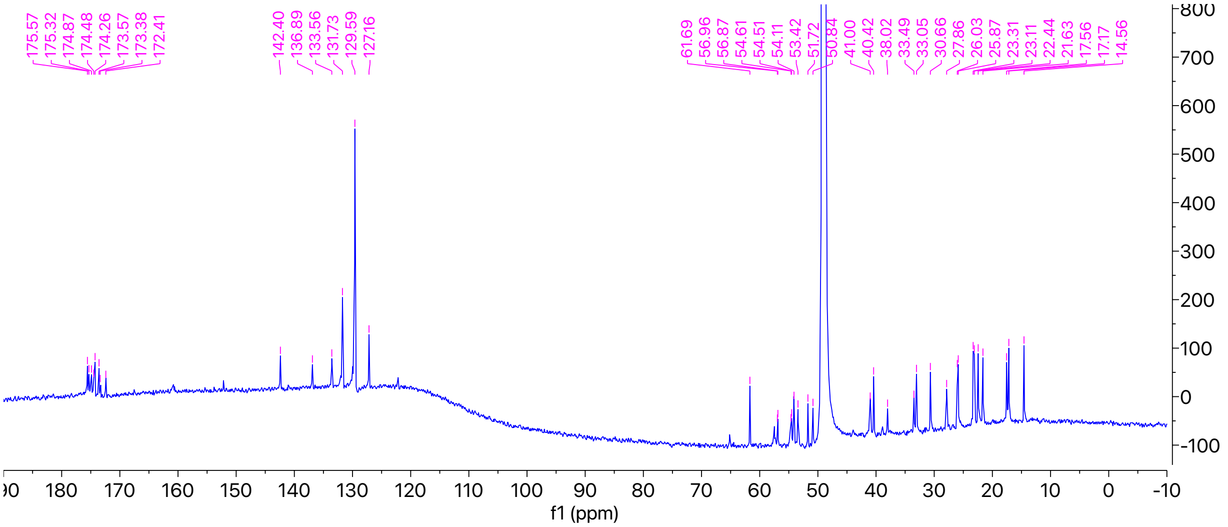

^{13}C NMR (201 MHz, Methanol-d_4) δ 175.57, 175.32, 174.87, 174.48, 174.26, 173.57, 173.38, 172.41, 142.40, 136.89, 133.56, 131.73, 129.59, 127.16, 61.69, 56.96, 56.87, 54.61, 54.51, 54.11, 53.42, 51.72, 50.84, 41.00, 40.42, 38.02, 33.49, 33.05, 30.66, 27.86, 26.03, 25.87, 23.31, 23.11, 22.44, 21.63, 17.56, 17.17, 14.56.

1E6 ^{1}H-^{1}H COSY

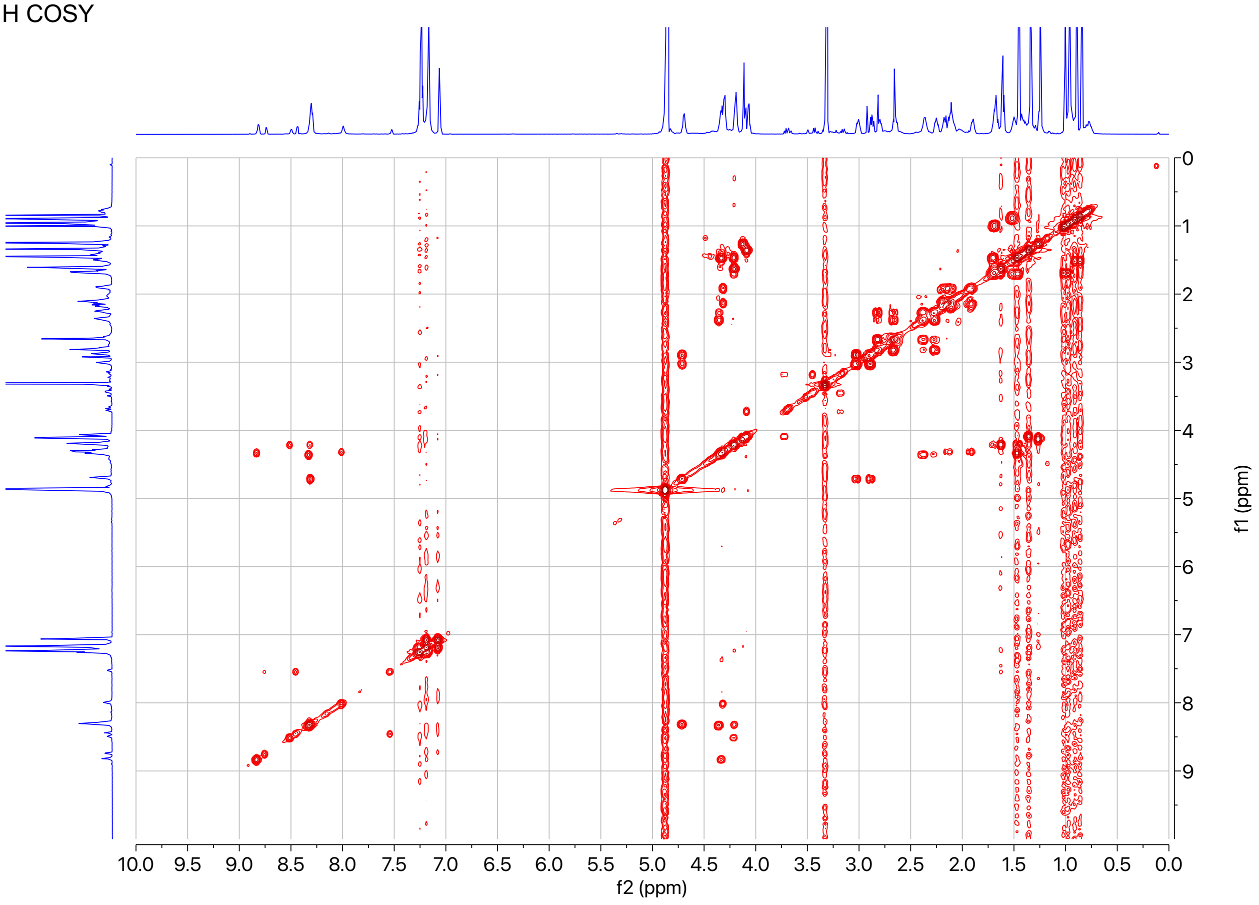

1E6 ^{1}H-^{13}C HSQC

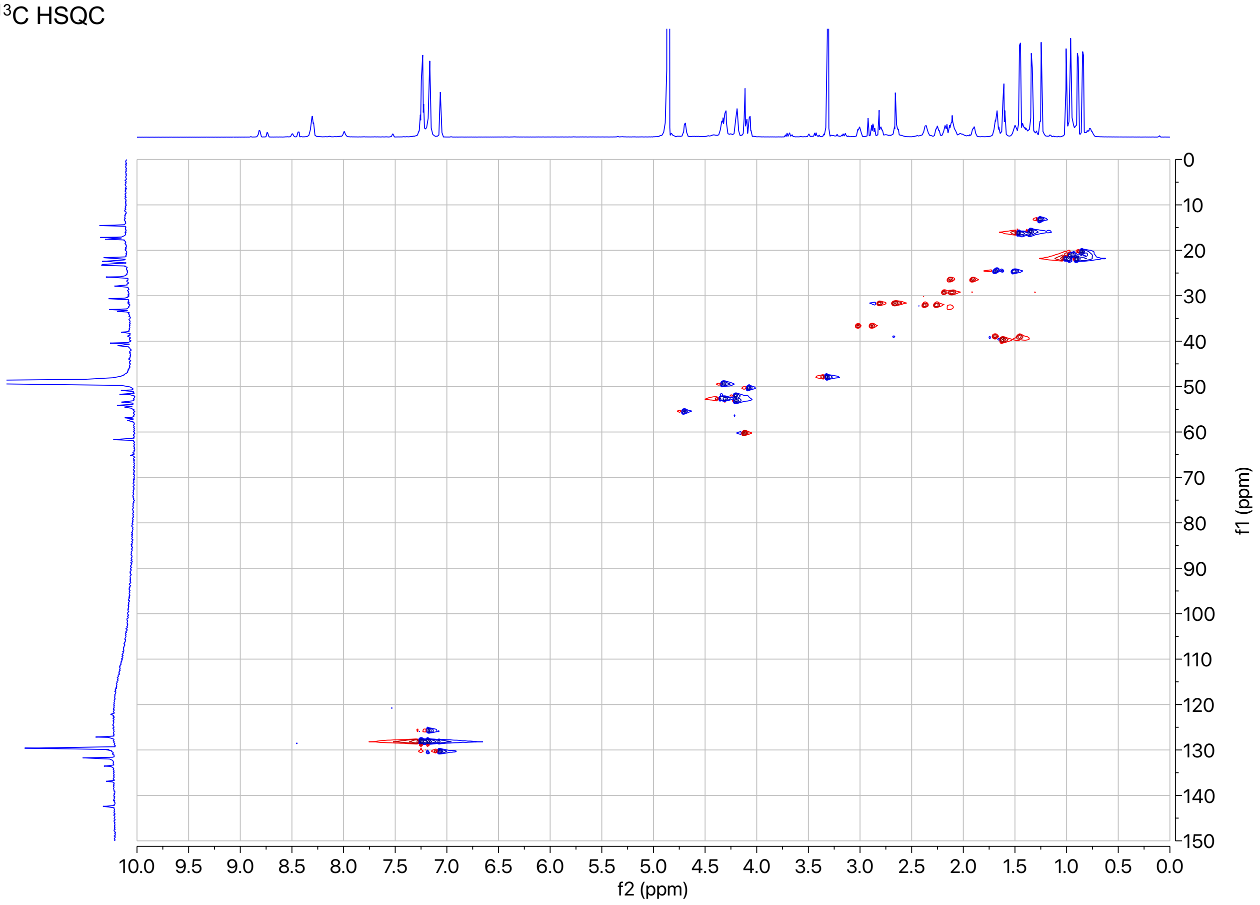

1E6 ^{1}H-^{13}C HMBC

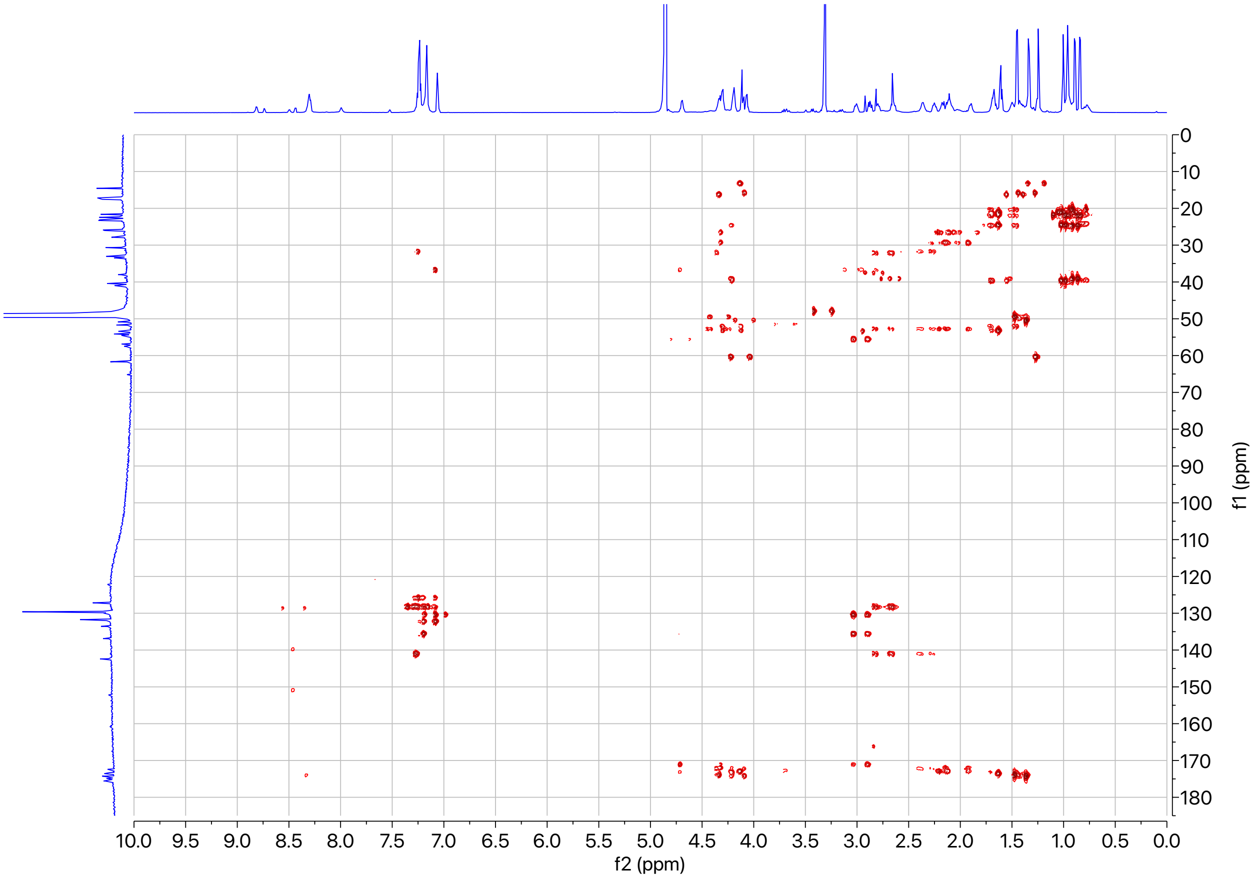

1E6 ^{1}H-^{1}H TOCSY

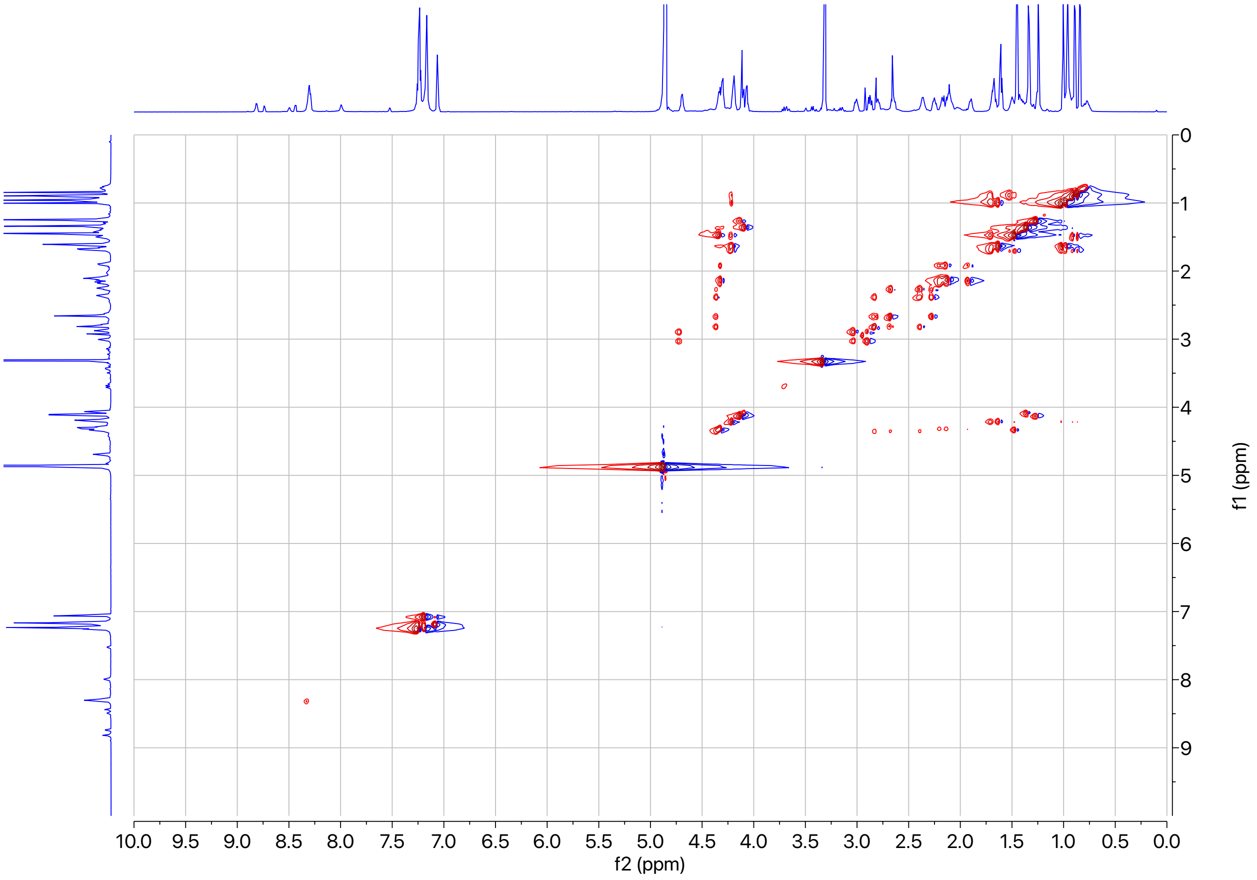

1E6 ^{1}H-^{13}C HSQC-TOCSY

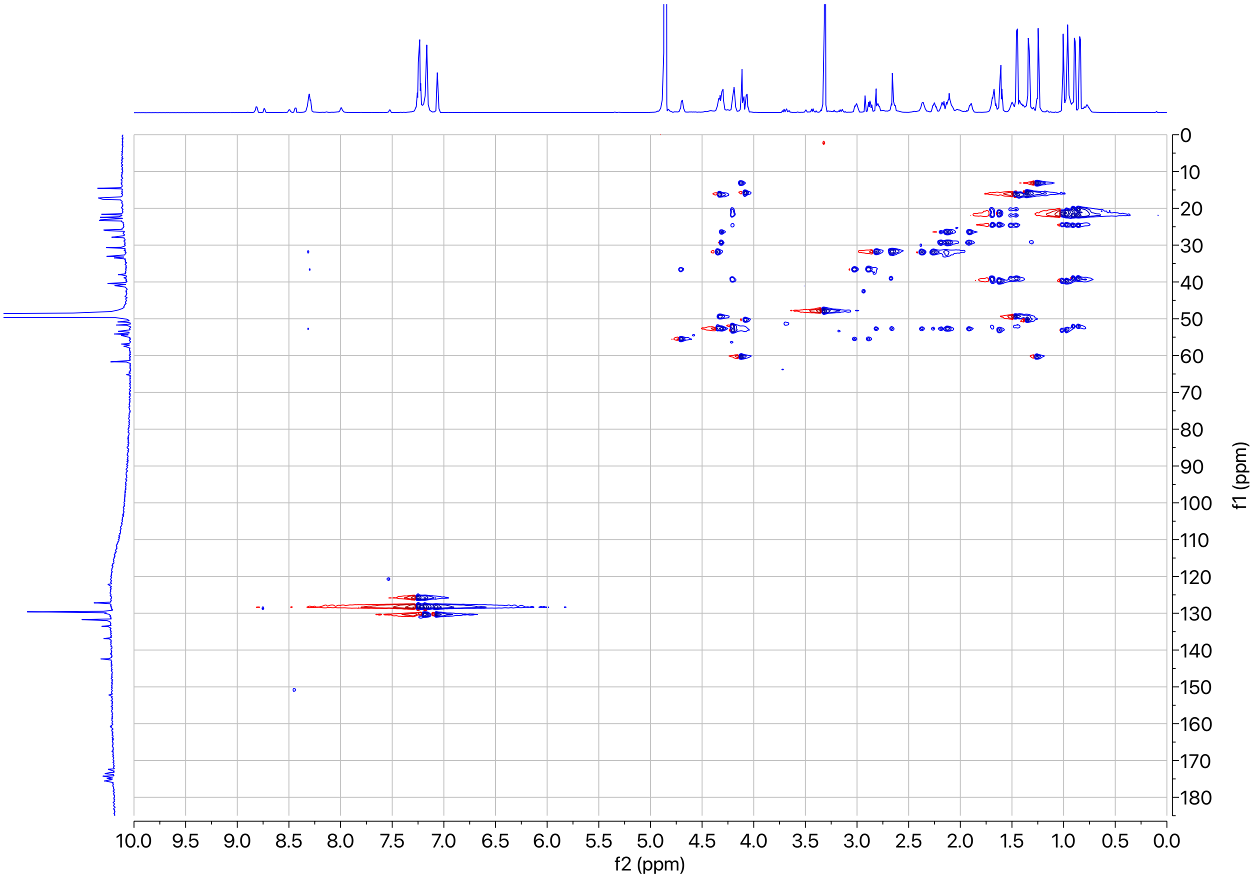

1E6 epimer

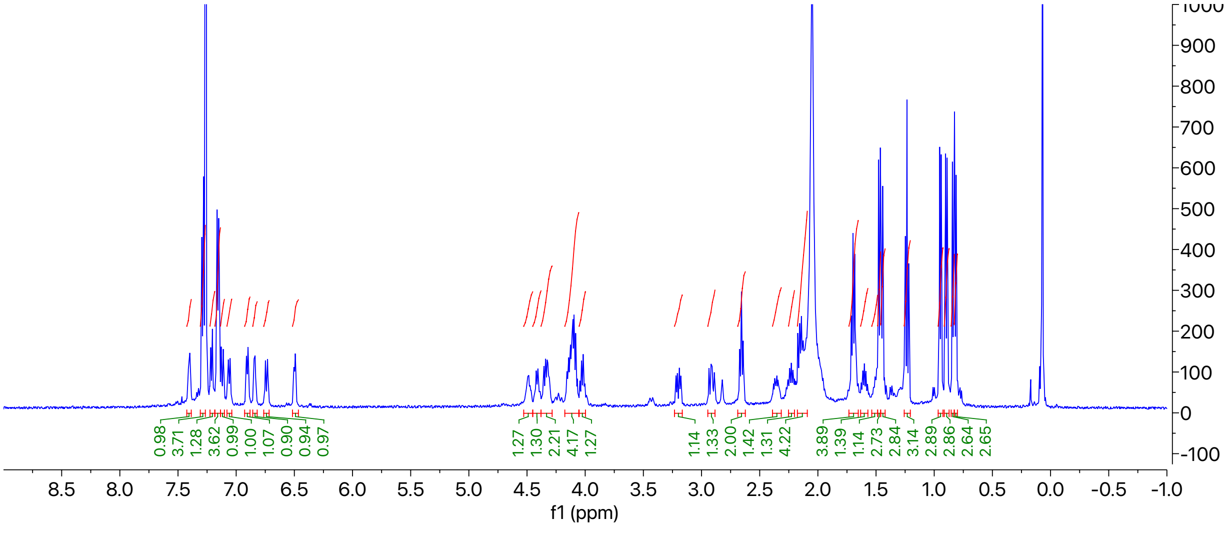

^{1}H NMR (500 MHz, Chloroform-*d*) δ 7.40 (d, *J* = 5.0 Hz, 1H), 7.28 (m, 4H), 7.20 (t, *J* = 7.4 Hz, 1H), 7.18 – 7.14 (m, 4H), 7.12 (d, *J* = 8.9 Hz, 1H), 7.06 (d, *J* = 8.0 Hz, 1H), 6.90 (d, *J* = 6.1 Hz, 1H), 6.84 (d, *J* = 4.5 Hz, 1H), 6.74 (d, *J* = 9.2 Hz, 1H), 6.50 (d, *J* = 5.1 Hz, 1H), 4.49 (m, 1H), 4.41 (q, *J* = 7.8 Hz, 1H), 4.33 (m, 2H), 4.11 (m, 4H), 4.02 (q, *J* = 7.3 Hz, 1H), 3.20 (dd, *J* = 14.2, 5.6 Hz, 1H), 2.91 (dd, *J* = 14.1, 8.9 Hz, 1H), 2.66 (t, *J* = 7.6 Hz, 2H), 2.35 (m, 1H), 2.25 – 2.20 (m, 1H), 2.18 – 2.09 (m, 4H), 1.69 (m, 4H), 1.60 (m, 1H), 1.50 (m, 1H), 1.47 (d, *J* = 7.3 Hz, 3H), 1.45 (d, *J* = 7.2 Hz, 3H), 1.23 (t, *J* = 7.1 Hz, 3H), 0.95 (d, *J* = 6.5 Hz, 3H), 0.90 (d, *J* = 6.5 Hz, 3H), 0.84 (d, *J* = 6.6 Hz, 3H), 0.82 (d, *J* = 6.5 Hz, 3H).

2B5

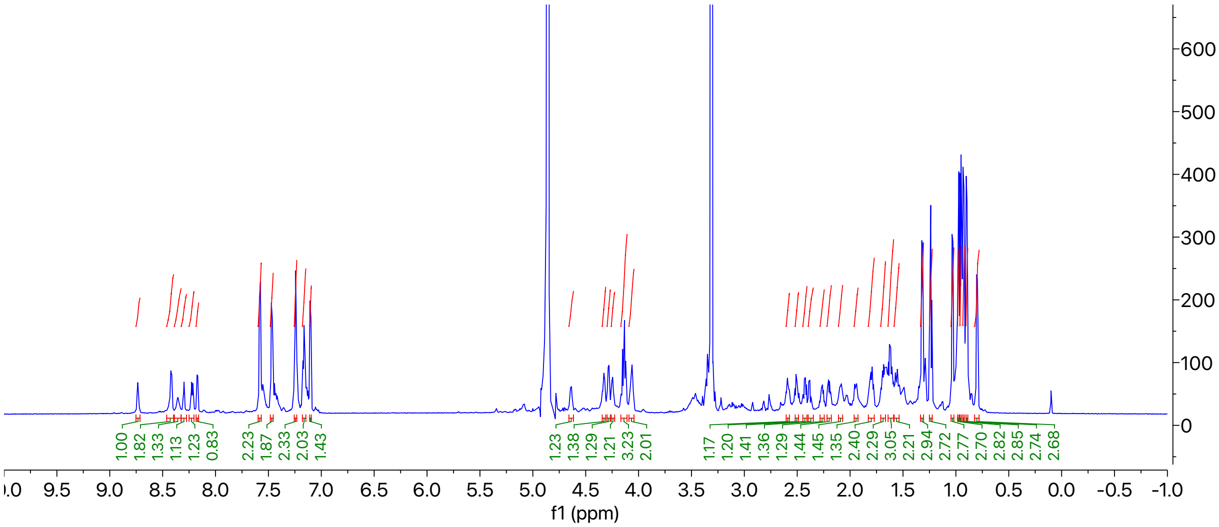

^{1}H NMR (800 MHz, Methanol-d_4) δ 8.73 (d, J = 7.3 Hz, 1H), 8.42 (d, J = 8.0 Hz, 2H), 8.36 (br, 1H), 8.32 – 8.27 (br, 1H), 8.22 (d, J = 9.4 Hz, 1H), 8.17 (d, J = 5.5 Hz, 1H), 7.58 (d, J = 7.9 Hz, 2H), 7.47 (d, J = 7.9 Hz, 2H), 7.24 (t, J = 7.2 Hz, 2H), 7.16 (t, J = 7.4 Hz, 2H), 7.10 (d, J = 7.6 Hz, 1H), 4.64 (m, 1H), 4.33 (m, 1H), 4.28 (q, J = 6.2 Hz, 1H), 4.25 (m, 1H), 4.14 (m, 3H), 4.06 (m, 2H), 2.59 (m, 1H), 2.52 – 2.49 (m, 1H), 2.45 – 2.41 (m, 1H), 2.38 (m, 1H), 2.28 – 2.24 (m, 1H), 2.22 – 2.18 (m, 1H), 2.09 (m, 1H), 1.95 (m, 1H), 1.83 – 1.77 (m, 2H), 1.69 (m, 2H), 1.61 (m, 3H), 1.58 – 1.54 (m, 2H), 1.32 (d, J = 7.0 Hz, 3H), 1.24 (t, J = 7.1, 3H), 1.03 (d, J = 6.5 Hz, 3H), 0.98 (d, J = 6.4 Hz, 3H), 0.96 (d, J = 6.8 Hz, 3H), 0.93 (d, J = 6.8 Hz, 3H), 0.90 (d, J = 6.5 Hz, 3H), 0.80 (d, J = 6.7 Hz, 3H).

2B5

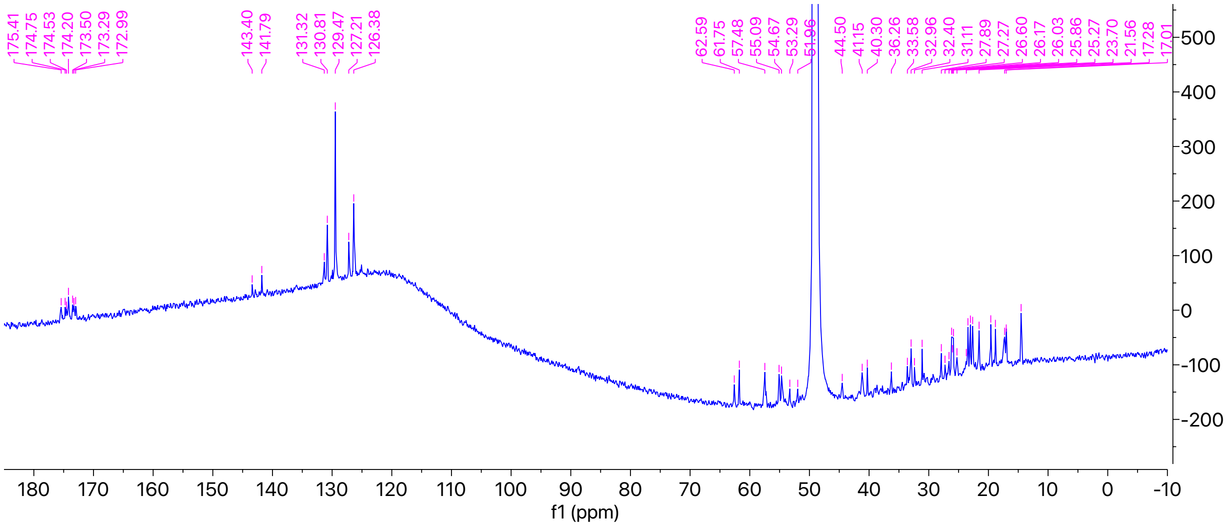

^{13}C NMR (201 MHz, Methanol-d_4) δ 175.41, 174.75, 174.53, 174.20, 173.50, 173.29, 172.99, 143.40, 141.79, 131.32, 130.81, 129.47, 127.21, 126.38, 62.59, 61.75, 57.48, 55.09, 54.67, 53.29, 51.96, 44.50, 41.15, 40.30, 36.26, 33.58, 32.96, 32.40, 31.11, 27.89, 27.27, 26.60, 26.17, 26.03, 25.86, 25.27, 23.70, 23.43, 23.02, 22.64, 21.56, 19.60, 18.83, 17.28, 17.01, 14.52.

2B5 ^{1}H-^{1}H COSY

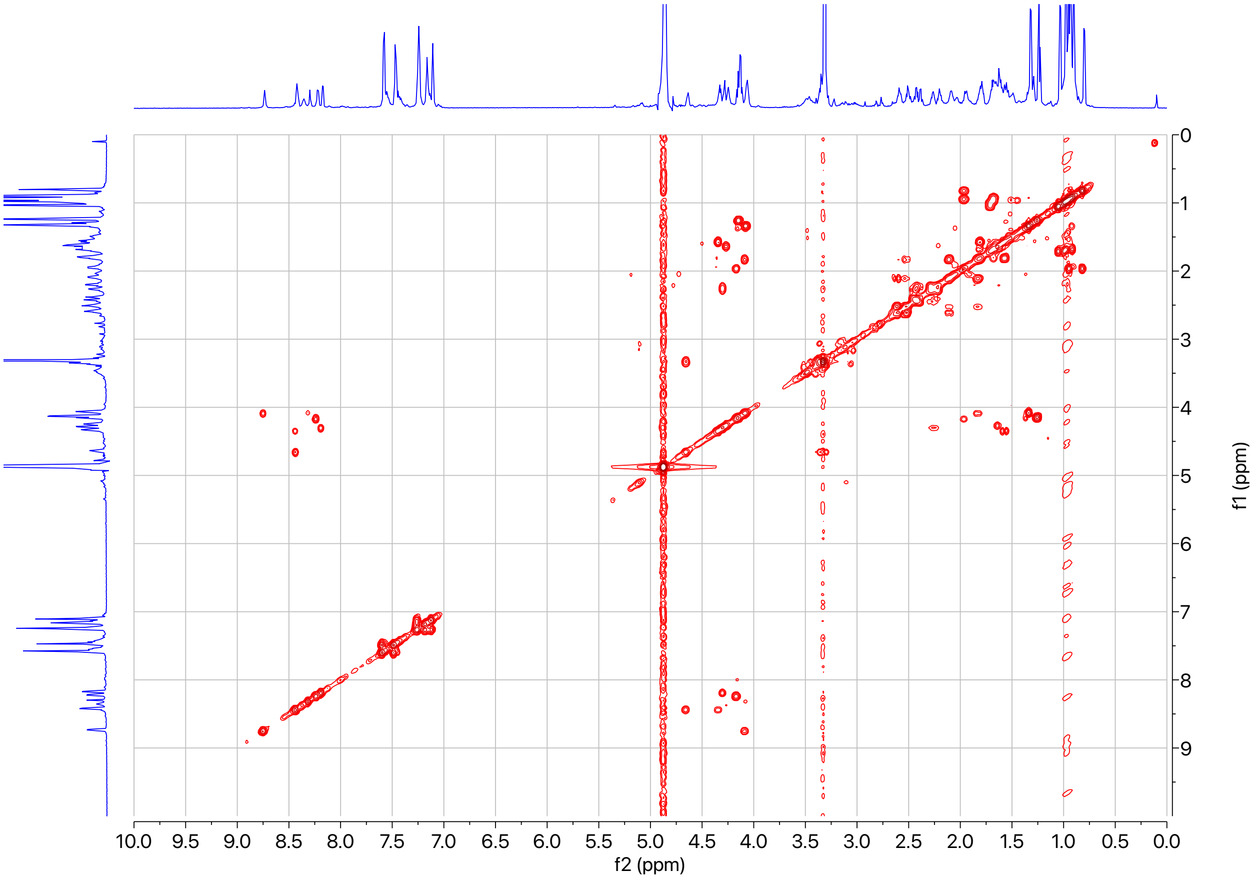

2B5 ^{1}H-^{13}C HSQC

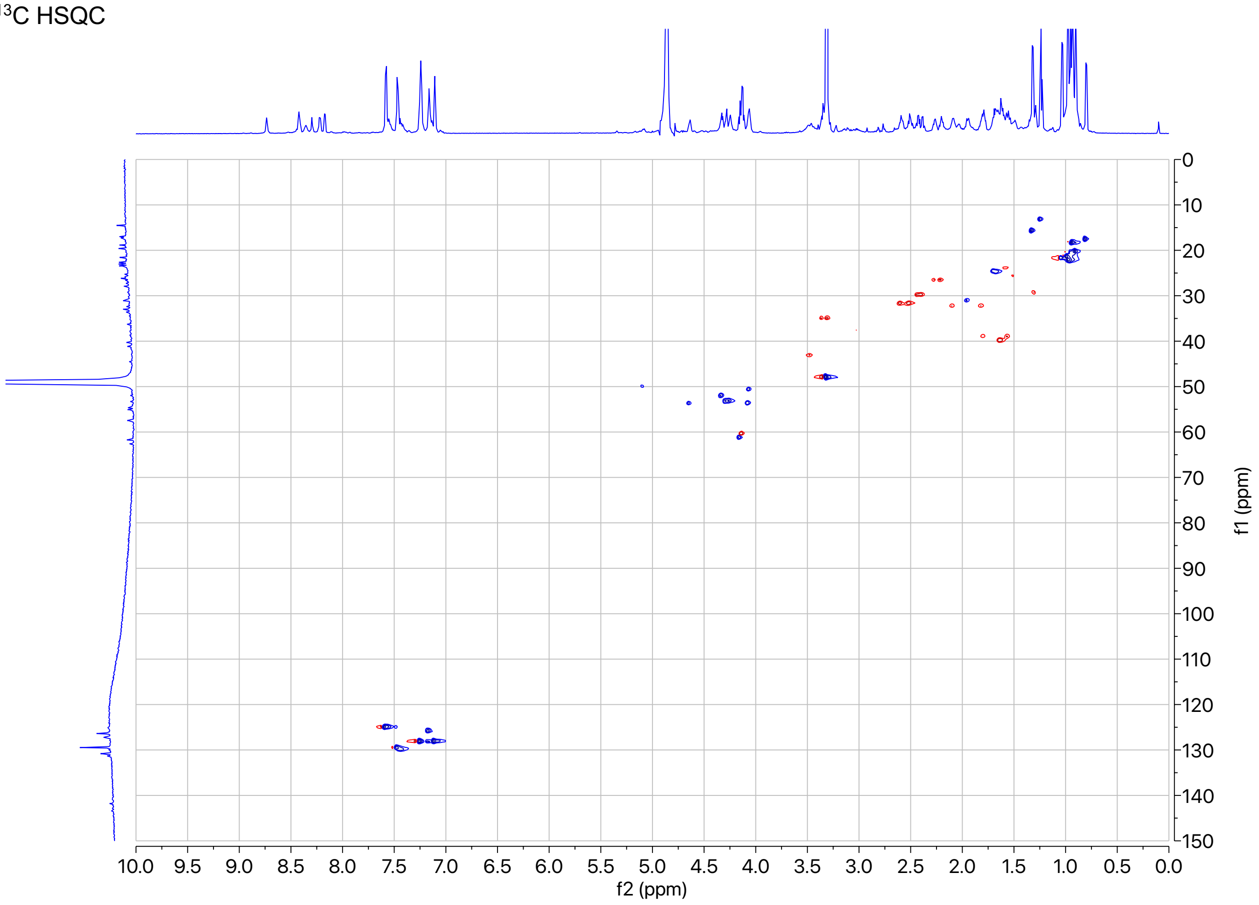

2B5 ^{1}H-^{13}C HMBC

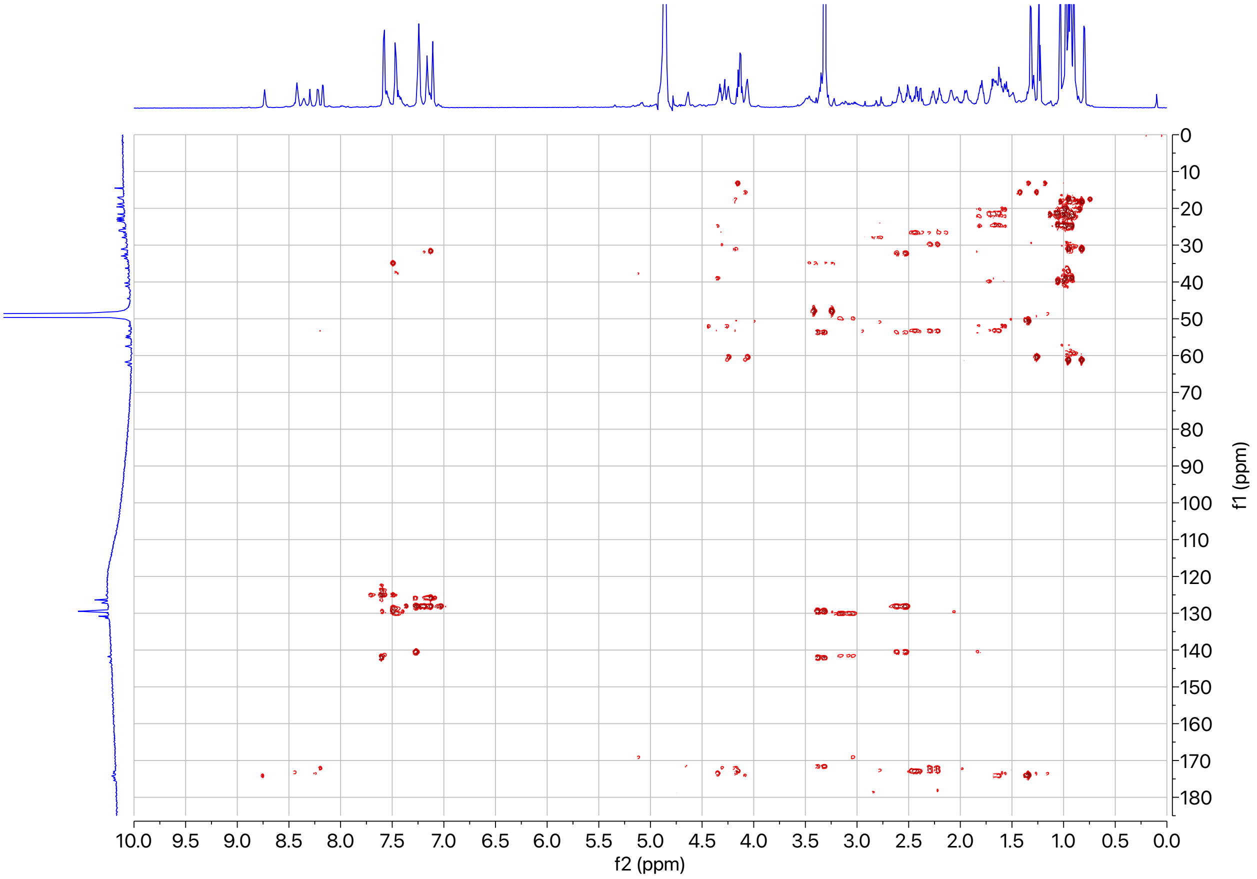

2B5 ^{1}H-^{1}H TOCSY

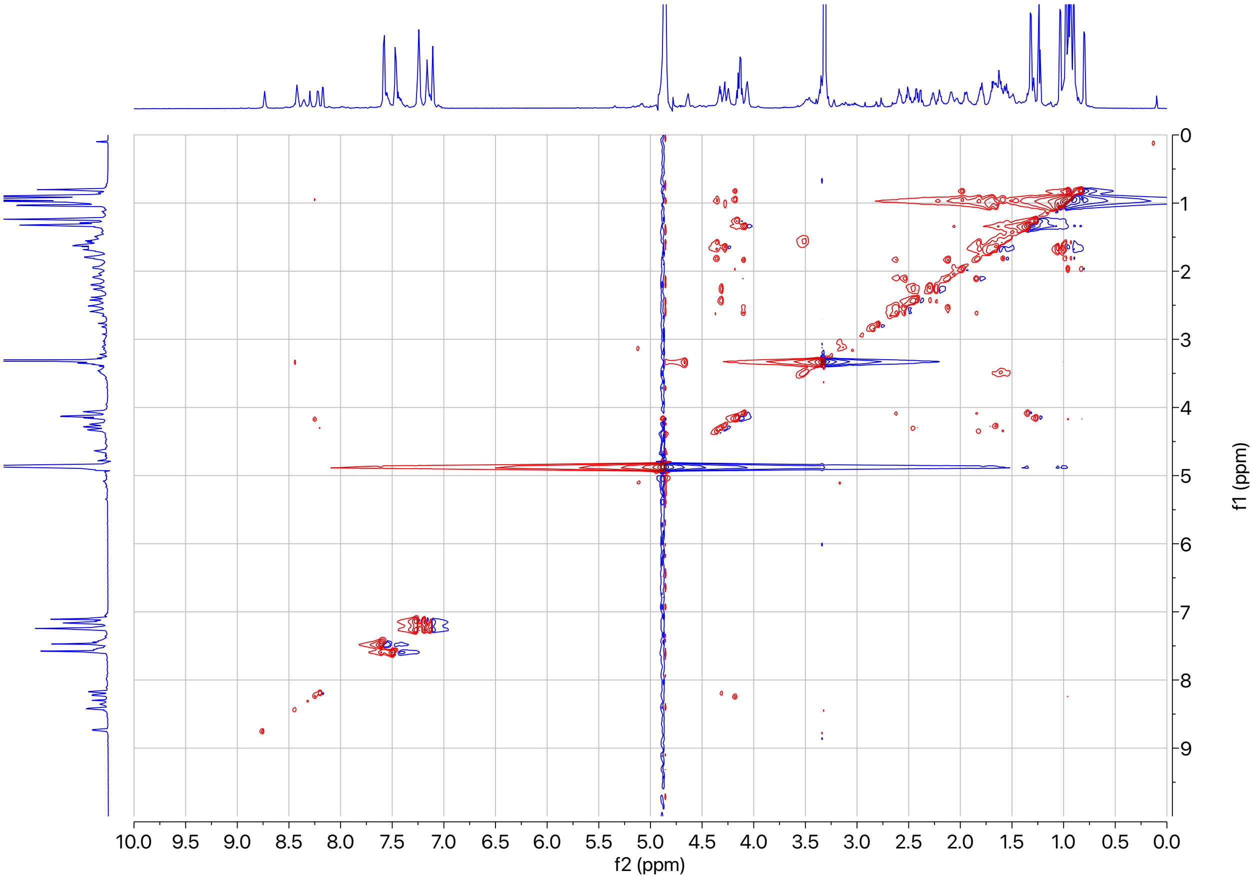

2B5 ^{1}H-^{13}C HSQC-TOCSY

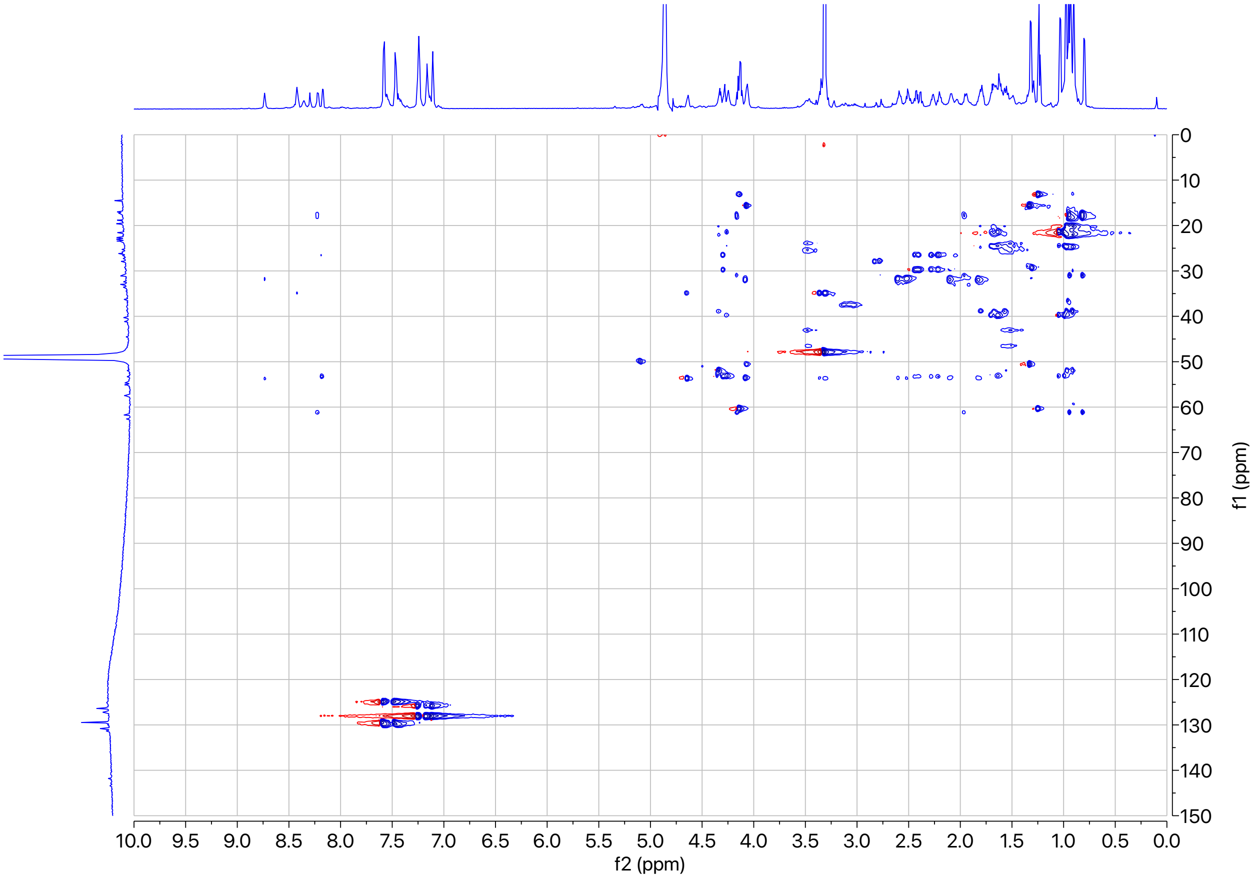

7-A[17]

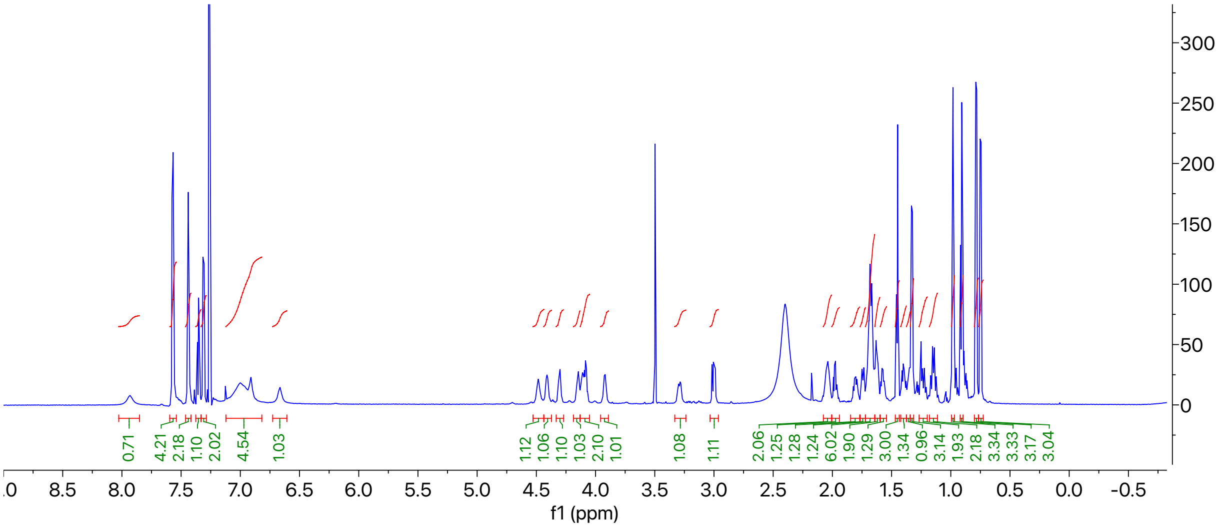

^{1}H NMR (800 MHz, Chloroform-*d*) δ 7.93 (br, 1H), 7.60 – 7.54 (m, 4H), 7.44 (t, *J* = 7.8 Hz, 2H), 7.38 – 7.33 (m, 1H), 7.31 (d, *J* = 7.8 Hz, 2H), 7.12-6.82 (br, 5H), 6.67 (br, 1H), 4.48 (m, 1H), 4.41 (m, 1H), 4.31 (m, 1H), 4.15 (m, 1H), 4.09 (m, 2H), 3.92 (m, 1H), 3.29 (m, 1H), 3.00 (dd, *J* = 14.2, 9.1 Hz, 1H), 2.04 (m, 2H), 1.97 (m, 1H), 1.85 – 1.77 (m, 1H), 1.74 (d, *J* = 13.1 Hz, 1H), 1.72 – 1.64 (m, 6H), 1.62 (m, 2H), 1.57 (m, 1H), 1.45 (d, *J* = 7.2 Hz, 3H), 1.40 (m, 1H), 1.35 (m, 1H), 1.33 (d, *J* = 7.0 Hz, 3H), 1.23 (m, 2H), 1.15 (m, 2H), 0.98 (t, *J* = 7.4 Hz, 3H), 0.91 (t, *J* = 7.4 Hz, 3H), 0.78 (d, *J* = 6.6 Hz, 3H), 0.75 (d, *J* = 6.6 Hz, 3H).

7-Hse^{2}A^7

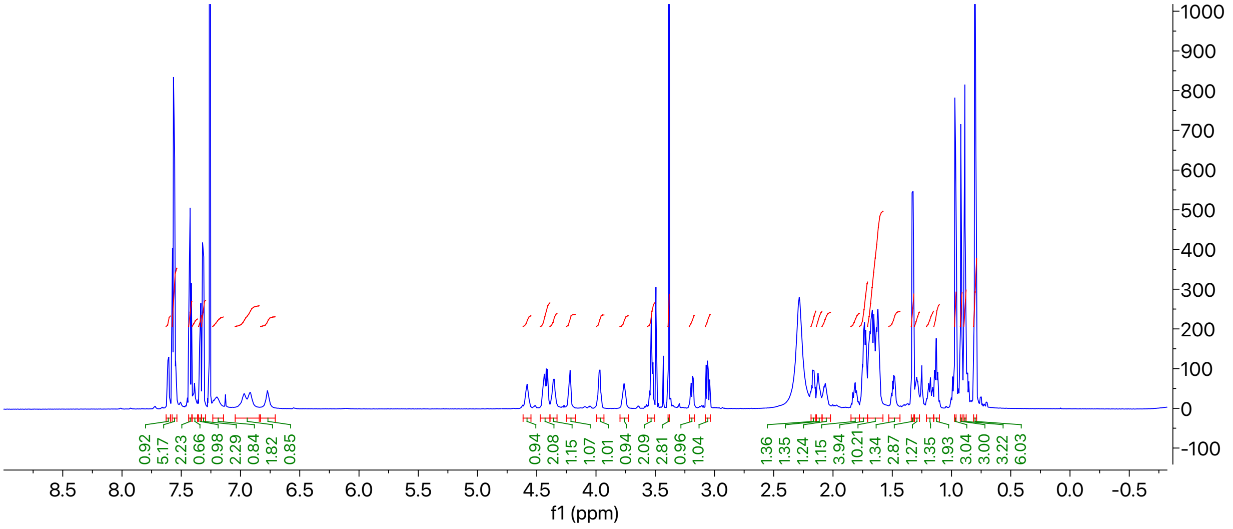

^{1}H NMR (800 MHz, Chloroform-*d*) δ 7.61 (d, *J* = 5.7 Hz, 1H), 7.58 – 7.54 (m, 5H), 7.44 – 7.41 (m, 2H), 7.41 – 7.36 (br, 1H), 7.35 – 7.33 (m, 1H), 7.33 – 7.29 (m, 2H), 7.23 – 7.14 (br, 1H), 6.94 (m, 2H), 6.77 (br, 1H), 4.58 (m, 1H), 4.42 (m, 2H), 4.36 (m, 1H), 4.25 – 4.17 (m, 1H), 3.97 (m, 1H), 3.76 (m, 1H), 3.53 (m, 2H), 3.39 (s, 3H), 3.19 (dd, *J* = 14.1, 5.7 Hz, 1H), 3.06 (dd, *J* = 14.1, 8.2 Hz, 1H), 2.17 (m, 1H), 2.14 – 2.09 (m, 1H), 2.07 (m, 1H), 1.81 (m, 1H), 1.73 (m, 4H), 1.71 – 1.58 (m, 10H), 1.53 – 1.43 (m, 1H), 1.33 (d, *J* = 7.0 Hz, 3H), 1.31 – 1.27 (m, 1H), 1.21 – 1.15 (m, 1H), 1.13 (m, 2H), 0.97 (d, *J* = 6.1 Hz, 3H), 0.92 (d, *J* = 6.2 Hz, 3H), 0.88 (t, *J* = 7.6 Hz, 3H), 0.80 (d, *J* = 6.6 Hz, 6H).

7-S^2

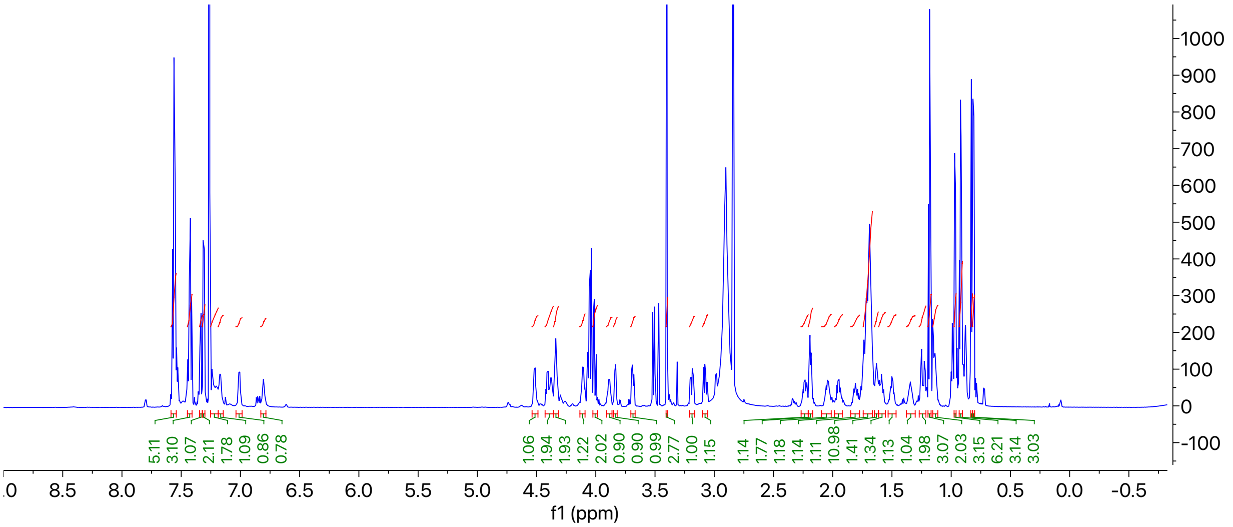

^{1}H NMR (800 MHz, Chloroform-*d*) δ 7.56 (m, 5H), 7.43 (m, 3H), 7.35 – 7.32 (m, 1H), 7.31 (d, *J* = 7.9 Hz, 2H), 7.25 – 7.19 (br, 2H), 7.17 (br, 1H), 7.01 (d, *J* = 7.2 Hz, 1H), 6.80 (br, 1H), 4.52 (m, 1H), 4.40 (m, 2H), 4.34 (m, 2H), 4.13 – 4.09 (m, 1H), 4.01 (dd, *J* = 13.3, 4.0 Hz, 2H), 3.89 (m, 1H), 3.84 (dd, *J* = 9.8, 4.3 Hz, 1H), 3.69 (dd, *J* = 9.8, 5.1 Hz, 1H), 3.40 (s, 3H), 3.19 (dd, *J* = 14.1, 6.5 Hz, 1H), 3.08 (dd, *J* = 14.0, 8.1 Hz, 1H), 2.27 – 2.21 (m, 1H), 2.19 (m, 2H), 2.04 (m, 1H), 1.98 – 1.92 (m, 1H), 1.85 – 1.77 (m, 1H), 1.74 – 1.67 (m, 11H), 1.63 (m, 1H), 1.59 (m, 1H), 1.50 (m, 1H), 1.38 – 1.30 (m, 1H), 1.27 – 1.21 (m, 2H), 1.18 (t, *J* = 7.1 Hz, 3H), 1.16 – 1.11 (m, 2H), 0.97 (d, *J* = 5.8 Hz, 3H), 0.91 (m, 6H), 0.83 (d, *J* = 6.5 Hz, 3H), 0.81 (d, *J* = 6.6 Hz, 3H).

7-A^{6}

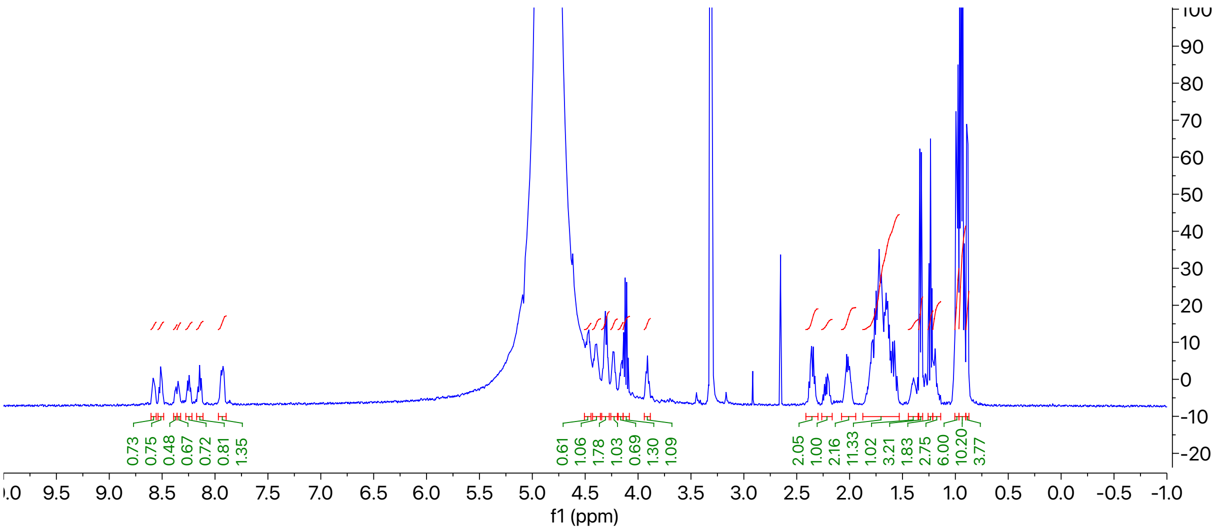

^{1}H NMR (500 MHz, Methanol-d_4) δ 8.61 – 8.56 (m, 1H), 8.51 (t, *J* = 7.5 Hz, 1H), 8.37 (br, 1H), 8.35 (br, 1H), 8.24 (t, *J* = 7.3 Hz, 1H), 8.15 (t, *J* = 7.5 Hz, 1H), 7.97 – 7.89 (m, 1H), 4.51 – 4.44 (m, 1H), 4.43 – 4.36 (m, 1H), 4.31 (m, 2H), 4.26 – 4.20 (m, 1H), 4.19 – 4.14 (m, 1H), 4.13 (q, *J* = 7.2 Hz,1H), 3.92 (m, 1H), 2.35 (m, 2H), 2.22 (m, 1H), 2.01 (m, 2H), 1.87 – 1.53 (m, 11H), 1.39 (m, 1H), 1.33 (d, *J* = 7.1 Hz, 3H), 1.23 (t, *J* = 7.2 Hz, 2H), 1.21 – 1.14 (m, 3H), 1.00 – 0.97 (m, 6H), 0.96 (m, 10H), 0.89 (d, *J* = 6.2 Hz, 4H).

7-A^{6} epimer

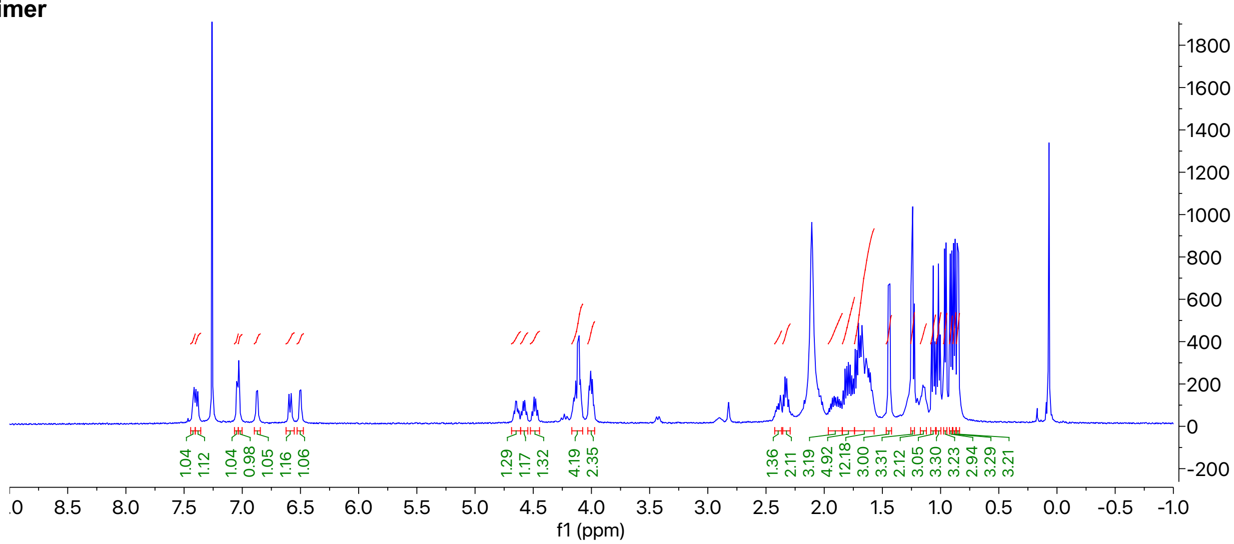

^{1}H NMR (500 MHz, Chloroform-*d*) δ 7.42 (d, *J* = 4.2 Hz, 1H), 7.39 (d, *J* = 8.1 Hz, 1H), 7.04 (d, *J* = 4.4 Hz, 1H), 7.03 (s, 1H), 6.87 (d, *J* = 5.2 Hz, 1H), 6.59 (d, *J* = 9.7 Hz, 1H), 6.50 (d, *J* = 5.5 Hz, 1H), 4.64 (m, 1H), 4.57 (m, 1H), 4.52 – 4.45 (m, 1H), 4.17 – 4.08 (m, 4H), 4.01 (m, 2H), 2.43 – 2.37 (m, 1H), 2.33 (m, 2H), 1.96 – 1.85 (m, 3H), 1.84 – 1.74 (m, 5H), 1.74 – 1.57 (m, 12H), 1.44 (d, *J* = 7.3 Hz, 3H), 1.30 – 1.27 (m, 2H), 1.24 (t, *J* = 7.1 Hz, 3H), 1.17 – 1.12 (m, 2H), 1.06 (t, *J* = 7.4 Hz, 3H), 1.02 (t, *J* = 7.4 Hz, 3H), 0.96 (d, *J* = 6.5 Hz, 3H), 0.91 (d, *J* = 6.6 Hz, 3H), 0.88 (d, *J* = 6.5 Hz, 3H), 0.85 (d, *J* = 6.5 Hz, 3H).

LC-MS Spectra

Morti01

ELSD
morti01_01.datx 2024.08.14 12:32:27 5 mins 200-2000 positive mode;
ESI + Settings for tune mix using source type ESI Positive.

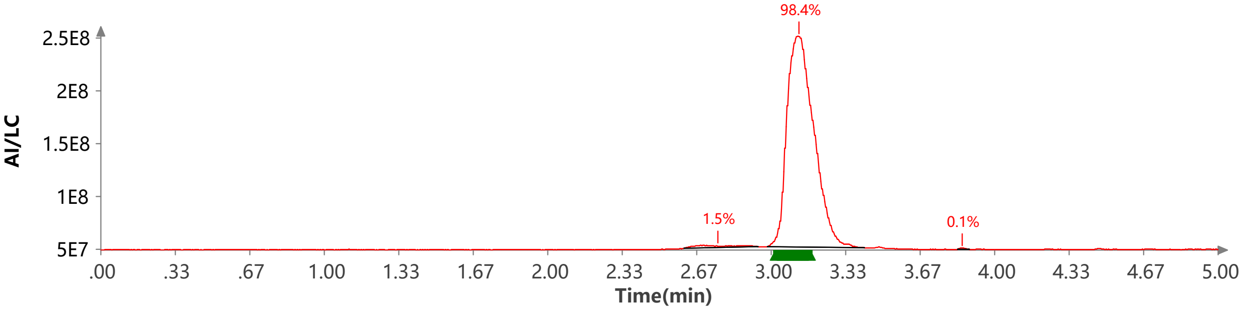

Spectrum RT 3.01 - 3.18 {49 scans}
morti01_01.datx;
ESI + Settings for tune mix using source type ESI Positive. Max: 1.5E8

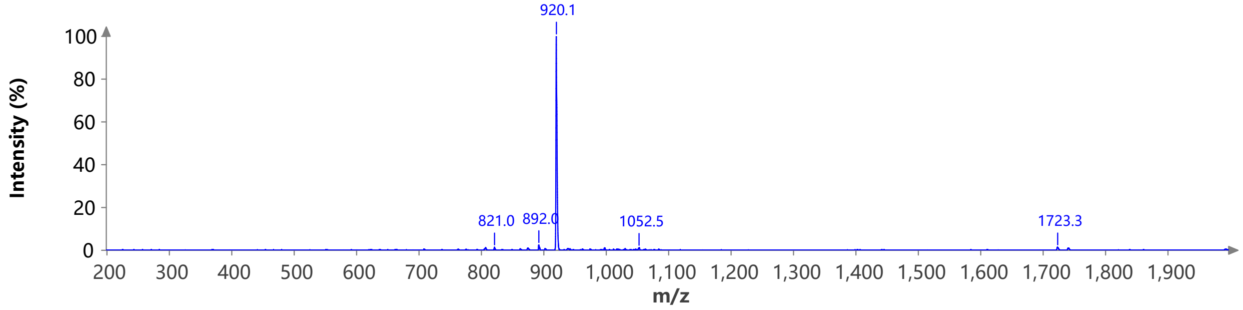

Morti02

UV 200.0 nm
JTmorti_RV2puri_OEt_01_UV.datx 2023.02.03 13:57:11;

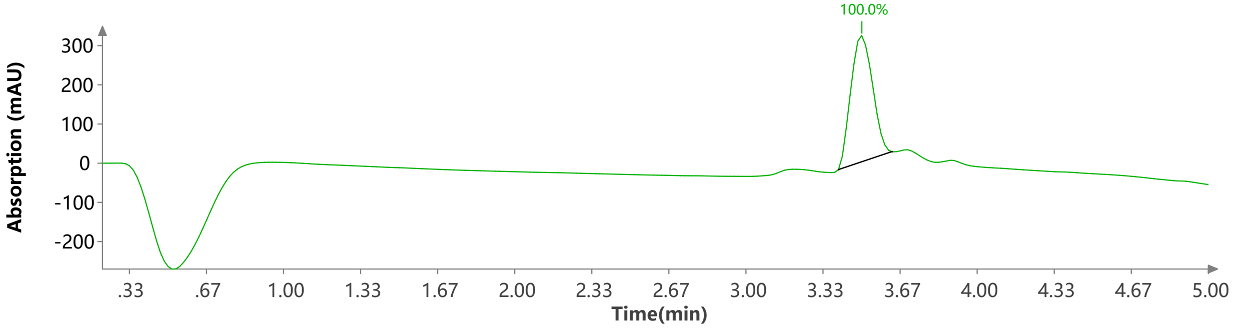

Spectrum RT 3.48 - 3.67 {54 scans}
JTmorti_RV2puri_OEt_01.datx;
ESI + Settings for tune mix using source type ESI Positive. Max: 1.4E8

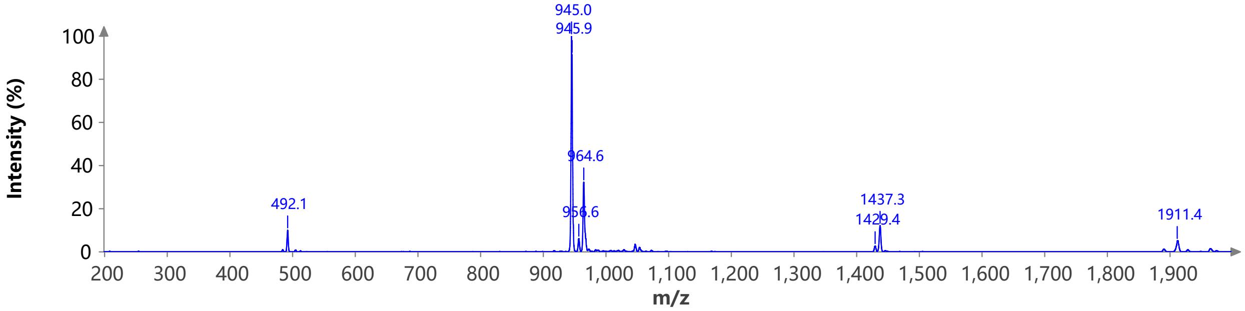

Morti03

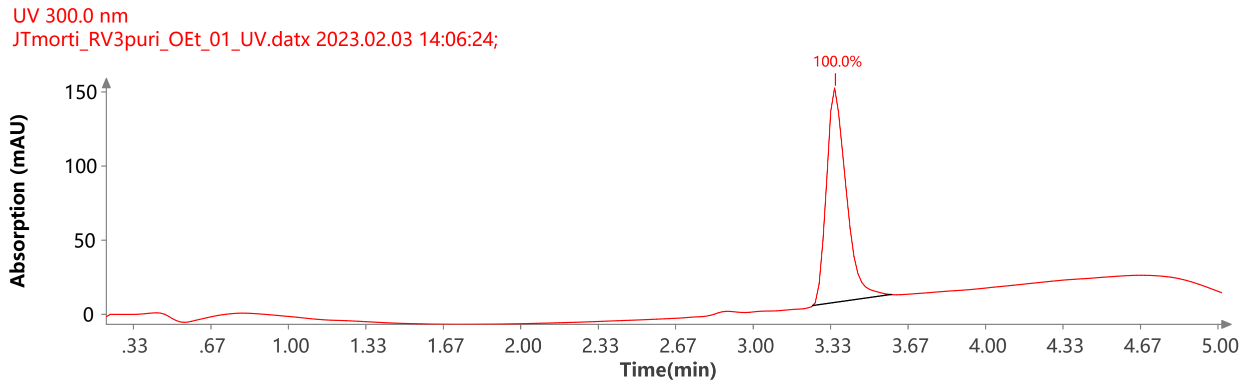

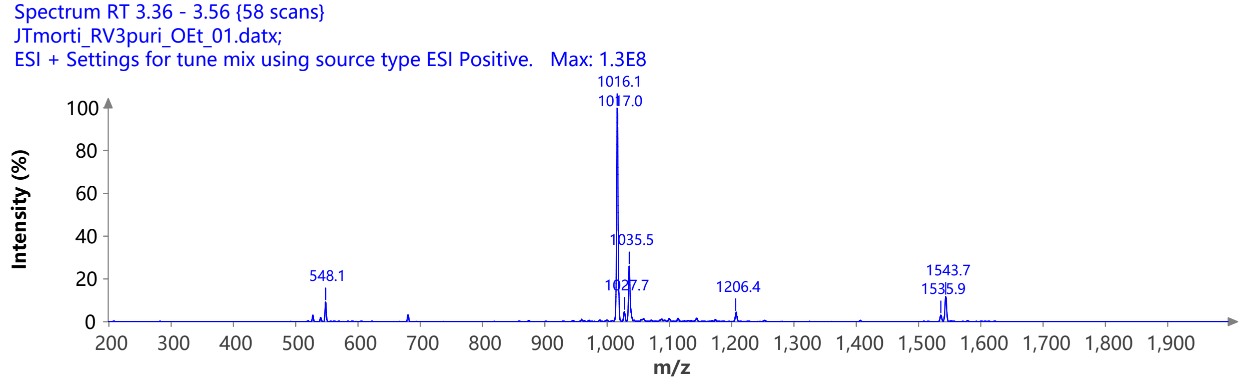

Morti04

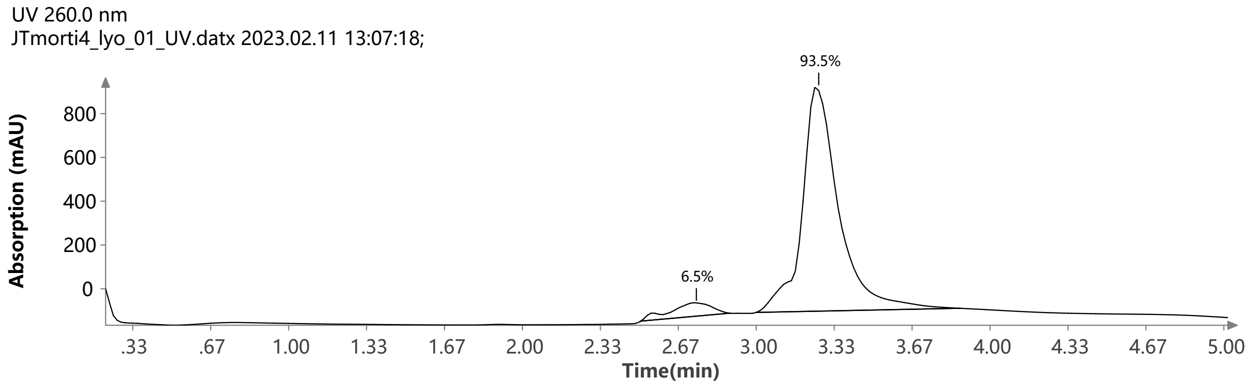

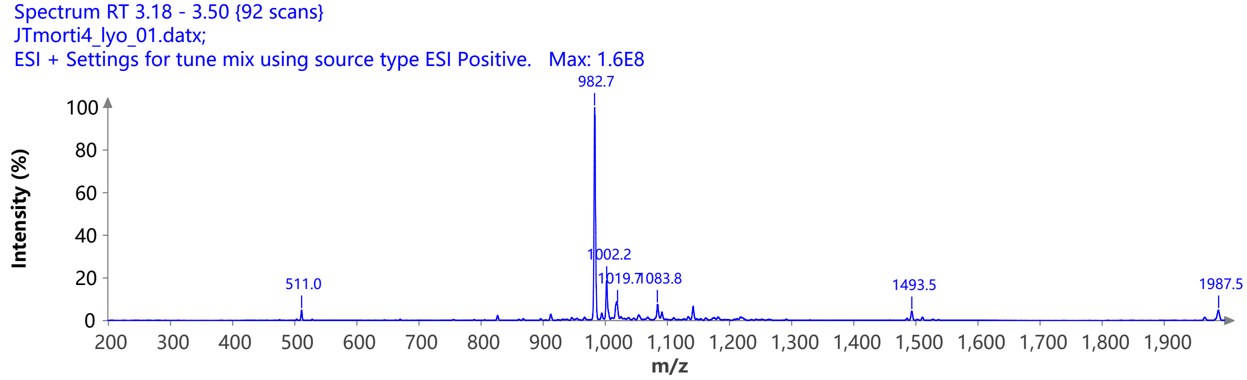

Morti05

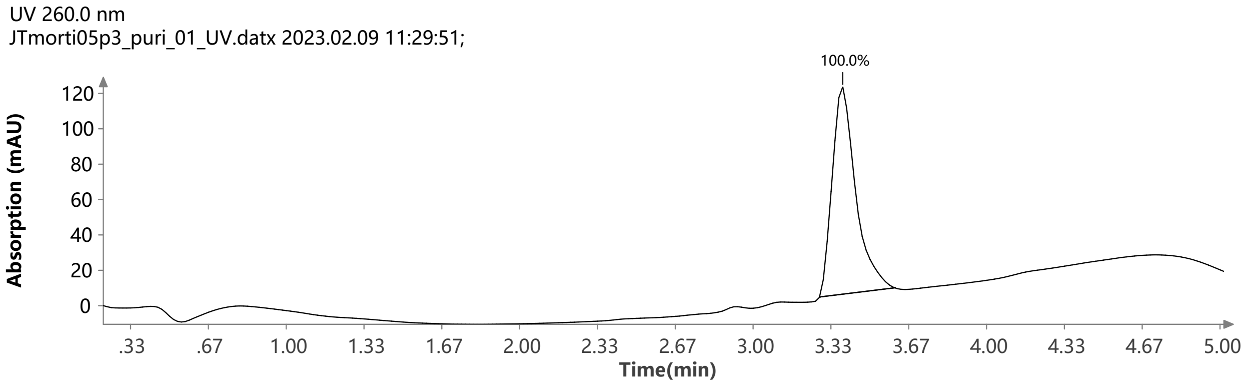

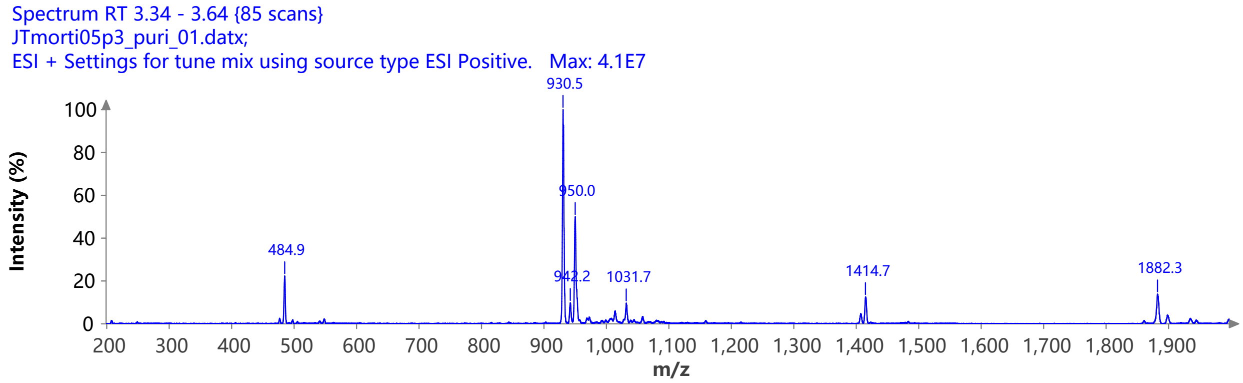

Morti06

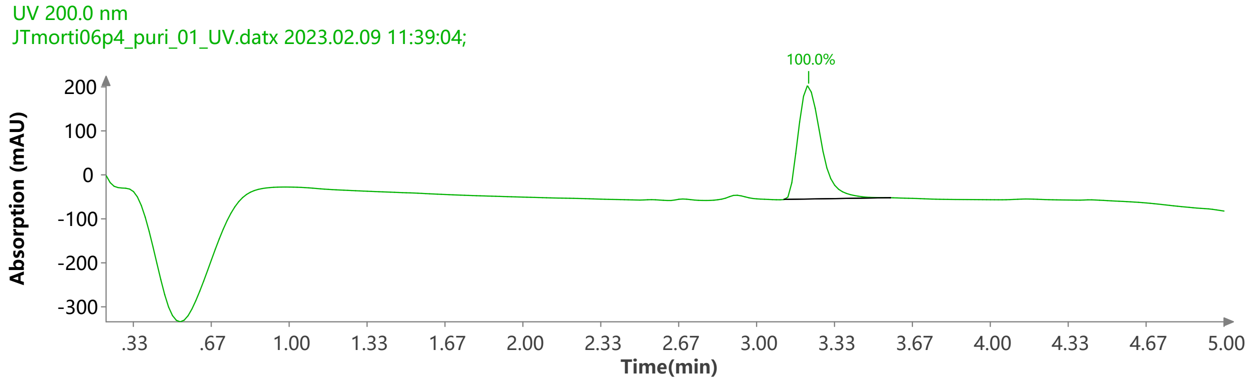

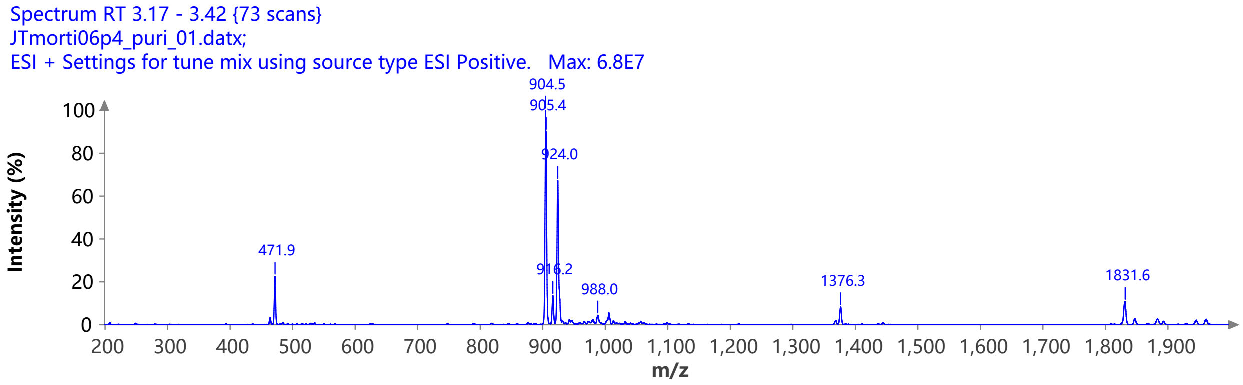

Morti07

UV 200.0 nm
JTmorti07p4_puri_01_UV.datx 2023.02.09 11:57:27;

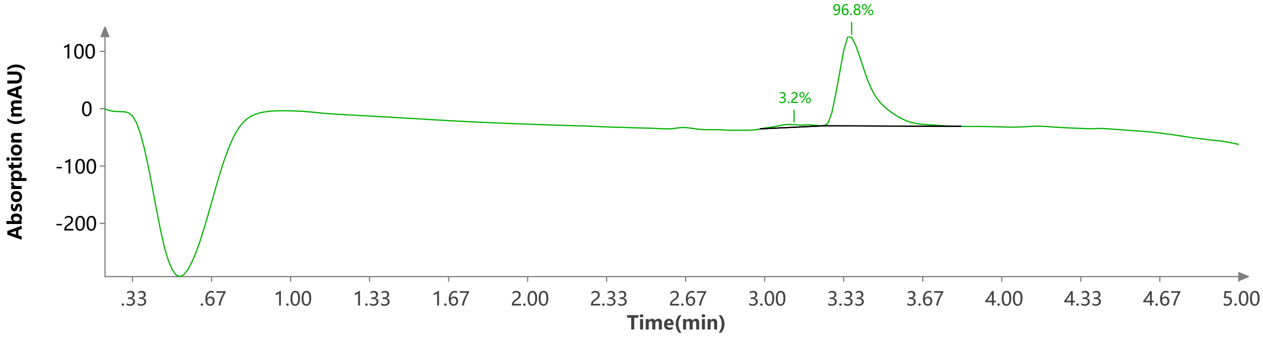

Spectrum RT 3.34 - 3.57 {66 scans}
JTmorti07p4_puri_01.datx;
ESI + Settings for tune mix using source type ESI Positive. Max: 3.1E7

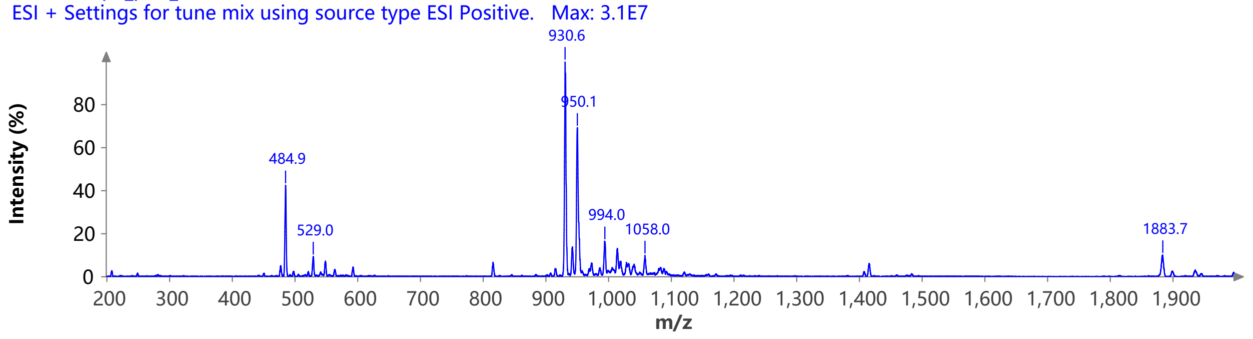

Morti08

UV 220.0 nm
morti08_re_f5_UV.datx 2023.03.09 11:42:55;

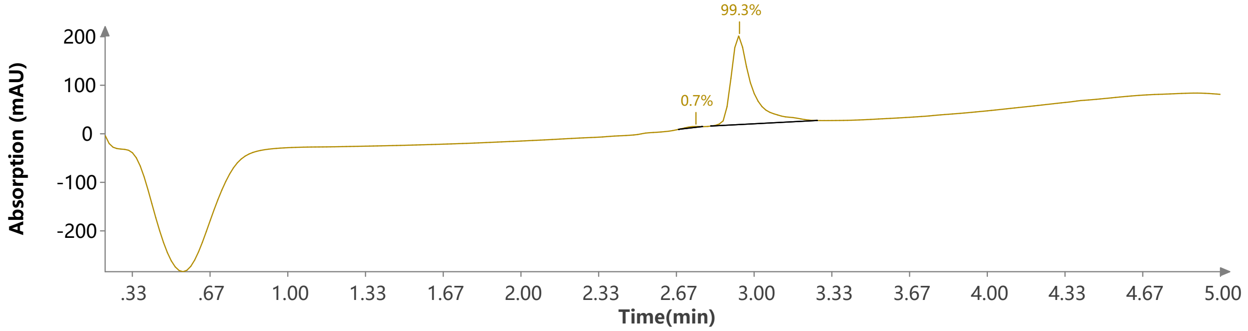

Spectrum RT 2.92 - 3.23 {87 scans}
morti08_re_f5.datx;
ESI + Settings for tune mix using source type ESI Positive. Max: 1.8E8

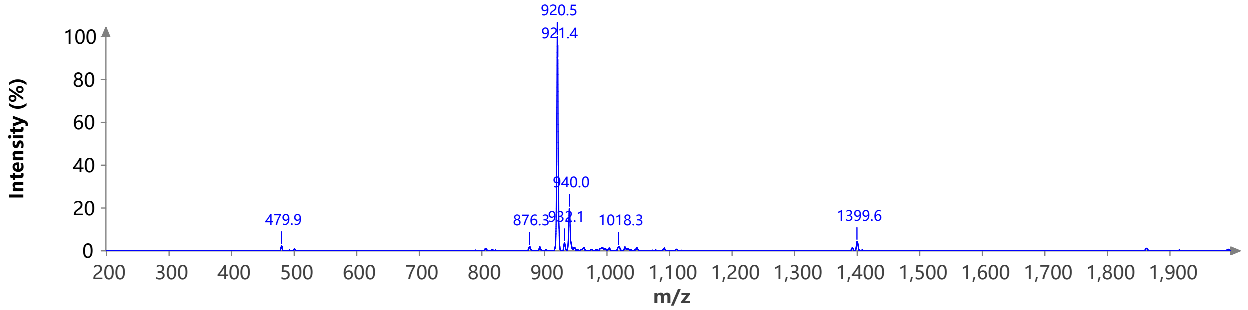

Morti09

UV 220.0 nm
morti09_re_f4_UV.datx 2023.03.09 12:10:27;

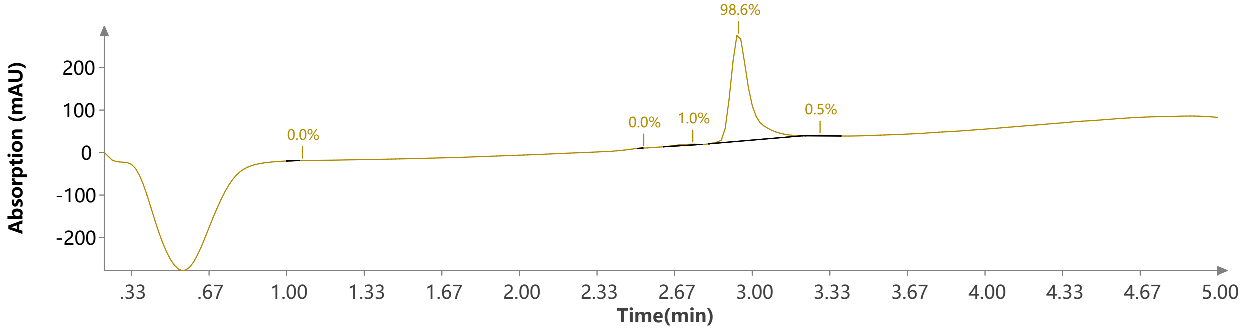

Spectrum RT 2.88 - 3.25 {107 scans}
morti09_re_f4.datx;
ESI + Settings for tune mix using source type ESI Positive. Max: 1.8E8

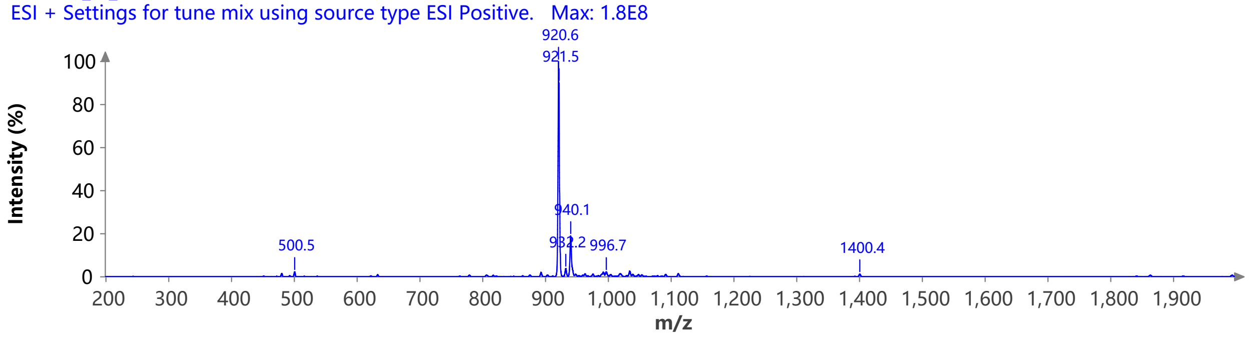

Morti10

UV 260.0 nm
morti10_OEt_f06_01_UV.datx 2023.03.08 10:00:46;

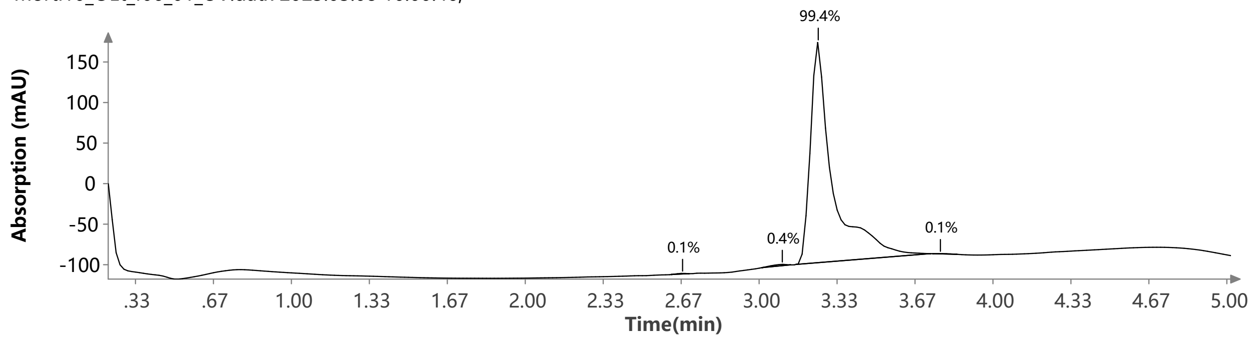

Spectrum RT 3.26 - 3.56 {85 scans}
morti10_OEt_f06_01.datx;
ESI + Settings for tune mix using source type ESI Positive. Max: 8.5E7

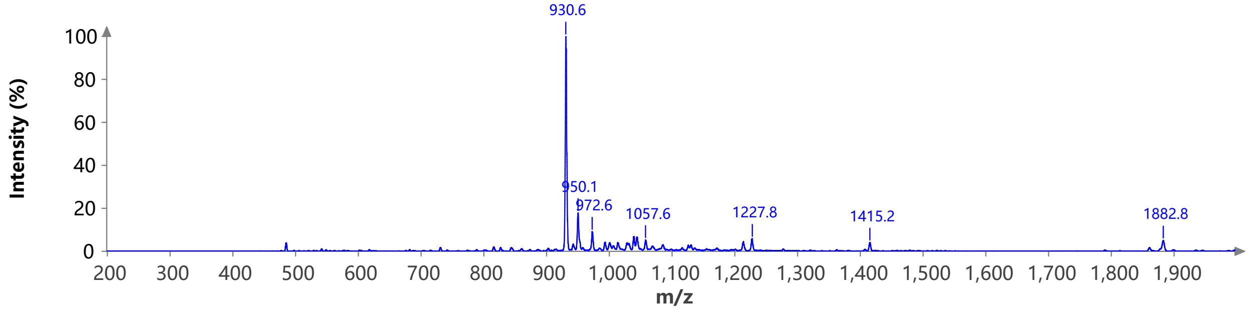

Morti11

UV 260.0 nm
morti11_re_f3_UV.datx 2023.03.09 12:38:00;

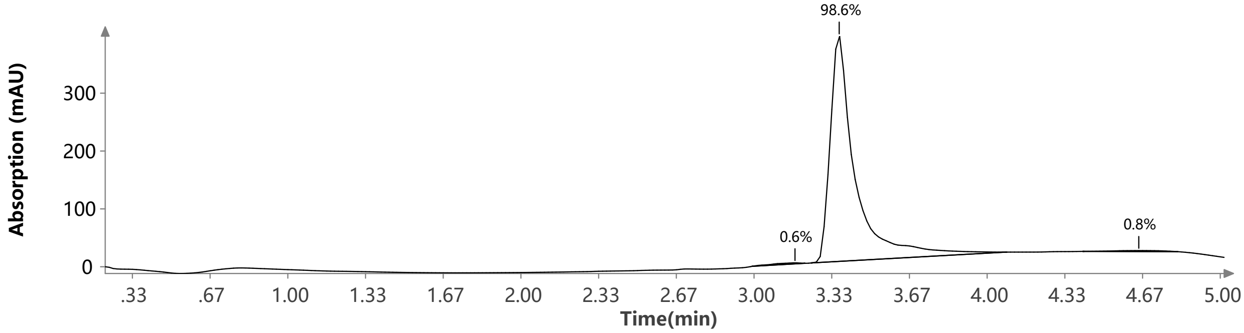

Spectrum RT 3.37 - 3.60 {66 scans}
morti11_re_f3.datx;
ESI + Settings for tune mix using source type ESI Positive. Max: 1.7E8

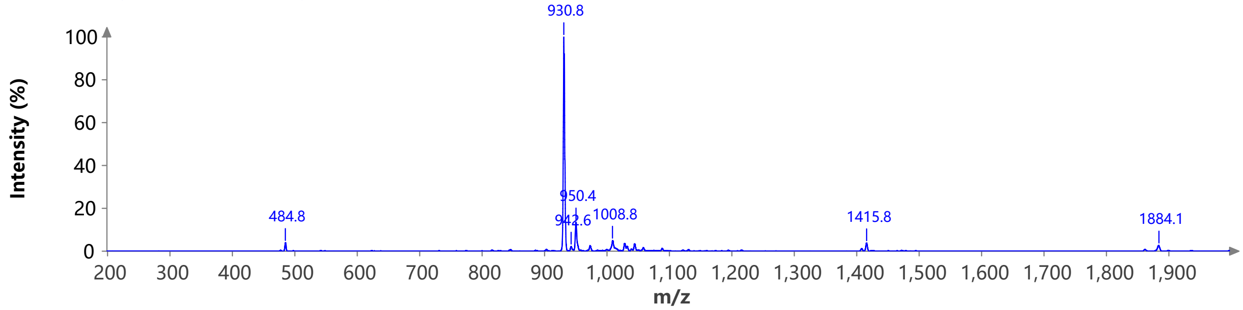

Morti12

UV 260.0 nm
morti12_f9_lyo_01_UV.datx 2023.03.10 11:43:33;

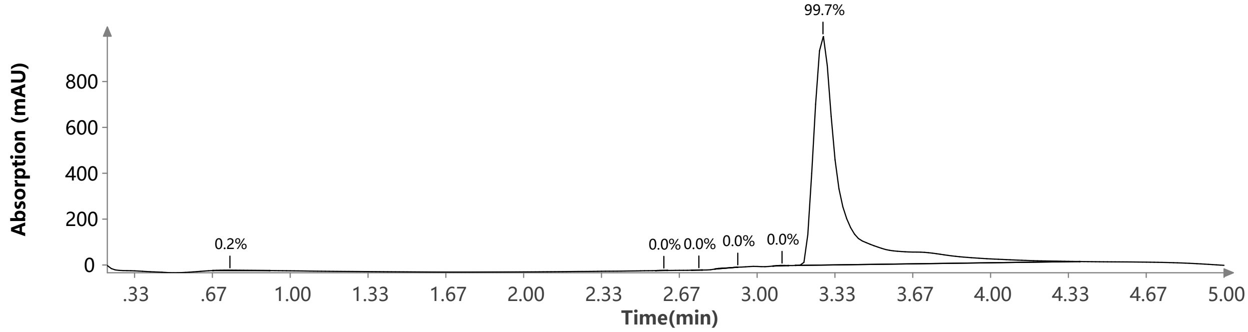

Spectrum RT 3.30 - 3.50 {58 scans}
morti12_f9_lyo_01.datx;
ESI + Settings for tune mix using source type ESI Positive. Max: 2.1E8

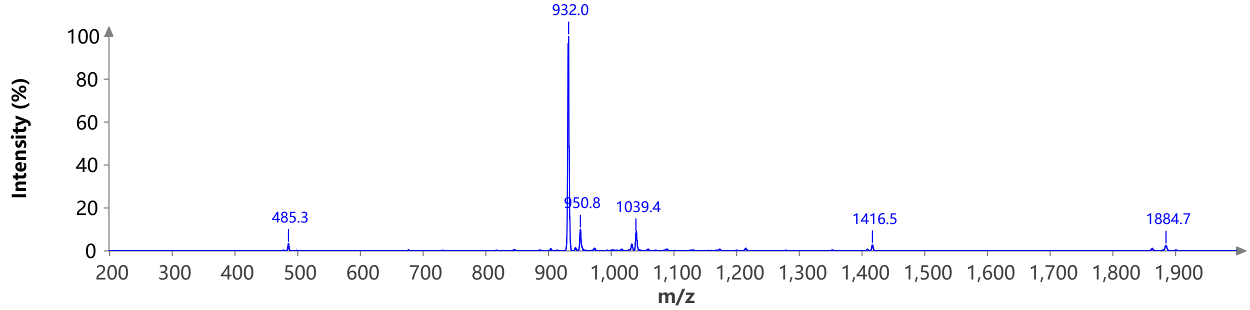

Morti13

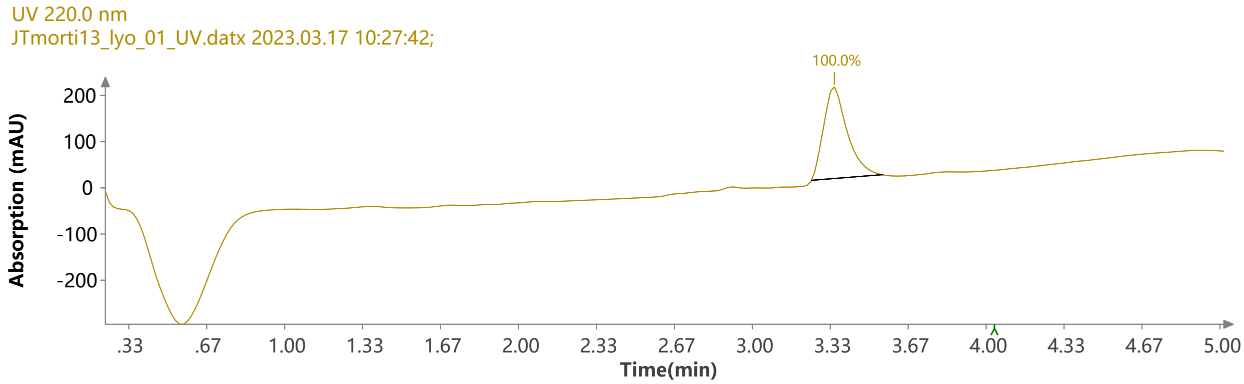

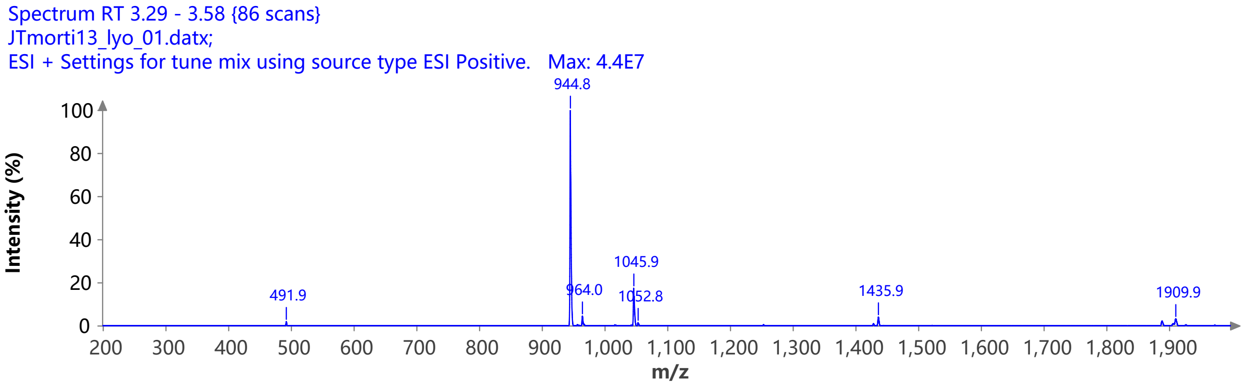

Morti14

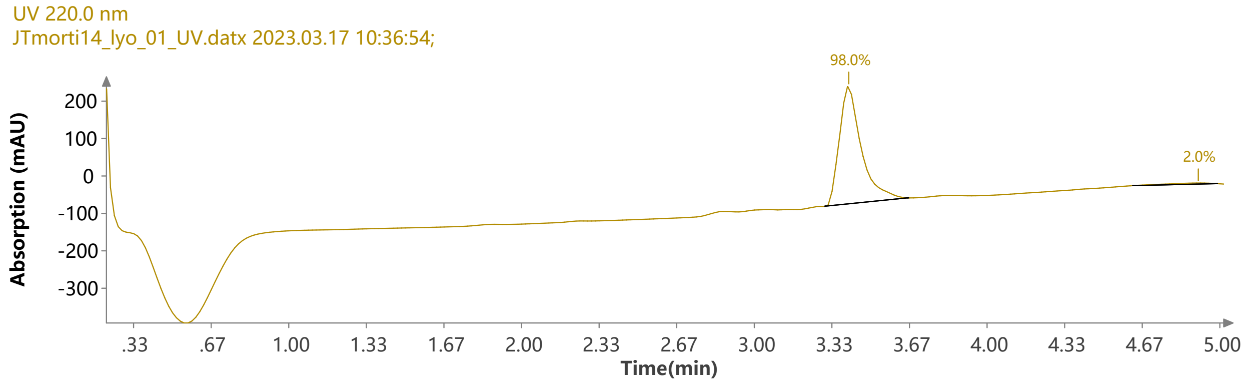

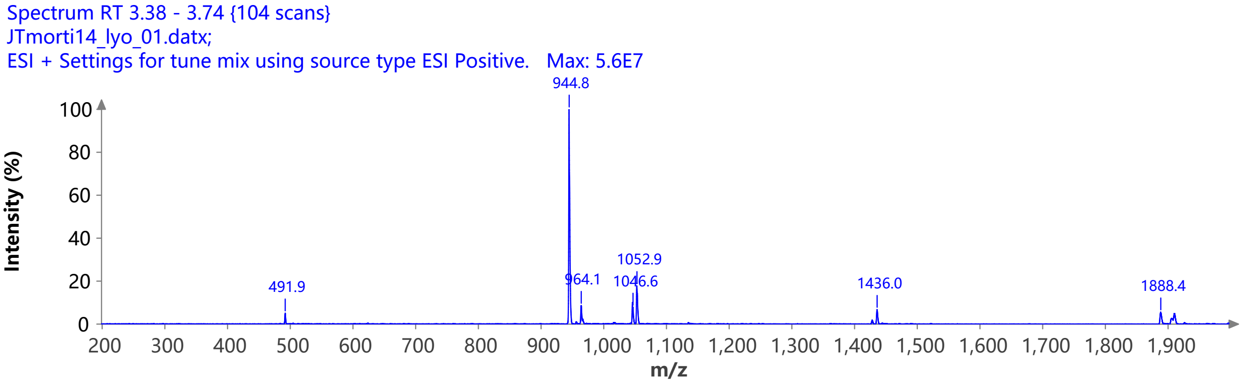

1A3

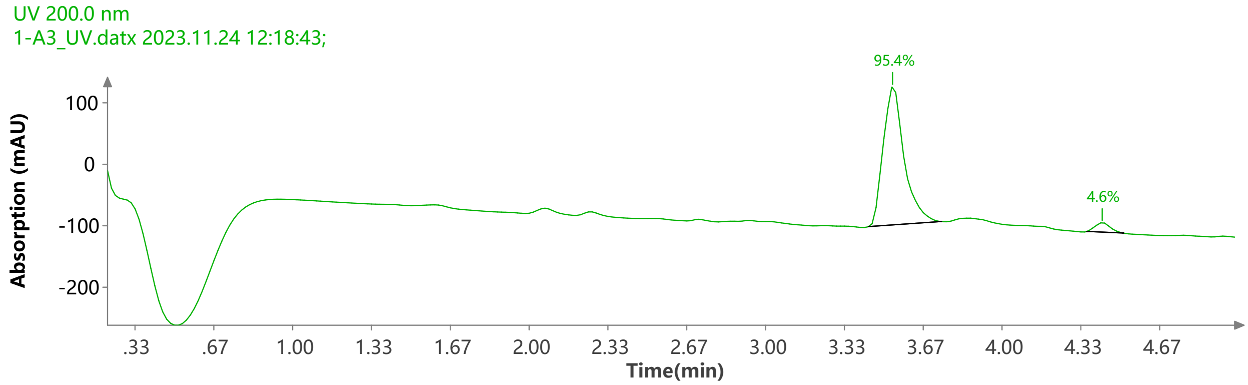
UV 200.0 nm
1-A3_UV.datx 2023.11.24 12:18:43;
95.4%
4.6%
Absorption (mAU)
Time(min)

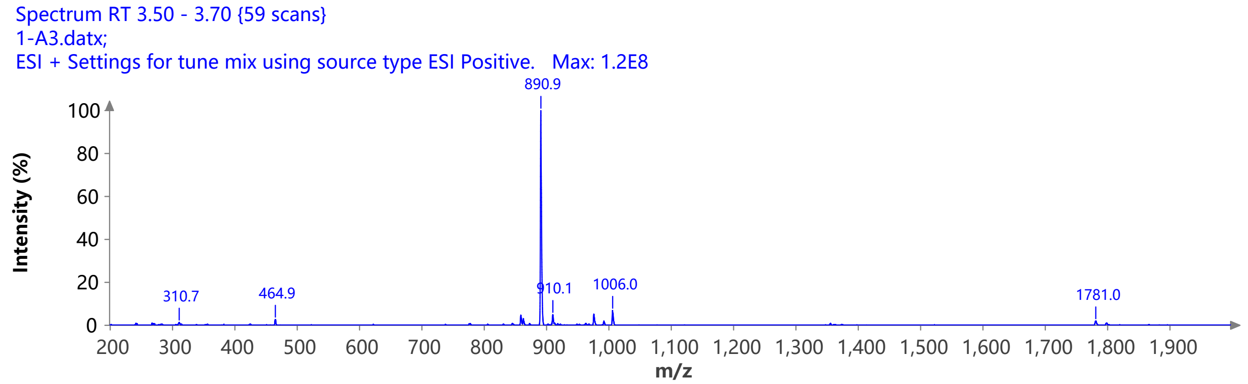
Spectrum RT 3.50 - 3.70 {59 scans}
1-A3.datx;
ESI + Settings for tune mix using source type ESI Positive. Max: 1.2E8
890.9
310.7
464.9
910.1
1006.0
1781.0
Intensity (%)
m/z

1A10

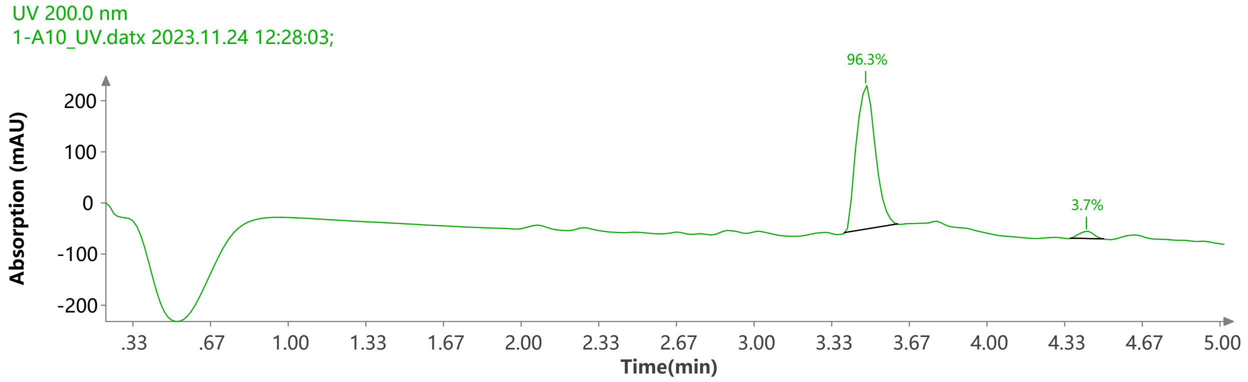
UV 200.0 nm
1-A10_UV.datx 2023.11.24 12:28:03;
96.3%
3.7%
Absorption (mAU)
Time(min)

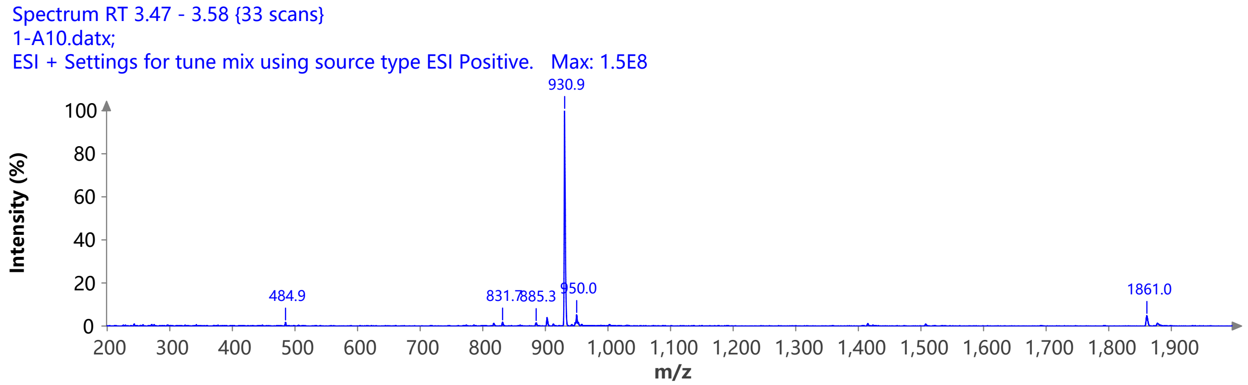
Spectrum RT 3.47 - 3.58 {33 scans}
1-A10.datx;
ESI + Settings for tune mix using source type ESI Positive. Max: 1.5E8
930.9
484.9
831.7
885.3
950.0
1861.0
Intensity (%)
m/z

1B7

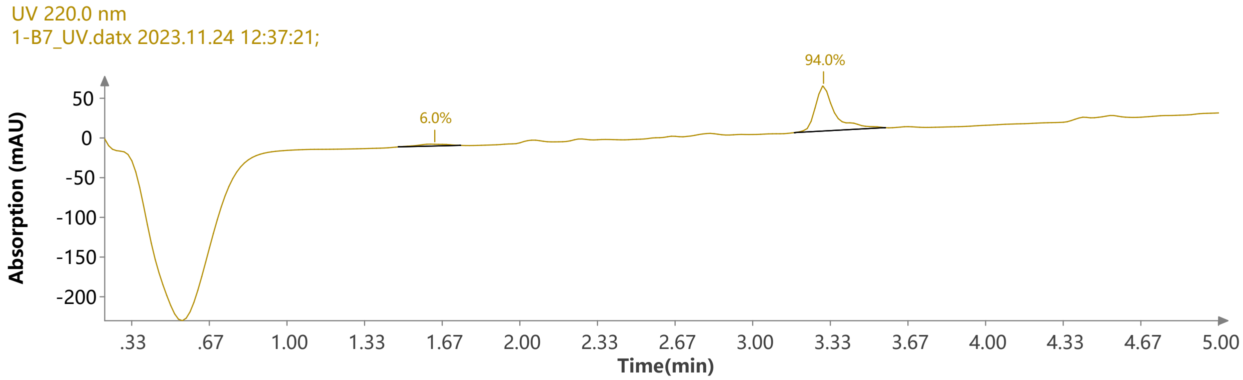
UV 220.0 nm
1-B7_UV.datx 2023.11.24 12:37:21;
6.0%
94.0%
Absorption (mAU)
50
0
-50
-100
-150
-200
.33
.67
1.00
1.33
1.67
2.00
2.33
2.67
3.00
3.33
3.67
4.00
4.33
4.67
5.00
Time(min)

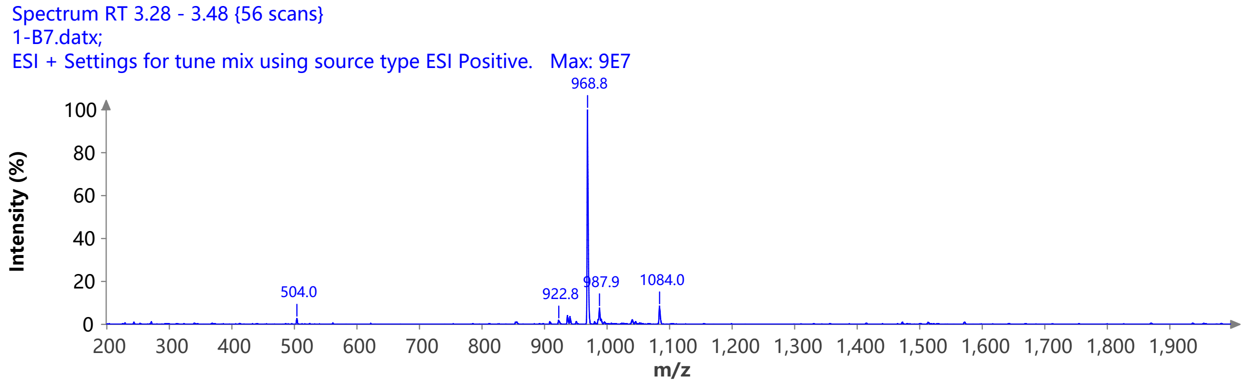
Spectrum RT 3.28 - 3.48 {56 scans}
1-B7.datx;
ESI + Settings for tune mix using source type ESI Positive.   Max: 9E7
968.8
504.0
922.8
987.9
1084.0
Intensity (%)
100
80
60
40
20
0
200
300
400
500
600
700
800
900
1,000
1,100
1,200
1,300
1,400
1,500
1,600
1,700
1,800
1,900
m/z

1C5

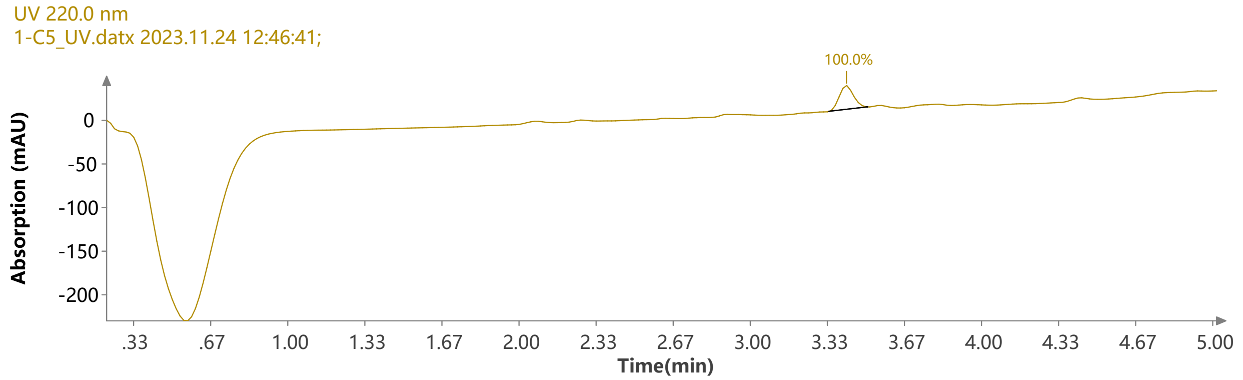
UV 220.0 nm
1-C5_UV.datx 2023.11.24 12:46:41;
100.0%
Absorption (mAU)
0
-50
-100
-150
-200
.33
.67
1.00
1.33
1.67
2.00
2.33
2.67
3.00
3.33
3.67
4.00
4.33
4.67
5.00
Time(min)

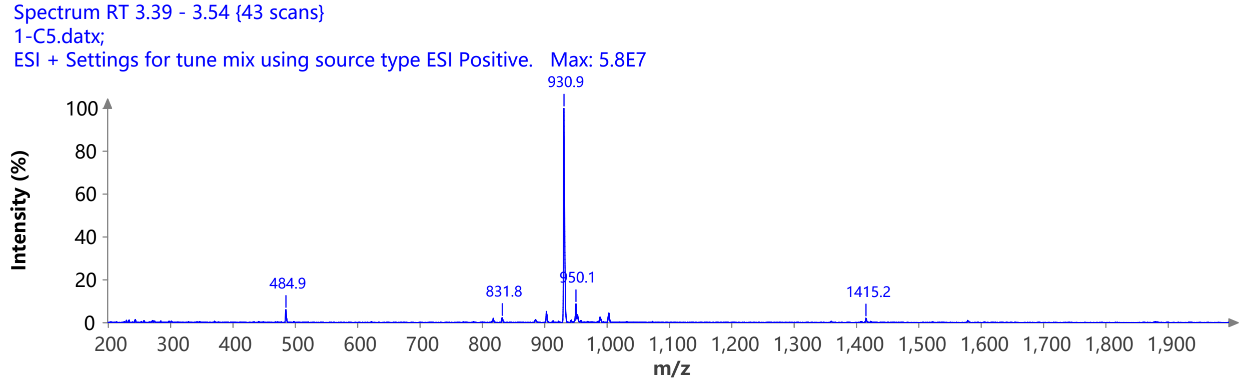
Spectrum RT 3.39 - 3.54 {43 scans}
1-C5.datx;
ESI + Settings for tune mix using source type ESI Positive.   Max: 5.8E7
930.9
484.9
831.8
950.1
1415.2
Intensity (%)
100
80
60
40
20
0
200
300
400
500
600
700
800
900
1,000
1,100
1,200
1,300
1,400
1,500
1,600
1,700
1,800
1,900
m/z

1C10

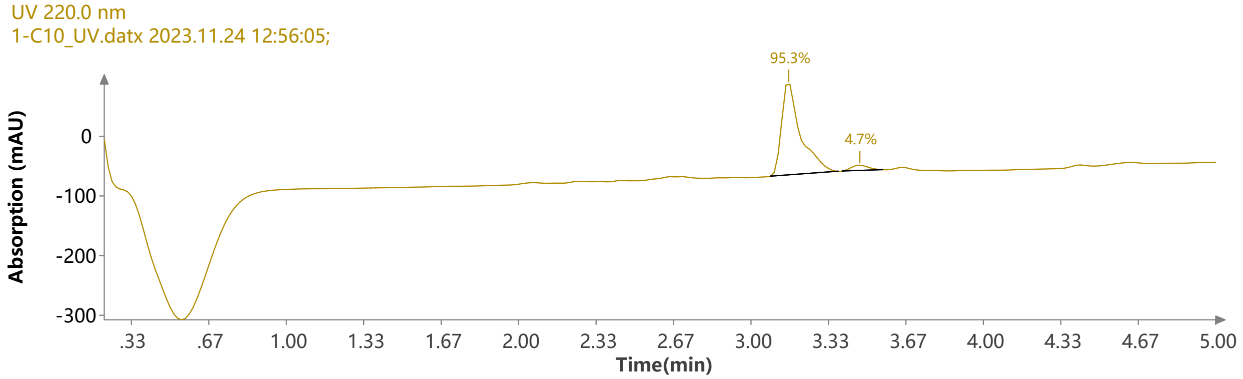

UV 220.0 nm
1-C10_UV.datx 2023.11.24 12:56:05;
95.3%
4.7%
Absorption (mAU)
Time(min)

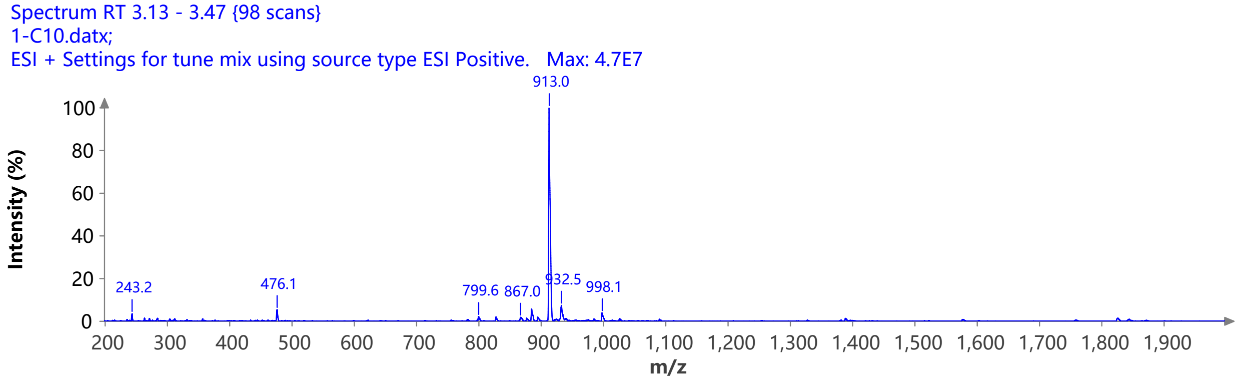

Spectrum RT 3.13 - 3.47 {98 scans}
1-C10.datx;
ESI + Settings for tune mix using source type ESI Positive. Max: 4.7E7
913.0
243.2
476.1
799.6
867.0
932.5
998.1
Intensity (%)
m/z

1D3

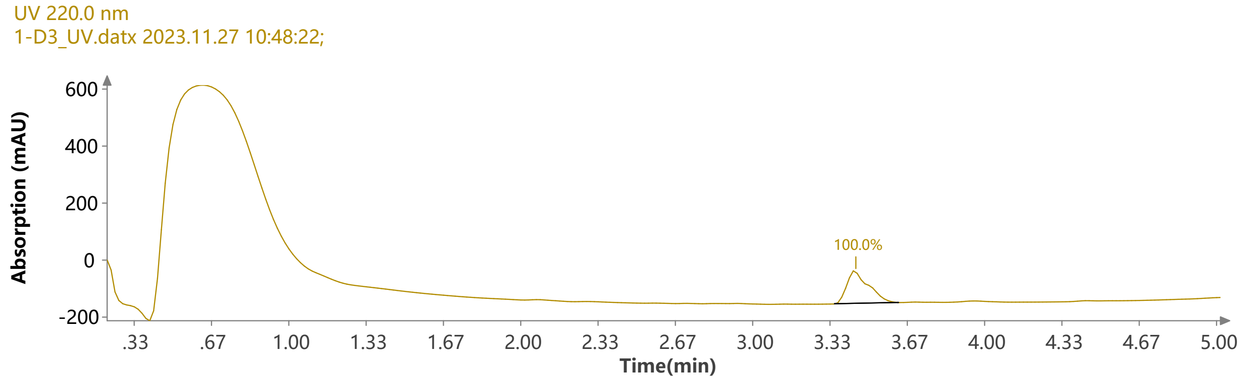

UV 220.0 nm
1-D3_UV.datx 2023.11.27 10:48:22;
100.0%
Absorption (mAU)
Time(min)

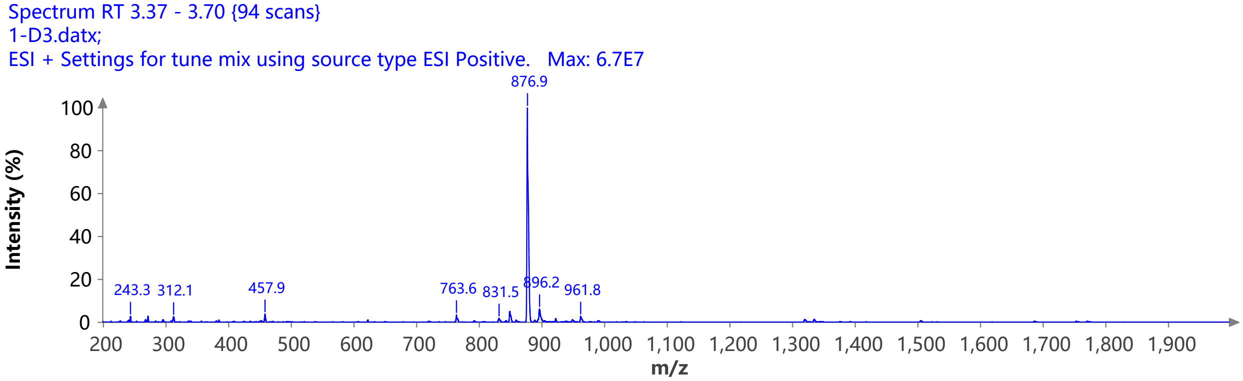

Spectrum RT 3.37 - 3.70 {94 scans}
1-D3.datx;
ESI + Settings for tune mix using source type ESI Positive. Max: 6.7E7
876.9
243.3
312.1
457.9
763.6
831.5
896.2
961.8
Intensity (%)
m/z

1D6

UV 260.0 nm
1-D6_UV.datx 2023.11.24 13:14:45;

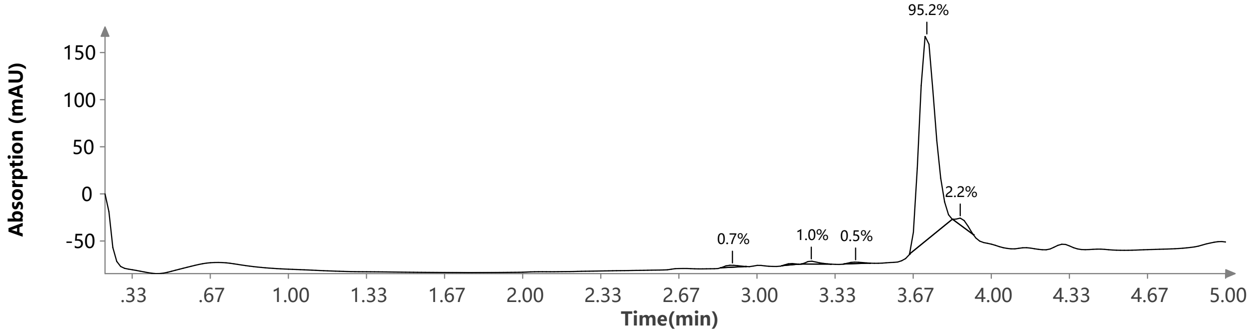

Spectrum RT 3.64 - 3.81 {47 scans}
1-D6.datx;
ESI + Settings for tune mix using source type ESI Positive. Max: 1.2E8

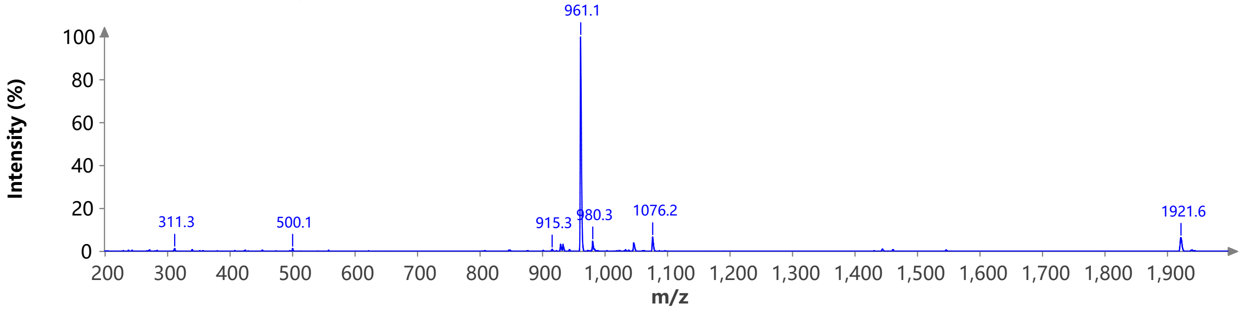

1D9

UV 220.0 nm
1-D9_UV.datx 2023.11.24 13:24:07;

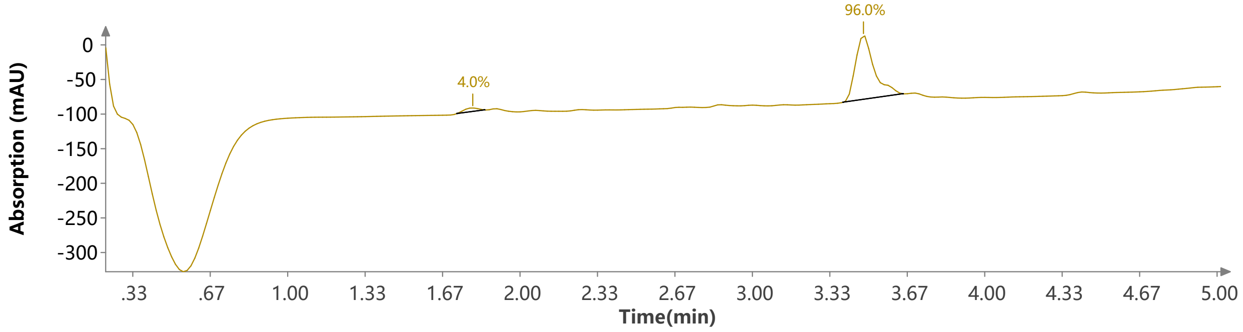

Spectrum RT 3.43 - 3.72 {82 scans}
1-D9.datx;
ESI + Settings for tune mix using source type ESI Positive. Max: 8.7E7

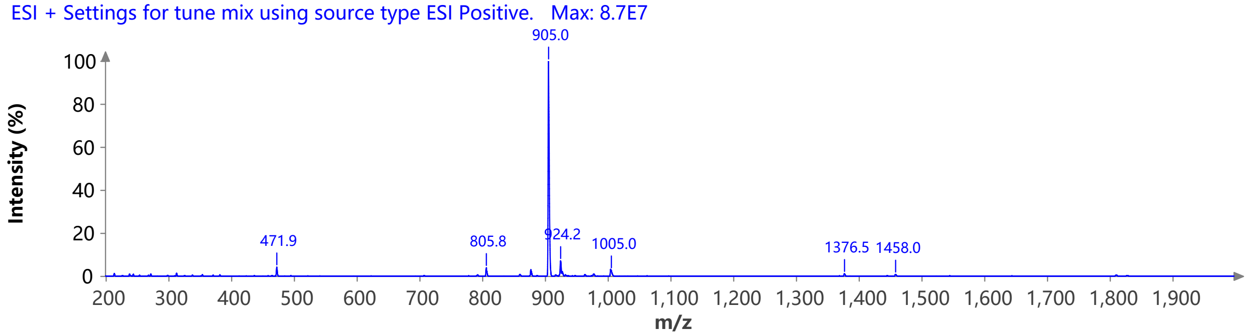

1E6

UV 220.0 nm
1-E6_UV.datx 2023.11.24 13:33:26;

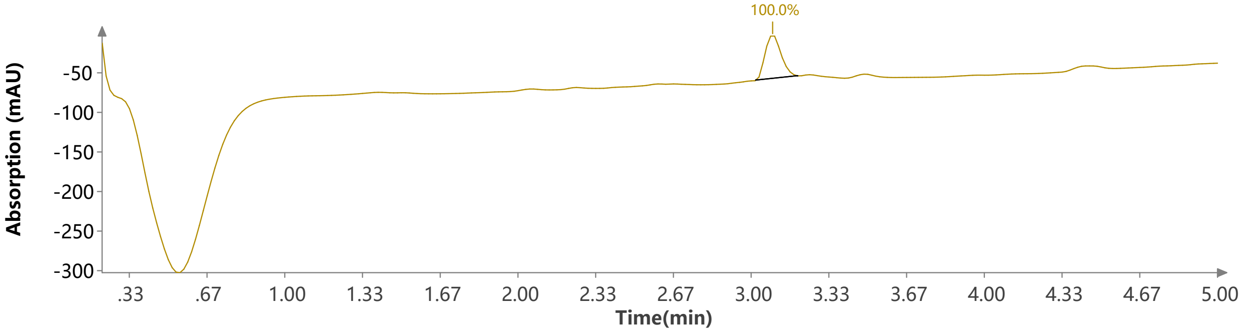

Spectrum RT 3.07 - 3.23 {47 scans}
1-E6.datx;
ESI + Settings for tune mix using source type ESI Positive.   Max: 4.8E7

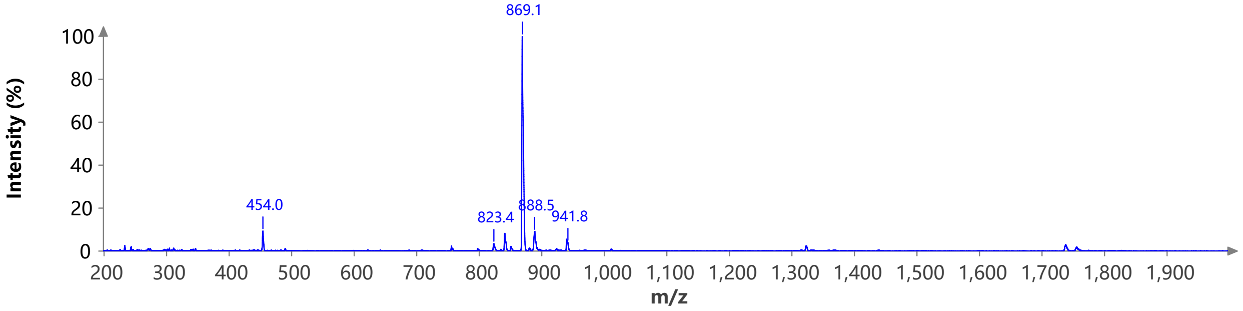

1E7

UV 200.0 nm
1E7OEt_01_UV.datx 2023.12.18 08:16:22;

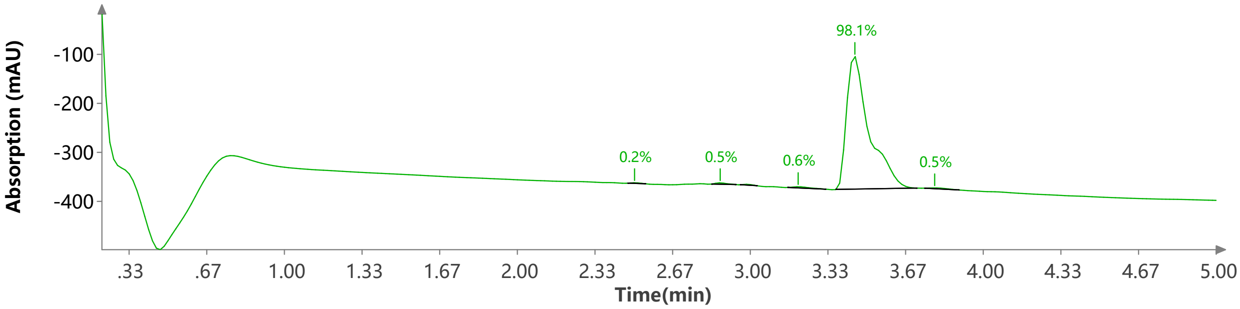

Spectrum RT 3.42 - 3.62 {57 scans}
1E7OEt_01.datx;
ESI + Settings for tune mix using source type ESI Positive.   Max: 7.4E6

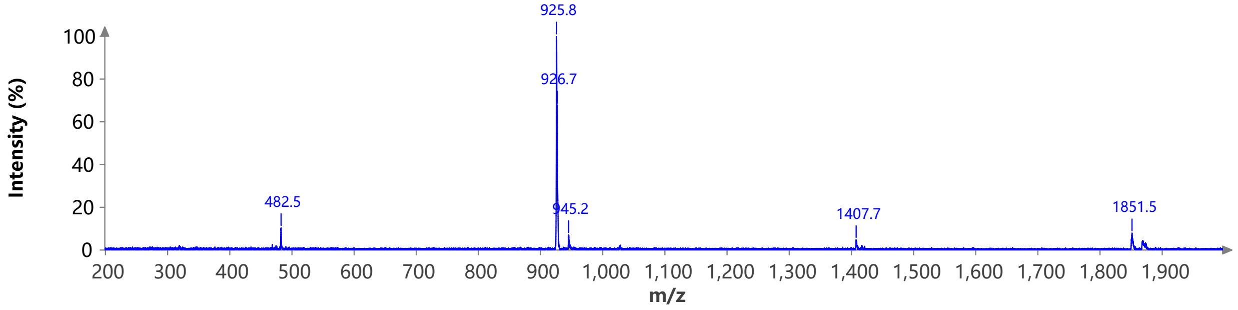

1E10

UV 260.0 nm
1-E10_UV.datx 2023.11.24 13:52:05;

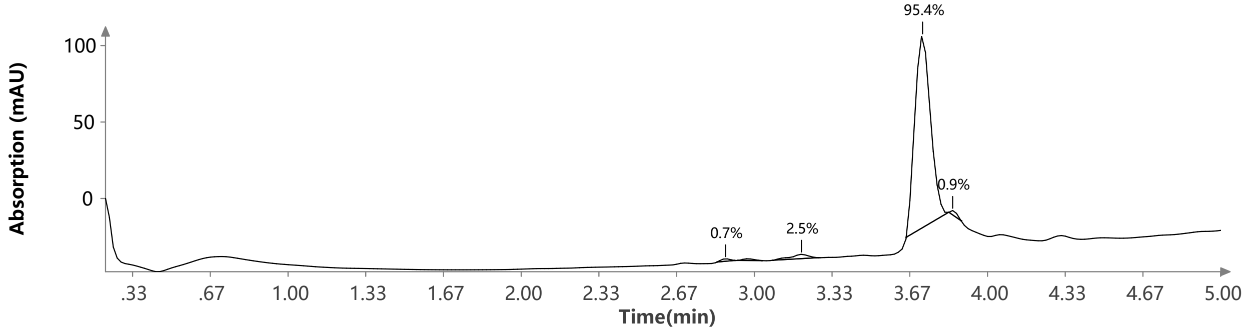

Spectrum RT 3.67 - 3.79 {33 scans}
1-E10.datx;
ESI + Settings for tune mix using source type ESI Positive.   Max: 1.2E8

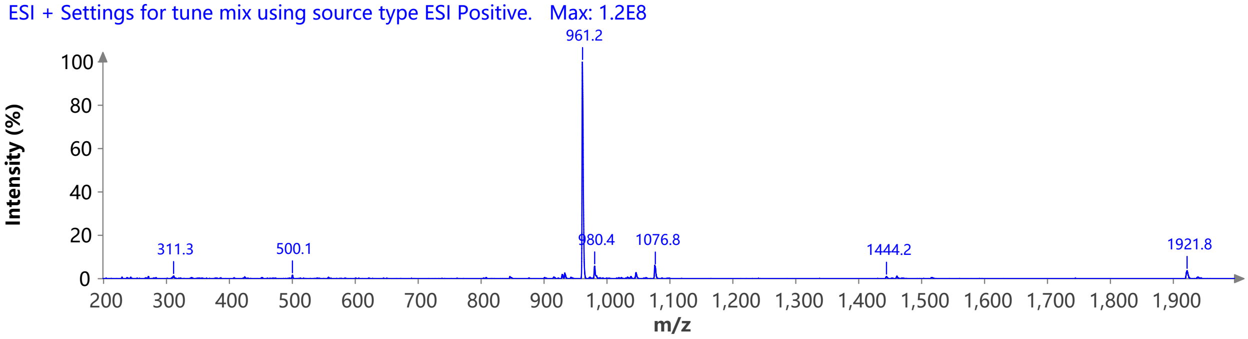

1F3

UV 220.0 nm
1-F3_UV.datx 2023.11.24 14:01:24;

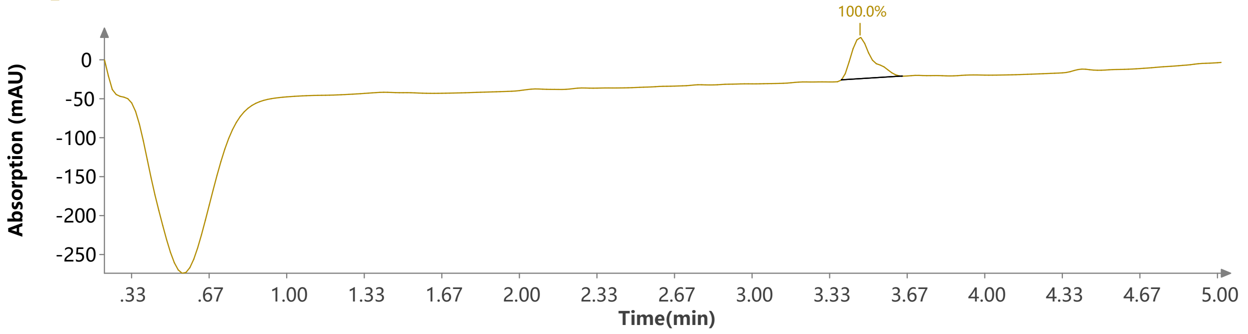

Spectrum RT 3.44 - 3.54 {29 scans}
1-F3.datx;
ESI + Settings for tune mix using source type ESI Positive.   Max: 9.8E7

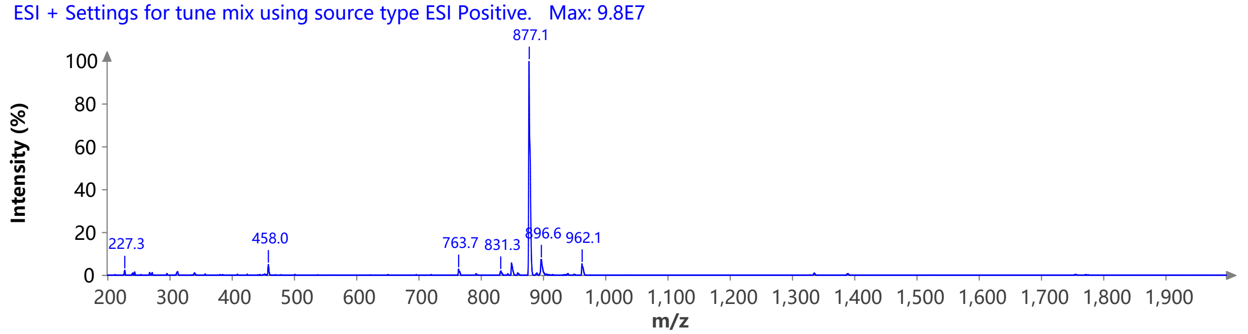

2A8

UV 220.0 nm
2-A8_UV.datx 2023.11.24 14:10:43;

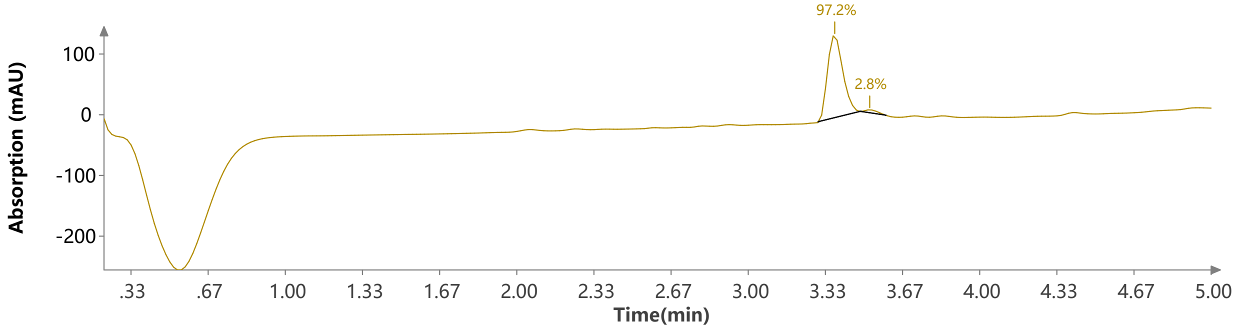

Spectrum RT 3.36 - 3.60 {67 scans}
2-A8.datx;
ESI + Settings for tune mix using source type ESI Positive. Max: 7.3E7

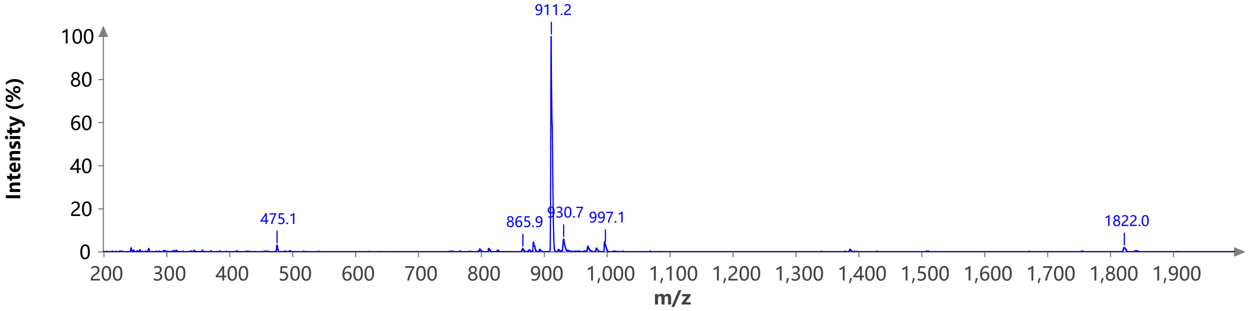

2A9

UV 220.0 nm
2-A9_UV.datx 2023.11.24 14:20:04;

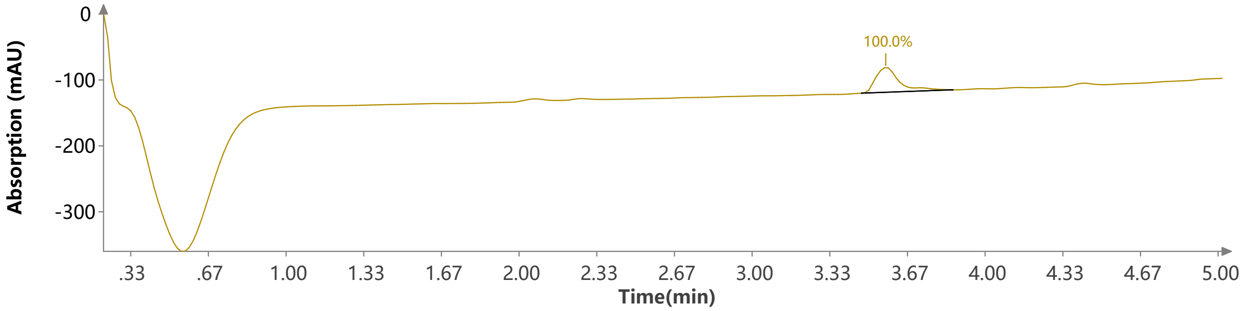

Spectrum RT 3.48 - 3.66 {52 scans}
2-A9.datx;
ESI + Settings for tune mix using source type ESI Positive. Max: 1.6E8

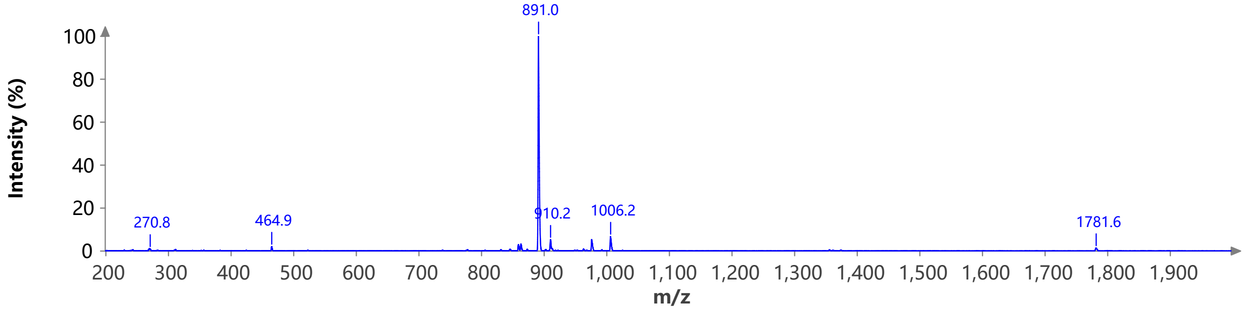

2A11

UV 220.0 nm
2-A11_UV.datx 2023.11.24 14:29:24;

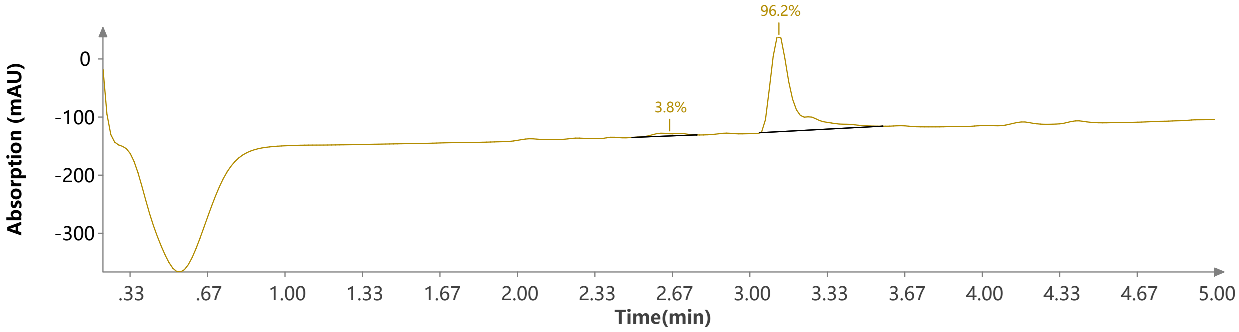

Spectrum RT 3.10 - 3.31 {61 scans}
2-A11.datx;
ESI + Settings for tune mix using source type ESI Positive. Max: 6.2E7

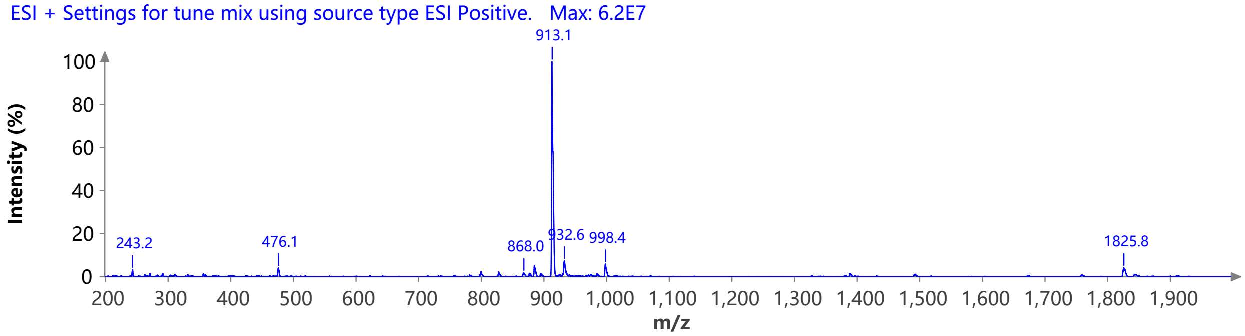

2B3

UV 200.0 nm
2B3OEt_01_UV.datx 2023.12.18 08:25:35;

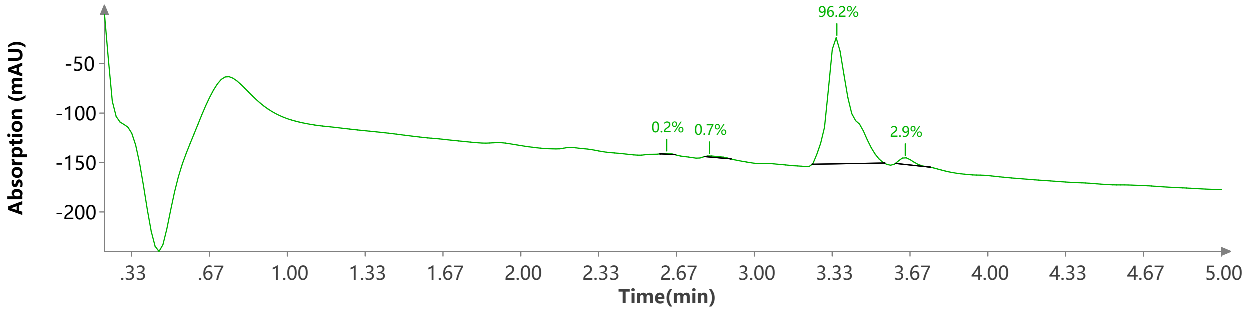

Spectrum RT 3.33 - 3.63 {87 scans}
2B3OEt_01.datx;
ESI + Settings for tune mix using source type ESI Positive. Max: 3.8E6

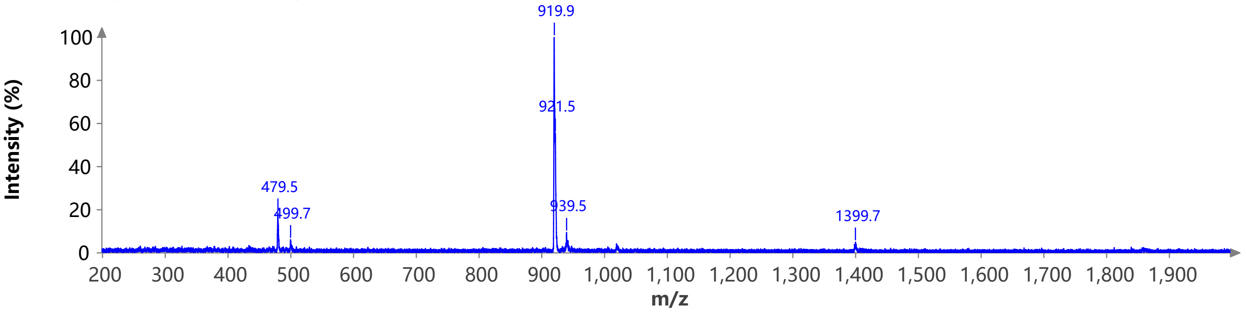

2B5

UV 220.0 nm
2B5OEt_01_UV.datx 2023.12.18 08:34:53;

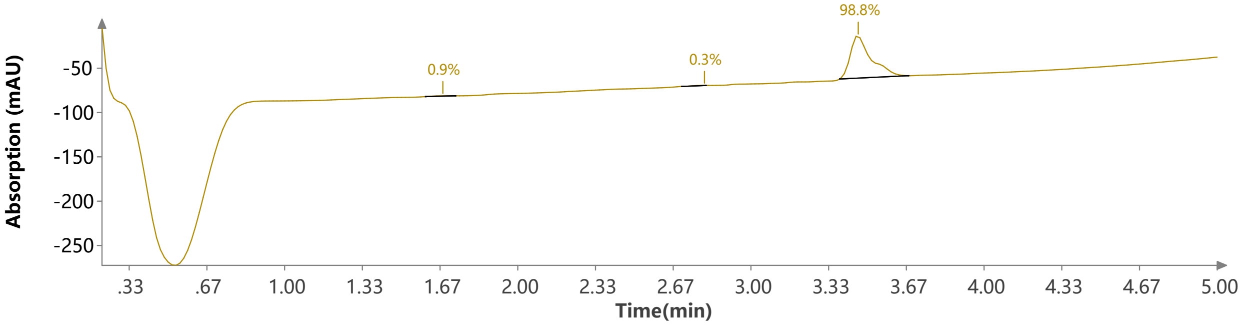

Spectrum RT 3.43 - 3.73 {87 scans}
2B5OEt_01.datx;
ESI + Settings for tune mix using source type ESI Positive. Max: 7.3E6

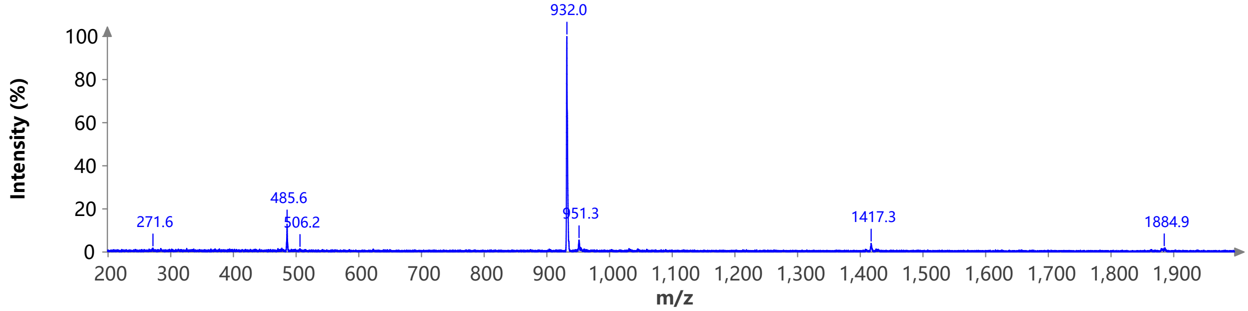

2B8

UV 220.0 nm
2-B8_UV.datx 2023.11.24 14:57:21;

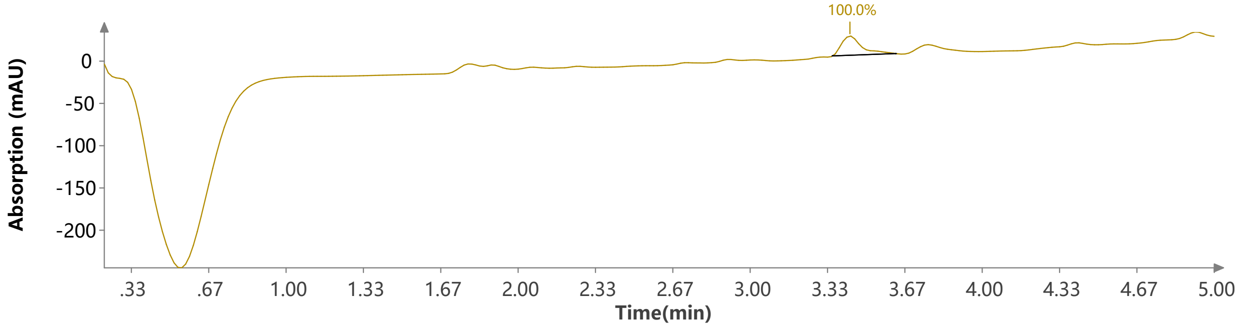

Spectrum RT 3.43 - 3.58 {45 scans}
2-B8.datx;
ESI + Settings for tune mix using source type ESI Positive. Max: 3.6E7

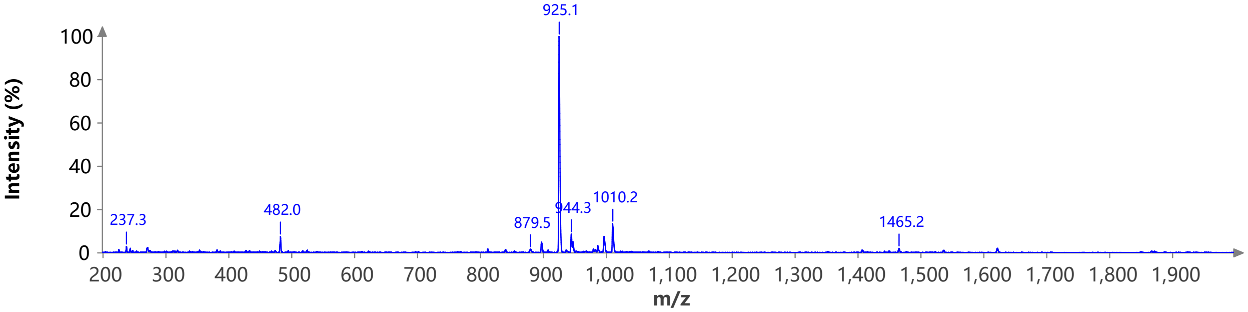

2C3

UV 220.0 nm
2-C3_UV.datx 2023.11.24 15:06:41;

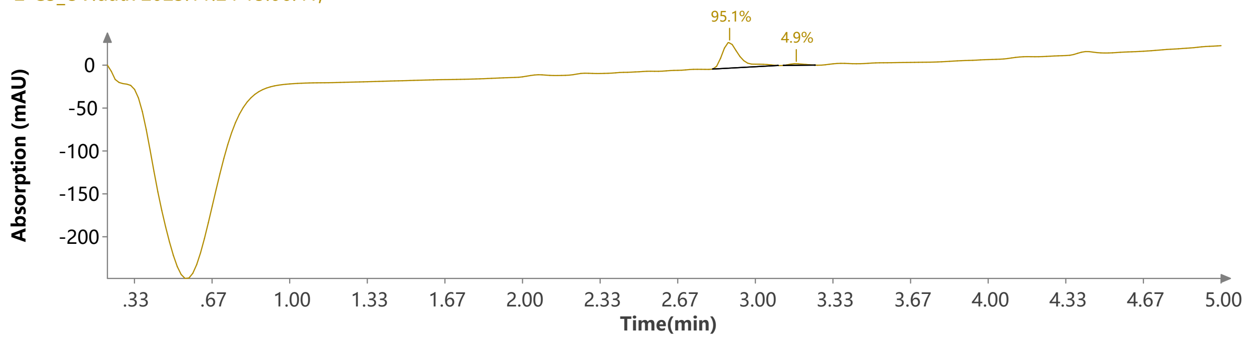

Spectrum RT 2.86 - 3.12 {75 scans}
2-C3.datx;
ESI + Settings for tune mix using source type ESI Positive. Max: 1.3E8

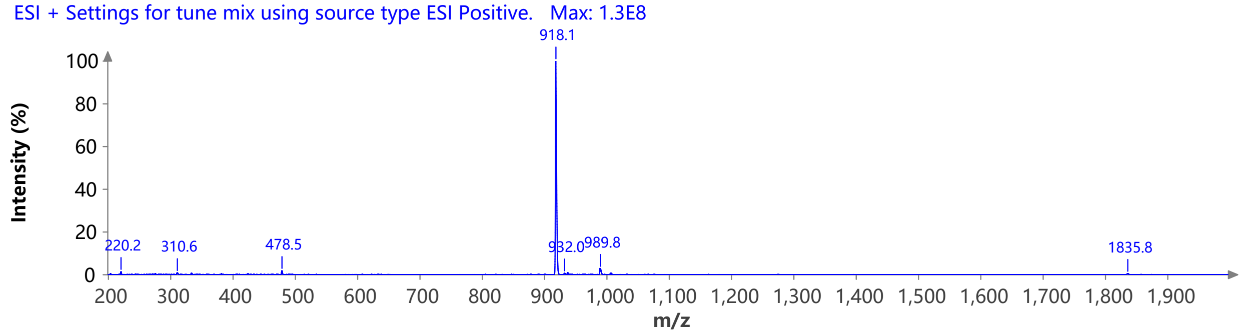

2C6

UV 220.0 nm
2-C6_UV.datx 2023.11.24 15:16:02;

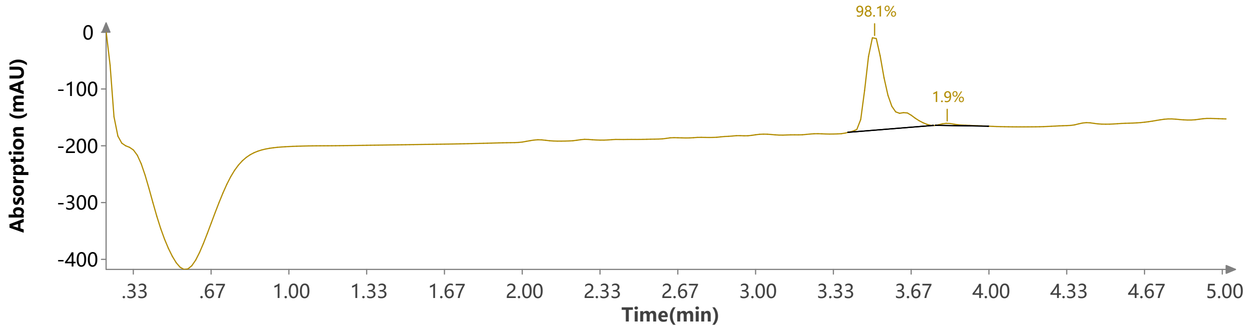

Spectrum RT 3.44 - 3.60 {47 scans}
2-C6.datx;
ESI + Settings for tune mix using source type ESI Positive. Max: 9.6E7

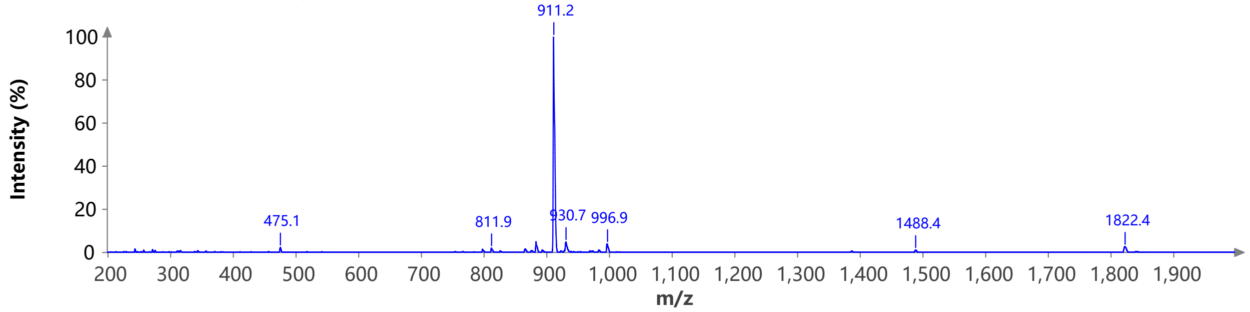

2C8

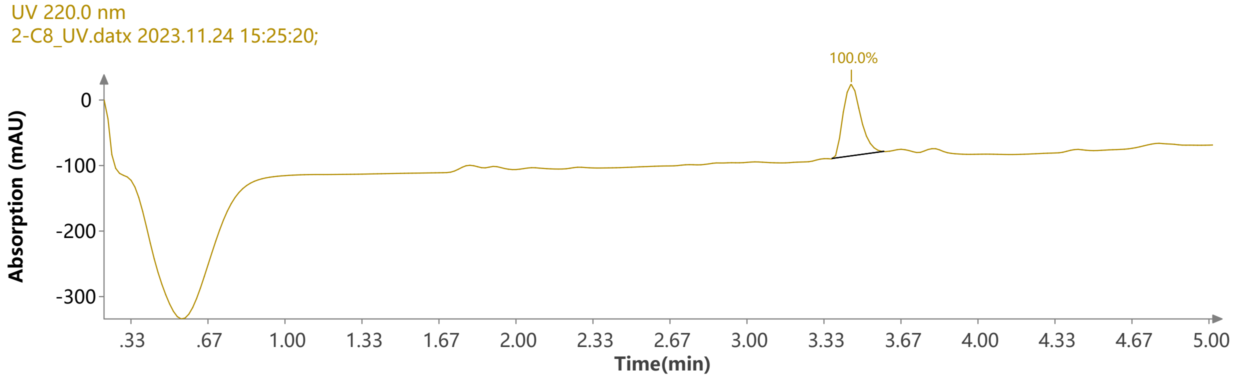
UV 220.0 nm
2-C8_UV.datx 2023.11.24 15:25:20;
100.0%
Absorption (mAU)
0
-100
-200
-300
.33
.67
1.00
1.33
1.67
2.00
2.33
2.67
3.00
3.33
3.67
4.00
4.33
4.67
5.00
Time(min)

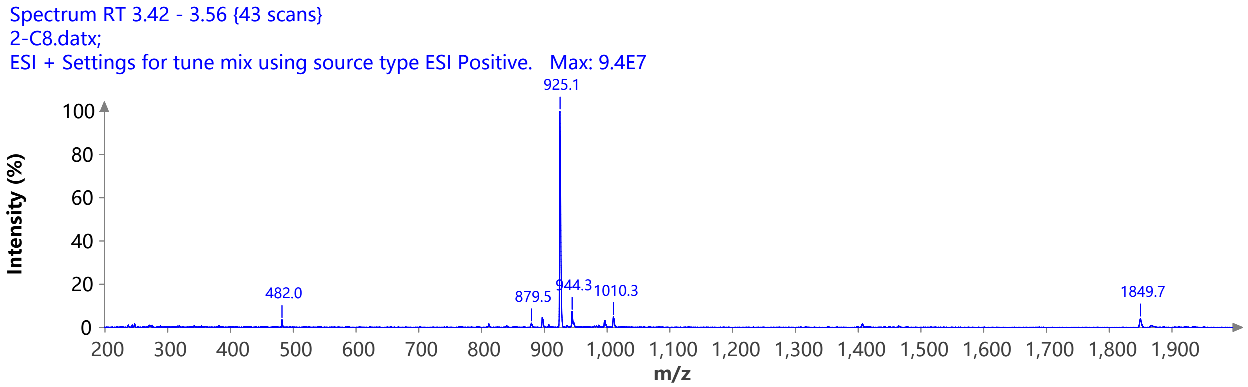
Spectrum RT 3.42 - 3.56 {43 scans}
2-C8.datx;
ESI + Settings for tune mix using source type ESI Positive. Max: 9.4E7
925.1
482.0
879.5
944.3
1010.3
1849.7
Intensity (%)
100
80
60
40
20
0
200
300
400
500
600
700
800
900
1,000
1,100
1,200
1,300
1,400
1,500
1,600
1,700
1,800
1,900
m/z

2C9

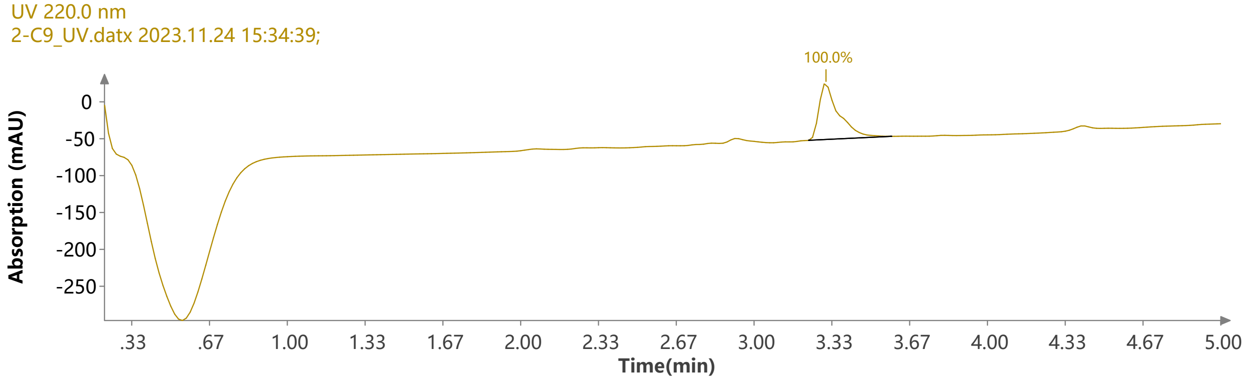
UV 220.0 nm
2-C9_UV.datx 2023.11.24 15:34:39;
100.0%
Absorption (mAU)
0
-50
-100
-150
-200
-250
.33
.67
1.00
1.33
1.67
2.00
2.33
2.67
3.00
3.33
3.67
4.00
4.33
4.67
5.00
Time(min)

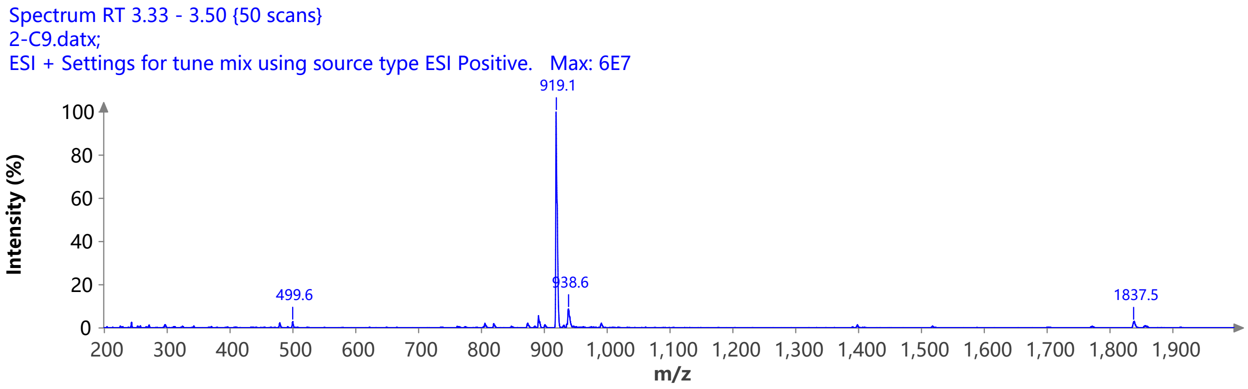
Spectrum RT 3.33 - 3.50 {50 scans}
2-C9.datx;
ESI + Settings for tune mix using source type ESI Positive. Max: 6E7
919.1
499.6
938.6
1837.5
Intensity (%)
100
80
60
40
20
0
200
300
400
500
600
700
800
900
1,000
1,100
1,200
1,300
1,400
1,500
1,600
1,700
1,800
1,900
m/z

2D2

UV 200.0 nm
2-D2_UV.datx 2023.11.24 15:43:58;

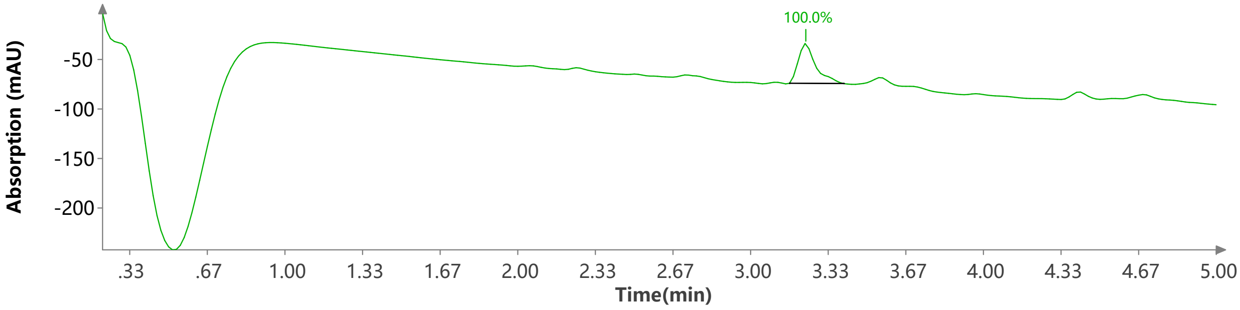

Spectrum RT 3.21 - 3.35 {40 scans}
2-D2.datx;
ESI + Settings for tune mix using source type ESI Positive.   Max: 1.5E7

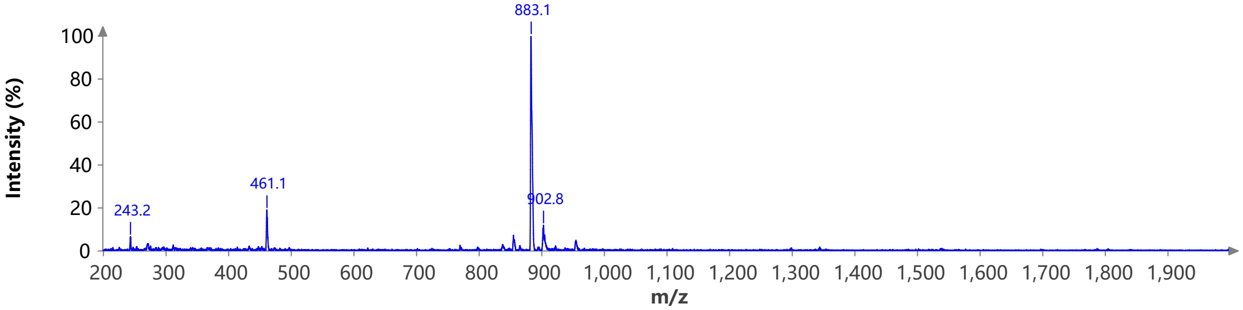

2D6

UV 220.0 nm
2-D6_UV.datx 2023.11.24 15:53:17;

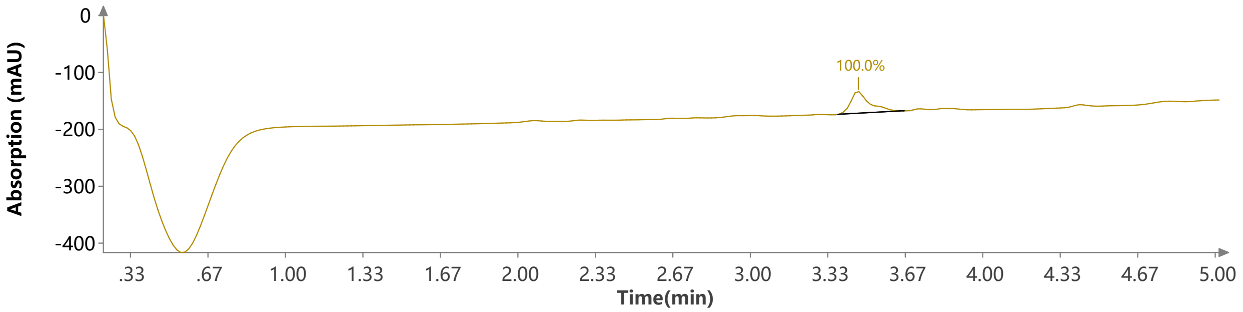

Spectrum RT 3.40 - 3.58 {54 scans}
2-D6.datx;
ESI + Settings for tune mix using source type ESI Positive.   Max: 3.8E7

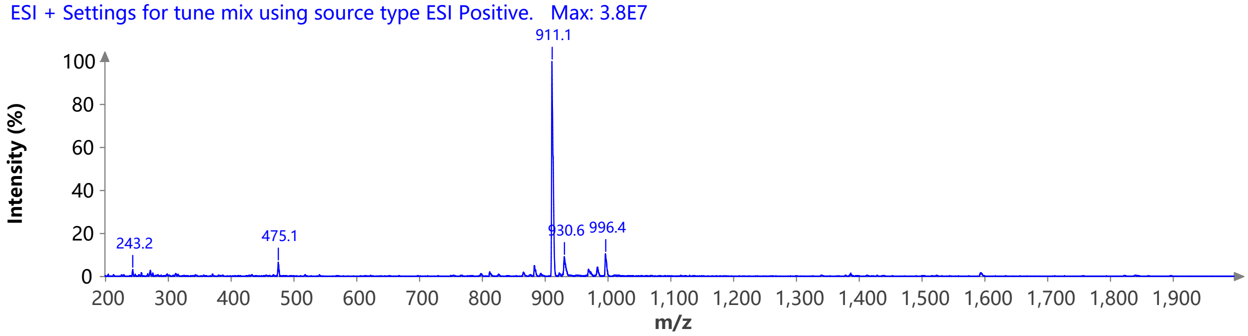

2E5

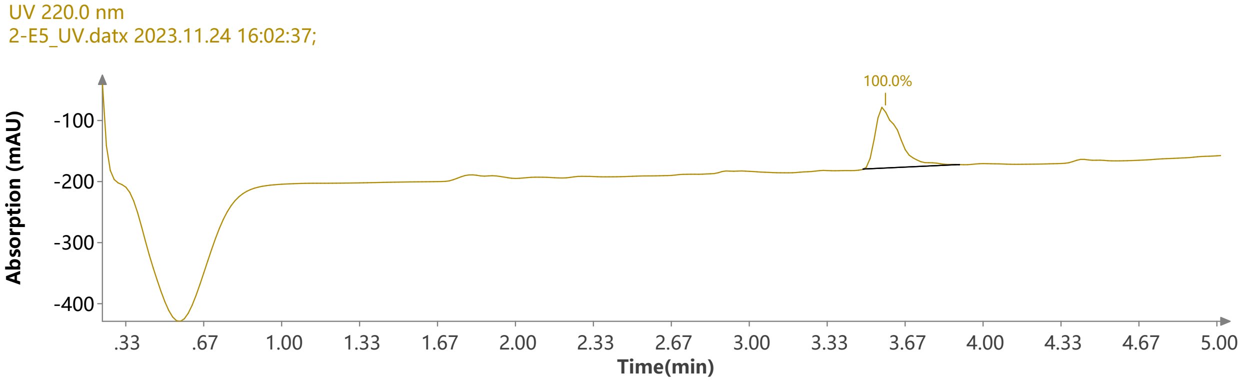

UV 220.0 nm
2-E5_UV.datx 2023.11.24 16:02:37;
100.0%
Absorption (mAU)
-100
-200
-300
-400
.33
.67
1.00
1.33
1.67
2.00
2.33
2.67
3.00
3.33
3.67
4.00
4.33
4.67
5.00
Time(min)

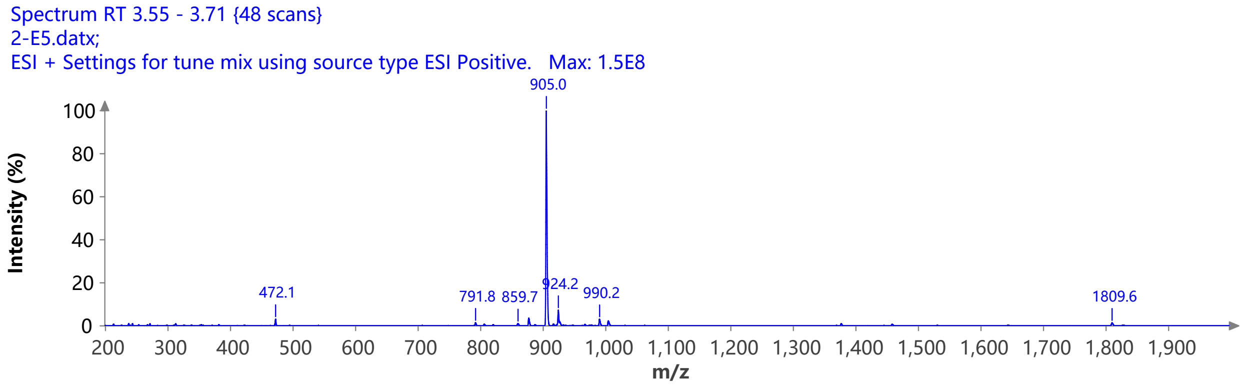

Spectrum RT 3.55 - 3.71 {48 scans}
2-E5.datx;
ESI + Settings for tune mix using source type ESI Positive. Max: 1.5E8
905.0
472.1
791.8
859.7
924.2
990.2
1809.6
Intensity (%)
m/z

2E8

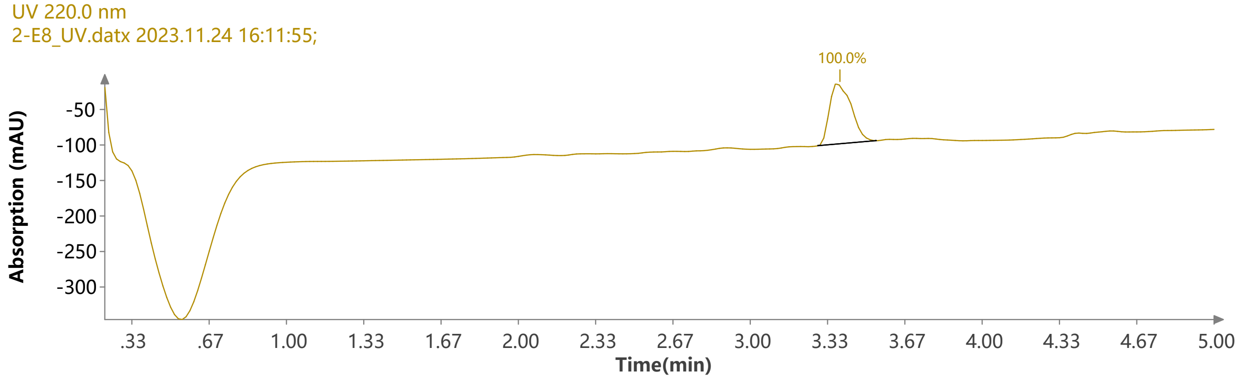

UV 220.0 nm
2-E8_UV.datx 2023.11.24 16:11:55;
100.0%
Absorption (mAU)
Time(min)

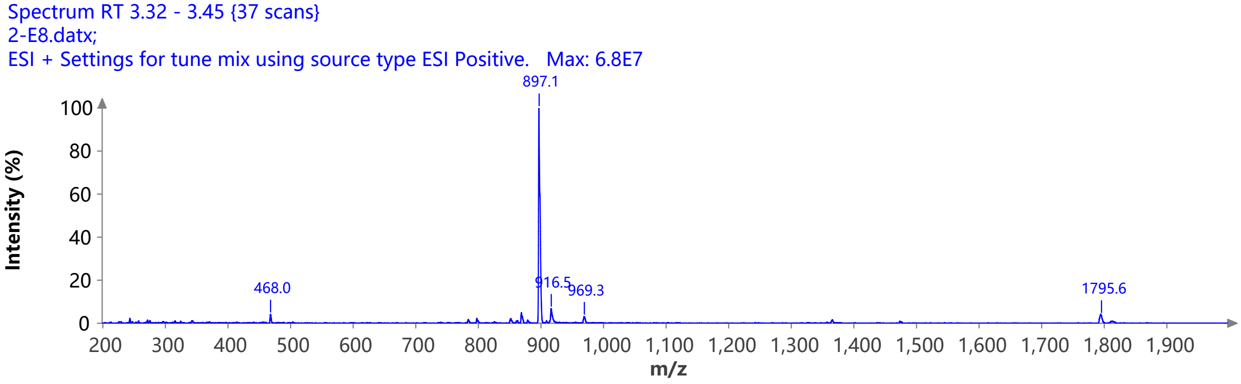

Spectrum RT 3.32 - 3.45 {37 scans}
2-E8.datx;
ESI + Settings for tune mix using source type ESI Positive. Max: 6.8E7
897.1
468.0
916.5
969.3
1795.6
Intensity (%)
m/z

2E11

UV 220.0 nm
2-E11_UV.datx 2023.11.24 16:21:14;

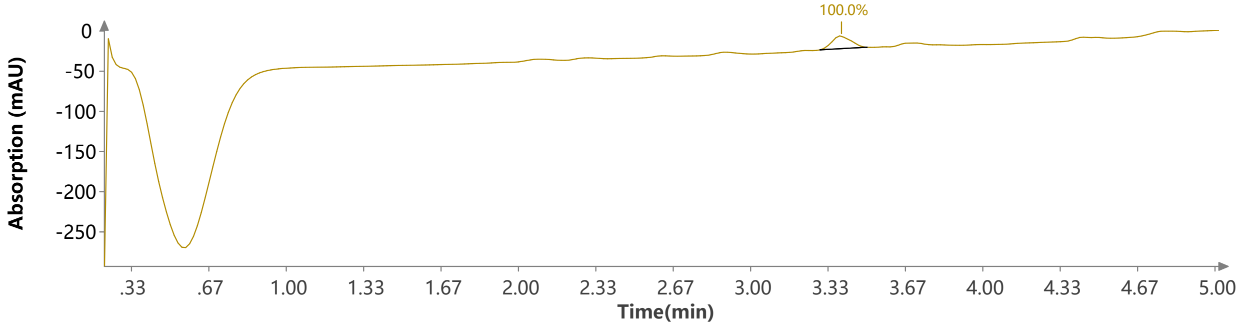

Spectrum RT 3.32 - 3.51 {56 scans}
2-E11.datx;
ESI + Settings for tune mix using source type ESI Positive. Max: 2.2E7

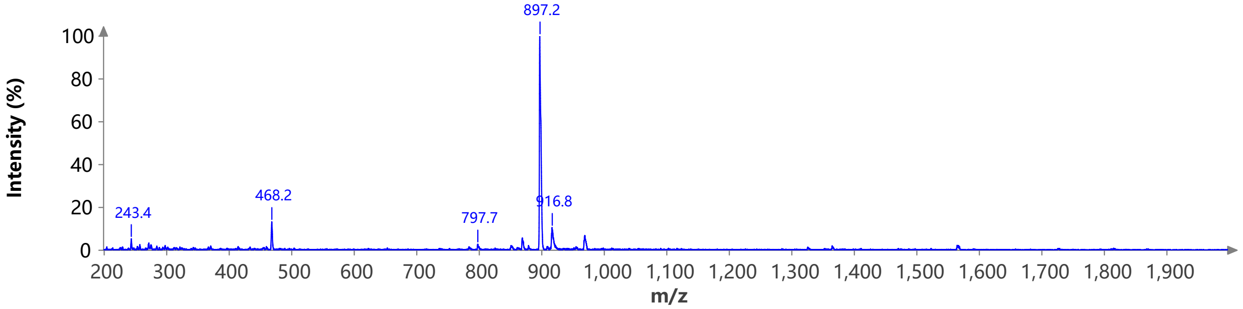

7-A[1]

UV 260.0 nm
JTmorti07_A1_OEt_UV.datx 2023.05.19 09:00:20;

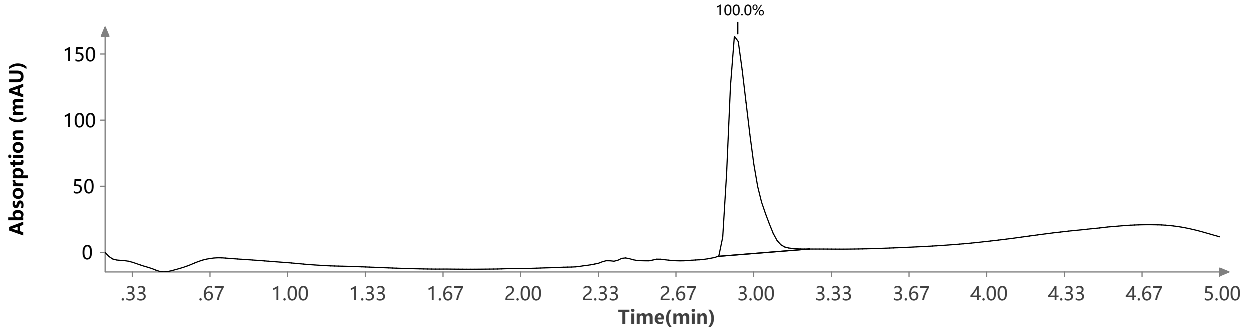

Spectrum RT 2.90 - 3.24 {98 scans}
JTmorti07_A1_OEt.datx;
ESI + Settings for tune mix using source type ESI Positive. Max: 3.8E7

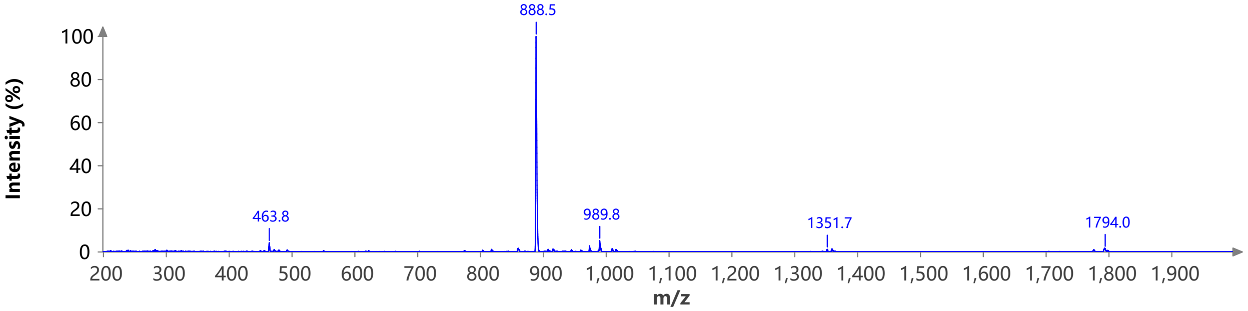

7-A^{2}

UV 260.0 nm
JTmorti07_A2_f4_UV.datx 2023.05.13 15:09:55;

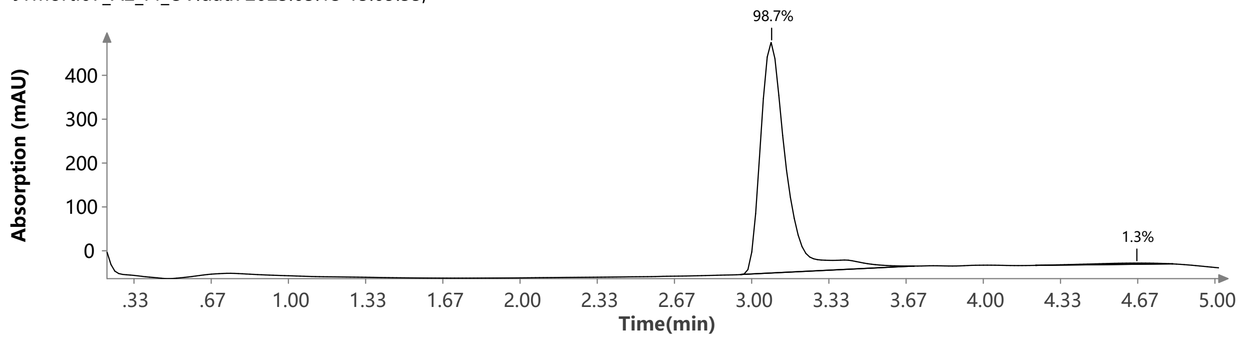

Spectrum RT 3.04 - 3.28 {69 scans}
JTmorti07_A2_f4.datx;
ESI + Settings for tune mix using source type ESI Positive. Max: 6.6E6

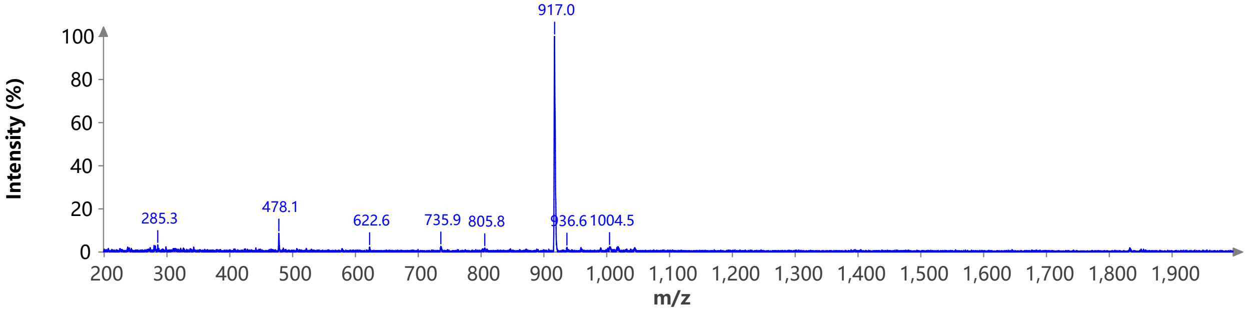

7-A^{3}

UV 260.0 nm
JTmorti07_A3_lyo_UV.datx 2023.05.22 12:21:27;

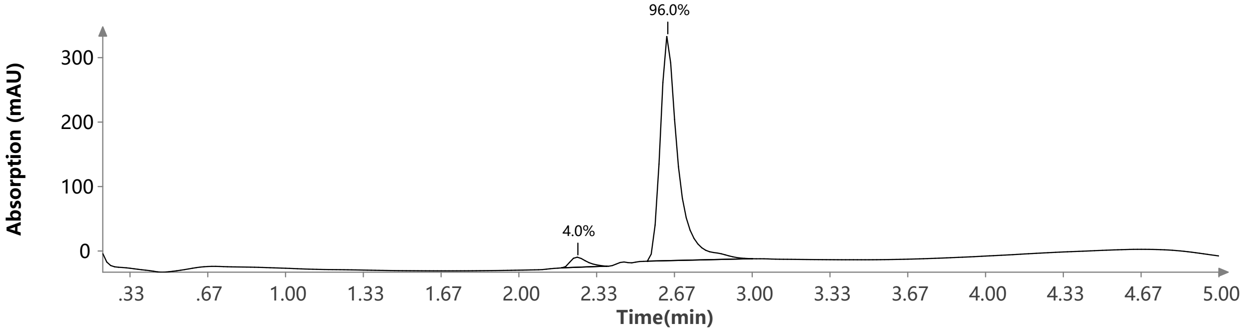

Spectrum RT 2.61 - 2.84 {68 scans}
JTmorti07_A3_lyo.datx;
ESI + Settings for tune mix using source type ESI Positive. Max: 1.9E7

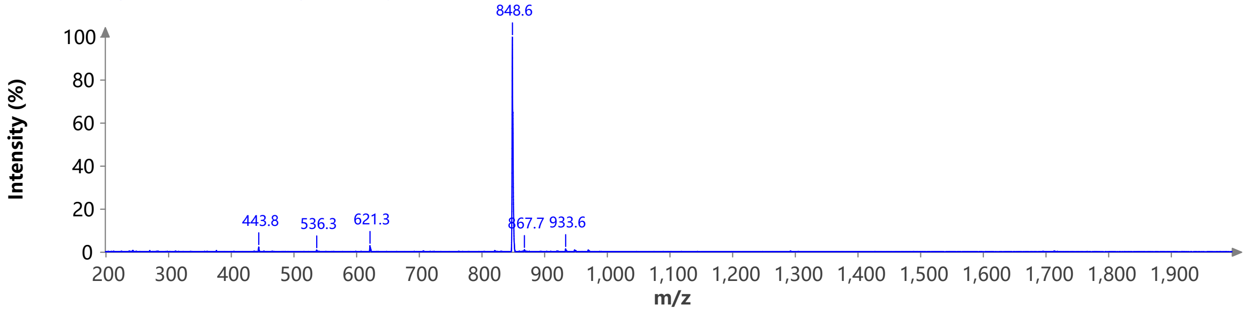

7-A^4

UV 260.0 nm
JTmorti07_A4_lyo_UV.datx 2023.05.22 12:32:40;

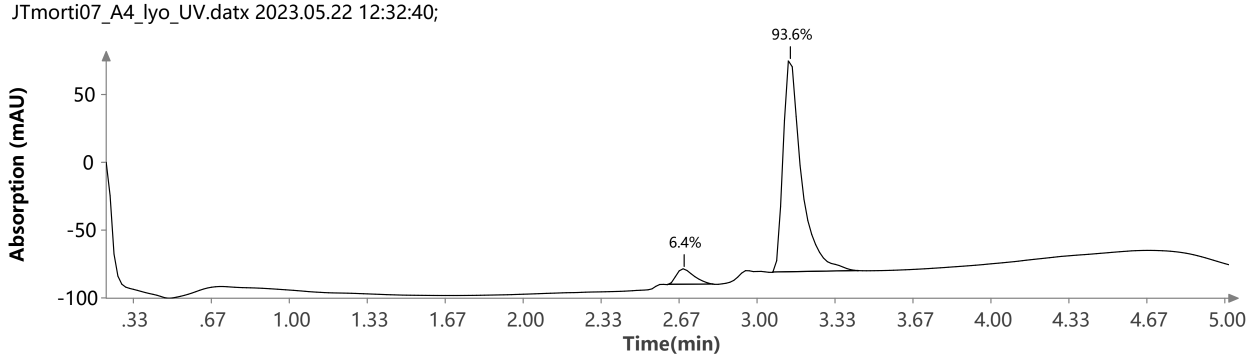

Spectrum RT 3.10 - 3.41 {90 scans}
JTmorti07_A4_lyo.datx;
ESI + Settings for tune mix using source type ESI Positive. Max: 5.8E6

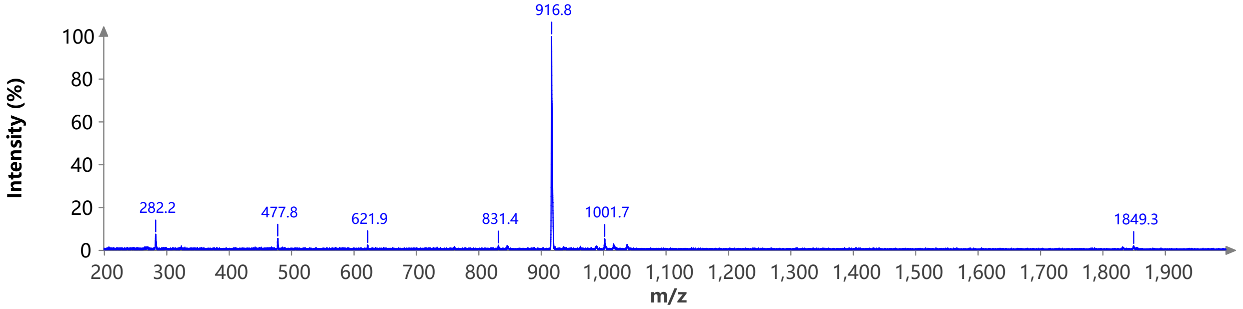

7-A^5

UV 260.0 nm
JTmorti07_A5_lyo_UV.datx 2023.05.22 12:43:59;

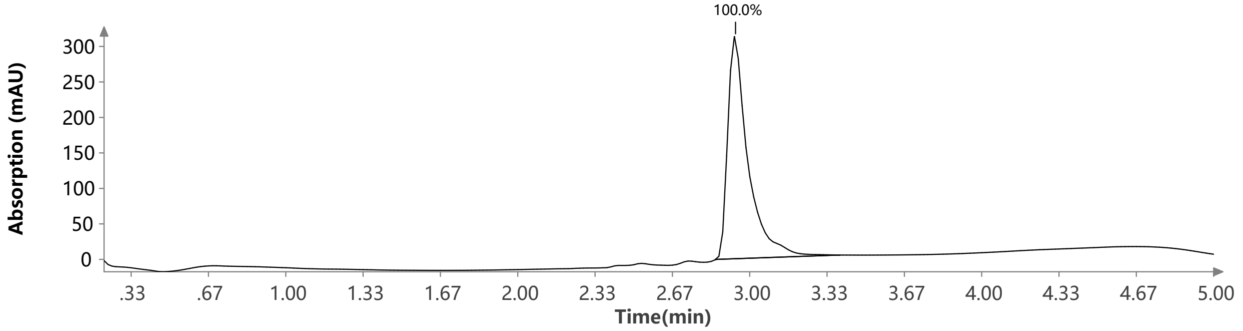

Spectrum RT 2.96 - 3.22 {76 scans}
JTmorti07_A5_lyo.datx;
ESI + Settings for tune mix using source type ESI Positive. Max: 1.1E7

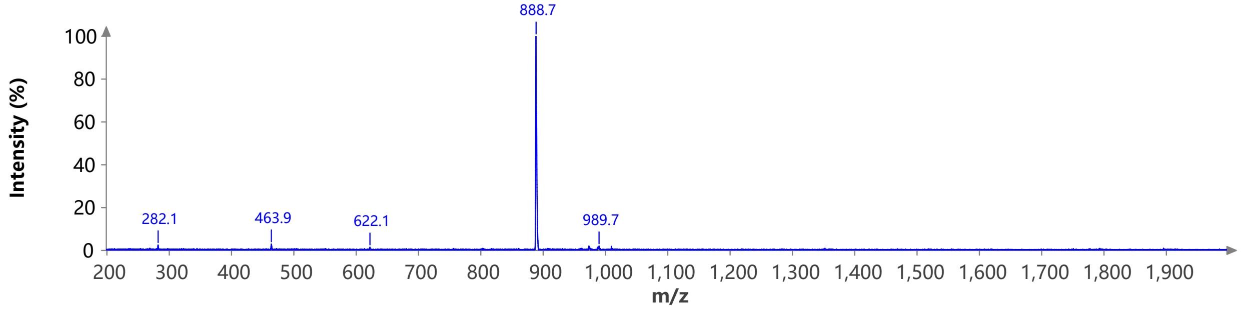

7-A[6]

UV 200.0 nm
JTmorti07_A6_lyo_UV.datx 2023.05.22 12:55:13;

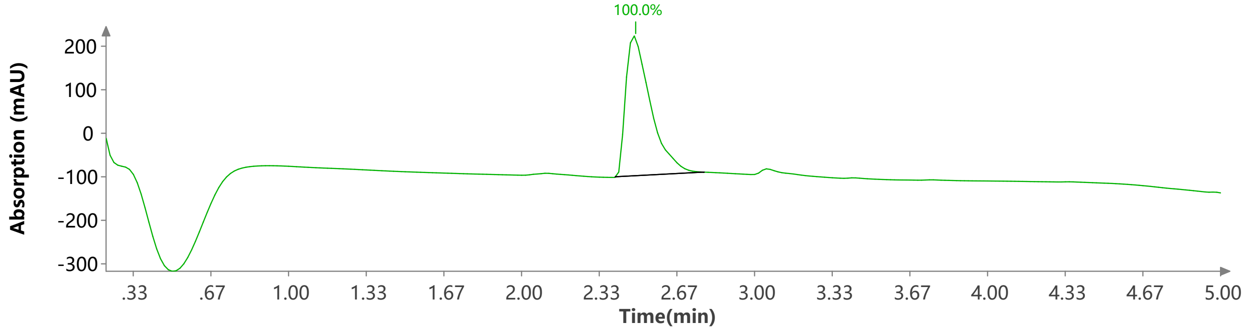

Spectrum RT 2.44 - 2.79 {101 scans}
JTmorti07_A6_lyo.datx;
ESI + Settings for tune mix using source type ESI Positive. Max: 2.7E7

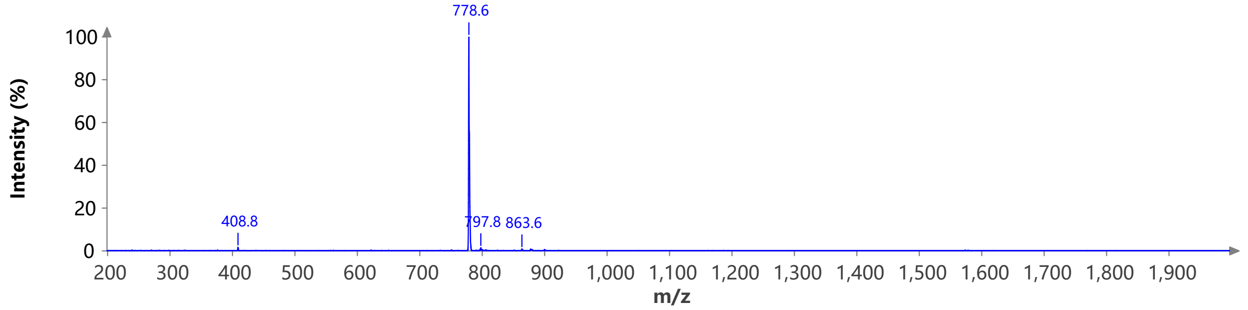

7-A[7]

UV 260.0 nm
JTmorti07_A7_lyo_UV.datx 2023.05.17 09:20:30;

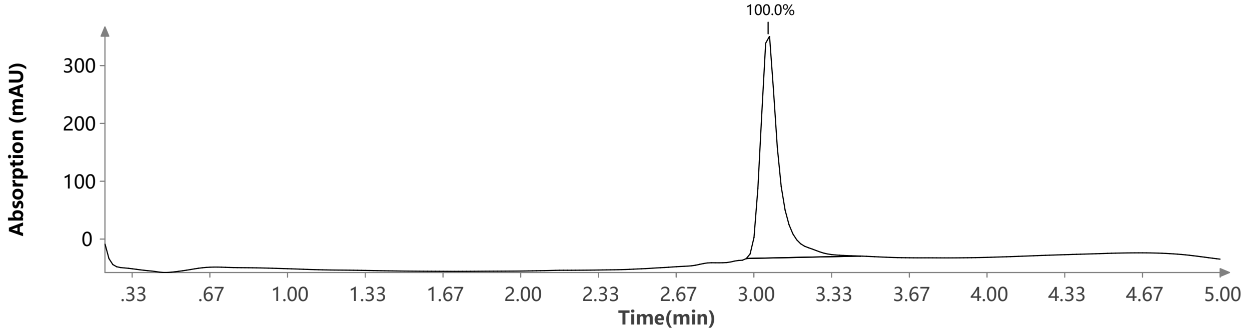

Spectrum RT 3.06 - 3.27 {62 scans}
JTmorti07_A7_lyo.datx;
ESI + Settings for tune mix using source type ESI Positive. Max: 1.1E8

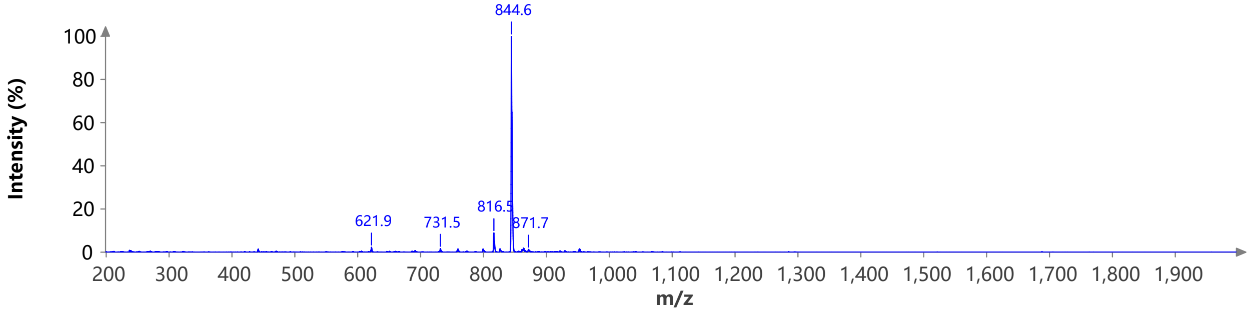

7-Hse²

UV 260.0 nm
JTmorti07_HSe2_f4_UV.datx 2023.05.13 15:19:10;

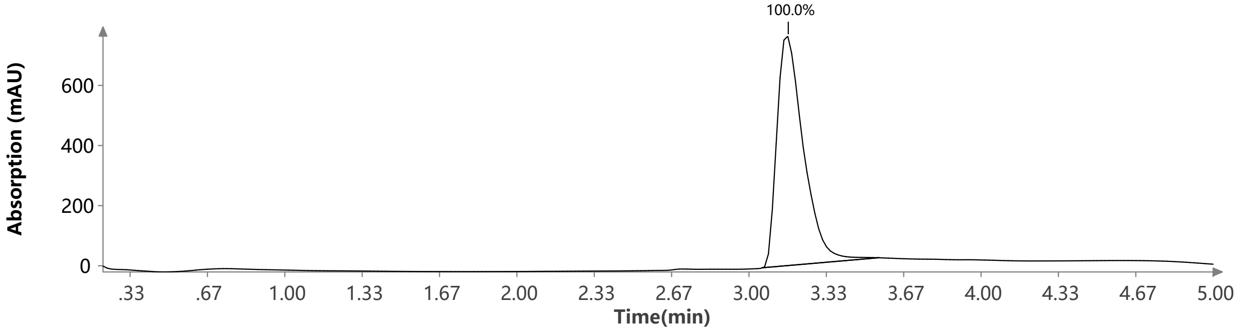

Spectrum RT 3.15 - 3.44 {84 scans}
JTmorti07_HSe2_f4.datx;
ESI + Settings for tune mix using source type ESI Positive. Max: 1E7

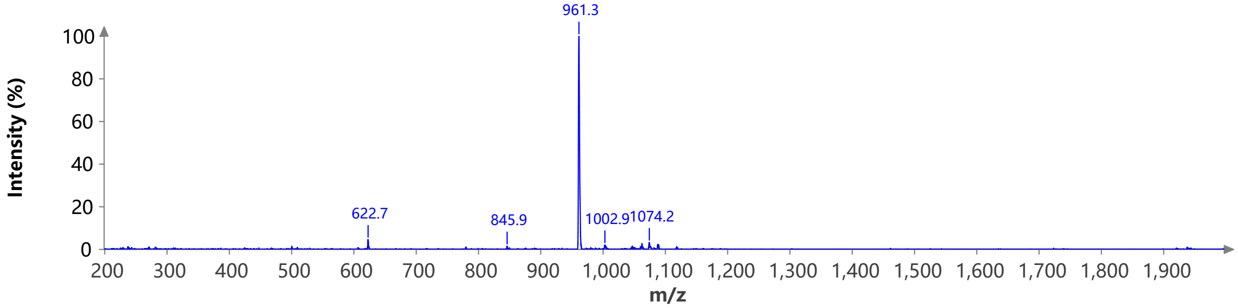

7-Hse⁴

UV 260.0 nm
JTmorti07_Hse4_f5_UV.datx 2023.05.19 12:30:10;

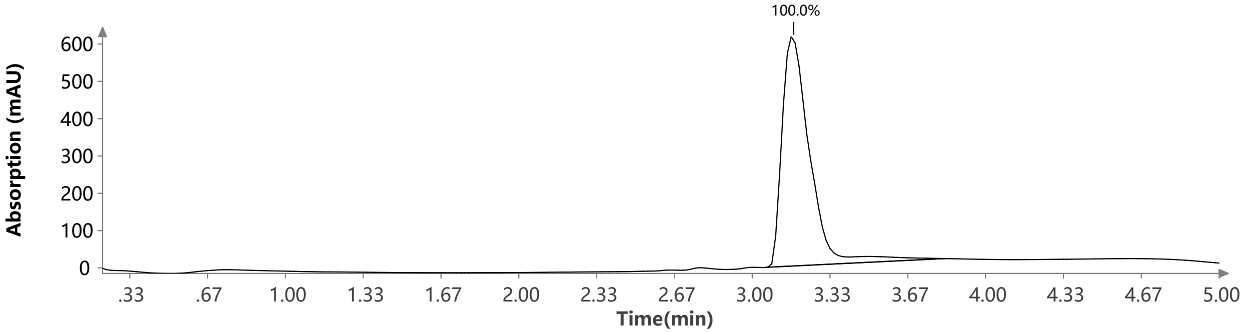

Spectrum RT 3.13 - 3.52 {114 scans}
JTmorti07_Hse4_f5.datx;
ESI + Settings for tune mix using source type ESI Positive. Max: 7.6E7

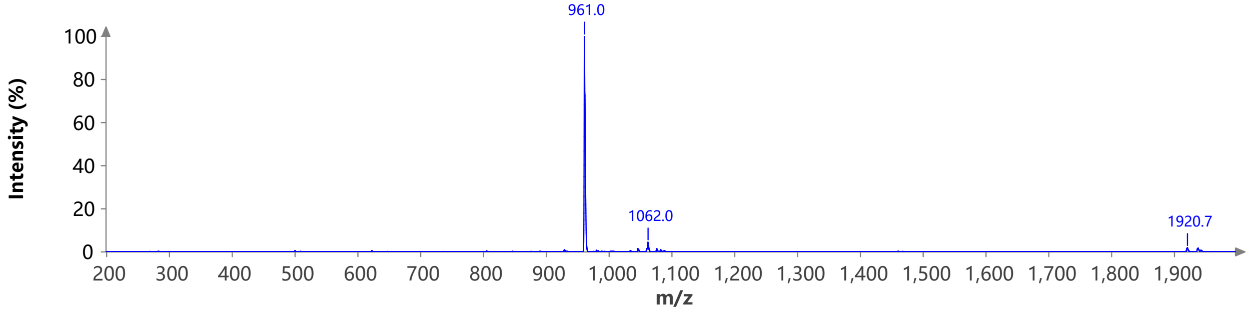

7-Hse[24]

UV 260.0 nm
JTmorti07_HSe24_f5_UV.datx 2023.05.13 15:28:30;

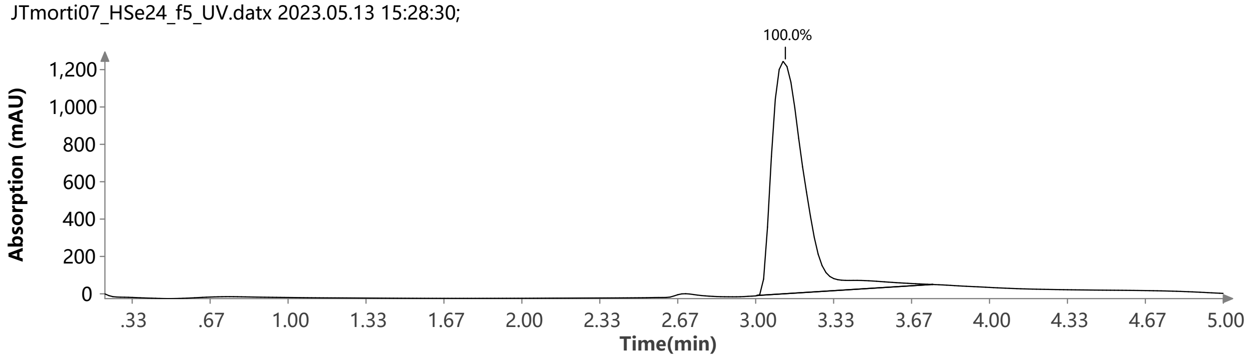

Spectrum RT 3.06 - 3.48 {118 scans}
JTmorti07_HSe24_f5.datx;
ESI + Settings for tune mix using source type ESI Positive. Max: 1.5E7

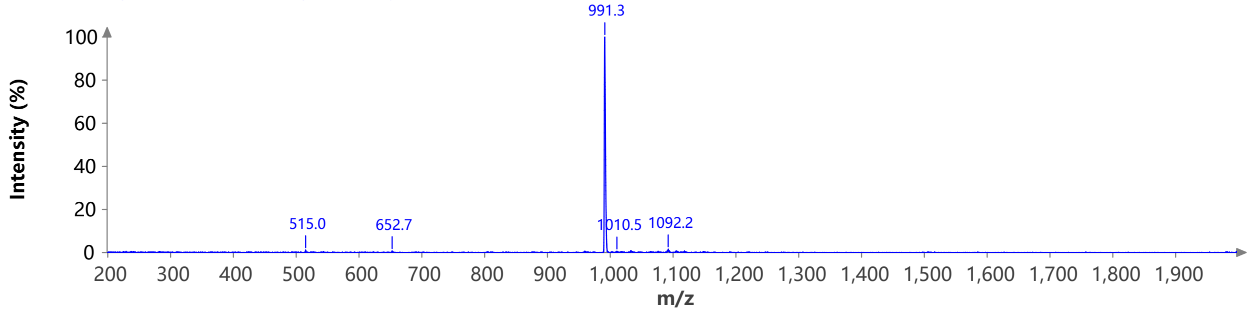

7-A[17]

UV 260.0 nm
JTmorti07_A17_f9_UV.datx 2023.05.14 13:20:06;

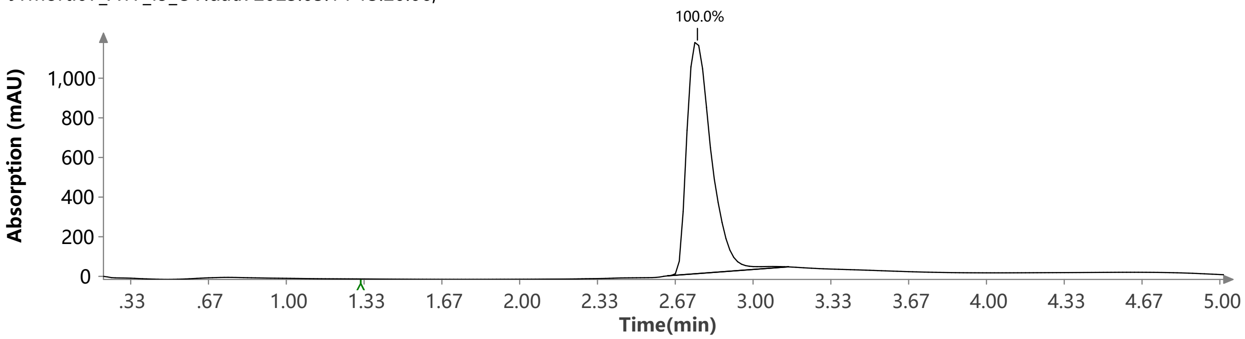

Spectrum RT 2.72 - 3.35 {181 scans}
JTmorti07_A17_f9.datx;
ESI + Settings for tune mix using source type ESI Positive. Max: 3E7

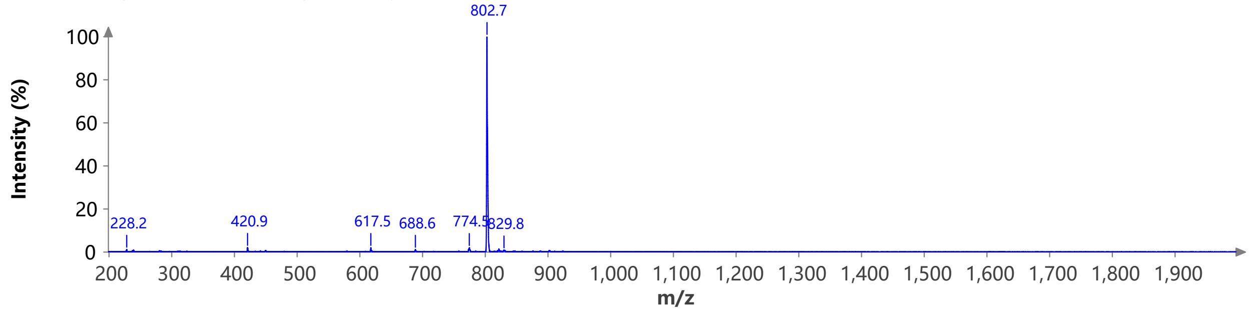

7-Hse^{2}A^7

UV 260.0 nm
JTmorti07_Hse2A7_lyo_UV.datx 2023.05.12 16:52:14;

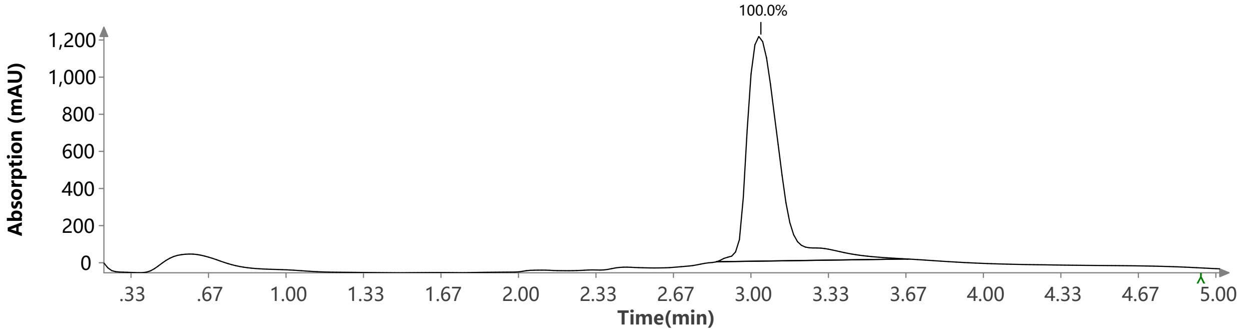

Spectrum RT 2.98 - 3.25 {78 scans}
JTmorti07_Hse2A7_lyo.datx;
ESI + Settings for tune mix using source type ESI Positive.   Max: 4.5E7

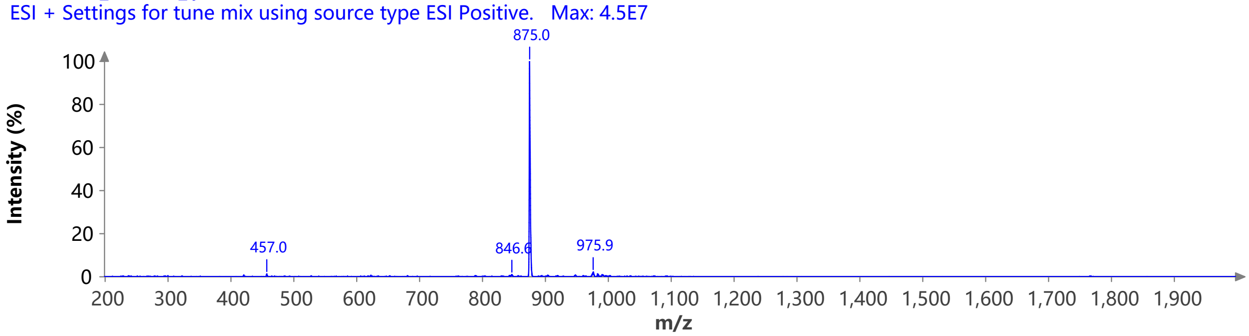

7-A^{57}

UV 260.0 nm
JTmorti07_A57_lyo_UV.datx 2023.05.15 10:44:46;

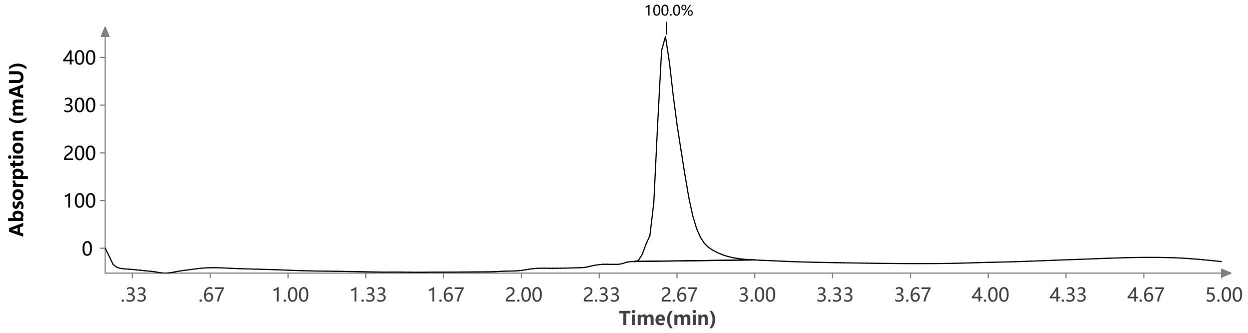

Spectrum RT 2.61 - 2.89 {81 scans}
JTmorti07_A57_lyo.datx;
ESI + Settings for tune mix using source type ESI Positive.   Max: 3.3E7

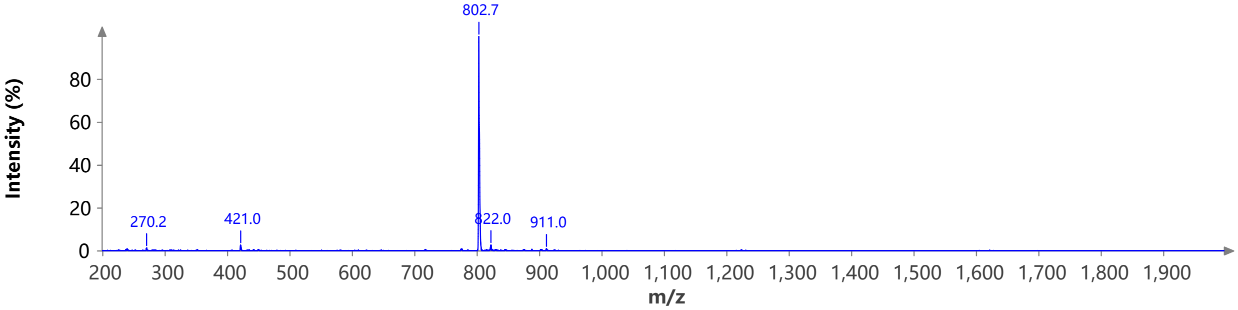

7-Hse⁴A⁷

UV 260.0 nm
JTmorti07_Hse4A7_lyo_UV.datx 2023.05.12 17:01:32;

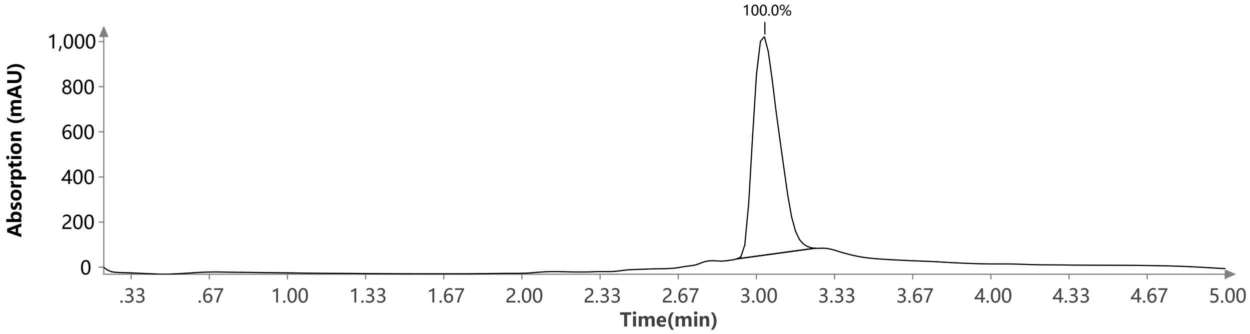

Spectrum RT 3.02 - 3.37 {100 scans}
JTmorti07_Hse4A7_lyo.datx;
ESI + Settings for tune mix using source type ESI Positive.   Max: 3.1E7

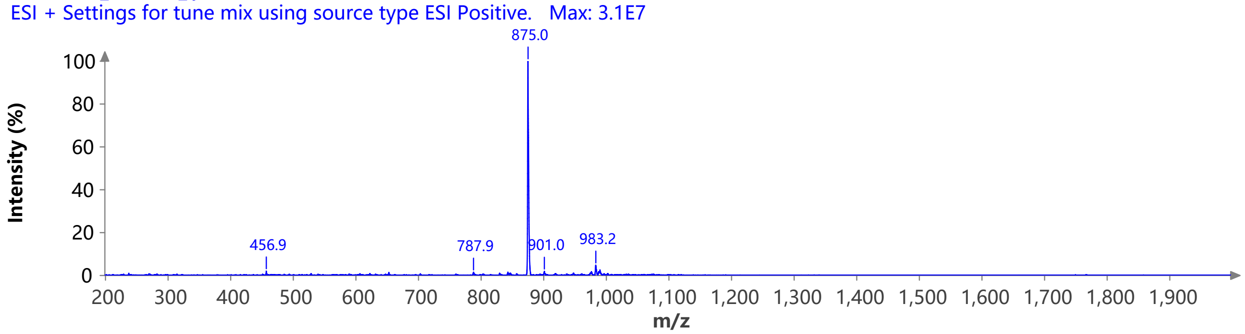

7-S²

UV 260.0 nm
JTmorti07_S2_f4_UV.datx 2023.05.13 15:37:47;

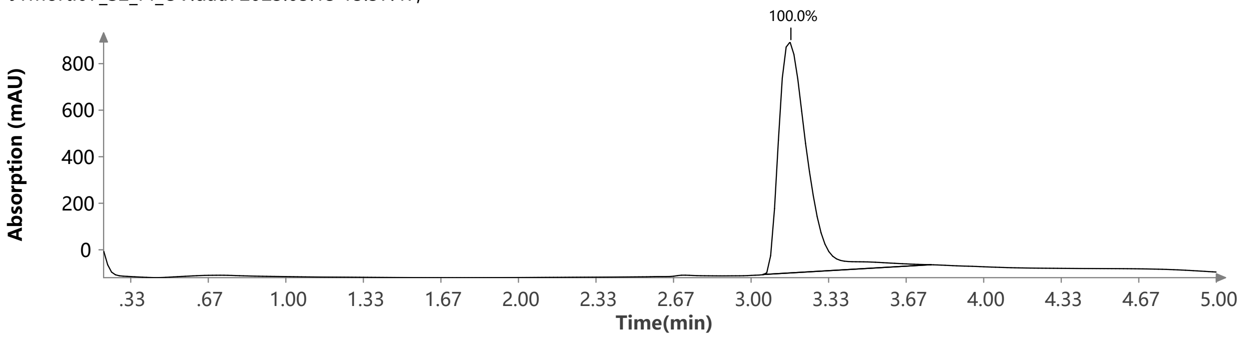

Spectrum RT 3.12 - 3.33 {59 scans}
JTmorti07_S2_f4.datx;
ESI + Settings for tune mix using source type ESI Positive.   Max: 1.4E7

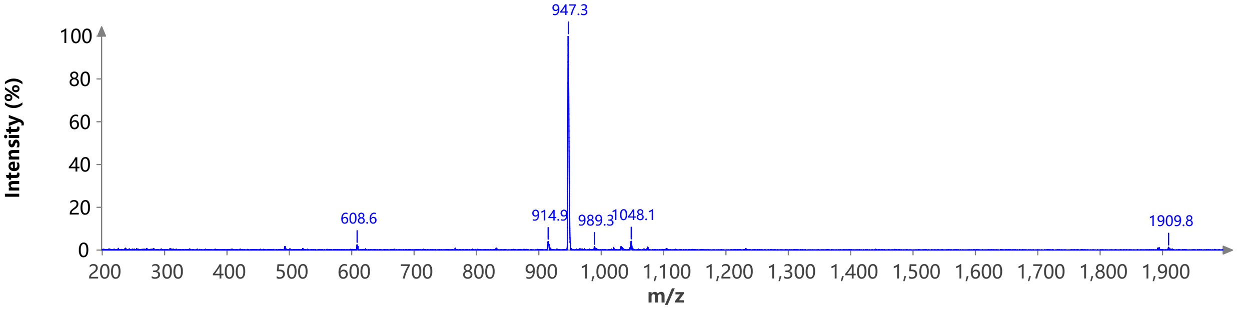

7-S[4]

UV 260.0 nm
JTmorti07_S4_lyo_UV.datx 2023.05.22 13:17:49;

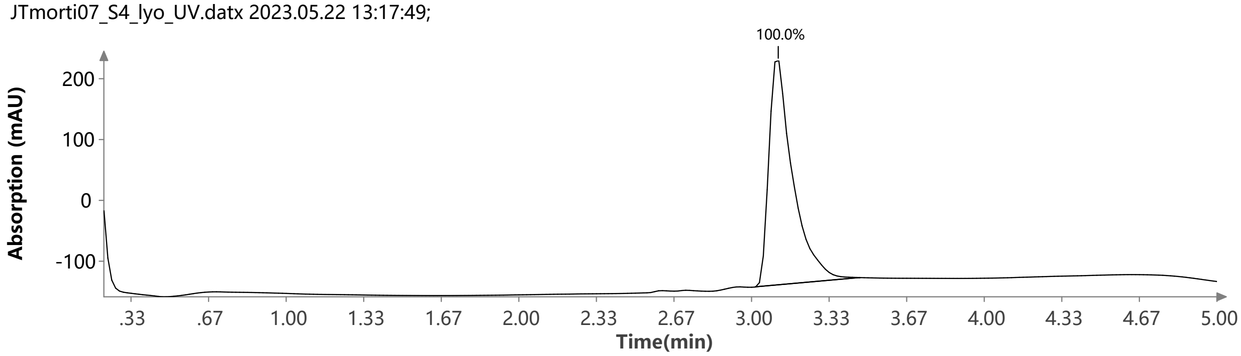

Spectrum RT 3.13 - 3.54 {117 scans}
JTmorti07_S4_lyo.datx;
ESI + Settings for tune mix using source type ESI Positive. Max: 7.9E6

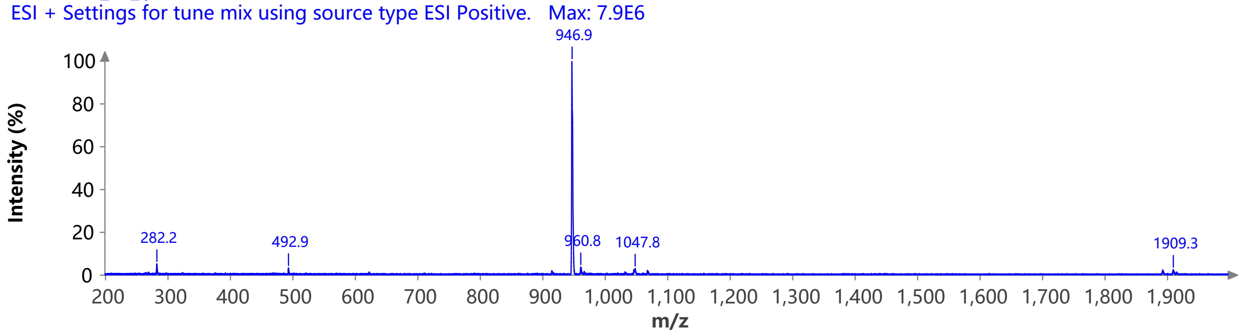

7-S[2]A[7]

UV 260.0 nm
JTmorti07_S2A7_lyo_UV.datx 2023.05.12 16:33:42;

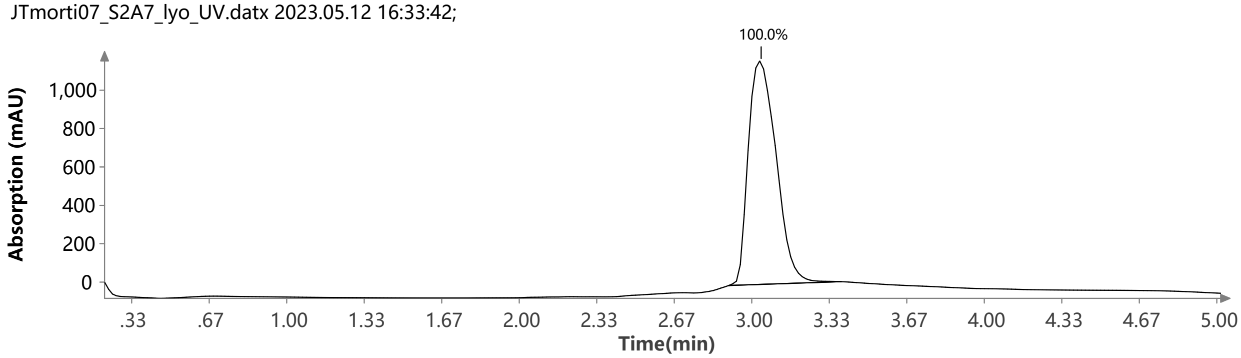

Spectrum RT 3.02 - 3.25 {67 scans}
JTmorti07_S2A7_lyo.datx;
ESI + Settings for tune mix using source type ESI Positive. Max: 4.3E7

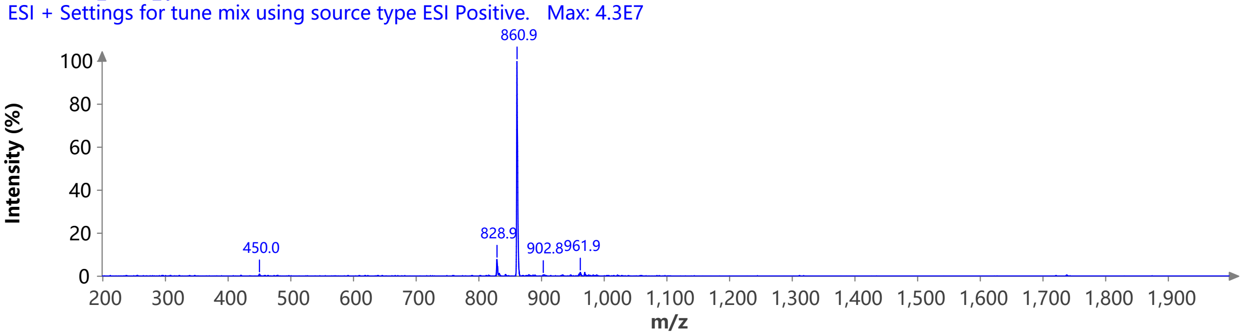

7-S^{4}A^7

UV 260.0 nm
JTmorti07_S4A7_lyo_UV.datx 2023.05.12 16:43:01;

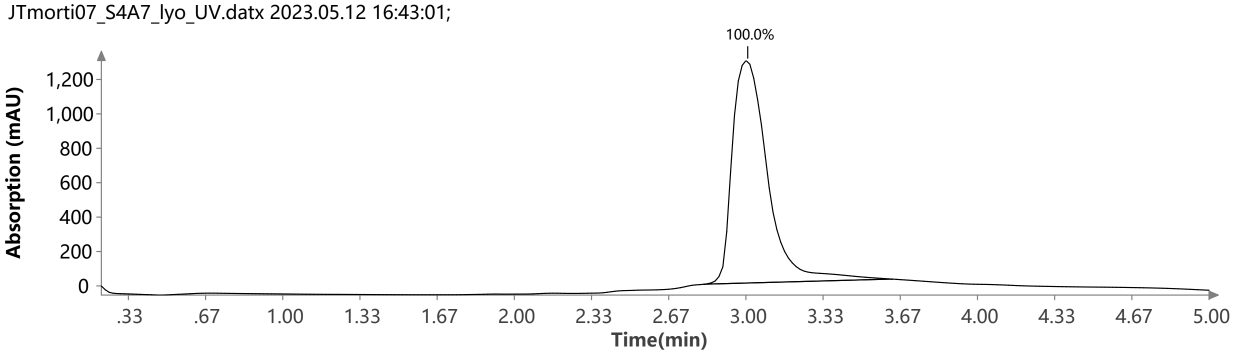

Spectrum RT 2.91 - 3.29 {107 scans}
JTmorti07_S4A7_lyo.datx;
ESI + Settings for tune mix using source type ESI Positive. Max: 3.5E7

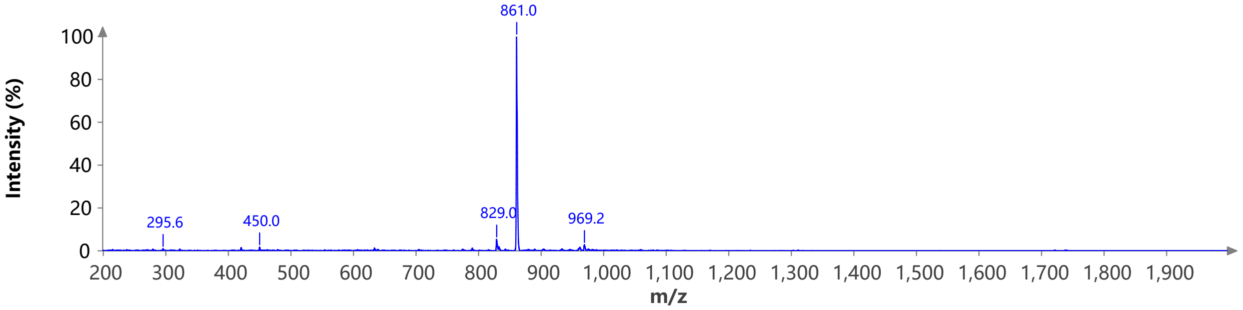

7-S^{2}Pye^7

UV 260.0 nm
JTmorti07_S2Py7_lyo_UV.datx 2023.06.05 12:39:01;

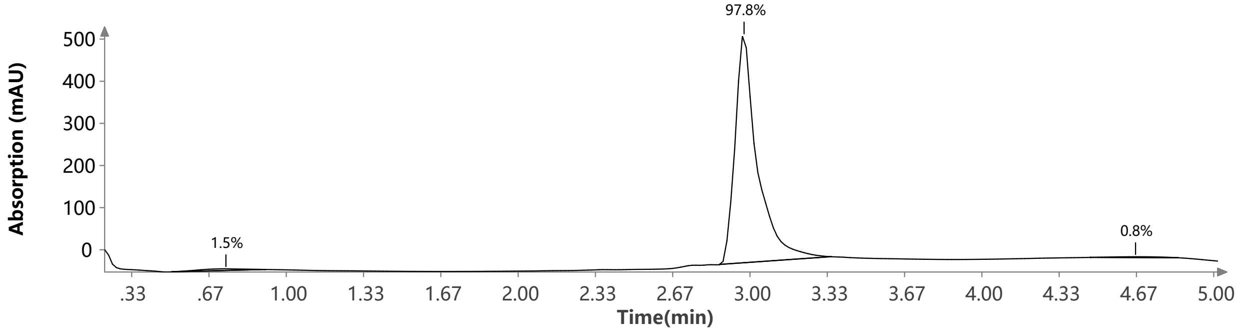

Spectrum RT 2.97 - 3.35 {108 scans}
JTmorti07_S2Py7_lyo.datx;
ESI + Settings for tune mix using source type ESI Positive. Max: 4E6

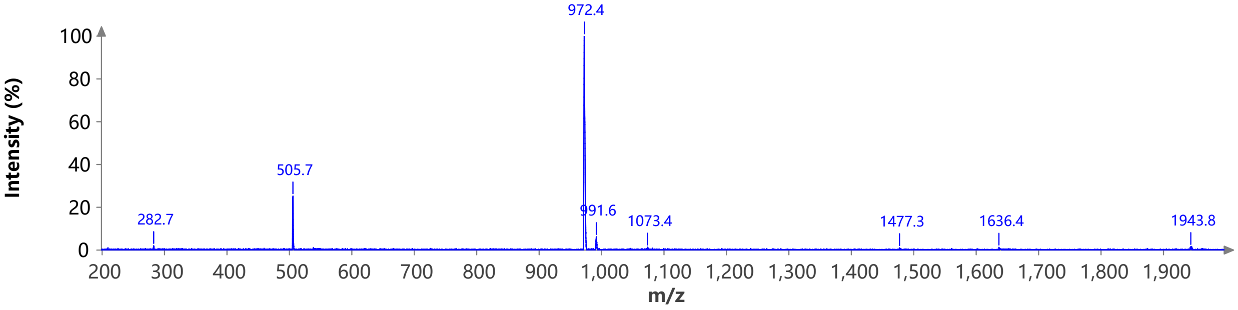

7-A¹Pye⁷

UV 260.0 nm
JTmorti07_A1Py7_lyo_UV.datx 2023.06.05 12:57:49;

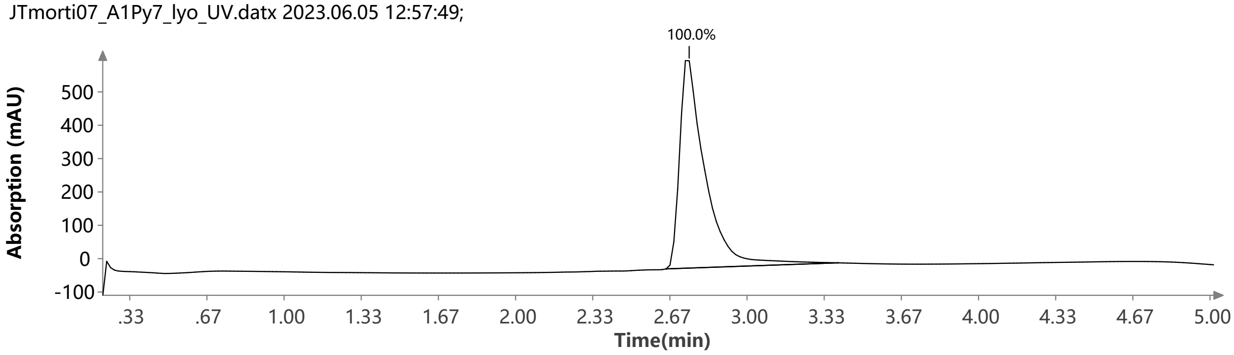

Spectrum RT 2.63 - 2.94 {89 scans}
JTmorti07_A1Py7_lyo.datx;
ESI + Settings for tune mix using source type ESI Positive. Max: 4.5E6

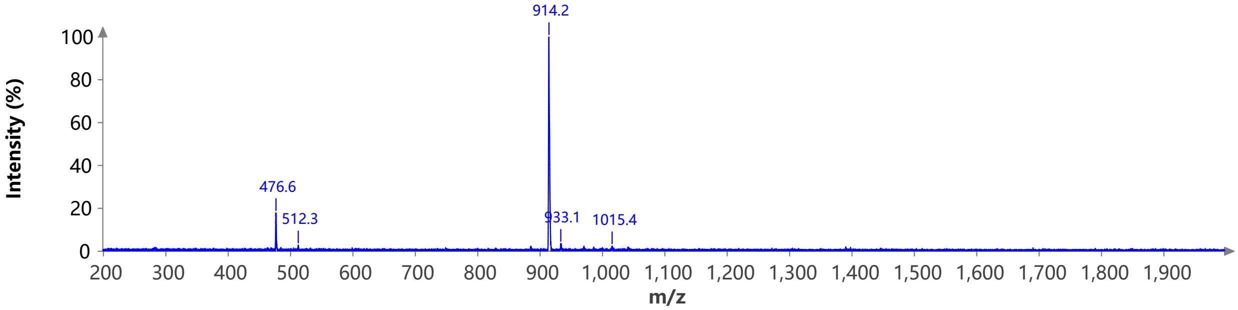

7-Hse²Pye⁷

UV 260.0 nm
JTmorti07_Hse2Py7_lyo_UV.datx 2023.06.05 12:48:27;

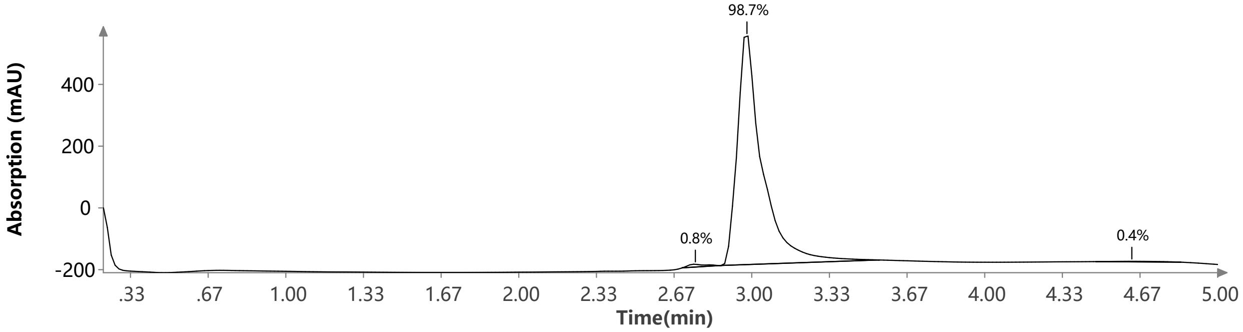

Spectrum RT 2.92 - 3.24 {92 scans}
JTmorti07_Hse2Py7_lyo.datx;
ESI + Settings for tune mix using source type ESI Positive. Max: 5.9E6

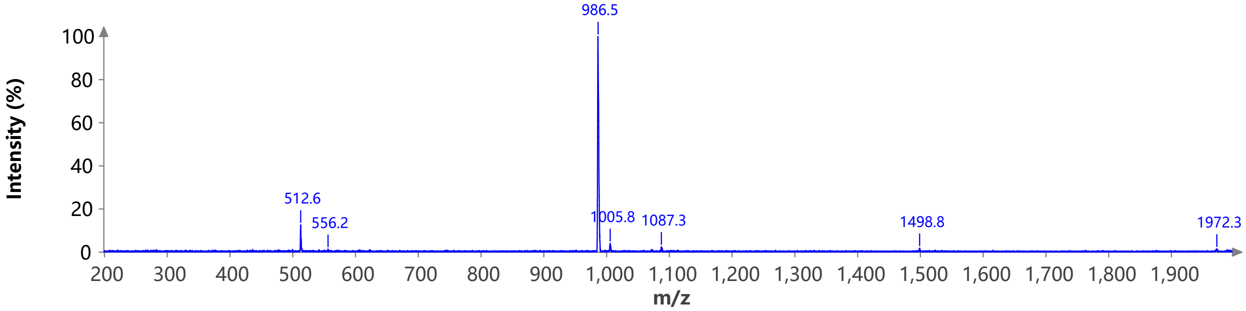

7-A⁵Pye⁷

UV 260.0 nm
JTmorti07_A5Py7_lyo_UV.datx 2023.06.05 12:20:10;

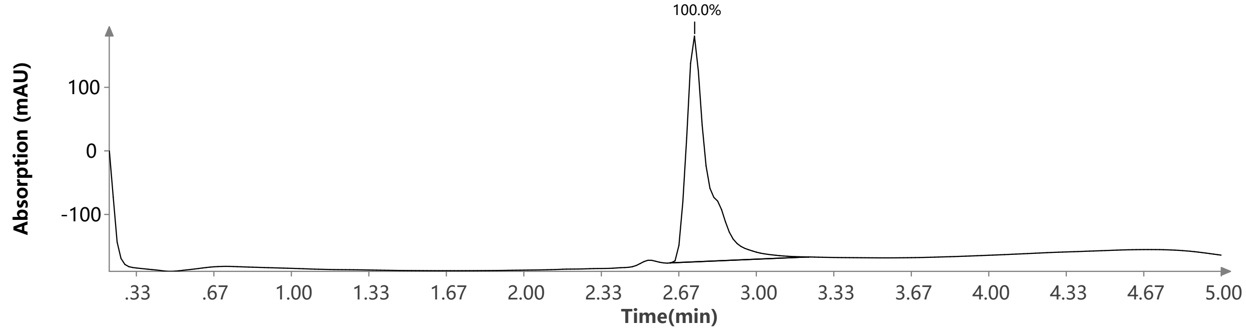

Spectrum RT 2.62 - 3.00 {108 scans}
JTmorti07_A5Py7_lyo.datx;
ESI + Settings for tune mix using source type ESI Positive. Max: 3.2E6

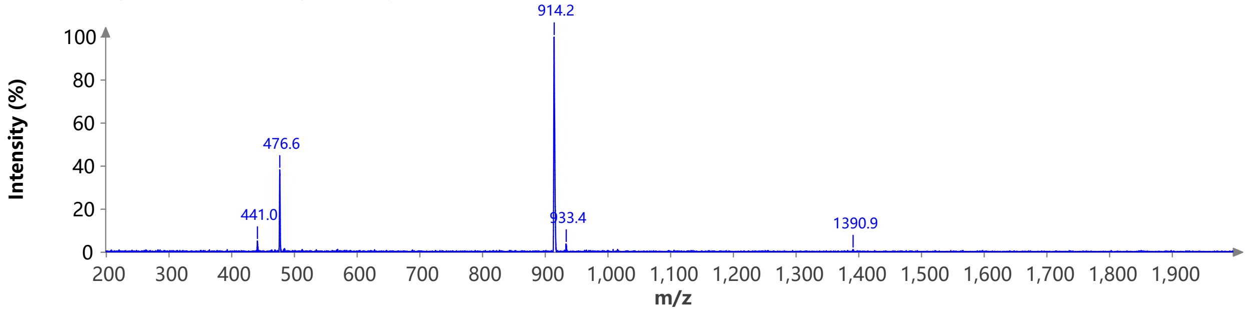

7-S⁴Pye⁷

UV 260.0 nm
JTmorti07_S4Py7_lyo_UV.datx 2023.06.05 12:29:36;

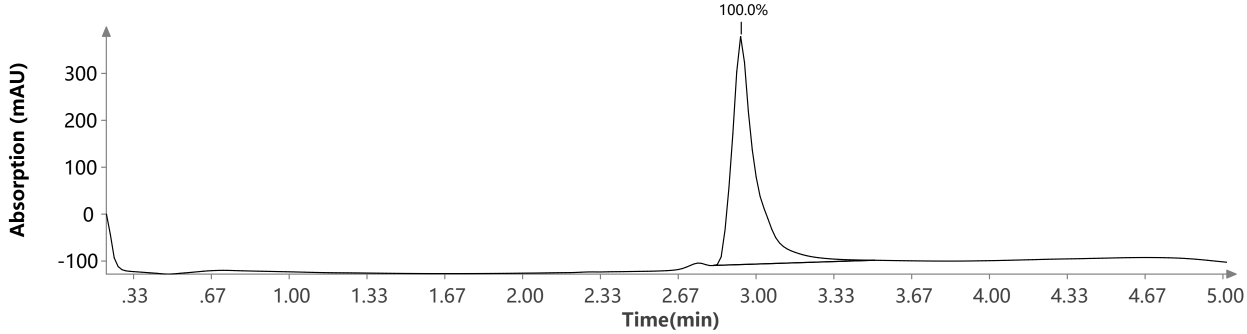

Spectrum RT 2.90 - 3.32 {118 scans}
JTmorti07_S4Py7_lyo.datx;
ESI + Settings for tune mix using source type ESI Positive. Max: 3.7E6

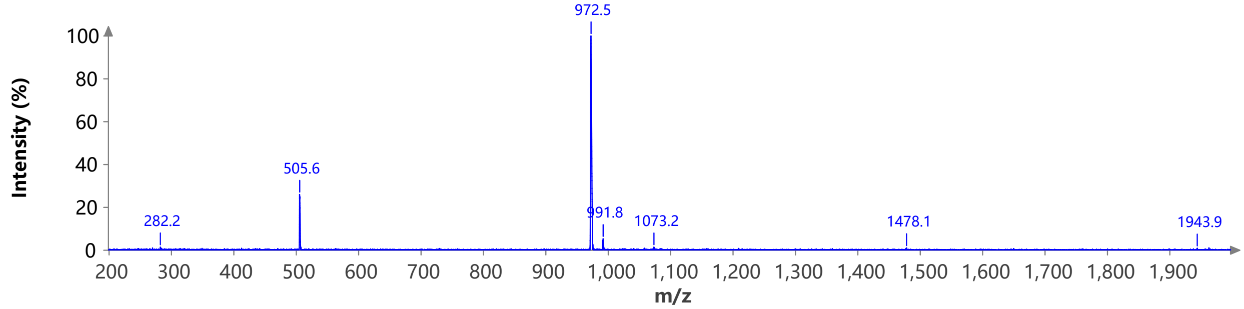

7-Hse^{4}Pye^{7}

UV 260.0 nm
JTmorti07_Hse4Py7_lyo_UV.datx 2023.06.05 13:45:25;

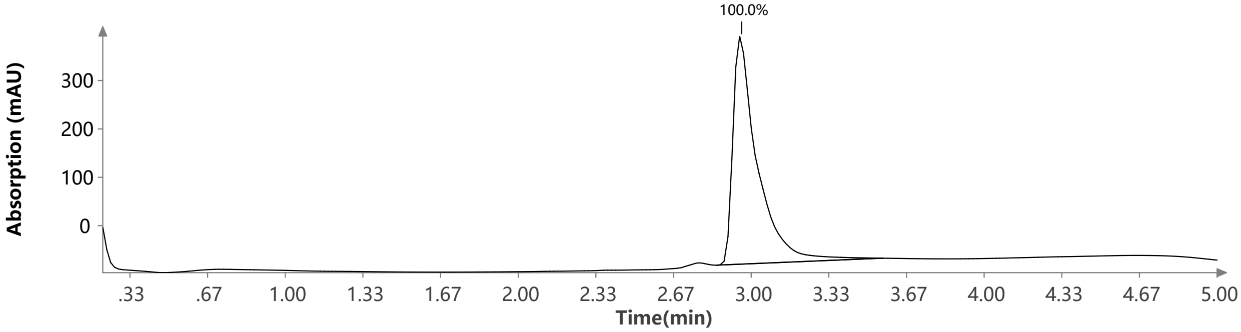

Spectrum RT 2.91 - 3.30 {113 scans}
JTmorti07_Hse4Py7_lyo.datx;
ESI + Settings for tune mix using source type ESI Positive. Max: 5E6

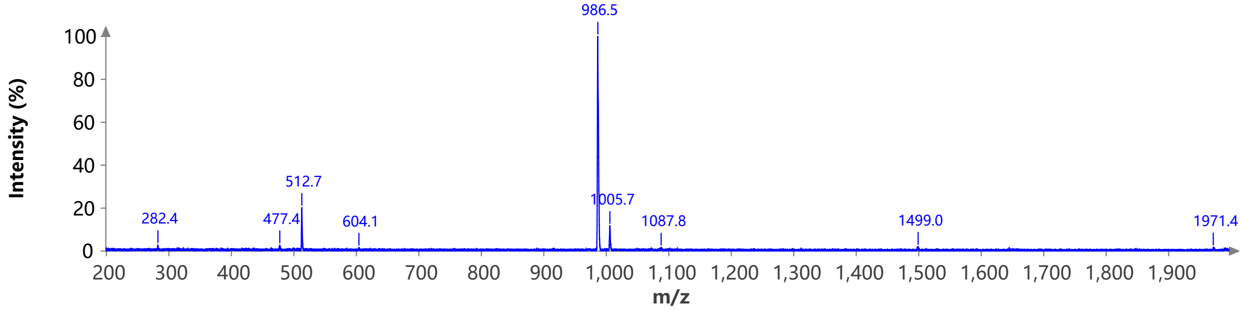

7-S^{2}NMe$_{2}$^{7}

UV 260.0 nm
JTmorti07_S2NMe7_lyo_UV.datx 2023.06.05 13:35:56;

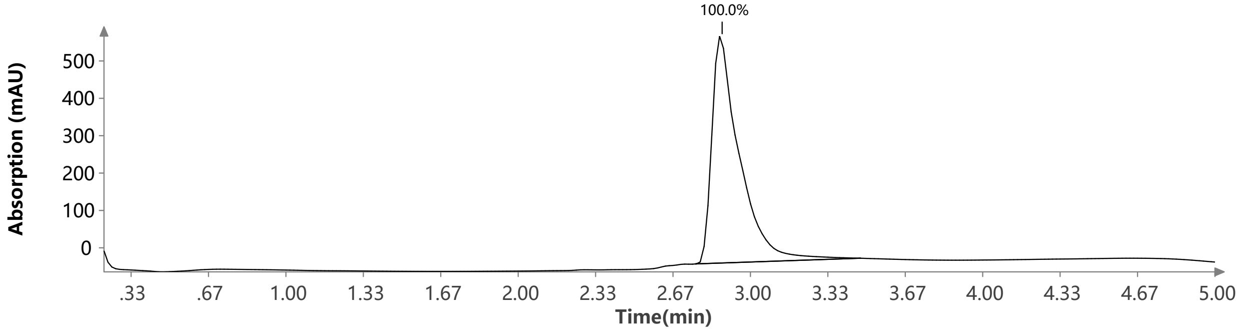

Spectrum RT 2.81 - 3.21 {114 scans}
JTmorti07_S2NMe7_lyo.datx;
ESI + Settings for tune mix using source type ESI Positive. Max: 5E6

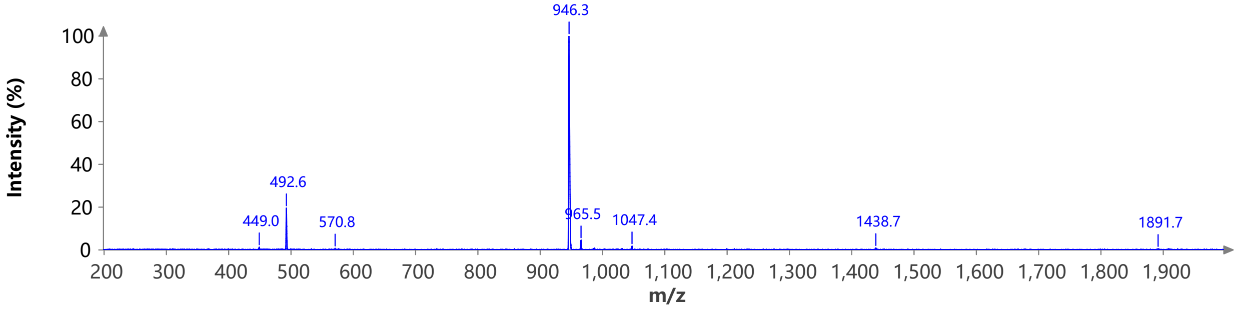

7-Hse^{2}NMe$_{2}$^{7}

UV 260.0 nm
JTmorti07_Hse2NMe7_lyo_UV.datx 2023.06.05 13:07:26;

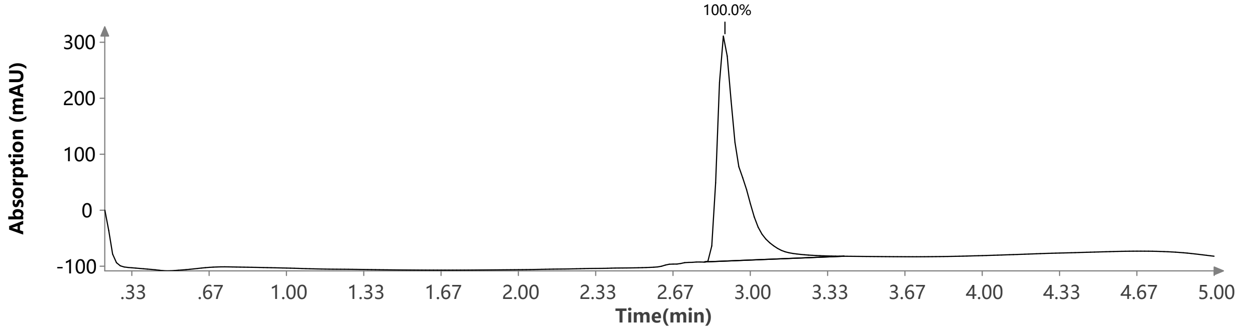

Spectrum RT 2.78 - 3.24 {132 scans}
JTmorti07_Hse2NMe7_lyo.datx;
ESI + Settings for tune mix using source type ESI Positive. Max: 3.1E6

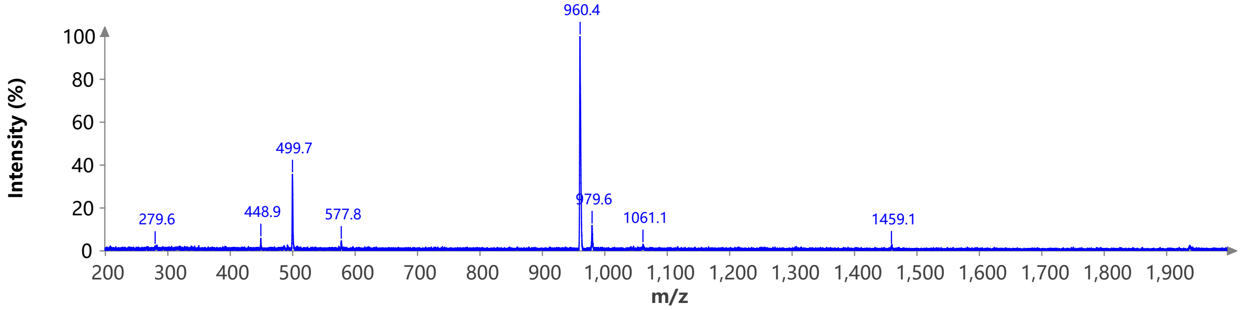

7-S^{4}NMe$_{2}$^{7}

UV 260.0 nm
JTmorti07_S4NMe7_lyo_UV.datx 2023.06.05 13:16:58;

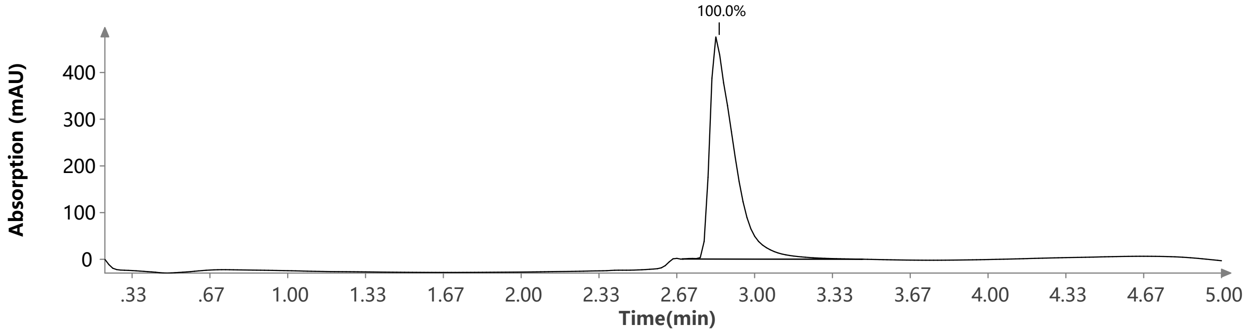

Spectrum RT 2.73 - 3.20 {135 scans}
JTmorti07_S4NMe7_lyo.datx;
ESI + Settings for tune mix using source type ESI Positive. Max: 3.4E6

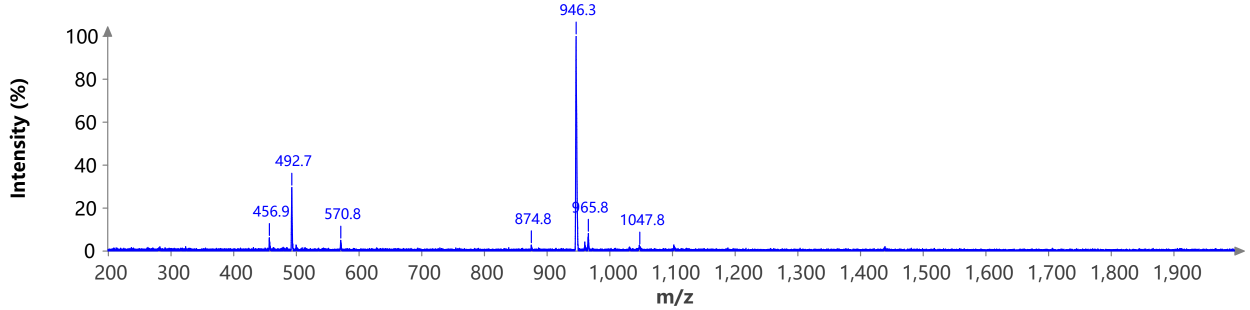

7-Hse^{4}NMe$_{2}$^{7}

UV 260.0 nm
JTmorti07_Hse4NMe7_lyo_UV.datx 2023.06.05 13:26:37;

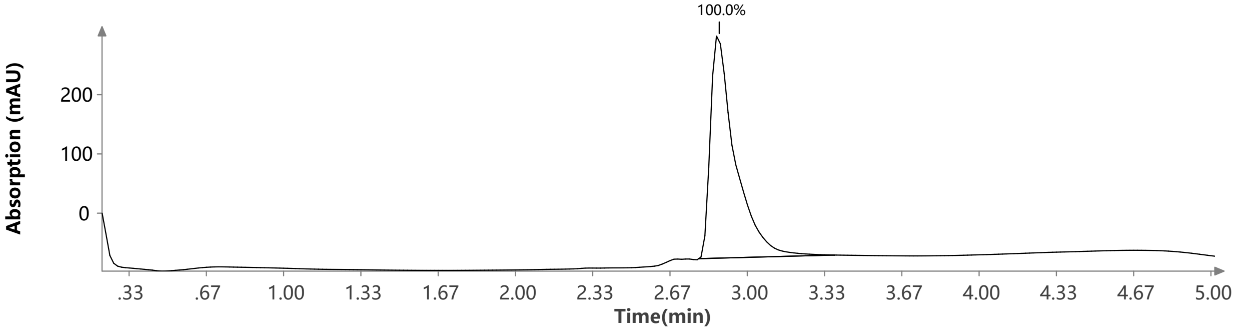

Spectrum RT 2.80 - 3.14 {100 scans}
JTmorti07_Hse4NMe7_lyo.datx;
ESI + Settings for tune mix using source type ESI Positive. Max: 4.4E6

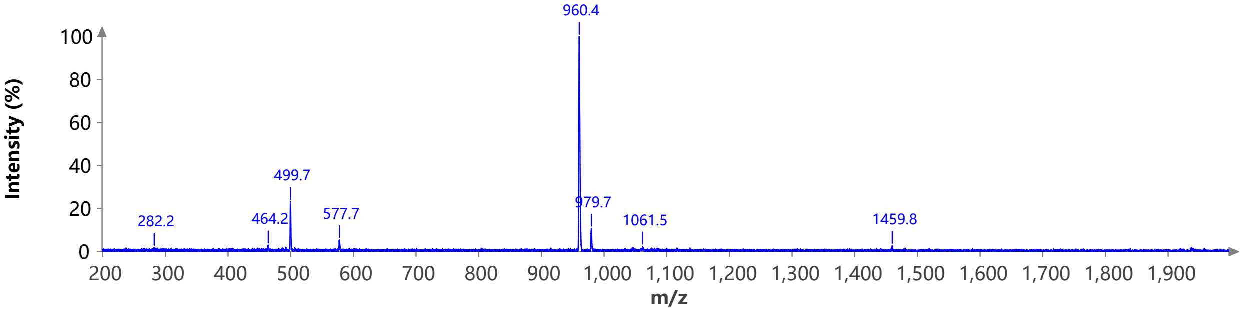

7-Thp^{3}

UV 260.0 nm
JTmorti07_Thp3_f3_UV.datx 2023.06.05 10:54:41;

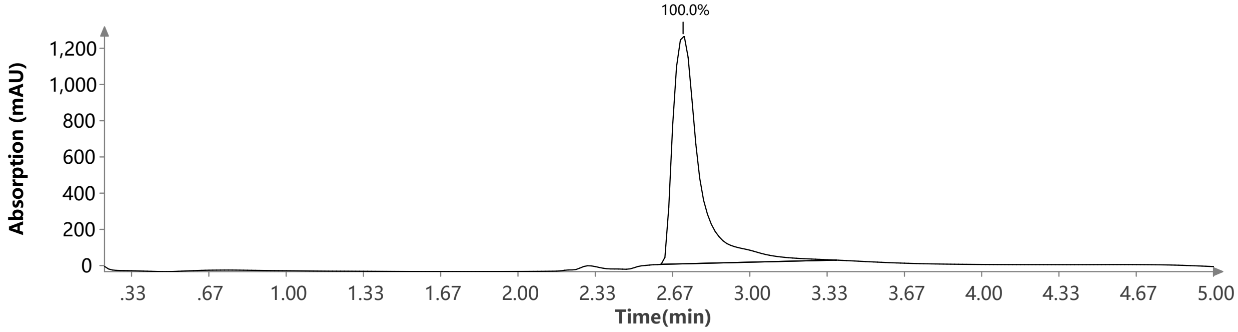

Spectrum RT 2.71 - 2.90 {55 scans}
JTmorti07_Thp3_f3.datx;
ESI + Settings for tune mix using source type ESI Positive. Max: 6.4E6

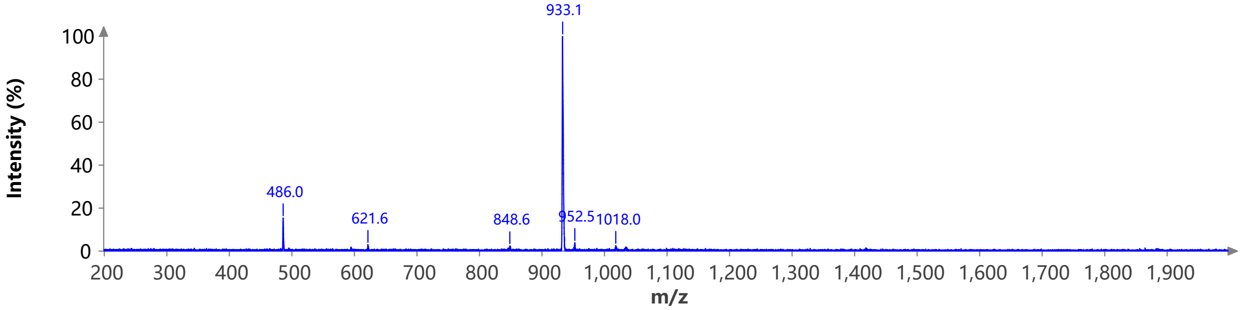

Morti01 enantiomer

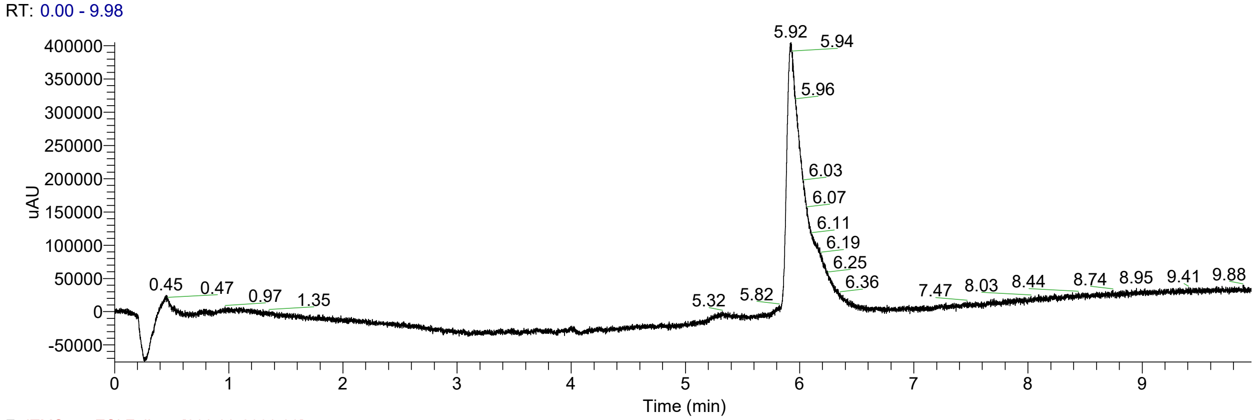

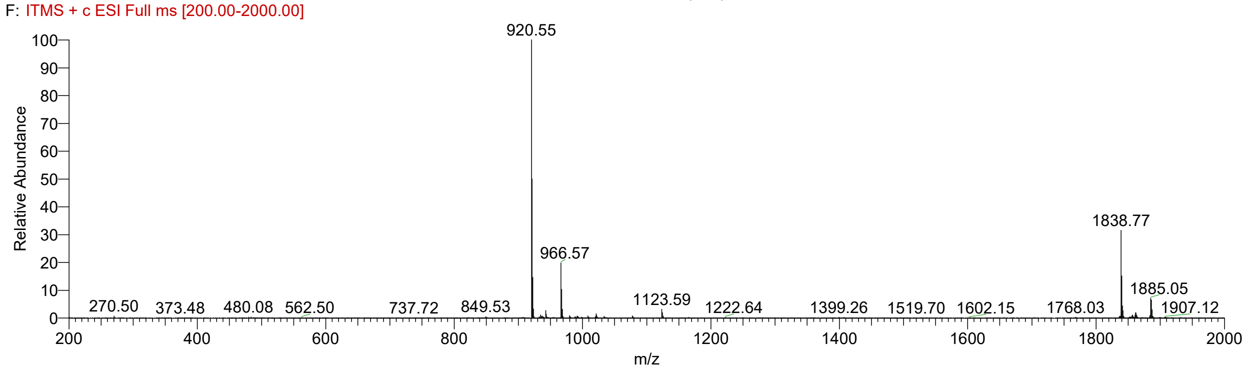

Morti03 enantiomer

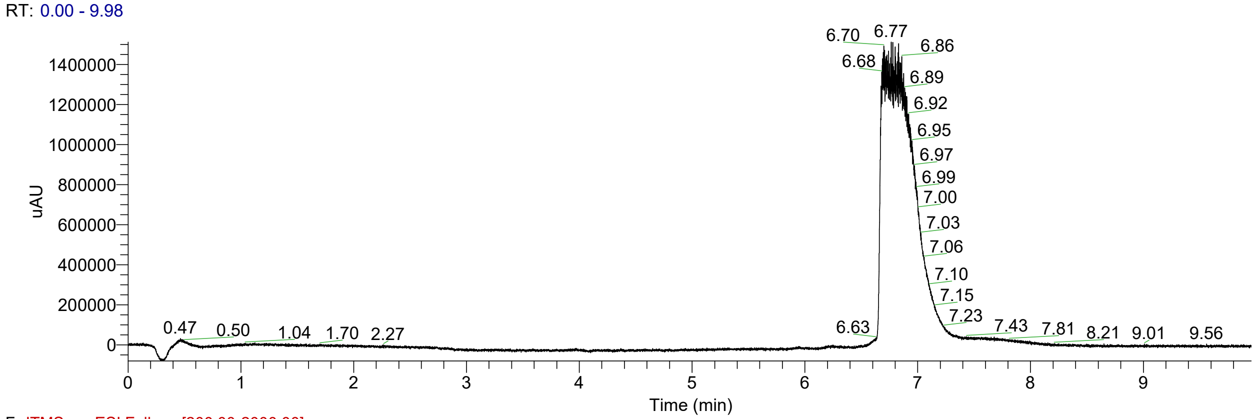

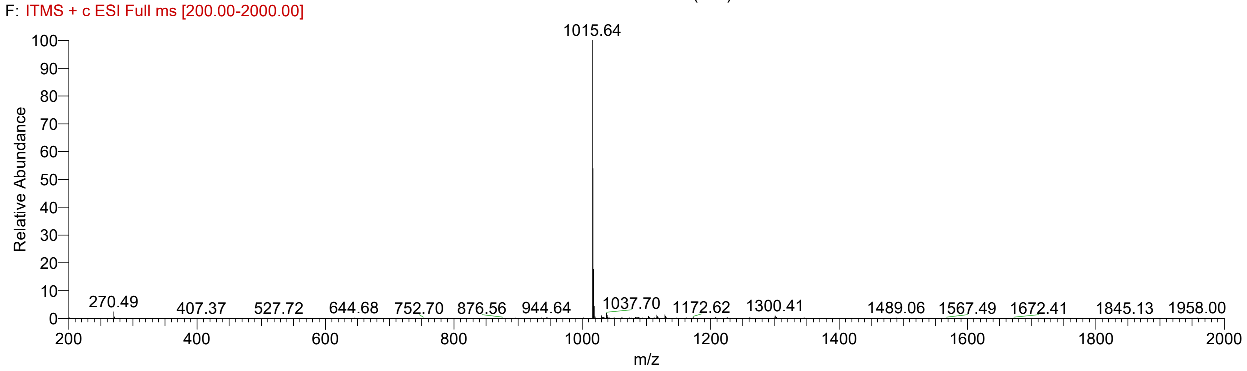

Morti04 enantiomer

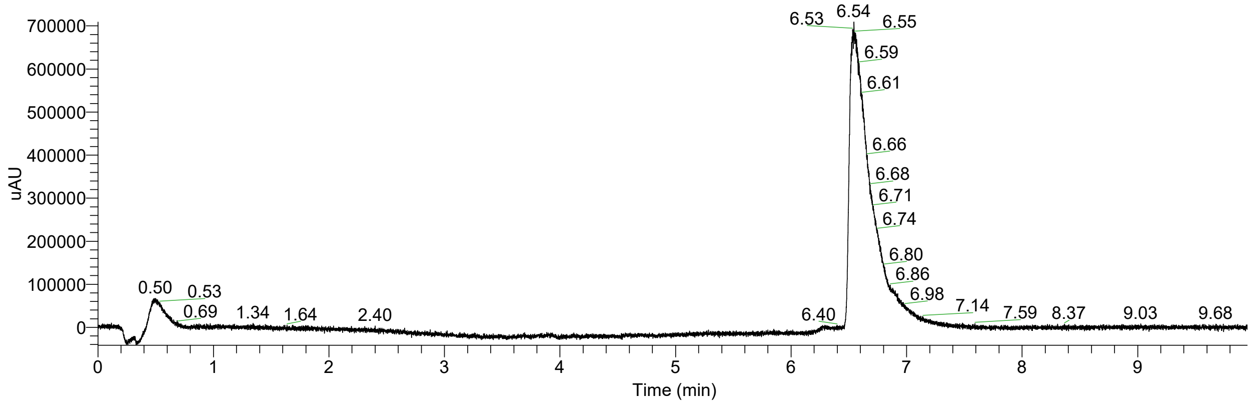

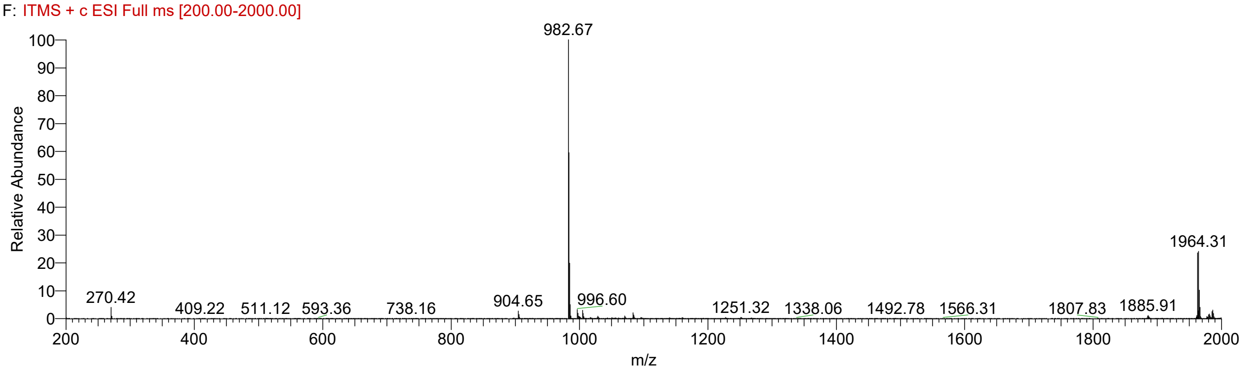

Morti06 enantiomer

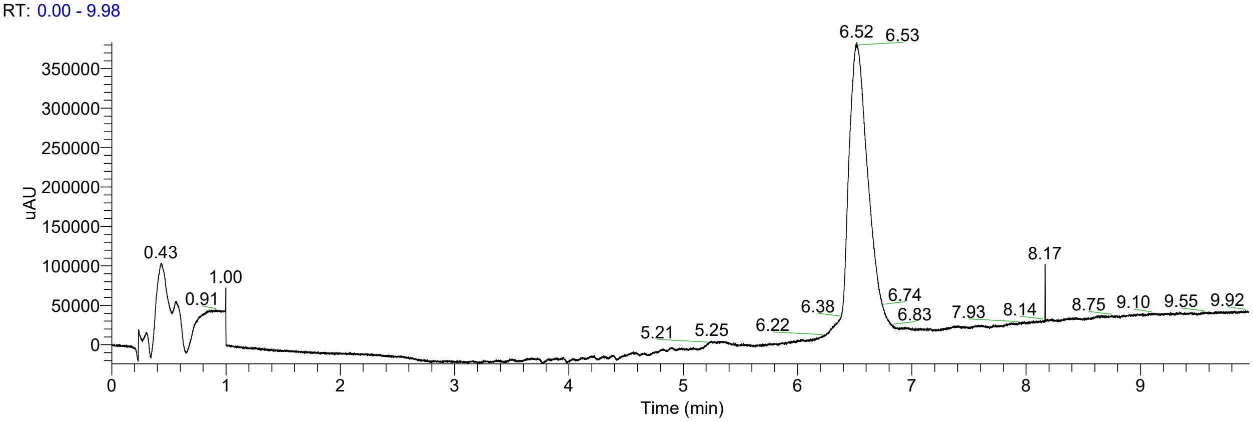

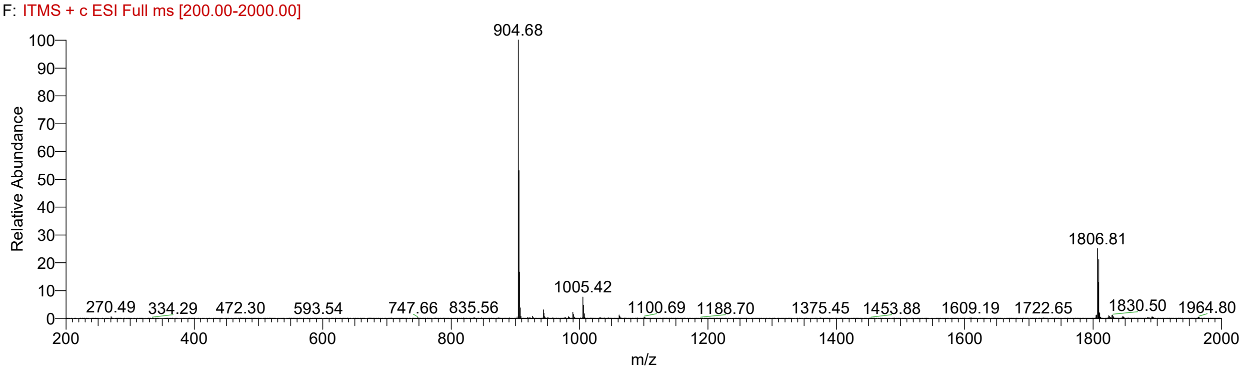

Morti07 enantiomer

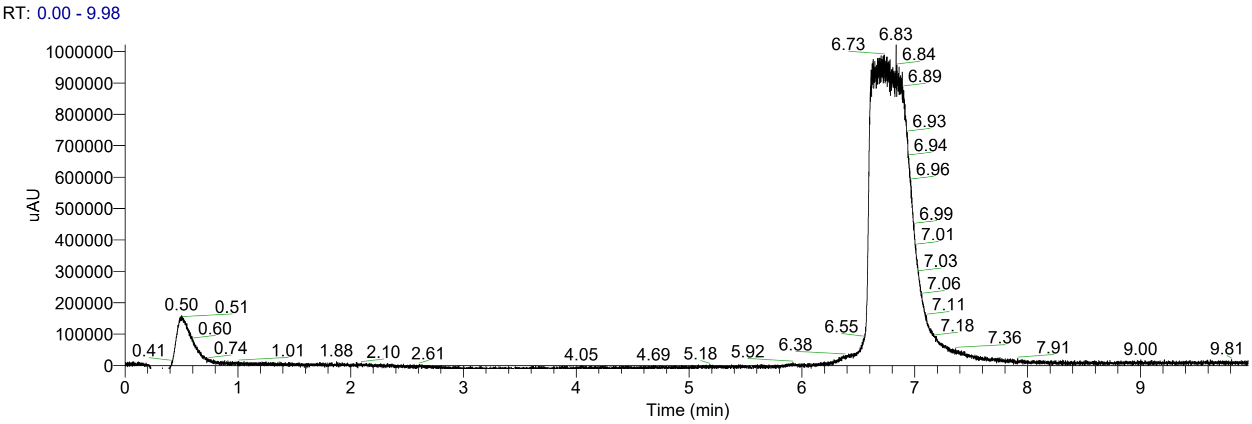

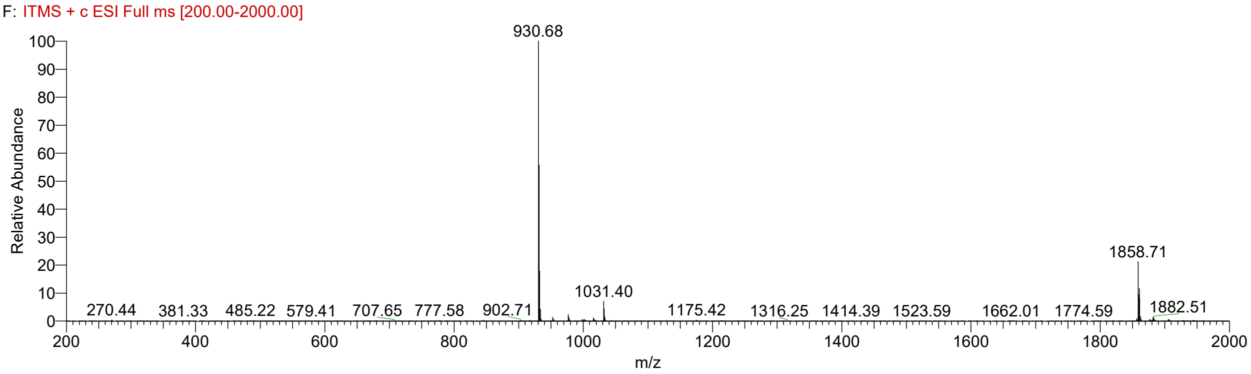

Morti09 enantiomer

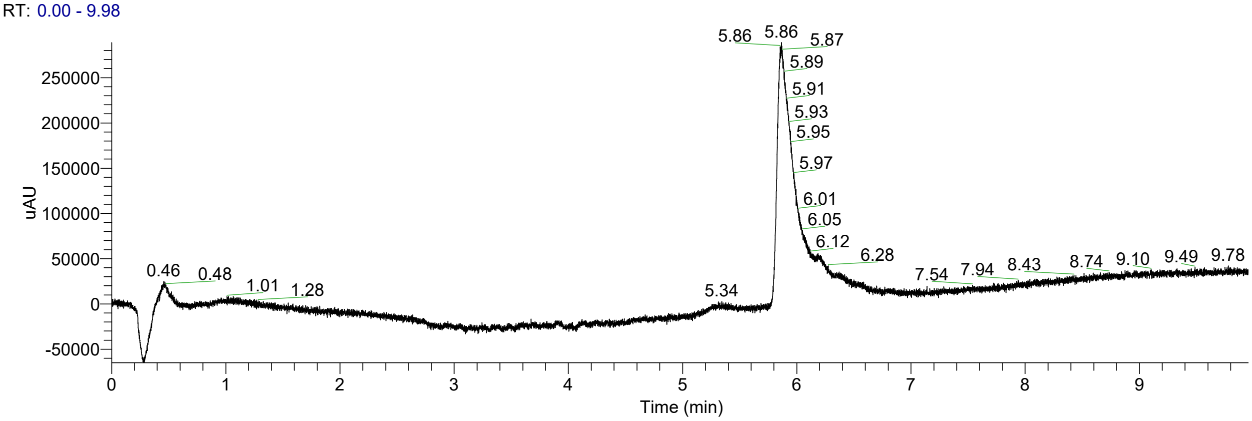

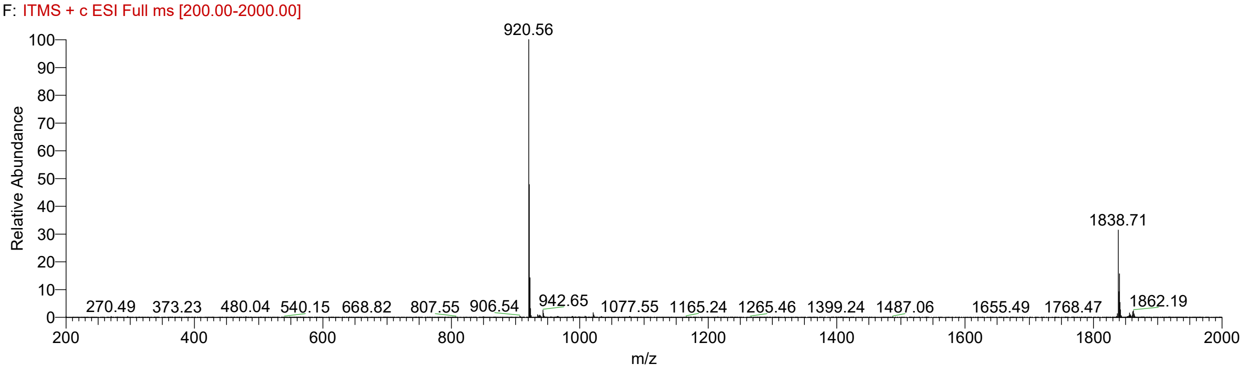

Morti10 enantiomer

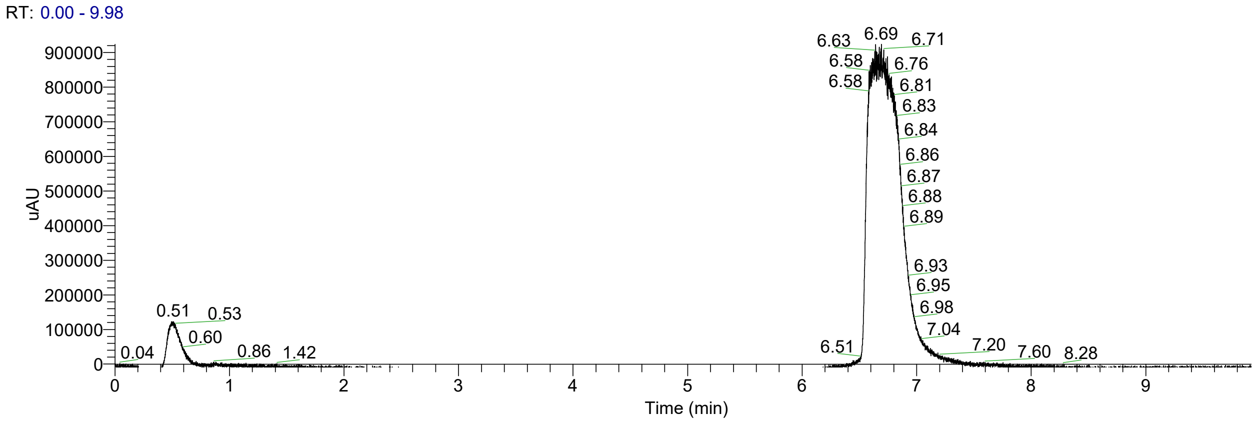

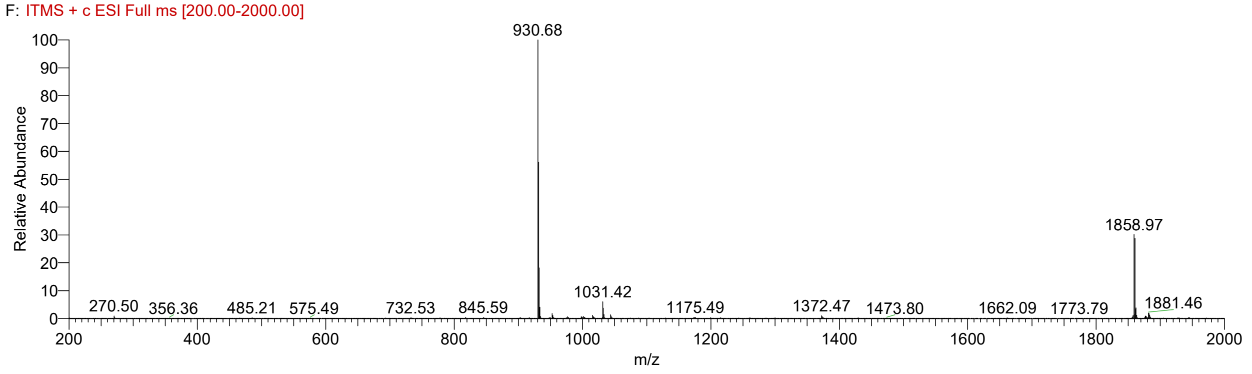

Morti11 enantiomer

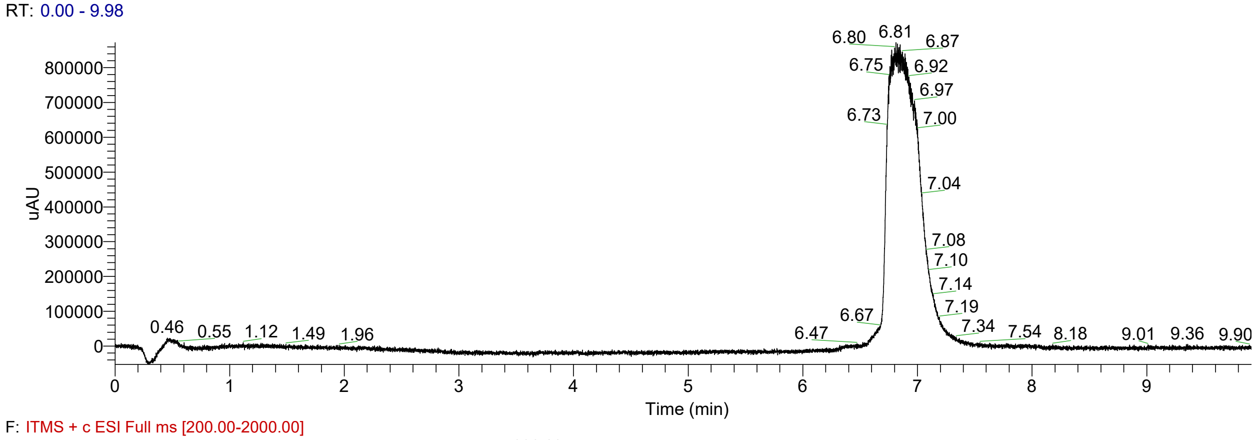

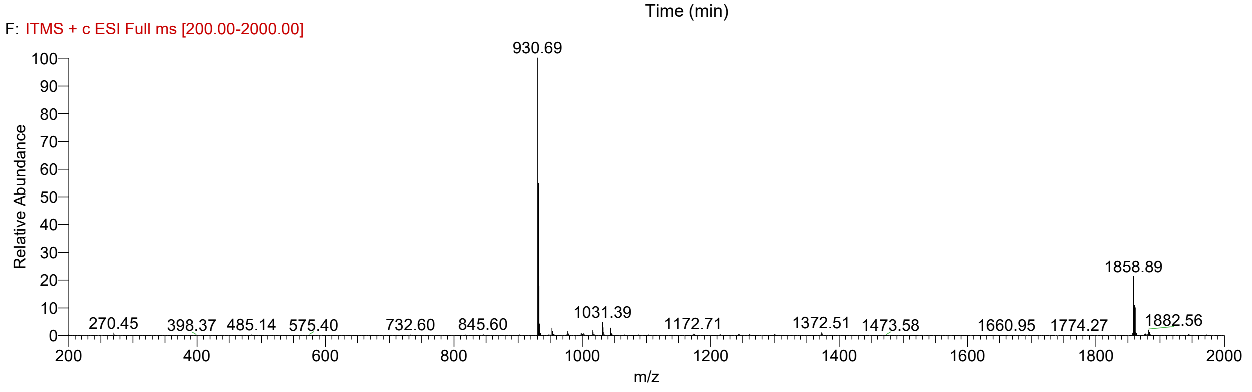

Morti13 enantiomer

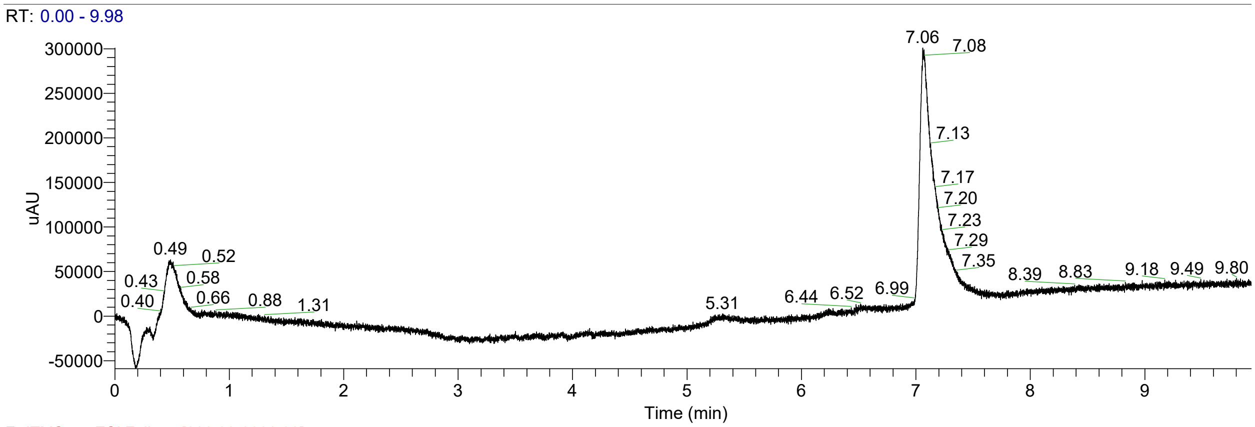

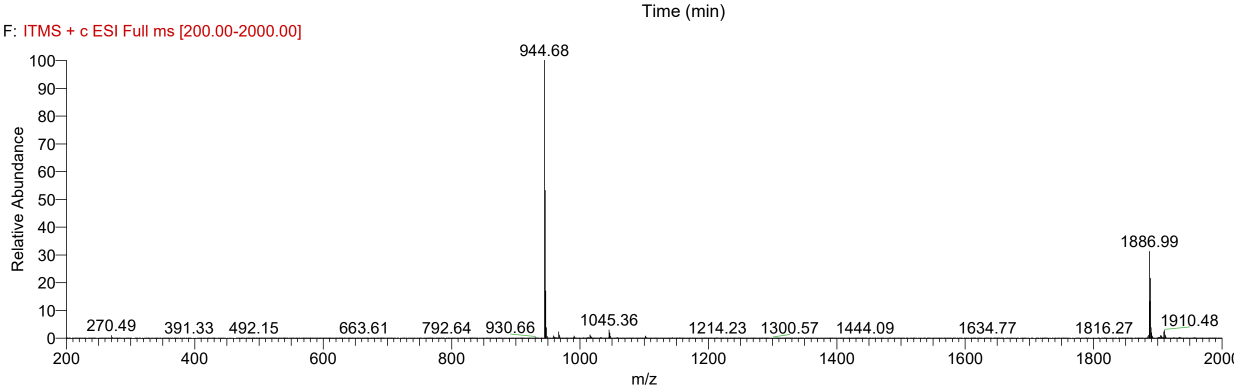

1E6 enantiomer

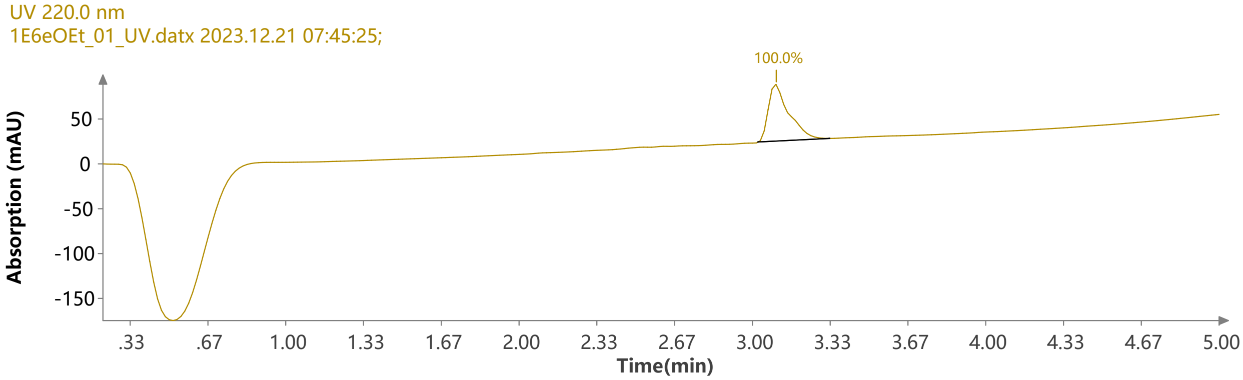

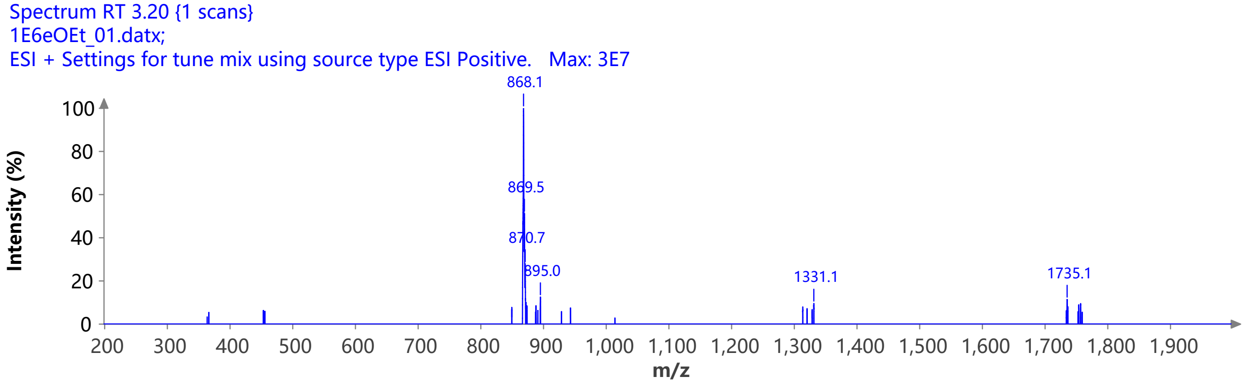

1E7 enantiomer

UV 200.0 nm
1E7eOEt_01_UV.datx 2023.12.21 08:22:25;

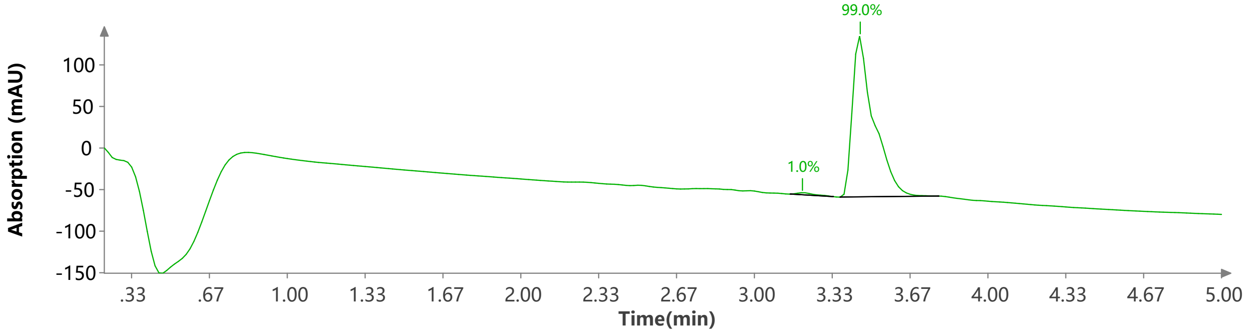

Spectrum RT 3.46 - 3.61 {43 scans}
1E7eOEt_01.datx;
ESI + Settings for tune mix using source type ESI Positive.   Max: 1.9E7

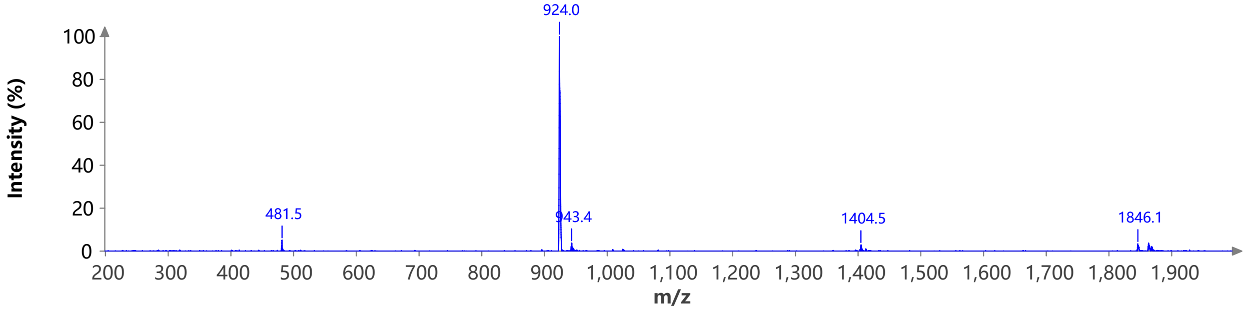

2A8 enantiomer

UV 220.0 nm
2A8eOEt_01_UV.datx 2023.12.21 07:54:40;

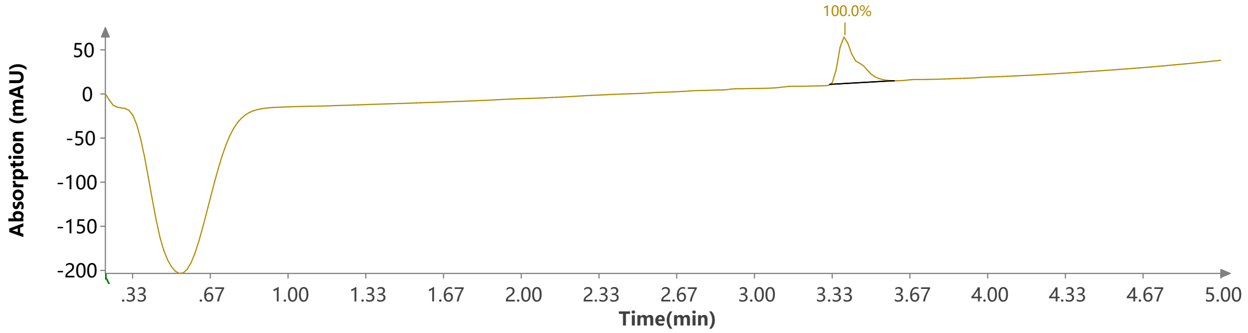

Spectrum RT 3.37 - 3.64 {79 scans}
2A8eOEt_01.datx;
ESI + Settings for tune mix using source type ESI Positive.   Max: 8.7E6

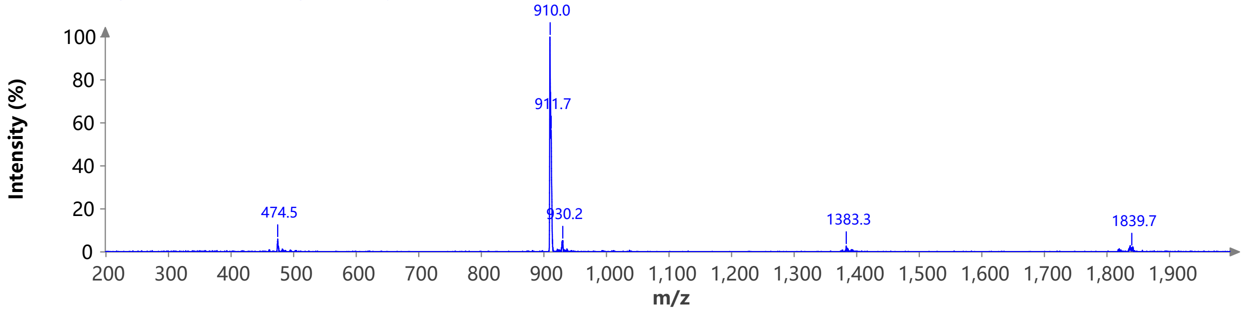

2A11 enantiomer

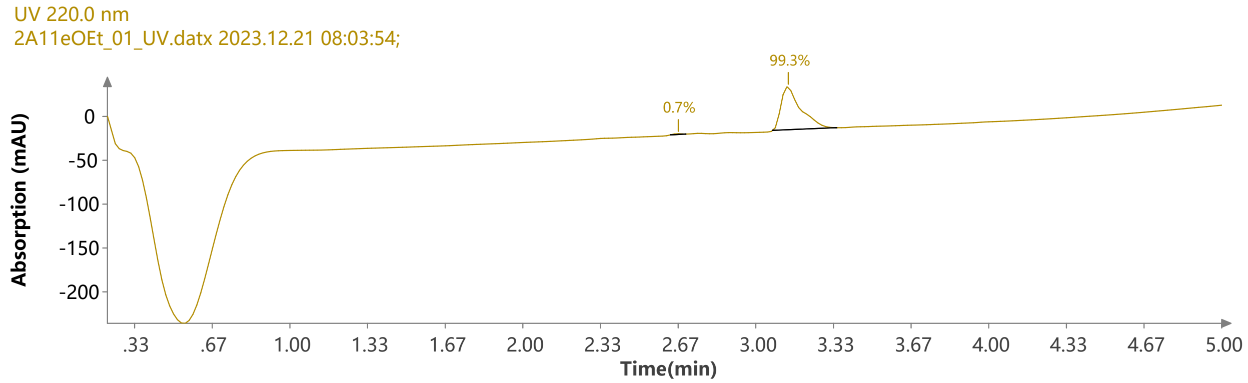

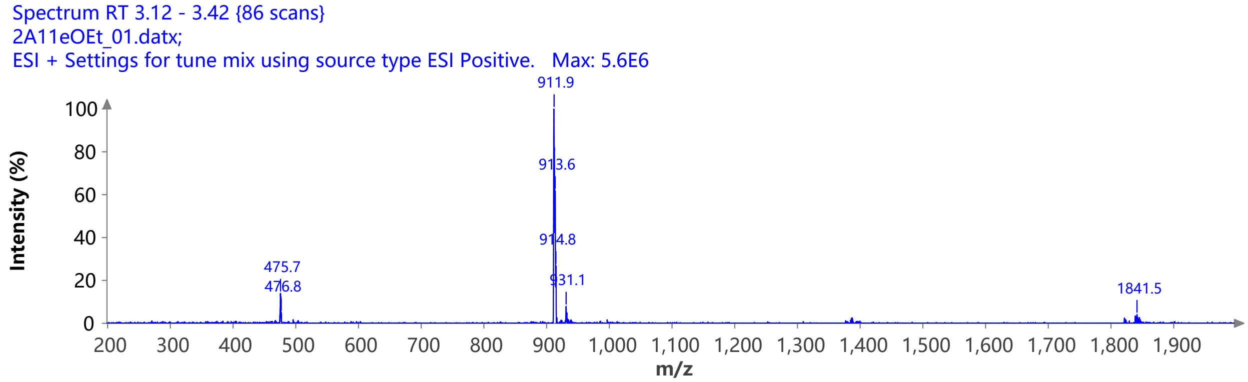

2E5 enantiomer

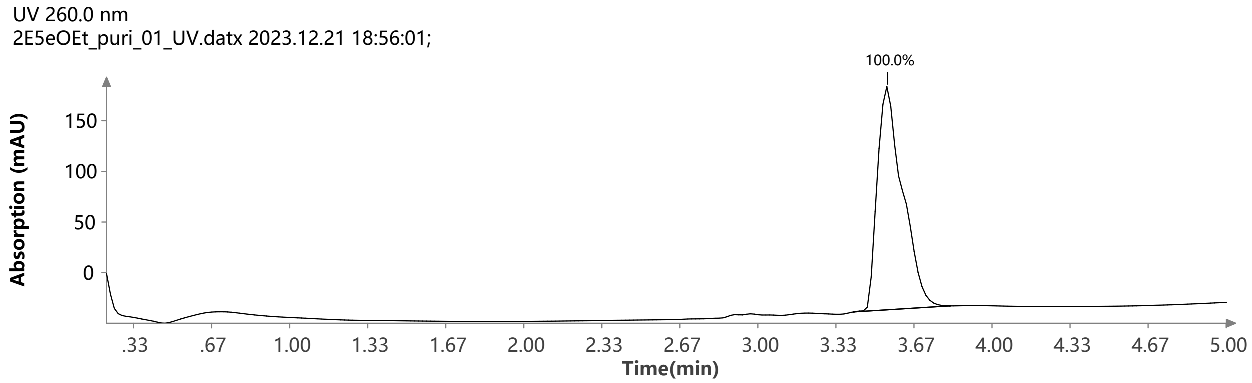

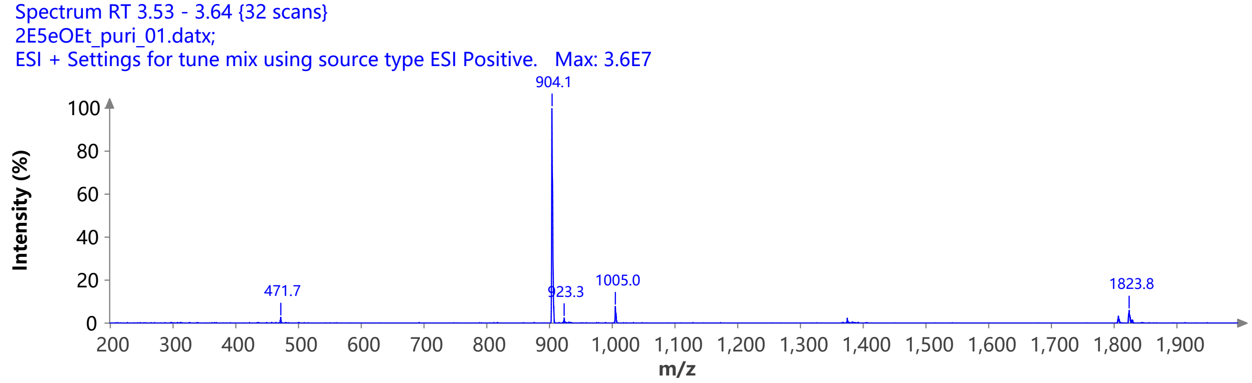

Morti07 epimer

ELSD
7inv-3_f06.datx 2024.10.22 17:11:00 7 mins 200-2000 positive mode;
ESI + Settings for tune mix using source type ESI Positive.

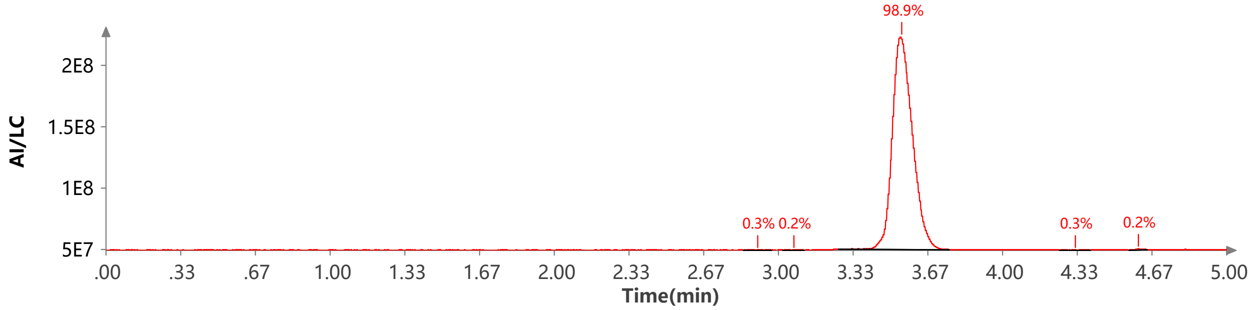

Spectrum RT 3.59 - 3.87 {80 scans}
7inv_f04_01.datx;
ESI + Settings for tune mix using source type ESI Positive. Max: 3.6E7

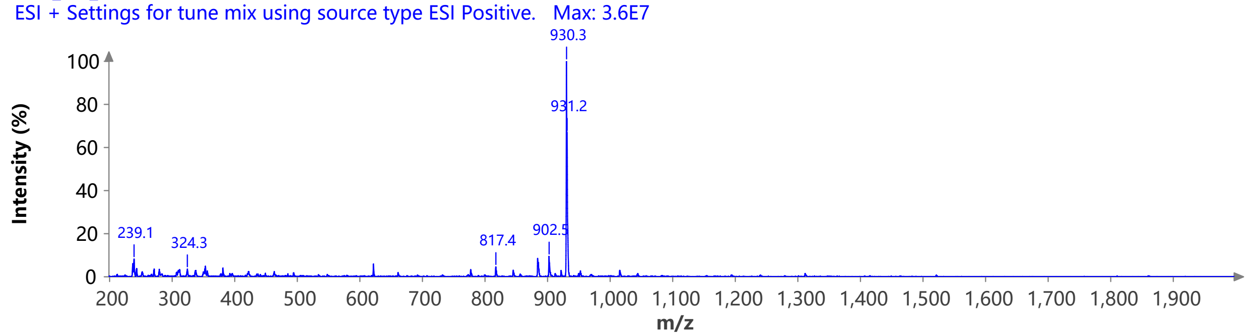

7-A⁶ epimer

ELSD
7A6_inv-s.datx 2025.07.06 13:14:04 7 mins 200-2000 positive mode;
ESI + Settings for tune mix using source type ESI Positive.

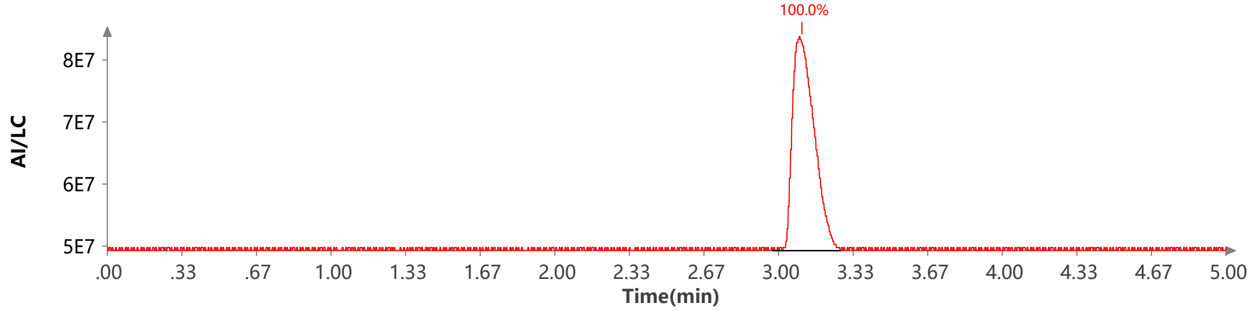

Spectrum RT 2.85 - 3.13 {80 scans}
7A6inv_lyo_01.datx;
ESI + Settings for tune mix using source type ESI Positive. Max: 6.8E7

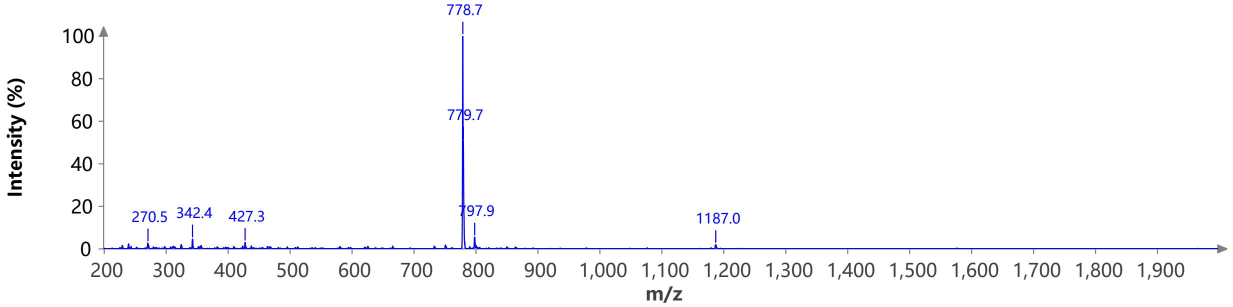

1E6 epimer

ELSD
1E6_inv-s.datx 2025.07.06 12:55:53 7 mins 200-2000 positive mode;
ESI + Settings for tune mix using source type ESI Positive.

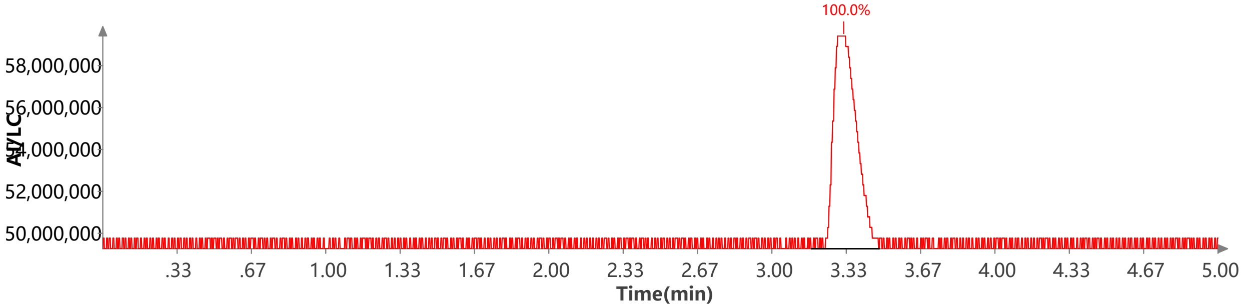

Spectrum RT 3.07 - 3.33 {73 scans}
1E6inv_f06_01.datx;
ESI + Settings for tune mix using source type ESI Positive. Max: 1.8E7

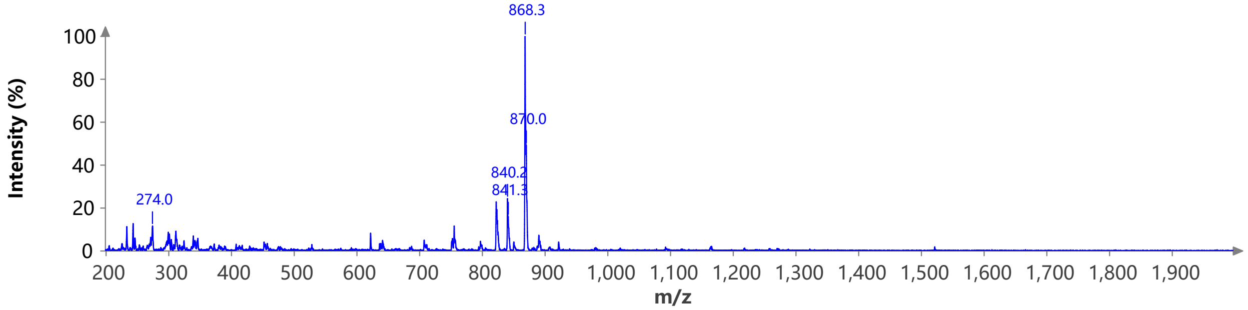

2B3 epimer

ELSD
2B3_inv-s.datx 2025.07.06 13:04:59 7 mins 200-2000 positive mode;
ESI + Settings for tune mix using source type ESI Positive.

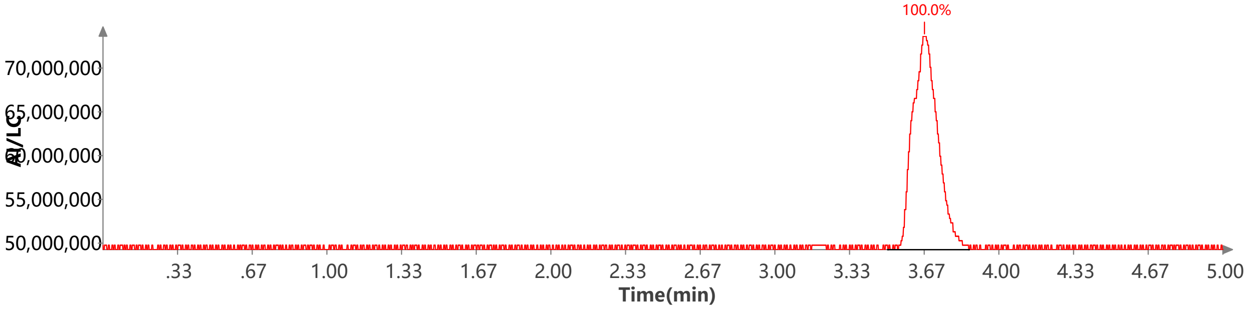

Spectrum RT 3.41 - 3.65 {69 scans}
2B3inv_f13_01.datx;
ESI + Settings for tune mix using source type ESI Positive. Max: 2.9E7

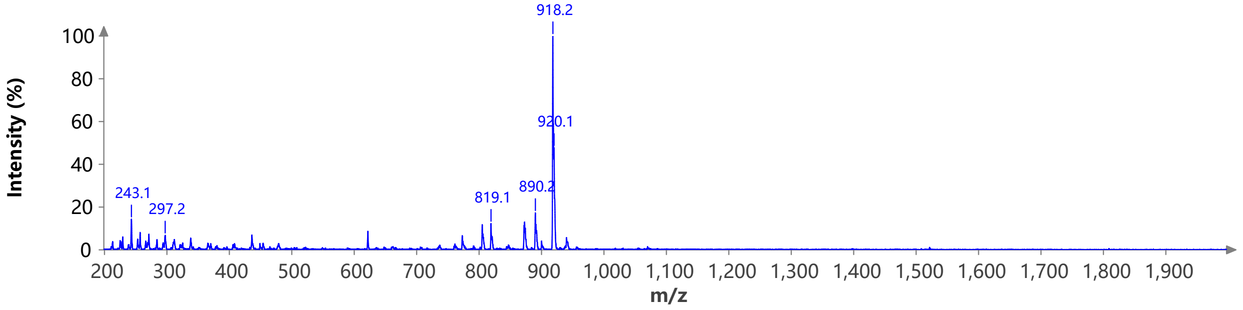

Mortiamide A

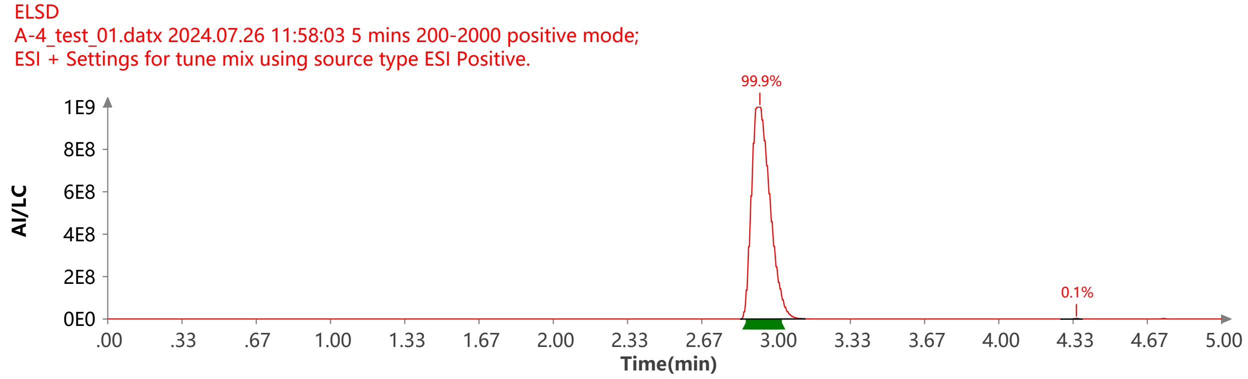

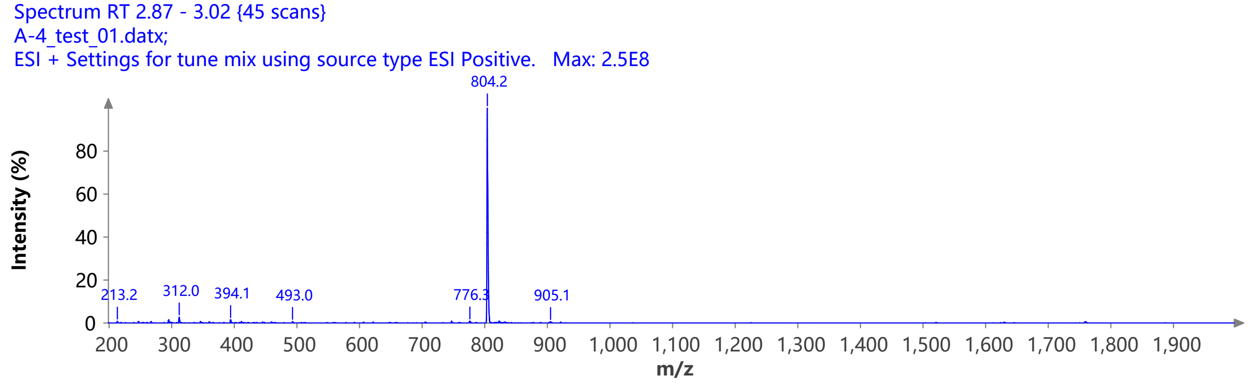

Mortiamide A enantiomer

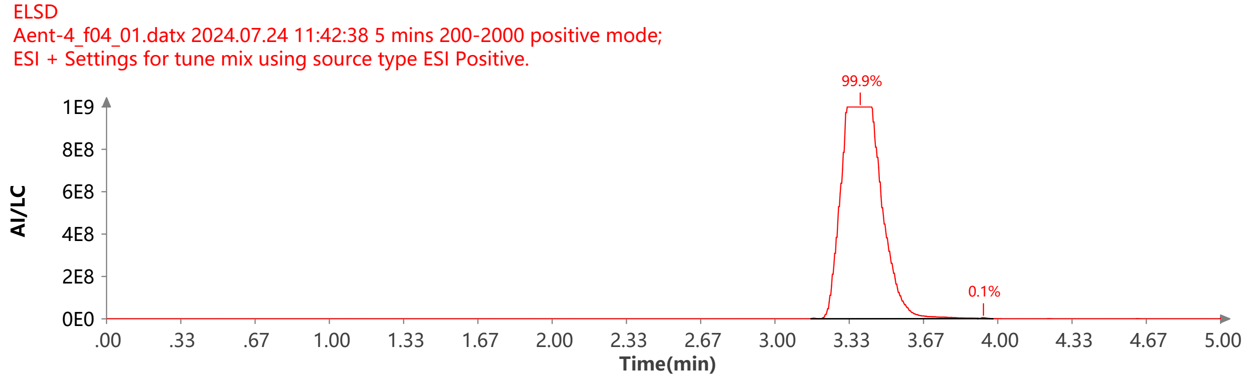

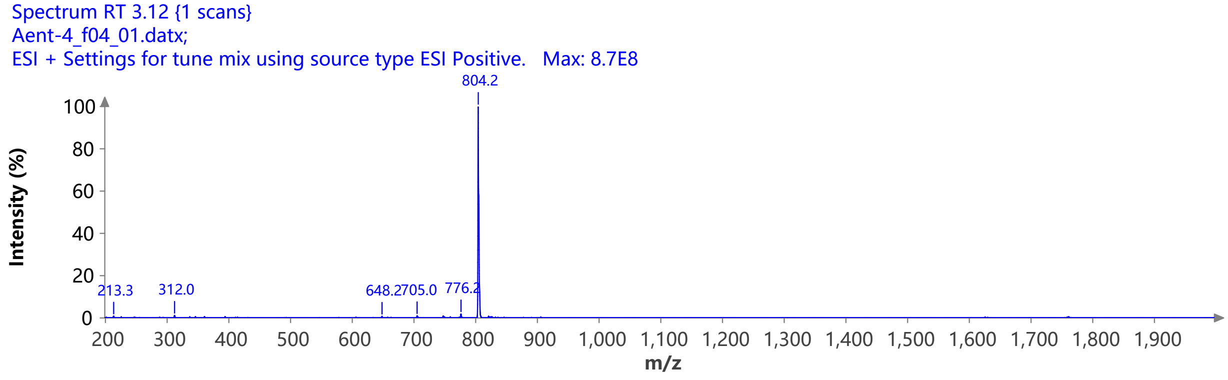

Mortiamide A epimer

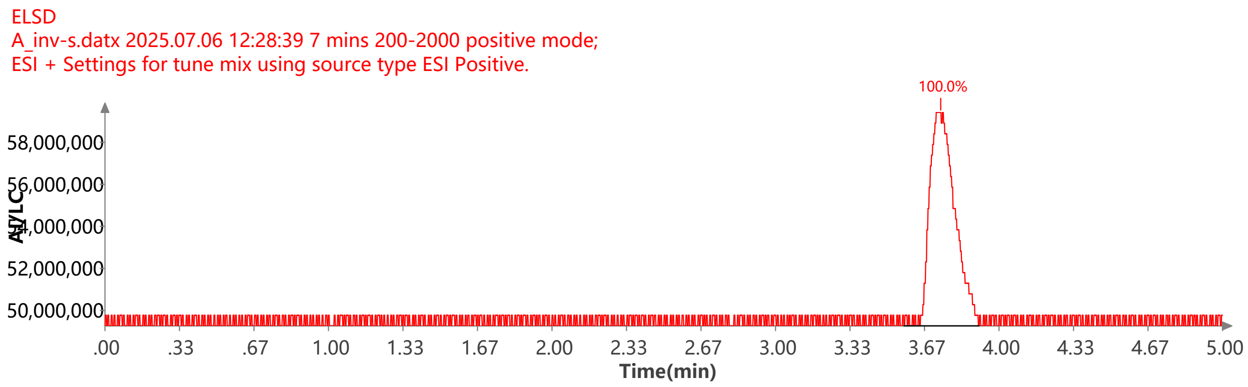

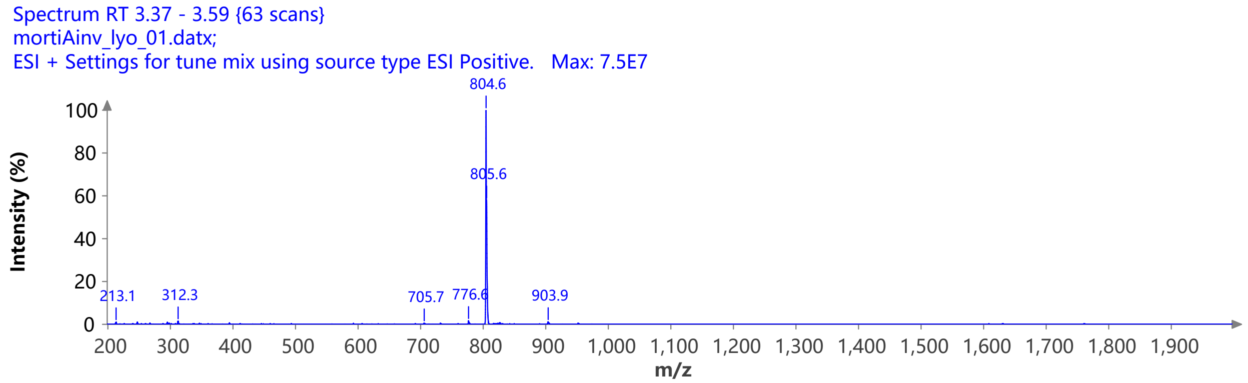

Mortiamide B

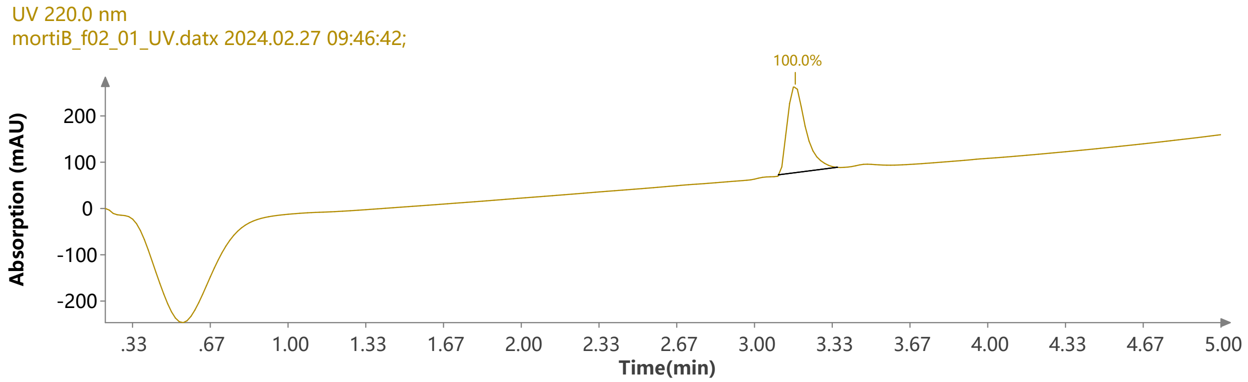

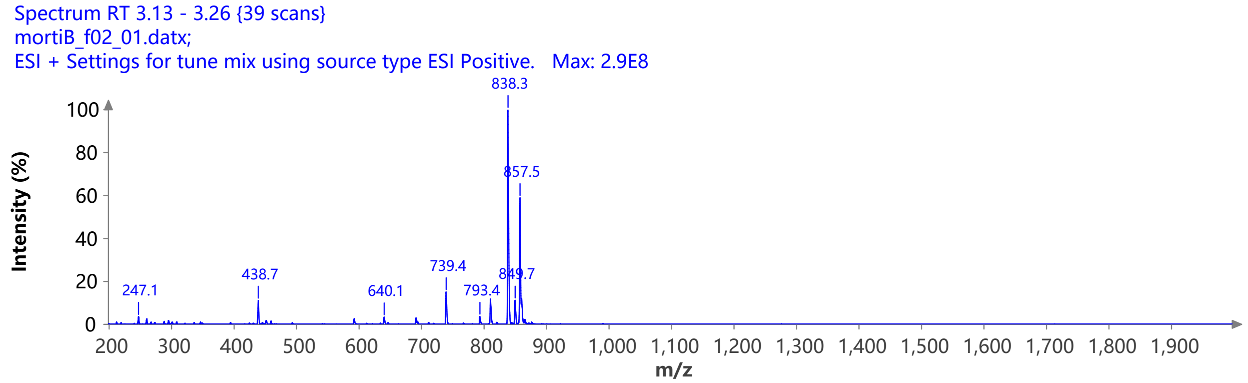

Mortiamide B enantiomer

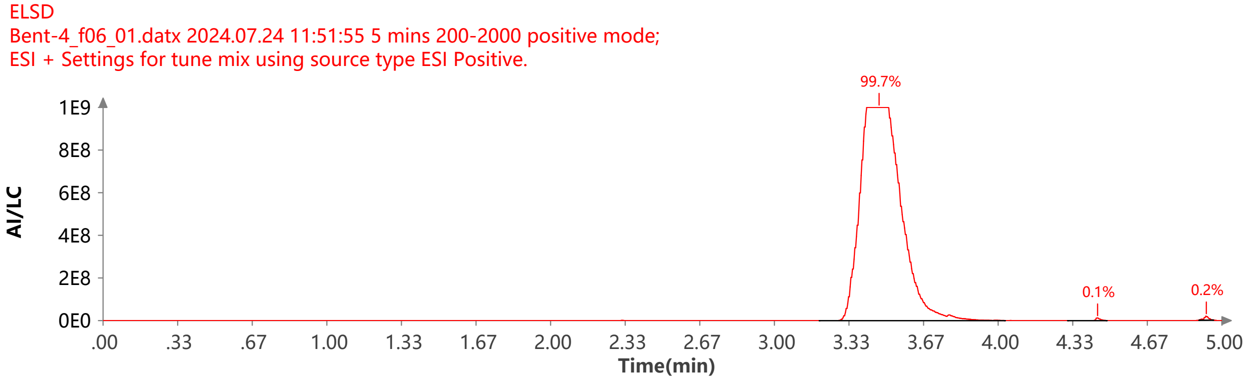

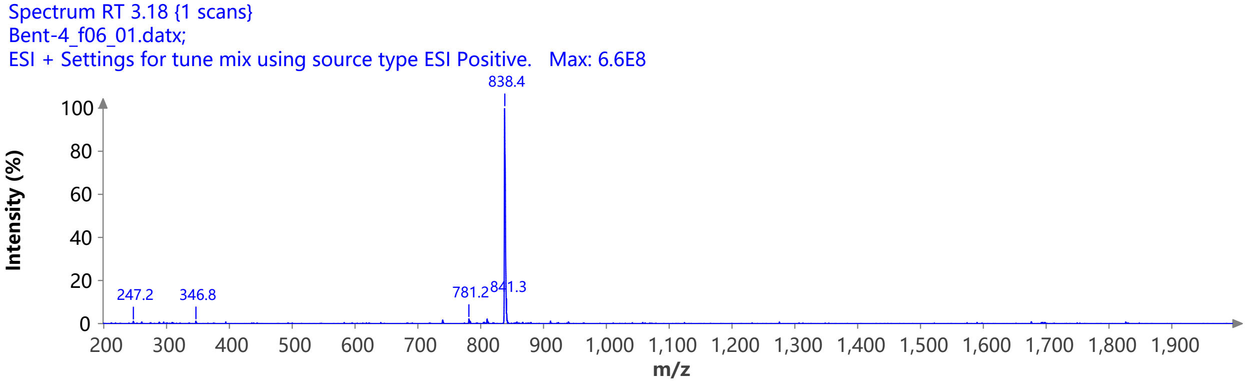

Mortiamide B epimer

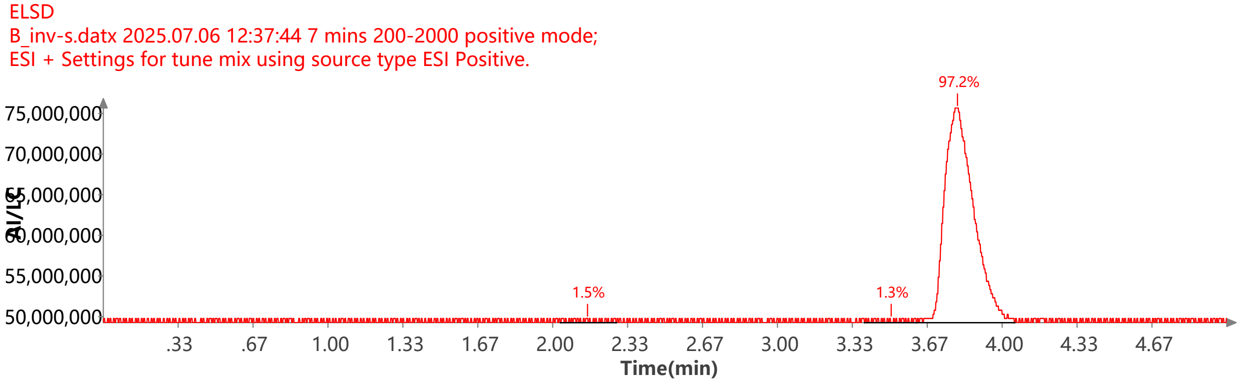

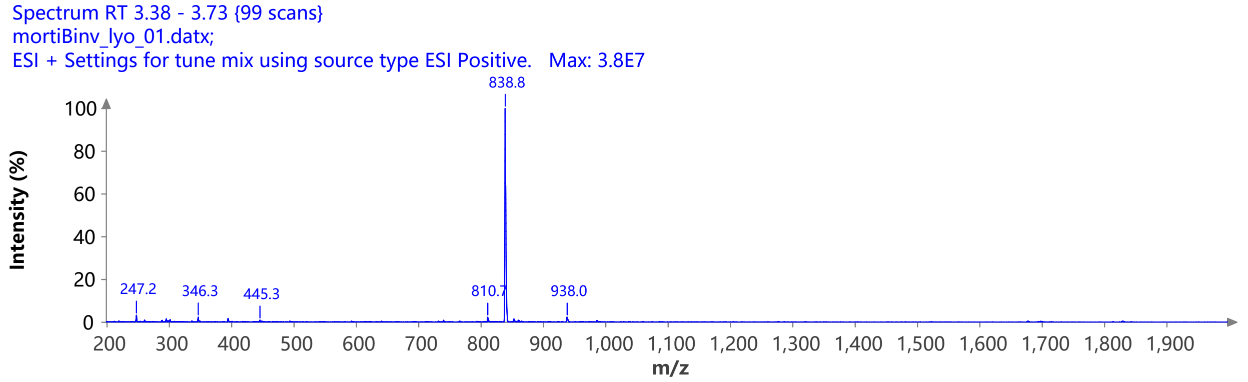

Mortiamide D

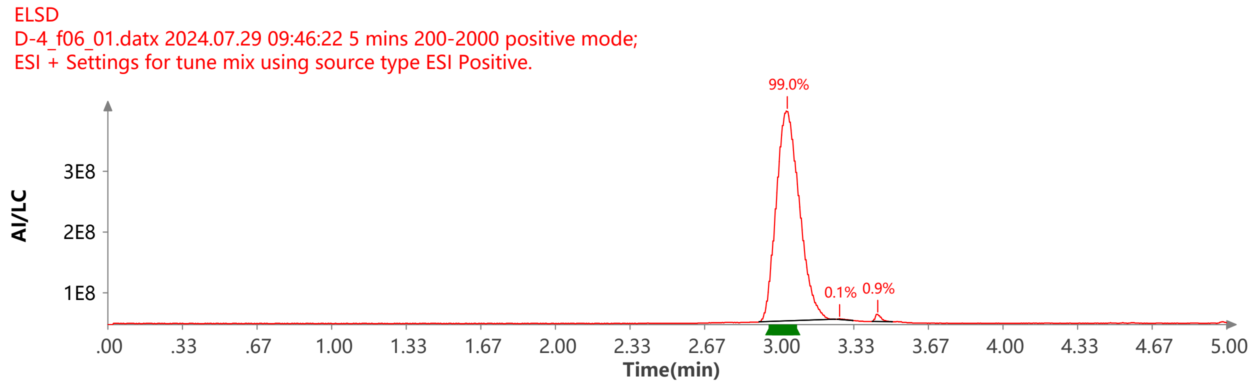

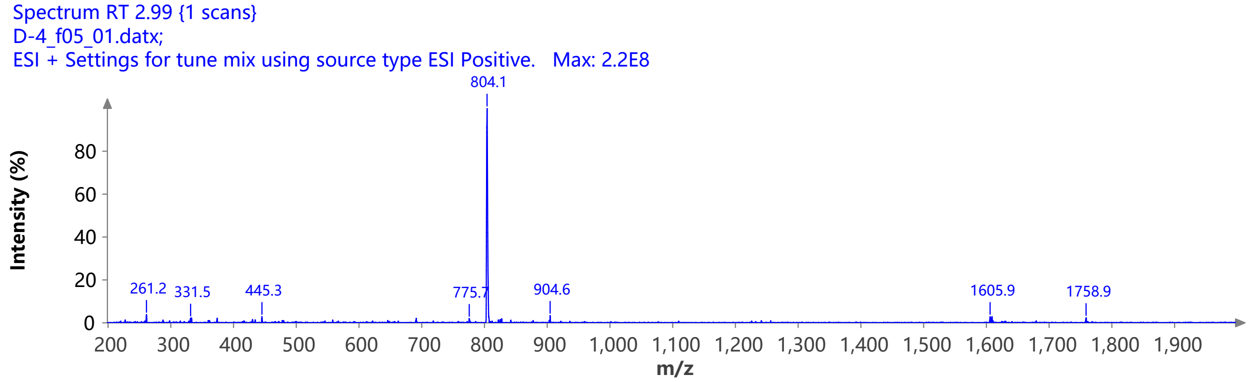

Mortiamide D enantiomer

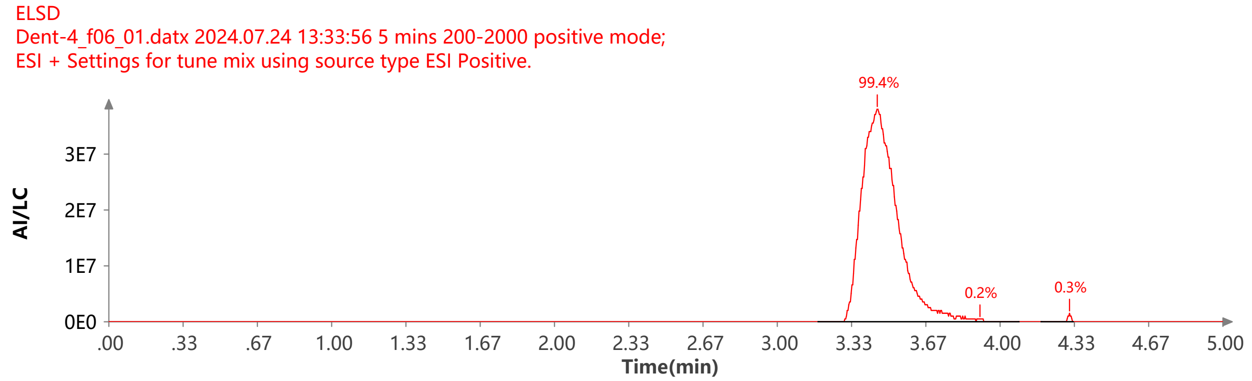

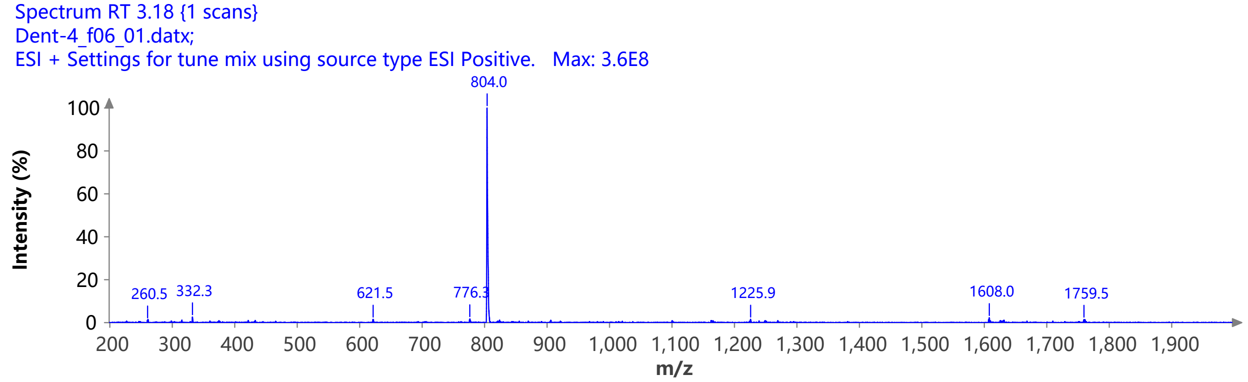

Mortiamide D epimer

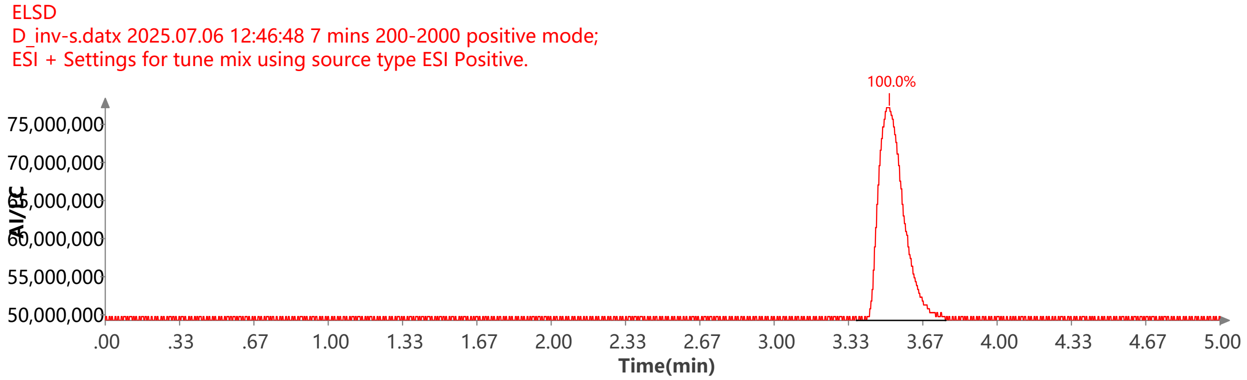

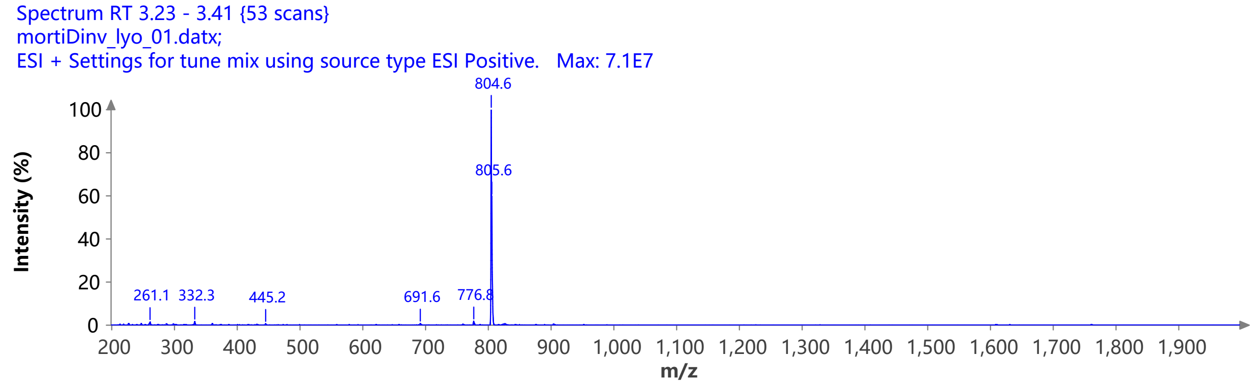

References

[1] G. Sheldrick, *Acta Crystallogr. Sect. A* **2015**, *71*, 3-8.
[2] G. Sheldrick, *Acta Crystallogr. Sect. C* **2015**, *71*, 3-8.
[3] P. Müller, *Crystallogr. Rev.* **2009**, *15*, 57-83.
